# Copper-catalyzed direct regioselective C5–H alkylation reactions of functionalized indoles with α-diazomalonates†

**DOI:** 10.1039/d5sc03417e

**Published:** 2025-07-15

**Authors:** Tomohiro Isono, Shingo Harada, Mai Yanagawa, Tetsuhiro Nemoto

**Affiliations:** a Graduate School of Pharmaceutical Sciences, Chiba University Chuo-ku Chiba 260-8675 Japan Sharada@chiba-u.jp tnemoto@faculty.chiba-u.jp

## Abstract

Functionalization of the pyrrole nucleus in indoles has been extensively investigated, whereas benzenoid ring functionalization studies remain scarce. Advances in direct C–H functionalization have been hindered by the inherent low reactivity and need for precise control of regioselectivity. Herein, we developed regioselective C5–H alkylation reactions of indoles bearing carbonyl functionalities at the C3 position using copper-carbene species. This methodology regioselectively affords functionalized heteroarenes bearing synthetically useful structural motifs characteristic of bioactive molecules and natural products. Experimental and computational mechanistic findings underscored the pivotal importance of the copper catalyst system and rationalized the origin of regioselectivity.

## Abstract 2

Remote C5-selective C–H functionalization and mechanistic investigation.

## Graphical abstract

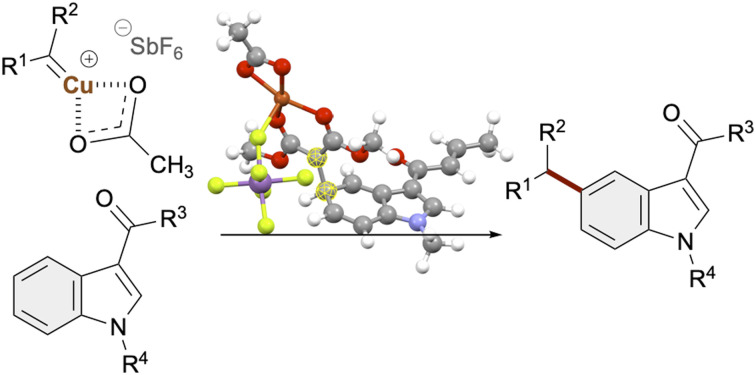

## Introduction

**Fig. 1:**
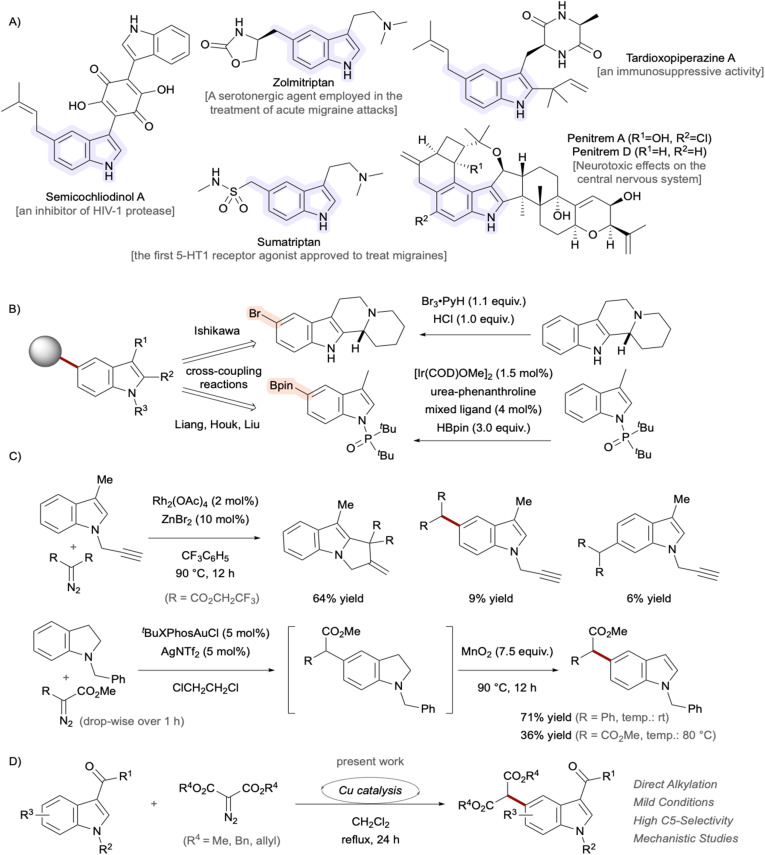
(A) Representative bioactive indole derivatives bearing alkyl substituents at the C5 position. (B) Established stepwise methodologies for accessing C5-functionalized indoles. (C) Reported reactions between indole/indoline derivatives and metal-carbene species. (D) Direct C5–H alkylation under copper catalysis.

The chemical structure 2,3-benzopyrrole, known as indole, possessing 10 π-electrons serves as a fundamental core architecture in numerous natural products, pharmaceutical drugs, and functional materials.^1^ Among the various substitution patterns on the essential scaffold, 5-substituted indole derivatives have attracted considerable attention in recent years due to their unique biological activities and synthetic challenges in organic chemistry.^2^ Notable examples of C5-functionalized indoles^3,4^ include the natural products semicochliodinol A,^5,6^ isolated from rice cultures of the fungus genus *Chaetomium*, and penitrem D,^7^ isolated from *Penicillium crustosum*, as well as the therapeutic agent sumatriptan,^8^ used to treat migraine headaches.^9,10^ The C2 and C3 positions of indole exhibit pronounced reactivity, enabling a diverse array of chemical modifications. Conversely, straightforward synthetic methodologies for functionalizing the less reactive C4 to C7 positions are limited.^11^ Functionalization at the C4–H or C7–H positions of indoles can be achieved through strategic placement of directed metalation groups at the C3 position or the indole nitrogen, respectively. Direct modification of the C5 and C6 positions, however, remains a formidable synthetic challenge.^12^ In 2024, Ishikawa *et al.* successfully accomplished a highly selective bromination of indolo[2,3-*a*]quinolizidine through a unique mechanistic pathway involving the transient *in situ* generation of aniline derivatives (Fig. 1A).^13^ More recently, Liang *et al.* made a groundbreaking contribution by reporting the iridium-catalyzed C5-borylation of indoles, succeeding in the theoretical design of ligands that induce multiple non-covalent interactions, particularly dispersion forces (Fig. 1B).^2^ In these strategies, the installed halogen^14^ or boron functionalities served as valuable handles for further diversification through various coupling reactions.^15–17^ As a landmark work, Shi *et al.* established catalyst-controlled direct C4–H and C5–H arylation reactions of indoles bearing a removable pivaloyl group at the C3 position.^18^ The highly efficient strategy enables regioselective construction of C(sp^2^)–C(sp^2^) bonds and can serve as a powerful synthetic platform for the assembly of pharmaceutically relevant scaffolds. On the other hand, successful C5-selective alkylation for constructing C(sp^3^)–C(sp^2^) bonds remains limited,^19–21^ often producing only modest to low yields^22,23^ with poor regioselectivity.^24,25^ Regiocontrol can be achieved through intramolecular reactions,^26–29^ but more general and versatile methodologies are needed. Although direct arene alkylation utilizing carbene species^30–35^ represents a powerful synthetic strategy,^36–38^ achieving precise regioselectivity control remains a significant challenge (Fig. 1C).^39^ As an alternative approach, a strategy proceeding through indoline intermediates was developed by exploiting the *para*-directing nature of aniline derivatives, followed by dehydrogenative reoxidation to indoles.^40,41^ Within the framework of our research program,^42–44^ we focused on carbene chemistry^45^ to develop selective transformations for synthesizing high-value molecules.^46^ Our group demonstrated the C4-selective functionalization of indoles employing rhodium-carbene species.^47^ α,β-Unsaturated enones at the C3 positions were utilized as both directing groups and enophiles. Building upon this study, further optimization of the catalytic system enabled remote C5–H alkylation using the same substrate (Fig. 1D). Mechanistic studies indicated that Cu(OAc)(SbF_6_) is the actual active catalyst for carbene-transfer reactions.^48–52^ Copper presents an attractive alternative to precious metals in synthetic chemistry due to its environmentally friendly nature, low cost, and abundance.^53–56^ Herein, we describe the development of a regioselective C5–H functionalization protocol of indoles with α-diazomalonates as carbene sources. Computational studies were also performed to shed light on the origin of the regioselectivity.

## Results and discussion

We initiated our investigation using *N*-benzyl indole 1a bearing an enone functionality as the model substrate, along with dimethyl α-diazomalonates 2a (Table 1). At the outset, a combination of rhodium(ii), copper(ii), and silver(i) salts was examined based on previous studies, leading to the formation of the C5–H functionalized product 3a in 18% yield (entry 1).^47^ Trace amounts of the C4–H alkylated product (4a) and its cyclized derivative (5a) were also detected in the crude NMR analysis. To identify the catalytically active species, individual metal salts were examined separately; however, none of the reactions afforded the targeted product 3a (entries 2–4).^57^ In contrast, evaluation of binary metal salt combinations revealed that the Cu(OAc)_2_·H_2_O/AgSbF_6_ system selectively promoted C5–H alkylation (entries 5–7). Replacing AgSbF_6_ with AgNTf_2_ or AgBF_4_, or substituting Cu(OAc)_2_·H_2_O with an alternative Lewis acidic salt hydrate, such as ScCl_3_·6H_2_O, did not improve the catalytic activity in this reaction system (entries 8–10).^58^ Solvent screening demonstrated that CH_2_Cl_2_ was the appropriate reaction medium (entries 11 and 12).^58^ Reducing the solvent volume by approximately half and increasing the concentration to 0.02 M led to a slight enhancement in yield of 3a (77% yield, entry 13). Notably, the yield and regioselectivity were maintained even when the catalyst loading was reduced, affording 3a in 77% yield (entry 14). Rhodium(iii)-mediated generation of Rh-carbene species facilitated C4–H alkylation (4a, 37% yield, entry 15). Ultraviolet irradiation-induced generation of a metal-free carbene did not afford 3a (entry 16), suggesting that Cu and Ag salts promote C5 selectivity *via* an alternative catalytic pathway. Further modifications of supporting ligands and copper valency did not improve the yield of 3a (entries 17–19); thus, the conditions described in entry 14 were determined to be optimal.

**Table 1:** Optimization of reaction conditions^a^

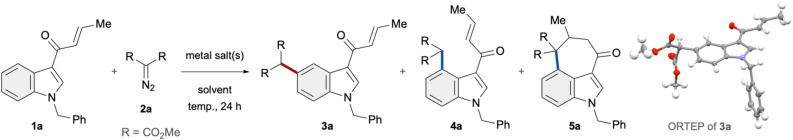						
Entry	Metal salt(s) (mol%)	Temp. (°C)	Solvent (M)	Yield of 3a (%)	Yield of 4a (%)	Yield of 5a (%)
1	Rh_2_(cap)_4_ (2.5), Cu(OAc)_2_·H_2_O (100), AgSbF_6_ (10)	40	ClCH_2_CH_2_Cl (0.0125)	18	Trace	Trace
2	Rh_2_(cap)_4_ (2.5)	40	ClCH_2_CH_2_Cl (0.0125)	0	0	0
3	AgSbF_6_ (10)	40	ClCH_2_CH_2_Cl (0.0125)	0	0	0
4	Cu(OAc)_2_·H_2_O (100)	40	ClCH_2_CH_2_Cl (0.0125)	0	0	0
5	Rh_2_(cap)_4_ (2.5), AgSbF_6_ (10)	40	ClCH_2_CH_2_Cl (0.0125)	0	0	0
6	Rh_2_(cap)_4_ (2.5), Cu(OAc)_2_·H_2_O (100)	40	ClCH_2_CH_2_Cl (0.0125)	0	0	0
7	Cu(OAc)_2_·H_2_O (100), AgSbF_6_ (10)	40	ClCH_2_CH_2_Cl (0.0125)	62	Trace	3
8	Cu(OAc)_2_·H_2_O (100), AgNTf_2_ (10)	40	ClCH_2_CH_2_Cl (0.0125)	0	0	0
9	Cu(OAc)_2_·H_2_O (100), AgBF_4_ (10)	40	ClCH_2_CH_2_Cl (0.0125)	20	7	Trace
10	ScCl_3_·6H_2_O (100), AgSbF_6_ (10)	40	ClCH_2_CH_2_Cl (0.0125)	0	0	0
11	Cu(OAc)_2_·H_2_O (100), AgSbF_6_ (10)	40	PhCl (0.0125)	42	Trace	3
12	Cu(OAc)_2_·H_2_O (100), AgSbF_6_ (10)	Reflux	CH_2_Cl_2_ (0.0125)	71	Trace	4
13	Cu(OAc)_2_·H_2_O (100), AgSbF_6_ (10)	Reflux	CH_2_Cl_2_ (0.02)	77	3	7
14	Cu(OAc)_2_·H_2_O (10), AgSbF_6_ (10)	Reflux	CH_2_Cl_2_ (0.02)	77	5	5
15	[Cp*RhCl_2_]_2_ (5), Cu(OAc)_2_·H_2_O (10), AgSbF_6_ (10)	Reflux	CH_2_Cl_2_ (0.02)	3	37	Trace
16	365 nm UV light instead of metal salt	40	CH_2_Cl_2_ (0.02)	0	0	0
17	CuOAc (10), AgSbF_6_ (10)	Reflux	CH_2_Cl_2_ (0.02)	28	Trace	3
18	Cu(acac)_2_ (10), AgSbF_6_ (10)	Reflux	CH_2_Cl_2_ (0.02)	21	4	2
19	Cu(NTf_2_)_2_ (10), AgSbF_6_ (10)	Reflux	CH_2_Cl_2_ (0.02)	42	Trace	4

a All reactions were conducted with metal salt(s) (0.0025–0.1 mmol), 1a (0.1 mmol) and 2a (0.2 mmol) under the indicated conditions for 24 h without using a syringe pump for slow addition of the diazo compounds. The yield was estimated by ^1^H NMR analysis using diphenylmethanol as the internal standard. cap, caprolactamate.

With the established conditions exhibiting high regioselectivity in hand, we next explored the generality and limitations of the current C–H functionalization methodology (Table 2). Utilization of dibenzyl malonate slightly enhanced the chemical yield, affording 3b in 79% yield. Replacing the enone functionality with a benzoyl group at the indole 3-position further increased the yield to 91% (3c). Products featuring aryl ketone functionalities with electron-donating and electron-withdrawing groups at the *ortho*-, *meta*-, and *para*-positions were obtained in good to excellent yields (3d–3h, 70–90%). With respect to substituents on the indole nitrogen, methyl, triisopropylsilyl, and *para*-methoxybenzyl groups, as well as a potentially reactive allyl group in carbene reactions, were well tolerated in the C5-selective functionalization processes (3i–3l, 72–79%). Substrates bearing a phenyl group at the C2 position and a methyl group at the C7 position were amenable to the catalysis (3m and 3p, 70% and 56% yield, respectively). Of note, reactions employing indole variants with substituents at the C6 position proximal to the reaction site proceeded, furnishing the corresponding products 3n and 3o in moderate to good yields. Substitution of the methyl group with a phenyl or secondary alkyl group at the β-position of the unsaturated ketone preserved the reactivity (3q and 3r, 74% yield for both), whereas 3-acetylindole derivatives produced diminished yields (3s, 55% yield). Additionally, non-symmetric malonate diesters, including acid-labile *tert*-butyl ester, were applicable, furnishing products 3u–3w in 68–75% yields. The Cu/Ag catalysis exhibited suboptimal performance in the C–H functionalization of the hydrocarbazolone variant (3x).

**Table 2:** Scope and generality of the regioselective C5–H alkylation reactions of indoles^a^

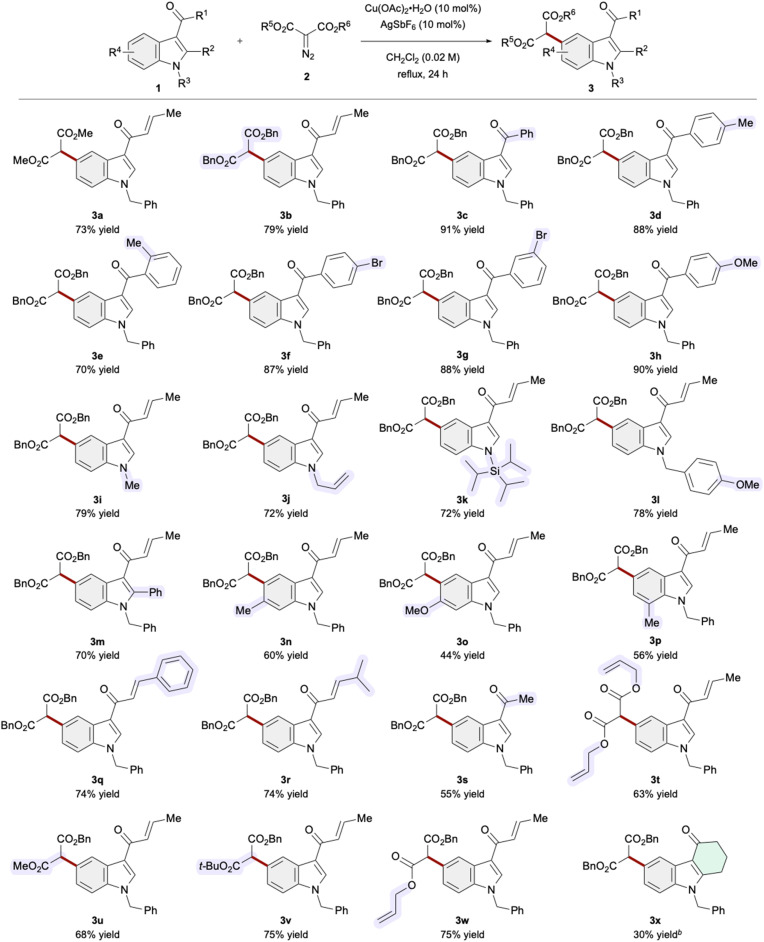

a All reactions were conducted with Cu(OAc)_2_·H_2_O (0.02 mmol), AgSbF_6_ (0.02 mmol), 1a (0.2 mmol), and 2a (0.4 mmol) under the indicated conditions without using a syringe pump. Isolated yields are reported.

b 1 mmol of 2 was used.

**Scheme 1:**
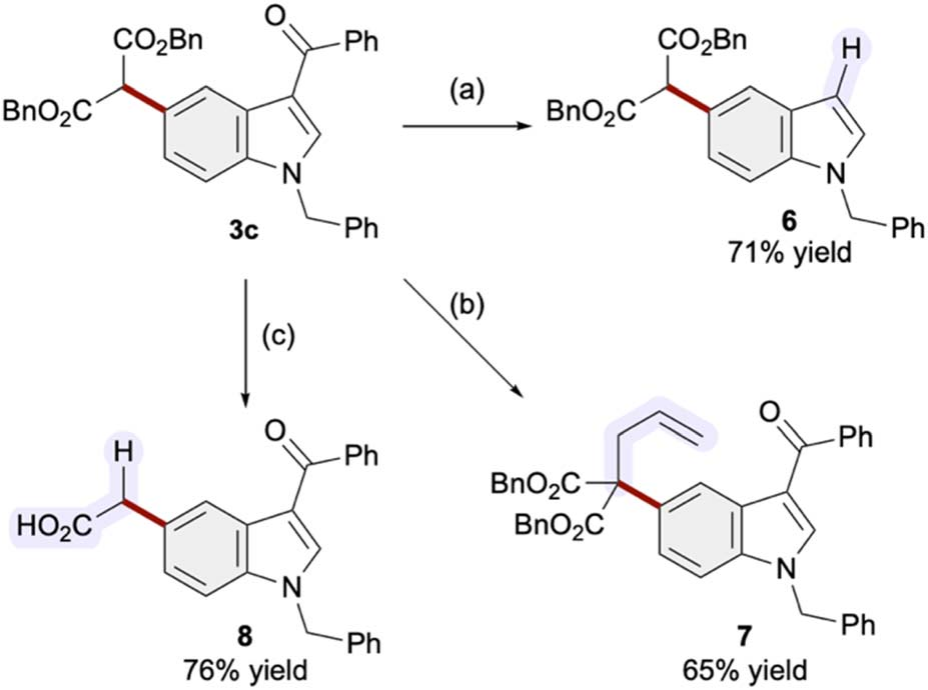
Derivatization of the product. Conditions: (a) TsOH (1 eq.), ethylene glycol (1.0 M), C_6_H_6_ (0.05 M), reflux, 6 h; (b) allyl bromide (1.5 eq.), NaH (1.2 eq.), DMF (0.2 M), rt, 5 h; (c) H_2_ (1 atm), Pd/C (10%), AcOEt (0.05 M), rt, 2 h; toluene/AcOEt (0.01 M), reflux, 1 h.

Scheme 1 illustrates the preliminary outcomes of product derivatization. The carbonyl group at the 3-position of indole was removed by heating under acidic conditions (3c → 6). An allylation of the malonate unit proceeded smoothly, affording 7 with the quaternary carbon in 65% yield (Scheme 1b). A Pd-catalyzed decarboxylation reaction facilitated the formation of a primary alkyl group, characteristic of those found in bioactive 5-substituted indoles (Scheme 1c and Fig. 1A).

Subsequently, we directed our attention to elucidating the reaction mechanism of the developed catalytic C–H functionalization. Copper and silver salts can react with acceptor-substituted diazo compounds, exhibiting similar reactivity in certain cases.^59^ However, replacing AgSbF_6_ with LiSbF_6_ afforded comparable yields, indicating that Ag-carbene species are not involved in the C5 functionalization process and highlighting the importance of the SbF_6_ counteranion (Table 3, entry 1). The results obtained using Zn(OAc)_2_·2H_2_O or Ni(OAc)_2_·4H_2_O demonstrated the essential role of the Cu core in this transformation (entries 2 and 3). Although reactions employing CuCl_2_/AgSbF_6_ or Cu(SbF_6_)_2_ proceeded, the efficiency was reduced compared to the optimal conditions, suggesting the involvement of the OAc counteranion (entries 4 and 5). Furthermore, mass balance considerations using a stoichiometric amount of Cu(OAc)_2_·H_2_O and a catalytic amount of AgSbF_6_ (Table 1, entry 13) strongly suggest the preferential formation of Cu(OAc)(SbF_6_) over Cu(SbF_6_)_2_.^60–62^ Next, the effect of Ag salt loading was investigated by varying the amount from 3 to 300 mol%. When 3 or 30 mol% of AgSbF_6_ was employed, the yield of 3a decreased to 62% or 35%, respectively (entries 6 and 8), compared to the optimized conditions (entry 7). Upon further increasing the AgSbF_6_ loading beyond 100 mol%, substantial amounts of black insoluble material appeared in the reaction flask, and both 1a and 2a remained unreacted (entries 9 and 10). These results indicate that a stoichiometric amount of Ag salt relative to Cu(OAc)_2_ is appropriate to achieve optimal catalytic performance for this C–H functionalization.

**Table 3:** Further studies of catalysis to elucidate mechanistic aspects

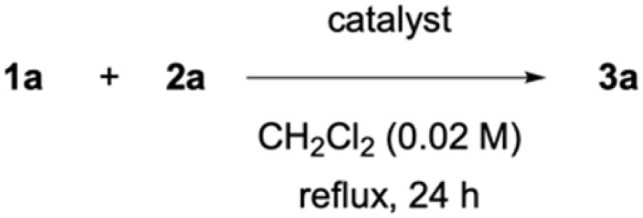		
Entry	Catalyst (mol%)	Yield (%)
1	Cu(OAc)_2_·H_2_O (10), LiSbF_6_ (10)	68
2	Zn(OAc)_2_·2H_2_O (10), AgSbF_6_ (10)	0
3	Ni(OAc)_2_·4H_2_O (10), AgSbF_6_ (10)	0
4	CuCl_2_ (5), AgSbF_6_ (10)	37
5	Cu(SbF_6_)_2_ (5)	40
6	Cu(OAc)_2_·H_2_O (10), AgSbF_6_ (3)	62
7^a^	Cu(OAc)_2_·H_2_O (10), AgSbF_6_ (10)	77
8	Cu(OAc)_2_·H_2_O (10), AgSbF_6_ (30)	35
9	Cu(OAc)_2_·H_2_O (10), AgSbF_6_ (100)	Trace
10	Cu(OAc)_2_·H_2_O (10), AgSbF_6_ (300)	Trace

a From entry 14 in Table 1 for comparison.

**Scheme 2:**
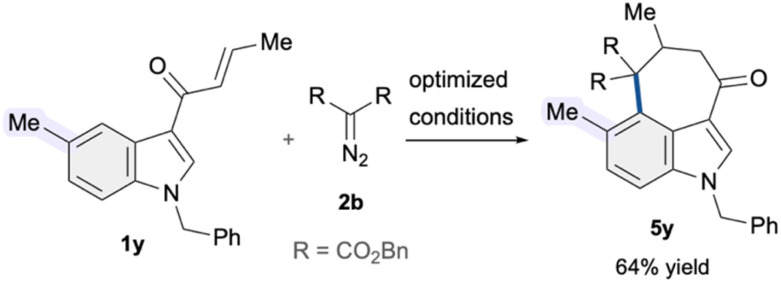
Reaction employing C5-substituted indole as a substrate.

We then investigated the reactivity pattern of indoles pre-substituted at the C5 position. Under the established conditions, substrate 1y featuring a simple methyl substituent at C5 underwent C4–H alkylation followed by intramolecular cyclization, giving 3,4-fused indole 5y in 64% yield. Accordingly, the developed copper-catalytic system also demonstrated potential to facilitate C4–H alkylation (Scheme 2).

**Fig. 2:**
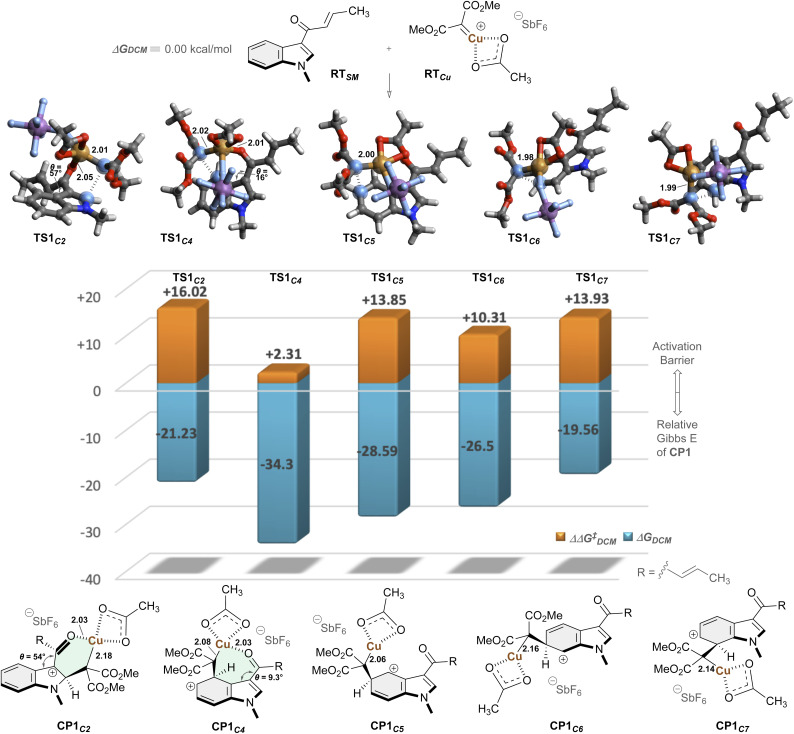
Computational analysis for elucidating the origin of regioselectivity. Calculation was carried out based on UCAM-B3LYP/6-311+G**/SDD//UB3LYP/Def2-SVPP level of theory in dichloromethane. Gibbs free energies are reported in kcal mol^−1^, and bond lengths in ångströms (Å).

To elucidate the origin of the regioselectivity and reaction processes, we next performed quantum chemical computation (Fig. 2).^63,64^ Calculations on the basis of dispersion-corrected density functional theory (DFT-D3)^65^ were performed in a spin-unrestricted formalism to account for the Cu(ii) species.^66,67^ First, we investigated elementary reactions between structurally simplified *N*-methylindole RT_SM_ and Cu-carbene species RT_Cu_, generated from the diazo compound. Computation at the UCAM-B3LYP/6-311+G**/SDD//UB3LYP/6-31G*/Def2-SVPP level of theory in dichloromethane revealed that electrophilic addition of the carbene species to the C5 position of the indole proceeds with a moderate activation barrier of 13.85 kcal mol^−1^, affording CP1_C5_*via*TS1_C5_. To elucidate the regioselectivity, we examined the reactivity at other positions of the indole ring. Notably, while the C2, C6, and C7 positions exhibited comparable activation energies (TS1_C2_: +16.02, TS1_C6_: +10.31, TS1_C7_: +13.93 kcal mol^−1^, respectively), the C4 position demonstrated remarkably enhanced reactivity with a dramatically lower activation barrier of only +2.31 kcal mol^−1^ (TS1_C4_). The observed pronounced enhancement in the reaction kinetics and marked stabilization were attributed to the coordination of the enone carbonyl functionality to the copper center (C=O⋯Cu) in both the transition state (2.01 Å, TS1_C4_) and the corresponding intermediate state (2.03 Å, CP1_C4_). Conversely, while carbonyl coordination to Cu was also possible in TS1_C2_, significant geometric distortion was observed, with the enone and indole planes twisted by 57°, suggesting disruption of π-conjugation.

**Fig. 3:**
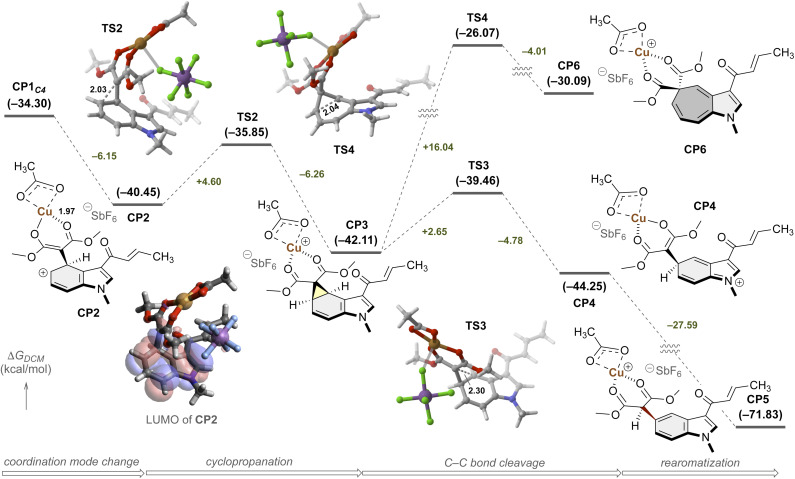
Gibbs free energy profile for reaction coordinates. The computation was implemented at the UCAM-B3LYP/6-311+G**/SDD//UB3LYP/Def2-SVPP level of theory in dichloromethane. Bond lengths are reported in Å.

Therefore, further computational analyses were performed starting from CP1_C4_ (Fig. 3). Following a coordination mode change to the more stable dicarbonyl chelate complex (−6.15 kcal mol^−1^, CP2), facile three-membered ring formation proceeded, driven by charge neutralization (+4.60 kcal mol^−1^, TS2).^68,69^ Indeed, visualization of the frontier orbitals of CP2 revealed that the lowest unoccupied molecular orbital (LUMO) is delocalized over the indole unit. The norcaradiene CP3 possessed a structural motif capable of undergoing ring expansion *via* electrocyclic reaction. Its divinylcyclopropane rearrangement proved both kinetically and thermodynamically unfavorable, however, presumably due to double dearomatization of the indole core (TS4: ΔΔ*G*^‡^ = +16.04, CP6: Δ*G* = −30.09 kcal mol^−1^). Instead, the ionic cleavage of the C–C bond proceeded more rapidly *via*TS3 (uphill by only 2.65 kcal mol^−1^), leading to CP4, driven by the electron push effect of the indole nitrogen and the electron pull effect of the Cu-activated 1,3-dicarbonyl structure on the fused cyclopropane.^70,71^ The subsequent proton transfer event afforded CP5, accompanied by significant energetic stabilization (Δ*G* = −27.59 kcal mol^−1^) due to rearomatization of the benzenoid core.^72^ These computational findings corroborate the experimentally observed regioselectivity and underscore the pivotal role of copper in this catalysis.

## Conclusions

In summary, we developed a direct, regioselective C5–H functionalization reaction of indoles under copper catalysis. The synthesized products exhibit a structural motif characteristic of indole alkaloids and active pharmaceutical ingredients, underscoring the synthetic utility of this methodology. Experimental mechanistic studies confirmed the crucial role of the combined Cu(OAc)_2_·H_2_O/AgSbF_6_ catalytic system. Computational mechanistic investigations revealed that the initial C4–H alkylation is directed by the carbonyl group at the 3-position of the indoles. Subsequent rapid formation and fragmentation of cyclopropane occur with an activation energy of less than 5 kcal mol^−1^. Hypothetical competing Buchner reactions were kinetically and thermodynamically unfavorable due to the loss of aromatic stabilization energy. We anticipate that the developed methodology and mechanistic insights will inspire future discoveries for direct and remote C–H functionalization of the benzenoid core in indole scaffolds. Further investigations utilizing metal-carbene species are currently ongoing in our laboratory.

## Author contributions

T. I. and M. Y. performed experiments. S. H. and T. N. supported and supervised the research. T. I. and S. H. performed computational studies. S. H. prepared the original draft of the manuscript with feedback from all authors. All authors discussed the experimental results.

## Conflicts of interest

There are no conflicts to declare.

## Supplementary Material

SC-OLF-D5SC03417E-s001

SC-OLF-D5SC03417E-s002

## Data Availability

General information, detailed experimental procedures, characterization data for compounds, and computational details are available in the ESI.†
