# Supplementary material for: Copper-catalyzed direct regioselective C5–H alkylation reactions of functionalized indoles with α-diazomalonates
Source: Chem Sci. 2025 Jul 15;16(33):14967–75. doi: 10.1039/d5sc03417e (PMC12278505; doi:10.1039/d5sc03417e)
Supplement: SC-016-D5SC03417E-s001 [file SC-016-D5SC03417E-s001.pdf]

This journal is © The Royal Society of Chemistry 2025

Copper-catalyzed direct regioselective C5–H alkylation reactions of functionalized indoles with α-diazomalonates

Tomohiro Isono,[a] Shingo Harada,*[a] Mai Yanagawa[a] and Tetsuhiro Nemoto*[a]

[a] Graduate School of Pharmaceutical Sciences, Chiba University, 1-8-1, Inohana, Chuo-ku, Chiba 260-8675, Japan

E-mail: Sharada@chiba-u.jp, tnemoto@faculty.chiba-u.jp

Table of Contents

**1.**	**General Information**	**S2**
**2.**	**Additional Studies**	**S3**
**3.**	**General Procedure for the Regioselective C5–H Alkylation of Indoles**	**S5**
**4.**	**Derivatization of the Product**	**S25**
**5.**	**Reaction employing C5-Substituted Indole**	**S29**
**6.**	**Preparation of Substrates**	**S31**
**7.**	**Single Crystal X-Ray Diffraction Analysis**	**S37**
**8.**	**Computational Details**	**S39**
**9.**	**Copy of ^{1}H-NMR and ^{13}C-NMR Spectra**	**S85**
**10.**	**References**	**S151**

1. General Information

NMR spectra were recorded on a JEOL ECZ 400 spectrometer, a JEOL ECP 400 spectrometer and a JEOL ECZ 600 spectrometer. Chemical shifts in $CDCl_3$ were reported downfield from TMS (= 0 ppm) for ^{1}H NMR. Data are reported in the following format: chemical shift, multiplicity (s = singlet, d = doublet, t = triplet, q = quartet, sep = septet, m = multiplet, and br = broad), integration and coupling constants in Hz. For ^{13}C NMR, chemical shifts were reported in the scale relative to the solvent signal [$CHCl_3$ (77.0 ppm)] as an internal reference. ESI mass spectra were measured on a JEOL AccuTOF LC-plus JMS-T100LP. Melting points were measured with a SIBATA NEL-270 melting point apparatus. Analytical thin layer chromatography was performed on Kieselgel $60F_{254}$, 0.25 mm thickness plates. Column chromatography was performed with silica gel 60 N (spherical, neutral 63-210 mesh). Reactions were conducted in dry solvent. Other reagents were purified by the usual methods.

2. Additional studies

Table s1 Screening of solvents for regioselective C5–H alkylation reaction

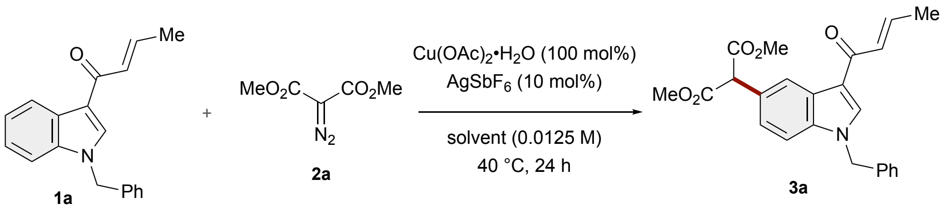

entry	solvent	yield (%)
1	DCE	62
2	Toluene	0
3	THF	0
4	DMF	0
5	MeCN	8
6	AcOEt	10
7	EtOH	23
8	DCM	71
9	$CHCl_3$	23
10	PhCl	42

Scheme s1 A control experiment

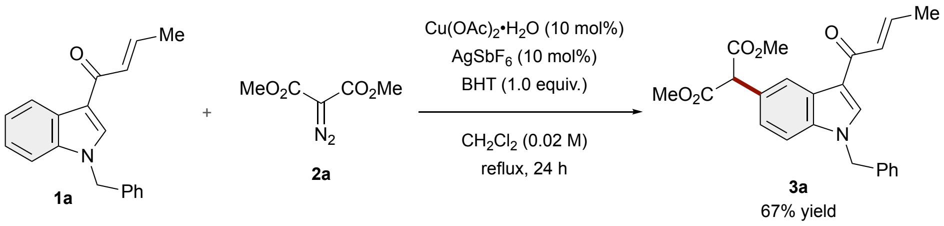

Scheme s2 Reactions using indoles bearing ester or cyano groups

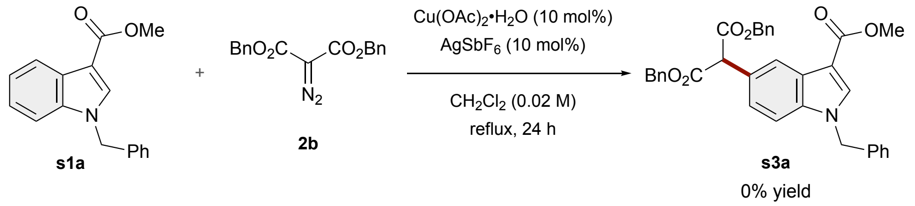

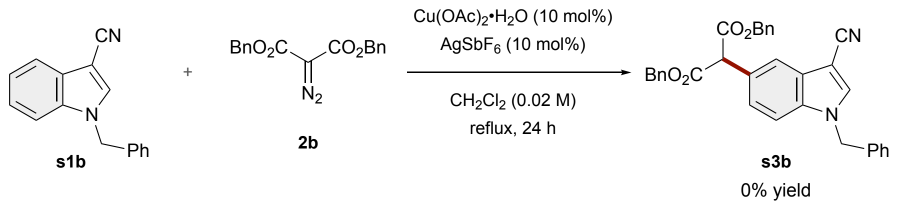

Scheme s3 Reactions using donor/acceptor-substituted and donor/donor-substituted diazo compounds

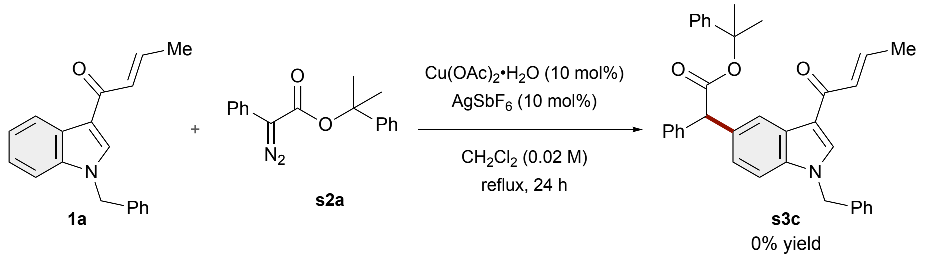

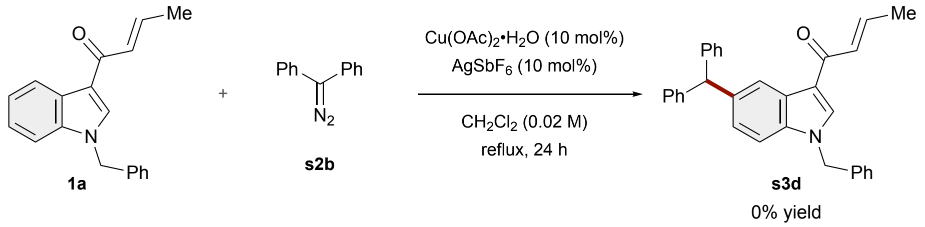

3. General Procedure for the Regioselective C5–H Alkylation of Indoles

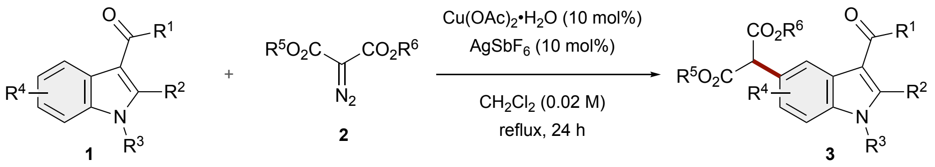

$Cu(OAc)_2 \cdot H_2O$ (4.0 mg, 10 mol%, 0.02 mmol), $AgSbF_6$ (6.9 mg, 10 mol%, 0.02 mmol), and indole substrate **1** (0.2 mmol, 1.0 equiv.) were introduced into a pre-dried 50-mL pear-shaped flask equipped with a magnetic stir bar. After injecting dichloromethane (5 mL) into the flask under an argon atmosphere, the solution was stirred at room temperature for 20 min. A solution of diazo compound **2** (0.4 mmol, 2.0 equiv.) in dichloromethane (5 mL) was then introduced into the reaction flask. The resulting solution was continuously stirred under reflux for 24 hours. After removing the solvent under reduced pressure, the obtained crude residue was purified by flash chromatography on silica gel (*n*-hexane/EtOAc) to afford the resultant product **3**. The analytical data for compound **3** are provided below.

Dimethyl (*E*)-2-(1-benzyl-3-(but-2-enoyl)-1*H*-indol-5-yl)malonate (3a)

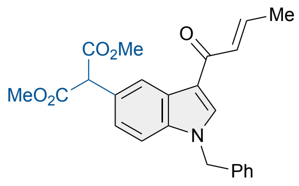

A pale orange amorphous solid (59.1 mg, 73% yield): TLC R_f = 0.25 (*n*-hexane/EtOAc, 2/1); ^{1}H NMR (400 MHz, $CDCl_3$) δ 8.45 (d, *J* = 1.2 Hz, 1H), 7.79 (s, 1H), 7.42 (dd, *J* = 8.8 Hz, 2.0 Hz, 1H), 7.36-7.30 (m, 4H), 7.17 (dd, *J* = 6.8 Hz, 1.6 Hz, 2H), 7.05 (qd, *J* = 15.6 Hz, 6.8 Hz, 1H), 6.73 (dd, *J* = 15.6 Hz, 1.6 Hz, 1H), 5.35 (s, 2H), 4.83 (s, 1H), 3.75 (s, 6H), 1.96 (dd, *J* = 6.8 Hz, 1.6 Hz, 3H); ^{13}C NMR (100 MHz, $CDCl_3$) δ 184.8, 169.2 (2C), 141.0, 137.0, 135.8, 135.2, 129.1 (2C), 128.7, 128.3, 127.20, 127.13 (2C), 127.09, 124.4 (2C), 117.6, 110.6, 57.8, 52.9 (2C), 50.9, 18.4; IR (ATR) ν 2953, 1732, 1658, 1603, 1523, 1258, 1217, 1177, 1146, 1061, 1027, 907 cm^{-1}; HRMS (ESI-TOF) $[M + Na]^+$ calcd for $C_{24}H_{23}NNaO_5^+$ m/z 428.1468, found 424.1468.

Dibenzyl (*E*)-2-(1-benzyl-3-(but-2-enoyl)-1*H*-indol-5-yl)malonate (3b)

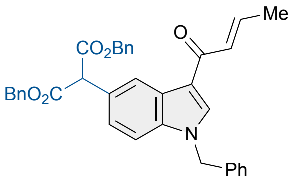

A pale orange amorphous solid (88.0 mg, 79% yield): TLC R_f = 0.25 (*n*-hexane/EtOAc, 5/2); ^{1}H NMR (400 MHz, $CDCl_3$) δ 8.48 (d, *J* = 1.6 Hz, 1H), 7.76 (s, 1H), 7.40 (dd, *J* = 8.8 Hz, 2.0 Hz, 1H), 7.35-7.22 (m, 14H), 7.14 (dd, *J* = 7.6 Hz, 2.0 Hz, 2H), 7.04 (qd, *J* = 15.2 Hz, 6.8 Hz, 1H), 6.72 (dd, *J* = 15.2 Hz, 1.6 Hz, 1H), 5.30 (s, 2H), 5.19 (d, *J* = 12.8 Hz, 2H), 5.12 (d, *J* = 12.8 Hz, 2H), 4.91 (s, 1H), 1.95 (dd, *J* = 6.8 Hz, 1.6 Hz, 3H); ^{13}C

NMR (400 MHz, $CDCl_3$) δ 184.8, 168.5 (2C), 141.0, 137.0, 135.8, 135.4 (2C), 135.1, 129.2 (2C), 128.7, 128.63, 128.58 (4C), 128.36, 128.30 (2C), 128.2 (4C), 127.11 (2C), 127.08, 124.63, 124.58, 117.6, 110.6, 67.5 (2C), 58.1, 50.9, 18.4; IR (ATR) ν 3064, 3032, 2943, 1730, 1658, 1604, 1523, 1455, 1212, 1176, 1139, 908 cm^{-1}; HRMS (ESI-TOF) $[M + Na]^+$ calcd for $C_{36}H_{31}NNaO_5^+$ m/z 580.2094, found 580.2102.

Dibenzyl 2-(3-benzoyl-1-benzyl-1*H*-indol-5-yl)malonate (3c)

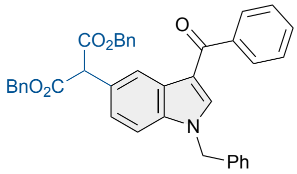

A pale orange amorphous solid (107.7 mg, 91% yield): TLC $R_f = 0.3$ (*n*-hexane/EtOAc, 5/2); ^1H NMR (400 MHz, $CDCl_3$) δ 8.46 (d, $J = 2.0$ Hz, 1H), 7.78 (dd, $J = 8.8$ Hz, 1.2 Hz, 2H), 7.58 (s, 1H), 7.51 (tt, $J = 7.2$ Hz, 1.6 Hz, 1H), 7.45 (dd, $J = 8.0$ Hz, 1.2 Hz, 2H), 7.41 (dd, $J = 8.4$ Hz, 1.6 Hz, 1H), 7.32-7.21 (m, 14H), 7.10 (dd, $J = 7.6$ Hz, 2.0 Hz, 2H), 5.28 (s, 2H), 5.18 (d, $J = 12.8$ Hz, 2H), 5.12 (d, $J = 12.8$ Hz, 2H), 4.92 (s, 1H); ^{13}C NMR (400 MHz, $CDCl_3$) δ 190.9, 168.5 (2C), 140.7, 137.8, 137.0, 135.8, 135.4 (2C), 131.4, 129.2 (2C), 128.8 (2C), 128.6 (4C), 128.5 (2C), 128.36 (2C), 128.33, 128.2 (4C), 127.5, 127.3, 126.9 (2C), 124.8, 124.4, 116.2, 110.8, 67.5 (2C), 58.1, 51.0; IR (ATR) ν 3062, 3032, 2948, 1731, 1616, 1575, 1521, 1379, 1246, 1212, 1172, 1137 cm^{-1}; HRMS (ESI-TOF)

[M + Na]$^+$ calcd for $C_{39}H_{31}NNaO_5^+$ m/z 616.2094, found 616.2092.

Dibenzyl 2-(1-benzyl-3-(4-methylbenzoyl)-1*H*-indol-5-yl)malonate (3d)

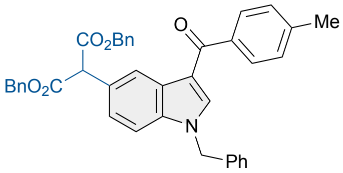

A pale yellow amorphous solid (106.9 mg, 88% yield): TLC R_f = 0.5 (*n*-hexane/EtOAc, 2/1); ^{1}H NMR (400 MHz, $CDCl_3$) δ 8.44 (d, *J* = 1.2 Hz, 1H), 7.71 (d, *J* = 8.0 Hz, 2H), 7.60 (s, 1H), 7.41 (dd, *J* = 8.4 Hz, 1.6 Hz, 1H), 7.33-7.22 (m, 16H), 7.11 (dd, *J* = 7.2 Hz, 2.0 Hz, 2H), 5.30 (s, 2H), 5.18 (d, *J* = 12.4 Hz, 2H), 5.13 (d, *J* = 12.4 Hz, 2H), 4.92 (s, 1H), 2.41 (s, 3H); ^{13}C NMR (400 MHz, $CDCl_3$) δ 190.6, 168.5 (2C), 141.9, 138.0, 137.5, 136.9, 135.9, 135.5 (2C), 129.14 (2C), 129.12 (2C), 129.0 (2C), 128.8, 128.6 (4C), 128.3 (2C), 128.2 (4C), 127.6, 127.2, 126.9 (2C), 124.7, 124.4, 116.3, 110.7, 67.5 (2C), 58.1, 51.0, 21.7; IR (ATR) ν 3063, 3032, 2946, 2362, 1731, 1619, 1604, 1521, 1378, 1173, 1138, 907 cm^{-1}; HRMS (ESI-TOF) [M + Na]$^+$ calcd for $C_{40}H_{33}NNaO_5^+$ m/z 630.2251, found 630.2270.

Dibenzyl 2-(1-benzyl-3-(2-methylbenzoyl)-1*H*-indol-5-yl)malonate (3e)

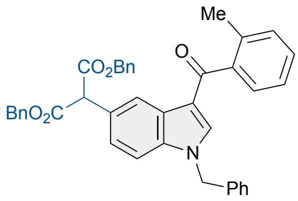

A pale yellow amorphous solid (84.8 mg, 70% yield): TLC R_f = 0.25 (*n*-hexane/EtOAc, 4/1); ^{1}H NMR (400 MHz, $CDCl_3$) δ 8.42 (d, *J* = 1.2 Hz, 1H), 7.42 (dd, *J* = 8.4 Hz, 1.6 Hz, 1H), 7.40-7.21 (m, 19H), 7.09 (dd, *J* = 7.6 Hz, 2.0 Hz, 2H), 5.26 (s, 2H), 5.19 (d, *J* = 12.4 Hz, 2H), 5.13 (d, *J* = 12.4 Hz, 2H), 4.92 (s, 1H), 2.39 (s, 3H); ^{13}C NMR (400 MHz, $CDCl_3$) δ 192.9, 168.4 (2C), 140.8, 138.5, 137.1, 136.1, 135.7, 135.4 (2C), 131.0, 129.6, 129.1 (2C), 128.8, 128.6 (4C), 128.5, 128.3 (2C), 128.2 (4C), 127.8, 127.1, 126.9 (2C), 125.3, 124.8, 124.4, 117.7, 110.8, 67.5 (2C), 58.1, 51.1, 19.8; IR (ATR) ν 3062, 3033, 2954, 1731, 1619, 1522, 1455, 1378, 1244, 1212, 1174, 1139 cm^{-1}; HRMS (ESI-TOF) $[M + Na]^+$ calcd for $C_{40}H_{33}NNaO_5^+$ m/z 630.2251, found 630.2260.

Dibenzyl 2-(1-benzyl-3-(4-bromobenzoyl)-1*H*-indol-5-yl)malonate (3f)

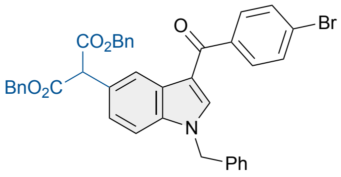

A pale yellow amorphous solid (116.8 mg, 87% yield): TLC R_f = 0.25 (*n*-hexane/EtOAc, 4/1); ^{1}H NMR (400 MHz, $CDCl_3$) δ 8.41 (d, *J* = 1.2 Hz, 1H), 7.68-7.55 (m, 5H), 7.43 (dd, *J* = 8.4 Hz, 1.6 Hz, 1H), 7.35-7.23 (m, 14H), 7.12 (dd, *J* = 7.2 Hz, 1.6 Hz, 2H), 5.32 (s,

2H), 5.19 (d, J = 12.0 Hz, 2H), 5.13 (d, J = 12.0 Hz, 2H), 4.92 (s, 1H); ^{13}C NMR (400 MHz, $CDCl_3$) δ 189.5, 168.4 (2C), 139.4, 137.6, 137.0, 135.7, 135.4 (2C), 131.7, 130.4 (2C), 129.2 (2C), 128.8 (2C), 128.6 (4C), 128.45, 128.41, 128.37 (2C), 128.2 (4C), 127.4 127.0 (2C), 125.0, 124.3, 115.9, 110.8, 67.5 (2C), 58.1, 51.0; IR (ATR) ν 3063, 3032, 2949, 1731, 1617, 1585, 1522, 1378, 1213, 1171, 1139, 1009 cm^{-1}; HRMS (ESI-TOF) $[M + Na]^+$ calcd for $C_{39}H_{30}BrNNaO_5^+$ m/z 694.1200, found 694.1200.

Dibenzyl 2-(1-benzyl-3-(3-bromobenzoyl)-1*H*-indol-5-yl)malonate (3g)

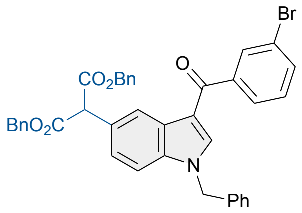

A pale yellow amorphous solid (117.8 mg, 88% yield): TLC R_f = 0.25 (*n*-hexane/EtOAc, 4/1); ^{1}H NMR (400 MHz, $CDCl_3$) δ 8.41 (d, J = 1.6 Hz, 1H), 7.92 (s, 1H), 7.70 (d, J = 7.6 Hz, 1H), 7.66 (ddd, J = 8.0 Hz, 1.2 Hz, 1.2 Hz, 1H), 7.58 (s, 1H), 7.44 (dd, J = 8.4 Hz, 1.6 Hz, 1H), 7.36-7.22 (m, 15 H), 7.14 (dd, J = 7.6 Hz, 1.6 Hz, 2H), 5.35 (s, 2H), 5.20 (d, J = 12.4 Hz, 2H), 5.14 (d, J = 12.4 Hz, 2H), 4.92 (s, 1H); ^{13}C NMR (100 MHz, $CDCl_3$) δ 188.9, 168.4 (2C), 142.6, 137.9, 137.0, 135.7, 135.4 (2C), 134.2, 131.7, 130.1, 129.2 (2C), 128.8, 128.6 (4C), 128.45, 128.39 (2C), 128.2 (4C), 127.5, 127.3, 127.0 (2C), 125.0, 124.3, 122.7, 115.8, 110.9, 67.5 (2C), 58.1, 51.0; IR (ATR) ν 3064, 3032, 2950,

1731, 1619, 1560, 1521, 1377, 1242, 1214, 1178, 1140 cm^{-1}; HRMS (ESI-TOF) [M + Na]$^+$ calcd for $C_{39}H_{30}BrNNaO_5^+$ m/z 694.1200, found 694.1195.

Dibenzyl 2-(1-benzyl-3-(4-methoxybenzoyl)-1*H*-indol-5-yl)malonate (3h)

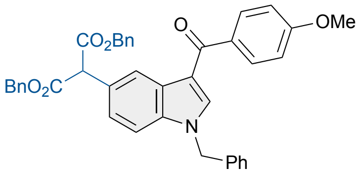

A pale orange amorphous solid (112.8 mg, 90% yield): TLC R_f = 0.2 (*n*-hexane/EtOAc, 5/2); ^{1}H NMR (400 MHz, $CDCl_3$) δ 8.40 (d, *J* = 2.0 Hz, 1H), 7.81 (d, *J* = 8.8 Hz, 2H), 7.60 (s, 1H), 7.41 (dd, *J* = 8.8 Hz, 2.0Hz, 1H), 7.32-7.20 (m, 14H), 7.11 (dd, *J* = 7.2 Hz, 2.0 Hz, 2H), 6.94 (d, *J* = 8.8 Hz, 2H), 5.29 (s, 2H), 5.18 (d, *J* = 12.4 Hz, 2H), 5.12 (d, *J* = 12.4 Hz, 2H), 4.91 (s, 1H), 3.84 (s, 3H); ^{13}C NMR (100 MHz, $CDCl_3$) δ 189.7, 168.5 (2C), 162.4, 137.0, 136.9, 135.9, 135.5 (2C), 133.3, 131.0 (2C), 129.1 (2C), 128.6 (4C), 128.33 (2C), 128.30, 128.2 (4C), 127.7, 127.02, 126.96 (2C), 124.6, 124.3, 116.3, 113.7 (2C), 110.7, 67.5 (2C), 58.1, 55.5, 50.9; IR (ATR) ν 3032, 2950, 2840, 1731, 1614, 1599, 1570, 1521, 1251, 1166, 1139, 1025 cm^{-1}; HRMS (ESI-TOF) [M + Na]$^+$ calcd for $C_{40}H_{33}NNaO_6^+$ m/z 646.2200, found 646.2200.

Dibenzyl (*E*)-2-(3-(but-2-enoyl)-1-methyl-1*H*-indol-5-yl)malonate (3i)

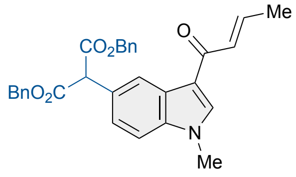

A brown amorphous solid (76.3 mg, 79% yield): TLC R_f = 0.4 (*n*-hexane/EtOAc, 1/1); ^{1}H NMR (400 MHz, $CDCl_3$) δ 8.43 (d, *J* = 1.6 Hz, 1H), 7.58 (s, 1H), 7.43 (dd, *J* = 8.8 Hz, 1.6 Hz, 1H), 7.33-7.20 (m, 11H), 7.01 (qd, *J* = 15.6 Hz, 6.8 Hz, 1H), 6.67 (dd, *J* = 15.6 Hz, 1.6 Hz, 1H), 5.20 (d, *J* = 12.4 Hz, 2H), 5.14 (d, *J* = 12.4 Hz, 2H), 4.92 (s, 1H), 3.67 (s, 3H), 1.95 (dd, *J* = 6.8 Hz, 1.6 Hz, 3H); ^{13}C NMR (400 MHz, $CDCl_3$) δ 184.7, 168.6 (2C), 140.6, 137.3, 136.0, 135.4 (2C), 128.8, 128.6 (4C), 128.3 (2C), 128.2 (4C), 126.9, 126.8, 124.4, 124.3, 116.9, 110.1, 67.5 (2C), 58.1, 33.6, 18.4; IR (ATR) ν 3033, 2942, 1730, 1658, 1603, 1527, 1456, 1284, 1213, 1140, 1081, 907 cm^{-1}; HRMS (ESI-TOF) [M + Na]$^+$ calcd for $C_{30}H_{27}NNaO_5^+$ m/z 504.1781, found 504.1779.

Dibenzyl (*E*)-2-(1-allyl-3-(but-2-enoyl)-1*H*-indol-5-yl)malonate (3j)

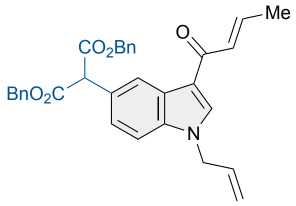

A yellow amorphous solid (73.2 mg, 72% yield): TLC R_f = 0.2 (*n*-hexane/EtOAc, 5/2); ^{1}H NMR (400 MHz, $CDCl_3$) δ 8.46 (d, *J* = 1.2 Hz, 1H), 7.73 (s, 1H), 7.44 (dd, *J* = 8.4 Hz, 1.6 Hz, 1H), 7.33-7.24 (m, 11H), 7.03 (qd, *J* = 15.2 Hz, 6.8 Hz, 1H), 6.73 (dd, *J* = 15.2

Hz, 1.6 Hz, 1H), 5.97 (tdd, J = 10.4 Hz, 7.2 Hz, 5.6 Hz, 1H), 5.27 (d, J = 10.4 Hz, 1H), 5.22-5.12 (m, 5H), 4.92 (s, 1H), 4.70 (d, J = 7.2 Hz, 2H), 1.96 (dd, J = 6.8 Hz, 1.6 Hz, 3H); ^{13}C NMR (400 MHz, $CDCl_3$) δ 184.8, 168.5 (2C), 140.8, 136.8, 135.5 (2C), 134.8, 132.1, 128.7, 128.6 (4C), 128.4, 128.3 (2C), 128.2 (4C), 127.0, 124.6, 124.4, 118.8, 117.4, 110.5, 67.5 (2C), 58.1, 49.5, 18.4; IR (ATR) ν 3033, 2947, 1731, 1658, 1604, 1523, 1386, 1285, 1191, 1141, 963, 909 cm^{-1}; HRMS (ESI-TOF) $[M + Na]^+$ calcd for $C_{32}H_{29}NNaO_5^+$ m/z 530.1938, found 530.1938.

Dibenzyl (*E*)-2-(3-(but-2-enoyl)-1-(triisopropylsilyl)-1*H*-indol-5-yl)malonate (3k)

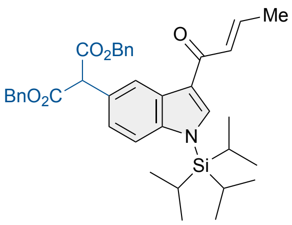

A pale yellow amorphous solid (89.5 mg, 72% yield): TLC R_f = 0.25 (*n*-hexane/EtOAc, 5/1); ^{1}H NMR (400 MHz, $CDCl_3$) δ 8.47 (d, J = 2.0 Hz, 1H), 7.90 (s, 1H), 7.48 (d, J = 7.2 Hz, 1H), 7.38 (dd, J = 8.8 Hz, 2.0 Hz, 1H), 7.32-7.24 (m, 10H), 7.05 (qd, J = 15.2 Hz, 7.2 Hz, 1H), 6.77 (dd, J = 15.2 Hz, 1.6 Hz, 1H), 5.20 (d, J = 12.4 Hz, 2H), 5.15 (d, J = 12.4 Hz, 2H), 4.92 (s, 1H), 1.98 (dd, J =7.2 Hz, 1.6 Hz, 3H), 1.73 (sep, J = 7.6 Hz, 3H), 1.17 (d, J = 7.6 Hz, 18H); ^{13}C NMR (400 MHz, $CDCl_3$) δ 185.3, 168.5 (2C), 141.4, 141.0, 138.9, 135.5 (2C), 128.82, 128.76, 128.6 (4C), 128.2 (2C), 128.1 (4C), 126.8, 124.3,

124.2, 120.5, 114.3, 67.4 (2C), 58.0, 18.4, 18.1 (6C), 12.8 (3C); IR (ATR) ν 3033, 2945, 2869, 1732, 1659, 1608, 1520, 1173, 1139, 1013, 908, 882 cm^{-1}; HRMS (ESI-TOF) [M + Na]$^+$ calcd for $C_{38}H_{45}NNaO_5Si^+$ m/z 646.2959, found 646.2963.

Dibenzyl (*E*)-2-(3-(but-2-enoyl)-1-(4-methoxybenzyl)-1*H*-indol-5-yl)malonate (3l)

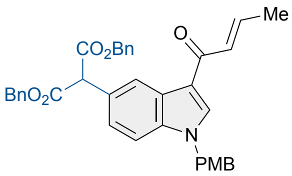

A pale orange amorphous solid (92.0 mg, 78% yield): TLC R_f = 0.25 (*n*-hexane/EtOAc, 2/1); ^{1}H NMR (400 MHz, $CDCl_3$) δ 8.47 (d, *J* = 1.2 Hz, 1H), 7.71 (s, 1H), 7.40 (dd, *J* = 8.8 Hz, 1.6 Hz, 1H), 7.30-7.23 (m, 11H), 7.10-6.97 (m, 3H), 6.84 (d, *J* = 8.4 Hz, 2H), 6.69 (dd, *J* = 15.2 Hz, 1.2 Hz, 1H), 5.20-5.10 (m, 6H), 4.91 (s, 1H), 3.76 (s, 3H), 1.93 (dd, *J* =6.8 Hz, 1.2 Hz, 3H); ^{13}C NMR (400 MHz, $CDCl_3$) δ 184.8, 168.5 (2C), 159.6, 140.8, 137.0, 135.5 (2C), 135.0, 128.7 (2C), 128.6 (4C), 128.5, 128.3 (2C), 128.2 (4C), 127.7, 127.1, 127.0, 124.6, 124.5, 117.5, 114.5 (2C), 110.6, 67.4 (2C), 58.1, 55.4, 50.4, 18.4; IR (ATR) ν 3033, 2936, 2837, 1731, 1658, 1606, 1513, 1247, 1211, 1173, 1138, 907 cm^{-1}; HRMS (ESI-TOF) [M + Na]$^+$ calcd for $C_{37}H_{33}NNaO_6^+$ m/z 610.2200, found 610.2201.

Dibenzyl (*E*)-2-(1-benzyl-3-(but-2-enoyl)-2-phenyl-1*H*-indol-5-yl)malonate (3m)

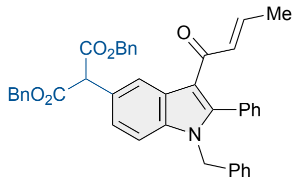

A pale yellow amorphous solid (88.9 mg, 70% yield): TLC R_f = 0.3 (*n*-hexane/EtOAc, 3/1); ^{1}H NMR (400 MHz, $CDCl_3$) δ 8.50 (d, *J* = 2.0 Hz, 1H), 7.48 (tt, *J* = 7.2 Hz, 1.2 Hz, 1H), 7.44-7.37 (m, 3H), 7.33-7.19 (m, 16H), 6.91 (dd, *J* = 6.8 Hz, 2.0 Hz, 2H), 6.80 (qd, *J* = 15.2 Hz, 6.8 Hz, 1H), 5.84 (dd, *J* = 15.2 Hz, 1.6 Hz, 1H), 5.21-5.11 (m, 6H), 4.93 (s, 1H), 1.55 (dd, *J* = 6.8 Hz, 1.6 Hz, 3H); ^{13}C NMR (400 MHz, $CDCl_3$) δ 187.2, 168.5 (2C), 146.5, 140.1, 136.8, 136.5, 135.5 (2C), 131.0, 130.9 (2C), 129.8, 128.9 (2C), 128.67 (2C), 128.64, 128.60 (4C), 128.5, 128.3 (2C), 128.2 (4C), 127.7, 127.1, 126.2 (2C), 124.6, 124.4, 116.7, 111.0, 67.4 (2C), 58.2, 47.9, 18.2; IR (ATR) ν 3063, 3032, 2949, 1731, 1653, 1600, 1529, 1471, 1404, 1215, 1137, 909 cm^{-1}; HRMS (ESI-TOF) $[M + Na]^+$ calcd for $C_{42}H_{35}NNaO_5^+$ m/z 656.2407, found 656.2408.

Dibenzyl (*E*)-2-(1-benzyl-3-(but-2-enoyl)-6-methyl-1*H*-indol-5-yl)malonate (3n)

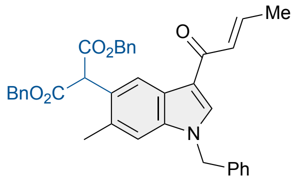

A pale orange amorphous solid (68.9 mg, 60% yield): TLC R_f = 0.25 (*n*-hexane/EtOAc, 5/2); ^{1}H NMR (600 MHz, $CDCl_3$) δ 8.50 (s, 1H), 7.70 (s, 1H), 7.36-7.27 (m, 13H), 7.14

(d, J = 7.8 Hz, 2H), 7.08 (s, 1H), 7.02 (qd, J = 15.0 Hz, 6.6 Hz, 1H), 6.70 (dd, J = 15.0 Hz, 1.8 Hz, 1H), 5.30 (s, 2H), 5.23 (d, J = 12.0 Hz, 2H), 5.20 (d, J = 12.0 Hz, 2H), 5.05 (s, 1H), 2.33 (s, 3H), 1.94 (dd, J = 6.6 Hz, 1.8 Hz, 3H); ^{13}C NMR (600 MHz, $CDCl_3$) δ 184.6, 168.4 (2C), 140.5, 137.2, 135.9, 135.4 (2C), 134.5, 132.5, 129.0 (2C), 128.9, 128.5 (4C), 128.4, 128.20 (4C), 128.17 (2C), 126.9 (2C), 126.6, 125.4, 124.0, 117.7, 111.6, 67.4 (2C), 55.6, 50.6, 20.7, 18.2; IR (ATR) ν 3033, 2941, 1732, 1658, 1604, 1525, 1455, 1382, 1215, 1180, 1144, 903 cm^{-1}; HRMS (ESI-TOF) [M + Na]$^+$ calcd for $C_{37}H_{33}NNaO_5^+$ m/z 594.2251, found 594.2255.

Dibenzyl (*E*)-2-(1-benzyl-3-(but-2-enoyl)-6-methoxy-1*H*-indol-5-yl)malonate (3o)

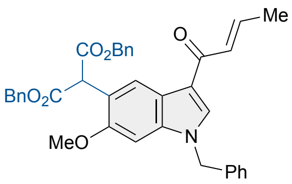

A pale yellow amorphous solid (51.5 mg, 44% yield): TLC R_f = 0.2 (*n*-hexane/EtOAc, 5/2); ^{1}H NMR (600 MHz, $CDCl_3$) δ 8.40 (s, 1H), 7.63 (s, 1H), 7.35-7.25 (m, 13H), 7.14 (d, J = 4.8 Hz, 2H), 7.01 (qd, J = 15.0 Hz, 7.2 Hz, 1H), 6.68 (dd, J = 15.0 Hz, 1.8 Hz, 1H), 6.62 (s, 1H), 5.26 (s, 2H), 5.21 (s, 4H), 5.15 (s, 1H), 3.60 (s, 3H), 1.94 (dd, J = 7.2 Hz, 1.8 Hz, 3H); ^{13}C NMR (600 MHz, $CDCl_3$) δ 184.6, 168.5 (2C), 154.9, 140.3, 137.8, 135.8, 135.7 (2C), 133.8, 129.0 (2C), 128.8, 128.4 (4C), 128.17 (4C), 128.15, 128.05

(2C), 127.0 (2C), 124.2, 120.4, 118.5, 117.7, 92.3, 67.1 (2C), 55.6, 53.3, 50.7, 18.2; IR (ATR) ν 3032, 2935, 1732, 1658, 1627, 1604, 1577, 1525, 1200, 1144, 1054, 901 cm^{-1}; HRMS (ESI-TOF) $[M + Na]^+$ calcd for $C_{37}H_{33}NNaO_6^+$ m/z 610.2200, found 610.2205.

Dibenzyl (*E*)-2-(1-benzyl-3-(but-2-enoyl)-7-methyl-1*H*-indol-5-yl)malonate (3p)

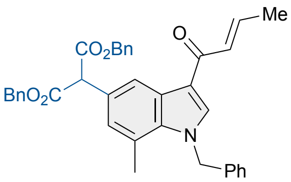

An orange amorphous solid (64.1 mg, 56% yield): TLC R_f = 0.25 (*n*-hexane/EtOAc, 5/2); ^{1}H NMR (400 MHz, $CDCl_3$) δ 8.41 (d, *J* = 1.6 Hz, 1H), 7.66 (s, 1H), 7.35-7.24 (m, 13H), 7.07 (d, *J* = 0.8 Hz, 1H), 7.02 (qd, *J* = 15.2 Hz, 6.8 Hz, 1H), 6.93 (dd, *J* = 8.0 Hz, 2.0 Hz, 2H), 6.69 (dd, *J* = 15.2 Hz, 1.6 Hz, 1H), 5.24 (s, 2H), 5.19 (d, *J* = 12.8 Hz, 2H), 5.13 (d, *J* = 12.8 Hz, 2H), 4.88 (s, 1H), 2.42 (s, 3H), 1.93 (dd, *J* = 6.8 Hz, 1.6 Hz, 3H); ^{13}C NMR (400 MHz, $CDCl_3$) δ 184.8, 168.5 (2C), 140.7, 137.9, 137.2, 135.9, 135.5 (2C), 129.2 (2C), 128.8, 128.6 (4C), 128.3 (2C), 128.2 (4C), 128.1, 128.0, 127.3, 127.1, 125.6 (2C), 122.5, 121.9, 117.3, 67.4 (2C), 57.9, 53.0, 19.5, 18.3; IR (ATR) ν 3064, 3032, 2957, 1730, 1658, 1603, 1533, 1453, 1217, 1177, 1138, 908 cm^{-1}; HRMS (ESI-TOF) $[M + Na]^+$ calcd for $C_{37}H_{33}NNaO_5^+$ m/z 594.2251, found 594.2252.

Dibenzyl 2-(1-benzyl-3-cinnamoyl-1*H*-indol-5-yl)malonate (3q)

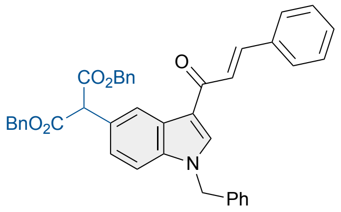

A pale orange amorphous solid (91.9 mg, 74% yield): TLC R_f = 0.3 (*n*-hexane/EtOAc, 5/2); ^{1}H NMR (400 MHz, $CDCl_3$) δ 8.54 (d, *J* = 2.0 Hz, 1H), 7.83 (s, 1H), 7.79 (d, *J* = 15.6 Hz, 1H), 7.61 (dd, *J* = 7.2 Hz, 2.0 Hz, 2H), 7.41-7.20 (m, 19H), 7.13 (dd, *J* = 7.2 Hz, 2.0 Hz, 2H), 5.28 (s, 2H), 5.19 (d, *J* = 12.4 Hz, 2H), 5.13 (d, *J* = 12.4 Hz, 2H), 4.94 (s, 1H); ^{13}C NMR (400 MHz, $CDCl_3$) δ 184.4, 168.5 (2C), 141.3, 137.1, 135.8, 135.44 (2C), 135.40, 135.37, 130.0, 129.2 (2C), 129.0 (2C), 128.6 (4C), 128.5, 128.35 (2C), 128.33 (2C), 128.2 (4C), 127.2, 127.11 (2C), 127.06, 124.7, 124.6, 123.9, 118.2, 110.8, 67.5 (2C), 58.1, 51.0; IR (ATR) ν 3063, 3032, 2948, 2360, 2252, 1730, 1648, 1593, 1523, 1175, 1139, 906 cm^{-1}; HRMS (ESI-TOF) $[M + Na]^+$ calcd for $C_{41}H_{33}NNaO_5^+$ m/z 642.2251, found 642.2261.

Dibenzyl (*E*)-2-(1-benzyl-3-(4-methylpent-2-enoyl)-1*H*-indol-5-yl)malonate (3r)

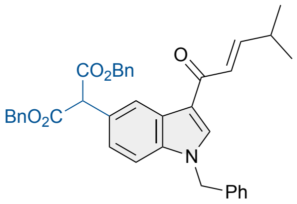

A pale yellow amorphous solid (86.4 mg, 74% yield): TLC R_f = 0.3 (*n*-hexane/EtOAc, 3/1); ^{1}H NMR (400 MHz, $CDCl_3$) δ 8.47 (d, *J* = 1.2 Hz, 1H), 7.77 (s, 1H), 7.40 (dd, *J* = 8.8 Hz, 1.6 Hz, 1H), 7.33-7.23 (m, 14H), 7.14 (dd, *J* = 7.6 Hz, 2.0 Hz, 2H), 7.00 (dd, *J* = 15.6 Hz, 7.2 Hz, 1H), 6.63 (dd, *J* = 15.6 Hz, 1.2 Hz, 1H), 5.30 (s, 2H), 5.18 (d, *J* = 12.4 Hz, 2H), 5.13 (d, *J* = 12.4 Hz, 2H), 4.91 (s, 1H), 2.53 (sepdd, *J* = 7.2 Hz, 7.2 Hz, 1.2 Hz, 1H), 1.12 (d, *J* = 7.2 Hz, 6H); ^{13}C NMR (400 MHz, $CDCl_3$) δ 185.4, 168.5 (2C), 152.2, 137.0, 135.9, 135.4 (2C), 135.1, 129.2 (2C), 128.63, 128.59 (4C), 128.51, 128.3 (2C), 128.2 (4C), 127.10, 127.05 (2C), 124.61, 124.56, 124.3, 117.8, 110.6, 67.4 (2C), 58.1, 50.9, 31.3, 21.7 (2C); IR (ATR) ν 3063, 3033, 2959, 2359, 1732, 1655, 1604, 1524, 1386, 1177, 1141, 907 cm^{-1}; HRMS (ESI-TOF) [M + Na]$^+$ calcd for $C_{38}H_{35}NNaO_5^+$ m/z 608.2407, found 608.2409.

Dibenzyl 2-(3-acetyl-1-benzyl-1*H*-indol-5-yl)malonate (3s)

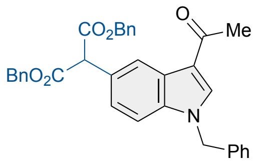

A white amorphous solid (58.2 mg, 55% yield): TLC R_f = 0.2 (*n*-hexane/EtOAc, 5/2); ^{1}H NMR (400 MHz, $CDCl_3$) δ 8.37 (d, *J* = 2.0 Hz, 1H), 7.70 (s, 1H), 7.38 (dd, *J* = 8.8 Hz, 2.0 Hz, 1H), 7.34-7.23 (m, 14H), 7.13 (dd, *J* = 7.6 Hz, 2.0 Hz, 2H), 5.28 (s, 2H), 5.18 (d,

J = 12.4 Hz, 2H), 5.13 (d, *J* = 12.4 Hz, 2H), 4.91 (s, 1H), 2.46 (s, 3H); ^{13}C NMR (400 MHz, $CDCl_3$) δ 193.1, 168.5 (2C), 136.9, 135.8, 135.6, 135.4 (2C), 129.2 (2C), 128.6 (4C), 128.37, 128.35 (2C), 128.2 (4C), 127.12 (2C), 127.05, 126.5, 124.4, 124.1, 117.6, 110.6, 67.5 (2C), 58.0, 50.9, 27.8; IR (ATR) ν 3063, 3032, 2949, 2359, 1730, 1645, 1619, 1527, 1213, 1177, 1140, 908 cm^{-1}; HRMS (ESI-TOF) $[M + Na]^+$ calcd for $C_{34}H_{29}NNaO_5^+$ m/z 554.1938, found 554.1935.

Diallyl (*E*)-2-(1-benzyl-3-(but-2-enoyl)-1*H*-indol-5-yl)malonate (3t)

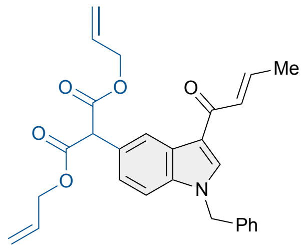

A pale orange amorphous solid (57.7 mg, 63% yield): TLC R_f = 0.25 (*n*-hexane/EtOAc, 5/2); ^{1}H NMR (400 MHz, $CDCl_3$) δ 8.46 (d, *J* = 1.6 Hz, 1H), 7.78 (s, 1H), 7.44 (dd, *J* = 8.4 Hz, 1.6 Hz, 1H), 7.36-7.28 (m, 4H), 7.15 (dd, *J* = 8.0 Hz, 2.0 Hz, 2H), 7.04 (qd, *J* = 15.2 Hz, 7.2 Hz, 1H), 6.73 (dd, *J* = 15.2 Hz, 1.6 Hz, 1H), 5.88 (tdd, *J* = 16.8 Hz, 10.4 Hz, 6.0 Hz, 2H), 5.32 (s, 2H), 5.28 (dd, *J* = 16.8 Hz, 1.2 Hz, 2H), 5.20 (dd, *J* = 10.4 Hz, 1.2 Hz, 2H), 4.87 (s, 1H), 4.64 (m, 4H), 1.96 (dd, *J* = 7.2 Hz, 1.6 Hz, 3H); ^{13}C NMR (400 MHz, $CDCl_3$) δ 184.8, 168.4 (2C), 141.0, 137.0, 135.7, 135.1, 131.6 (2C), 129.2 (2C),

128.7, 128.4, 127.15, 127.13 (2C), 127.06, 124.6, 124.5, 118.7 (2C), 117.6, 110.6, 66.3 (2C), 58.0, 50.9, 18.4; IR (ATR) ν 3033, 2943, 1732, 1658, 1605, 1524, 1206, 1178, 1143, 991, 966, 935 cm^{-1}; HRMS (ESI-TOF) $[M + Na]^+$ calcd for $C_{28}H_{27}NNaO_5^+$ m/z 480.1781, found 480.1781.

1-benzyl 3-methyl (*E*)-2-(1-benzyl-3-(but-2-enoyl)-1*H*-indol-5-yl)malonate (3u)

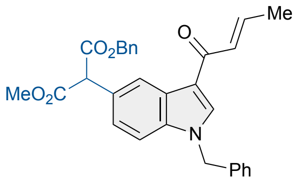

A brown amorphous solid (65.8 mg, 68% yield): TLC R_f = 0.2 (*n*-hexane/EtOAc, 5/2); ^{1}H NMR (400 MHz, $CDCl_3$) δ 8.46 (d, *J* = 2.0 Hz, 1H), 7.76 (s, 1H), 7.40 (dd, *J* = 8.4 Hz, 2.0 Hz, 1H), 7.34-7.25 (m, 9H), 7.14 (dd, *J* = 7.6 Hz, 2.0 Hz, 2H), 7.03 (qd, *J* = 14.8 Hz, 7.2 Hz, 1H), 6.71 (dd, *J* = 14.8 Hz, 1.6 Hz, 1H), 5.29 (s, 2H), 5.21 (d, *J* = 12.4 Hz, 1H), 5.15 (d, *J* = 12.4 Hz, 1H), 4.87 (s, 1H), 3.71 (s, 3H), 1.95 (dd, *J* = 7.2 Hz, 1.6 Hz, 3H); ^{13}C NMR (400 MHz, $CDCl_3$) δ 184.8, 169.1, 168.5, 140.9, 137.0, 135.8, 135.5, 135.1, 129.2 (2C), 128.7, 128.63, 128.59 (2C), 128.35, 128.32, 128.2 (2C), 127.12 (2C), 127.09, 124.54, 124.50, 117.6, 110.6, 67.4, 57.9, 52.9, 50.9, 18.4; IR (ATR) ν 3033, 2952, 1731, 1658, 1604, 1523, 1388, 1259, 1213, 1176, 1143, 907 cm^{-1}; HRMS (ESI-TOF) $[M + Na]^+$ calcd for $C_{30}H_{27}NNaO_5^+$ m/z 504.1781, found 504.1799.

1-benzyl 3-(tert-butyl) (*E*)-2-(1-benzyl-3-(but-2-enoyl)-1*H*-indol-5-yl)malonate (3v)

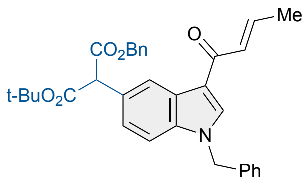

A pale orange amorphous solid (78.4 mg, 75% yield): TLC R_f = 0.25 (*n*-hexane/EtOAc, 3/1); ^{1}H NMR (400 MHz, $CDCl_3$) δ 8.45 (d, *J* = 1.2 Hz, 1H), 7.74 (s, 1H), 7.40 (dd, *J* = 8.8 Hz, 1.6 Hz, 1H), 7.34-7.24 (m, 9H), 7.13 (dd, *J* = 7.2 Hz, 2.0 Hz, 2H), 7.03 (qd, *J* = 15.2 Hz, 6.8 Hz, 1H), 6.71 (dd, *J* = 15.2 Hz, 1.2 Hz, 1H), 5.26 (s, 2H), 5.22 (d, *J* = 12.4 Hz, 1H), 5.13 (d, *J* = 12.4 Hz, 1H), 4.77 (s, 1H), 1.94 (dd, *J* = 6.8 Hz, 1.2 Hz, 3H), 1.39 (s, 9H); ^{13}C NMR (400 MHz, $CDCl_3$) δ 184.9, 168.9, 167.6, 140.8, 136.9, 135.9, 135.7, 135.1, 129.1 (2C), 128.8, 128.62, 128.57 (2C), 128.4, 128.3 (2C), 127.6, 127.1 (2C), 127.0, 124.6, 124.5, 117.6, 110.4, 82.4, 67.2, 59.1, 50.9, 27.9 (3C), 18.3; IR (ATR) ν 3033, 2978, 2934, 1726, 1658, 1604, 1524, 1389, 1369, 1284, 1136, 908 cm^{-1}; HRMS (ESI-TOF) [M + Na]$^+$ calcd for $C_{33}H_{33}NNaO_5^+$ m/z 546.2251, found 546.2255.

1-allyl 3-benzyl (*E*)-2-(1-benzyl-3-(but-2-enoyl)-1*H*-indol-5-yl)malonate (3w)

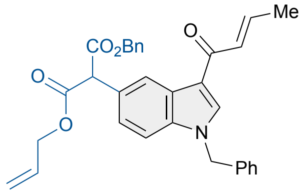

A brown amorphous solid (76.4 mg, 75% yield): TLC R_f = 0.2 (*n*-hexane/EtOAc, 5/2); ^{1}H NMR (400 MHz, $CDCl_3$) δ 8.48 (d, *J* = 1.6 Hz, 1H), 7.73 (s, 1H), 7.40 (dd, *J* = 8.8 Hz, 2.0 Hz, 1H), 7.32-7.23 (m, 9H), 7.12 (dd, *J* = 7.2 Hz, 1.6 Hz, 2H), 7.03 (qd, *J* = 14.8 Hz, 6.8 Hz, 1H), 6.70 (dd, *J* = 14.8 Hz, 1.6 Hz, 1H), 5.82 (tdd, *J* = 14.4 Hz, 10.4 Hz, 5.6 Hz, 1H), 5.26 (s, 2H), 5.22-5.13 (m, 4H), 4.89 (s, 1H), 4.61 (m, 2H), 1.93 (dd, *J* = 6.8 Hz, 1.6 Hz, 3H); ^{13}C NMR (400 MHz, $CDCl_3$) δ 184.8, 168.5, 168.3, 140.9, 137.0, 135.8, 135.5, 135.2, 131.6, 129.1 (2C), 128.74, 128.64, 128.60 (2C), 128.34, 128.32, 128.2 (2C), 127.12 (2C), 127.08, 124.55, 124.51, 118.7, 117.6, 110.6, 67.4, 66.3, 58.0, 50.9, 18.4; IR (ATR) ν 3033, 2942, 1730, 1658, 1604, 1523, 1207, 1176, 1139, 1061, 962, 906 cm^{-1}; HRMS (ESI-TOF) $[M + Na]^+$ calcd for $C_{32}H_{29}NNaO_5^+$ m/z 530.1938, found 530.1931.

Dibenzyl 2-(9-benzyl-4-oxo-2,3,4,9-tetrahydro-1*H*-carbazol-6-yl)malonate (3x)

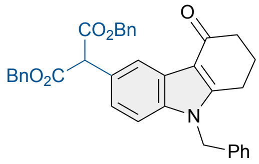

A white amorphous solid (33.6 mg, 30% yield): TLC R_f = 0.25 (*n*-hexane/EtOAc, 1/1); ^{1}H NMR (400 MHz, $CDCl_3$) δ 8.27 (d, *J* = 2.0 Hz, 1H), 7.37 (dd, *J* = 8.8 Hz, 2.0 Hz, 1H),

7.32-7.22 (m, 14H), 7.02 (dd, J = 7.6 Hz, 1.6 Hz, 2H), 5.28 (s, 2H), 5.19 (d, J = 12.4 Hz, 2H), 5.13 (d, J = 12.4 Hz, 2H), 4.90 (s, 1H), 2.84 (t, J = 6.4 Hz, 2H), 2.57 (t, J = 6.4 Hz, 2H), 2.21 (tt, J = 6.4 Hz, 6.4 Hz, 2H); ^{13}C NMR (400 MHz, $CDCl_3$) δ 193.9, 168.5 (2C), 152.3, 137.0, 135.9, 135.4 (2C), 129.2 (2C), 128.6 (4C), 128.3 (2C), 128.2 (4C), 128.1, 127.1, 126.2 (2C), 125.0, 124.1, 123.2, 113.3, 110.1, 67.4 (2C), 58.0, 47.2, 37.9, 23.4, 22.4; IR (ATR) ν 3062, 3033, 2945, 2360, 1730, 1643, 1621, 1534, 1455, 1136, 1097, 907 cm^{-1}; HRMS (ESI-TOF) [M + Na]$^+$ calcd for $C_{36}H_{31}NNaO_5^+$ m/z 580.2094, found 580.2090.

4. Derivatization of the Product

Dibenzyl 2-(1-benzyl-1*H*-indol-5-yl)malonate (6)

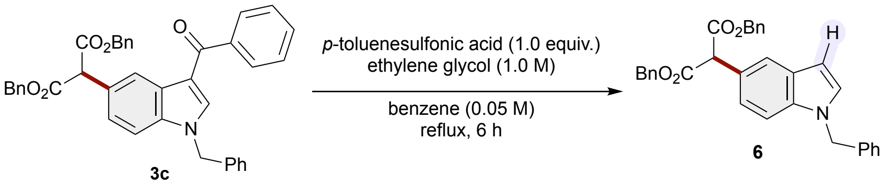

A solution of **3c** (0.1 mmol, 59.4 mg), ethylene glycol (0.1 mL), and *p*-toluenesulfonic acid monohydrate (0.1 mmol, 1 equiv., 21 mg) in benzene (2 mL) was stirred under reflux for 6 hours. Upon completion of the reaction (monitored by TLC), the reaction mixture was allowed to cool to room temperature. After the addition of a saturated aqueous $NaHCO_3$ solution, the mixture was extracted three times with EtOAc. The combined organic layers were washed with a saturated aqueous $NaHCO_3$ solution, then with water, dried over Na_2SO_4, and concentrated under reduced pressure. Finally, the residue was purified by flash chromatography on silica gel to afford the title compound.

Pink oil (35.0 mg, 71% yield): TLC R_f = 0.4 (*n*-hexane/EtOAc, 5/2); ^{1}H NMR (400 MHz, $CDCl_3$) δ 7.66 (d, *J* = 1.2 Hz, 1H), 7.31-7.20 (m, 15H), 7.14-7.08 (m, 3H), 6.52 (dd, *J* = 3.2 Hz, 0.8 Hz, 1H), 5.30 (s, 2H), 5.19 (d, *J* = 12.4 Hz, 2H), 5.13 (d, *J* = 12.4 Hz, 2H), 4.82 (s, 1H); ^{13}C NMR (400 MHz, $CDCl_3$) δ 168.7 (2C), 137.5, 136.2, 135.6 (2C), 129.0, 128.91, 128.89 (2C), 128.6 (4C), 128.3 (2C), 128.2 (4C), 127.8, 126.9 (2C), 123.7, 123.0,

122.2, 110.0, 102.1, 67.4 (2C), 58.0, 50.3; IR (ATR) ν 3063, 3033, 2947, 1730, 1606, 1586, 1511, 1454, 1262, 1225, 1175, 1132 cm^{-1}; HRMS (ESI-TOF) $[M + Na]^+$ calcd for $C_{32}H_{27}NNaO_4^+$ m/z 512.1832, found 512.1830.

Dibenzyl 2-allyl-2-(3-benzoyl-1-benzyl-1*H*-indol-5-yl)malonate (7)

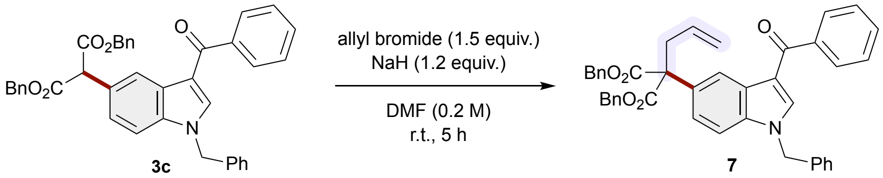

A solution of **3c** (59.4 mg, 0.10 mmol) in DMF (0.25 mL) was added dropwise to a suspension of NaH (60% suspension in oil; 4.8 mg, 1.2 equiv., 0.12 mmol) in DMF (0.25 mL) at 0 °C. After stirring for 30 min at room temperature, allyl bromide (0.0127 mL, 1.5 equiv., 0.15 mmol) was added dropwise to the reaction mixture. After 5 hours of stirring, the reaction was quenched with water. The mixture was extracted three times with EtOAc. The combined organic layers were washed with brine, dried over Na_2SO_4, and concentrated under reduced pressure. Finally, the residue was purified by flash chromatography on silica gel to afford the title compound.

A white amorphous solid (41.0 mg, 65% yield): TLC $R_f = 0.4$ (*n*-hexane/EtOAc, 3/1); ^1H NMR (400 MHz, $CDCl_3$) δ 8.56 (d, J = 1.6 Hz, 1H), 7.83-7.79 (m, 2H), 7.62 (s, 1H), 7.54 (tt, J = 7.2 Hz, 2.4 Hz, 1H), 7.47 (tt, J = 8.4 Hz, 1.2 Hz, 2H), 7.39-7.30 (m, 4H), 7.27-

7.19 (m, 11H), 7.14 (dd, *J* = 7.2 Hz, 1.6 Hz, 2H), 5.80 (tdd, *J* = 17.2 Hz, 10.4 Hz, 7.2 Hz, 1H), 5.33 (s, 2H), 5.16 (d, *J* = 12.4 Hz, 2H), 5.11 (d, *J* = 12.4 Hz, 2H), 5.04 (dd, *J* = 17.2 Hz, 1.2 Hz, 1H), 4.98 (dd, *J* = 10.4 Hz, 1.2 Hz, 1H), 3.25 (d, *J* = 7.2 Hz, 2H); ^{13}C NMR (600 MHz, $CDCl_3$) δ 190.8, 170.5 (2C), 140.8, 137.5, 136.5, 135.8, 135.5 (2C), 133.1, 131.3, 131.1, 129.1 (2C), 128.8 (2C), 128.5 (4C), 128.4 (2C), 128.3, 128.19 (4C), 128.16 (2C), 127.2, 126.9(2C), 125.3, 122.1, 119.1, 116.4, 110.0, 67.3 (2C), 63.2, 51.0 40.0; IR (ATR) ν 3062, 3033, 2926, 1730, 1625, 1575, 1522, 1380, 1219, 1173, 911, 899 cm^{-1}; HRMS (ESI-TOF) $[M + Na]^+$ calcd for $C_{42}H_{35}NNaO_5^+$ m/z 656.2407, found 656.2406.

2-(3-Benzoyl-1-benzyl-1*H*-indol-5-yl)acetic acid (8)

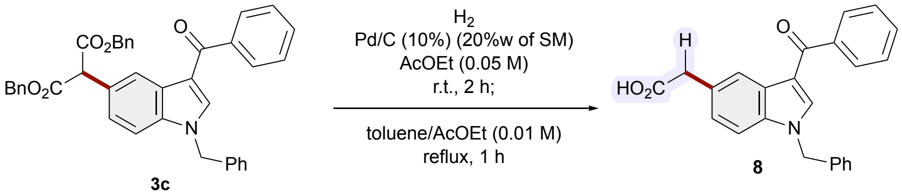

A suspension of **3c** (59.4 mg, 0.1 mmol) and 10% palladium on carbon (11.9 mg) in EtOAc (2.0 mL) was stirred for 2 hours at room temperature under a hydrogen atmosphere (1 atm). The mixture was then filtered through celite to remove insoluble material. The filtrate was concentrated to give a foam. This residue was dissolved in toluene/EtOAc (1:1, 10 mL) and the solution was stirred under reflux for 1 hour. After removing the solvent under reduced pressure, the obtained crude residue was purified by flash

chromatography on silica gel to afford the product **8**.

A white solid (28.2 mg, 76% yield): m.p. 123-125 °C; TLC R_f = 0.3 (EtOAc); ^{1}H NMR (400 MHz, CD_3COCD_3) δ 8.25 (s, 1H), 7.92 (s, 1H), 7.71 (d, J = 7.2 Hz, 2H), 7.44 (tt, J = 7.2 Hz, 1.2 Hz, 1H), 7.40-7.35 (m, 2H), 7.30 (d, J = 8.4 Hz, 1H), 7.20-7.09 (m, 6H), 5.43 (s, 2H), 3.61 (s, 2H); ^{13}C NMR (600 MHz, CD_3COCD_3) δ 189.8, 172.7, 141.2, 138.4, 137.1, 136.2, 131.0, 129.4, 128.8 (2C), 128.6 (2C), 128.3 (2C), 127.9, 127.7, 127.2 (2C), 125.2, 123.1, 115.2, 110.7, 50.3, 40.9; IR (ATR) ν 3030, 2924, 1702, 1614, 1572, 1520, 1455, 1381, 1246, 1173, 1024, 851 cm^{-1}; HRMS (ESI-TOF) $[M + Na]^+$ calcd for $C_{24}H_{19}NNaO_3^+$ m/z 392.1257, found 392.1253.

5. Reaction employing C5-Substituted Indole

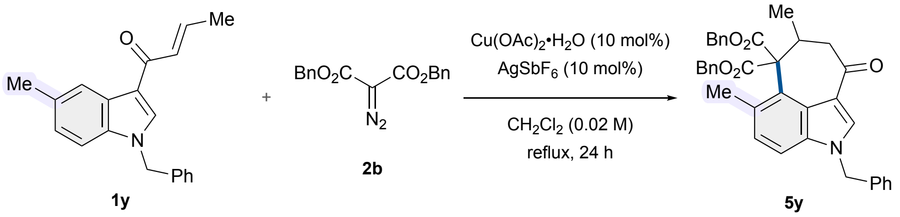

$Cu(OAc)_2 \cdot H_2O$ (4.0 mg, 10 mol%, 0.02 mmol), $AgSbF_6$ (6.9 mg, 10 mol%, 0.02 mmol), and indole substrate **1y** (0.2 mmol, 1.0 equiv.) were introduced into a pre-dried 50-mL pear-shaped flask equipped with a magnetic stir bar. After injecting dichloromethane (5 mL) into the flask under an argon atmosphere, the solution was stirred at room temperature for 20 min. A solution of diazo compound **2b** (0.4 mmol, 2.0 equiv.) in dichloromethane (5 mL) was then introduced into the reaction flask. The resulting solution was continuously stirred under reflux for 24 hours. After removing the solvent under reduced pressure, the obtained crude residue was purified by flash chromatography on silica gel (*n*-hexane/EtOAc) to afford the resultant product **5y**.

Colourless oil (73.7 mg, 64% yield): TLC R_f = 0.25 (*n*-hexane/EtOAc, 2/1); ^{1}H NMR (400 MHz, $CDCl_3$) δ 7.94 (s, 1H), 7.37-7.14 (m, 19H), 7.05 (d, J = 8.4 Hz, 1H), 5.28 (s, 2H), 5.25-5.08 (m, 4H), 3.27-3.18 (m, 1H), 2.90-2.80 (br, 1H), 2.77-2.52 (br, 1H), 1.23-1.14 (br, 3H); ^{13}C NMR (400 MHz, $CDCl_3$) δ 195.1, 170.63, 170.59, 136.6, 135.3 (2C), 135.2, 135.1, 134.6, 129.2 (2C), 128.8, 128.7, 128.60 (2C), 128.57 (2C), 128.49 (2C),

128.43 (2C), 128.42 (2C), 128.0, 127.6 (2C), 127.5, 124.6, 110.4, 67.59, 67.55, 53.6, 51.1, 47.8, 35.9, 21.8, 14.3; IR (ATR) ν 3063, 3032, 2947, 1725, 1643, 1522, 1497, 1454, 1207, 1170, 1027, 907 cm^{-1}; HRMS (ESI-TOF) $[M + Na]^+$ calcd for $C_{37}H_{33}NNaO_5^+$ m/z 594.2251, found 594.2264.

6. Preparation of Substrates

The substrates **1a**,[1] **1b**,[2] **1c**,[2] **1d**,[3] **1g**,[3] **1h**,[4] **1j**,[5] **1k**,[1] **1l**,[1] **1o**,[1] **1p**,[1] **1q**,[1] **1r**,[6] **1s**,[7] **2a**,[6] **2b**,[6] **2c**,[6] **2d**,[8] **2e**[9] and **2f**[10] were synthesized by the reported procedure. The substrates **1e**, **1f**, **1i**, **1m** and **1n** were synthesized as below.

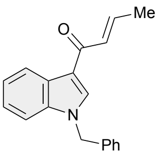
1a

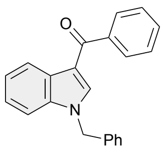
1b

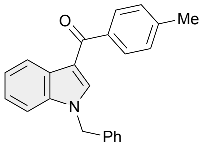
1c

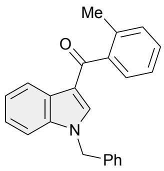
1d

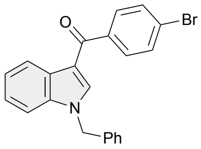
1e

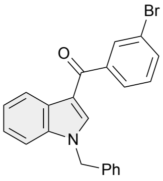
1f

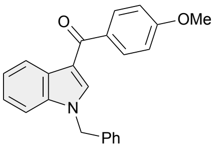
1g

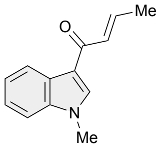
1h

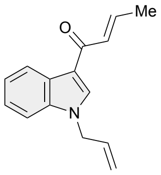
1i

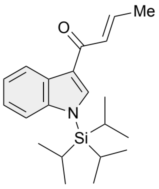
1j

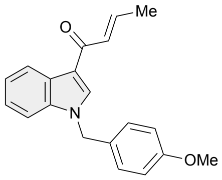
1k

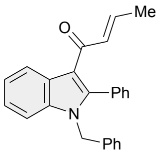
1l

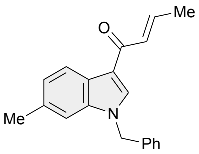
1m

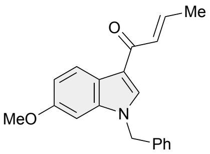
1n

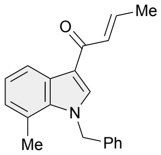
1o

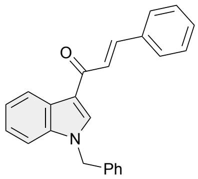
1p

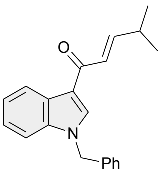
1q

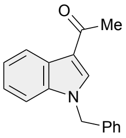
1r

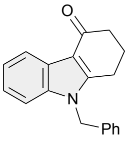
1s

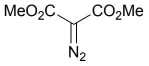
2a

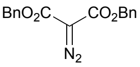
2b

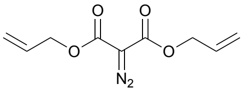
2c

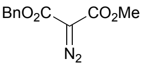
2d

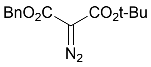
2e

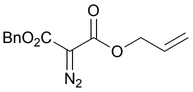
2f

General procedure for the preparation of indole derivatives (1e, 1f, 1m, 1n)

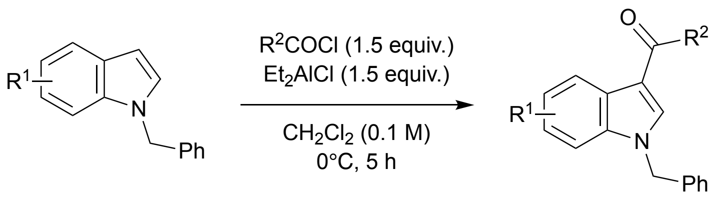

To a stirred solution of *N*-benzyl indole derivative (1.0 equiv.) in CH_2Cl_2 was added Et_2AlCl (0.87 mol/L in hexane; 1.5 equiv.) at 0 °C. The reaction mixture was stirred for 30 min at the same temperature. To this solution was added dropwise an acid chloride (1.5 equiv.) at 0 °C. The resulting solution was stirred for 5 hours at 0 °C. The reaction was quenched by the addition of saturated aqueous $NaHCO_3$ solution followed by H_2O, and the layers were separated. The aqueous layer was extracted with CH_2Cl_2, and the combined organic layers were washed with brine, dried over Na_2SO_4, and concentrated under reduced pressure. The crude mixture was purified by flash chromatography on silica gel (n-hexane/EtOAc).

(1-benzyl-1*H*-indol-3-yl)(4-bromophenyl)methanone (1e)

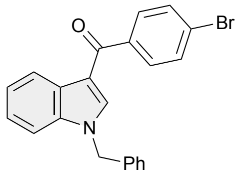

A yellow solid (257.0 mg, 66% yield, 1 mmol scale): m.p. 117-119 °C; TLC R_f = 0.4 (*n*-hexane/EtOAc, 5/1); ^1H NMR (400 MHz, $CDCl_3$) δ 8.40 (dd, *J* = 8.4 Hz, 1.2 Hz, 1H),

7.71-7.67 (m, 2H), 7.63-7.58 (m, 3H), 7.35-7.29 (m, 6H), 7.14 (dd, *J* = 7.6 Hz, 1.6 Hz, 2H), 5.37 (s, 2H); ^{13}C NMR (600 MHz, $CDCl_3$) δ 189.7, 139.6, 137.2, 137.1, 135.8, 132.5, 132.0, 131.7 (2C), 130.4 (2C), 129.2, 128.4, 127.4, 126.9, 126.0, 124.1, 123.1, 122.8, 115.9, 110.4, 50.9; IR (ATR) ν 3057, 3030, 2925, 1790, 1725, 1611, 1585, 1520, 1379, 1170, 1010, 875 cm^{-1}; HRMS (ESI-TOF) [M + Na]$^+$ calcd for $C_{22}H_{16}BrNNaO^+$ m/z 412.0308, found 412.0315.

(1-benzyl-1*H*-indol-3-yl)(3-bromophenyl)methanone (1f)

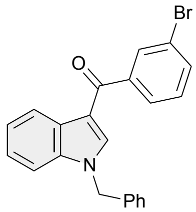

A white solid (300.2 mg, 77% yield, 1 mmol scale): m.p. 123-125 °C; TLC R_f = 0.4 (*n*-hexane/EtOAc, 5/1); ^{1}H NMR (400 MHz, $CDCl_3$) δ 8.41 (dd, *J* = 7.2 Hz, 1.6 Hz, 1H), 7.94 (s, 1H), 7.73 (dd, *J* = 7.2 Hz, 0.8 Hz, 1H), 7.66 (dd, *J* = 7.2 Hz, 0.8 Hz, 1H), 7.56 (s, 1H), 7.37-7.30 (m, 7H), 7.15 (dd, *J* = 7.2 Hz, 1.2 Hz, 2H), 5.39 (s, 2H); ^{13}C NMR (600 MHz, $CDCl_3$) δ 189.1, 142.7, 137.4, 137.2, 135.7, 134.1, 131.7, 130.0, 129.2, 128.4, 127.4, 127.1, 126.8, 124.2, 123.2, 123.0, 122.8, 122.7, 122.6, 115.8, 110.5, 51.0; IR (ATR) ν 3108, 3060, 3031, 1621, 1575, 1560, 1519, 1463, 1378, 1193, 1176, 883 cm^{-1}; HRMS (ESI-TOF) [M + Na]$^+$ calcd for $C_{22}H_{16}BrNNaO^+$ m/z 412.0308, found 412.0313.

(*E*)-1-(1-benzyl-6-methyl-1*H*-indol-3-yl)but-2-en-1-one (1m)

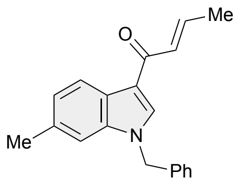

A yellow green solid (241.6 mg, 56% yield, 1.5 mmol scale): m.p. 109-111 °C; TLC R_f = 0.25 (*n*-hexane/EtOAc, 4/1); ^{1}H NMR (600 MHz, $CDCl_3$) δ 8.36 (dd, *J* = 8.4 Hz, 1.8 Hz, 1H), 7.68 (s, 1H), 7.32-7.27 (m, 3H), 7.13-7.10 (m, 3H), 7.05 (s, 1H), 7.02 (qd, *J* = 14.4 Hz, 6.6 Hz, 1H), 6.71 (dd, *J* = 14.4 Hz, 1.8 Hz, 1H), 5.25 (s, 2H), 2.42 (s, 3H), 1.92 (dd, *J* = 6.6 Hz, 1.8 Hz, 3H); ^{13}C NMR (600 MHz, $CDCl_3$) δ 184.9, 140.5, 137.7, 136.1, 134.4, 133.8, 129.1, 128.9 (2C), 128.2, 127.0 (2C), 124.9, 124.5, 122.8, 117.6, 110.1, 50.6, 22.0, 18.3; IR (ATR) ν 3103, 3030, 2912, 2371, 2329, 1705, 1657, 1600, 1570, 1523, 1379, 1175 cm^{-1}; HRMS (ESI-TOF) $[M + Na]^+$ calcd for $C_{20}H_{19}NNaO^+$ m/z 312.1359, found 312.1360.

(*E*)-1-(1-benzyl-6-methoxy-1*H*-indol-3-yl)but-2-en-1-one (1n)

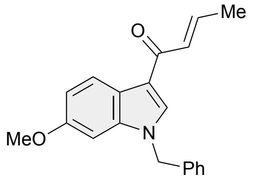

A yellow solid (286.7 mg, 63% yield, 1.5 mmol scale): m.p. 110-112 °C; TLC R_f = 0.25

(n-hexane/EtOAc, 4/1); ^1H NMR (400 MHz, $CDCl_3$) δ 8.36 (d, J = 8.8 Hz, 1H), 7.70 (s, 1H), 7.37-7.31 (m, 3H), 7.16 (dd, J = 7.6 Hz, 1.6 Hz, 2H), 7.03 (qd, J = 14.8 Hz, 6.8 Hz, 1H), 6.95 (dd, J = 8.8 Hz, 2.0 Hz, 1H), 6.73 (m, 2H), 5.30 (s, 2H), 3.80 (s, 3H), 1.95 (dd, J = 6.8 Hz, 1.6 Hz, 3H); ^{13}C NMR (600 MHz, $CDCl_3$) δ 184.9, 157.4, 140.4, 138.2, 136.0, 134.2, 129.1, 128.7 (2C), 128.2, 127.0 (2C), 123.8, 121.2, 117.6, 111.8, 94.1, 55.7, 50.7, 18.3; IR (ATR) ν 3110, 3032, 2935, 2833, 2372, 2335, 1656, 1600, 1574, 1523, 1496, 911 cm^{-1}; HRMS (ESI-TOF) $[M + Na]^+$ calcd for $C_{20}H_{19}NNaO_2^+$ m/z 328.1308, found 328.1318.

Experimental procedure for the preparation of the *N*-allyl indole derivative (1i)

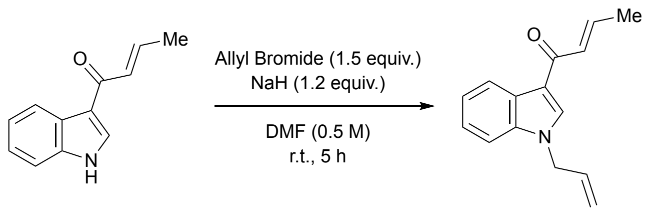

A solution of (E)-1-(1H-indol-3-yl)-3-phenylprop-2-en-1-one (277.8 mg, 1.0 equiv., 1.5 mmol) in DMF (1.5 mL) was added dropwise to a suspension of NaH (60% suspension in oil; 72 mg, 1.2 equiv., 1.8 mmol) in DMF (1.5 mL) at 0 °C. After stirring for 30 minutes at room temperature, allyl bromide (0.195 mL, 1.5 equiv., 2.25 mmol) was added to the reaction mixture. After stirring for 5 hours, the reaction was quenched by the addition of H_2O. The mixture was extracted three times with EtOAc, and the combined organic layers

were washed with brine, dried over Na_2SO_4, and concentrated under reduced pressure. The crude mixture was purified by flash chromatography on silica gel to afford the title compound as a pale yellow oil (144.8 mg, 43% yield).

(*E*)-1-(1-allyl-1*H*-indol-3-yl)but-2-en-1-one (1i)

A pale yellow oil (144.8 mg, 43% yield); TLC $R_f = 0.25$ (*n*-hexane/EtOAc, 3/1); ^{1}H NMR (600 MHz, $CDCl_3$) δ 8.48 (dd, *J* = 8.4 Hz, 1.2 Hz, 1H), 7.73 (s, 1H), 7.30-7.26 (m, 3H), 7.03 (qd, *J* = 15.0 Hz, 6.6 Hz, 1H), 6.75 (dd, *J* = 15.0 Hz, 1.2 Hz, 1H), 5.96 (tdd, *J* = 16.8 Hz, 10.2 Hz, 6.0 Hz, 1H), 5.25 (d, *J* = 10.2 Hz, 1H), 5.12 (d, *J* = 16.8 Hz, 1H), 4.69 (d, *J* = 6.0 Hz, 2H), 1.94 (dd, *J* = 6.6 Hz, 1.2 Hz, 3H); ^{13}C NMR (600 MHz, $CDCl_3$) δ 184.9, 140.6, 137.1, 134.6, 132.2, 128.9, 127.0, 123.6, 123.1, 122.6, 118.7, 117.3, 110.1, 49.4, 18.3; IR (ATR) ν 3102, 3054, 2967, 2911, 2371, 2360, 1657, 1599, 1520, 1385, 960, 900, 809 cm^{-1}; HRMS (ESI-TOF) $[M + Na]^+$ calcd for $C_{15}H_{15}NNaO^+$ m/z 248.1046, found 248.1052.

7. Single Crystal X-Ray Diffraction Analysis

Crystal Data for $C_{24}H_{23}NO_5$ (**3a**, *M* =405.43 g/mol): triclinic, space group P-1 (no. 2), *a* = 8.1618(2) Å, *b* = 11.0202(3) Å, *c* = 12.1858(3) Å, α = 80.226(6)°, β = 71.038(5)°, γ = 81.929(6)°, *V* = 1017.24(6) Å^3, *Z* = 2, *T* = 93.15 K, μ(Cu Kα) = 0.760 mm^{-1}, *Dcalc* = 1.324 g/cm^3, 11746 reflections measured (7.74° ≤ 2Θ ≤ 136.372°), 3652 unique (R_{int} = 0.0356, R_{sigma} = 0.0445) which were used in all calculations. The final R_1 was 0.0429 (I > 2σ(I)) and wR_2 was 0.1165 (all data). The ellipsoid contour probability level in the ORTEP is 50%.

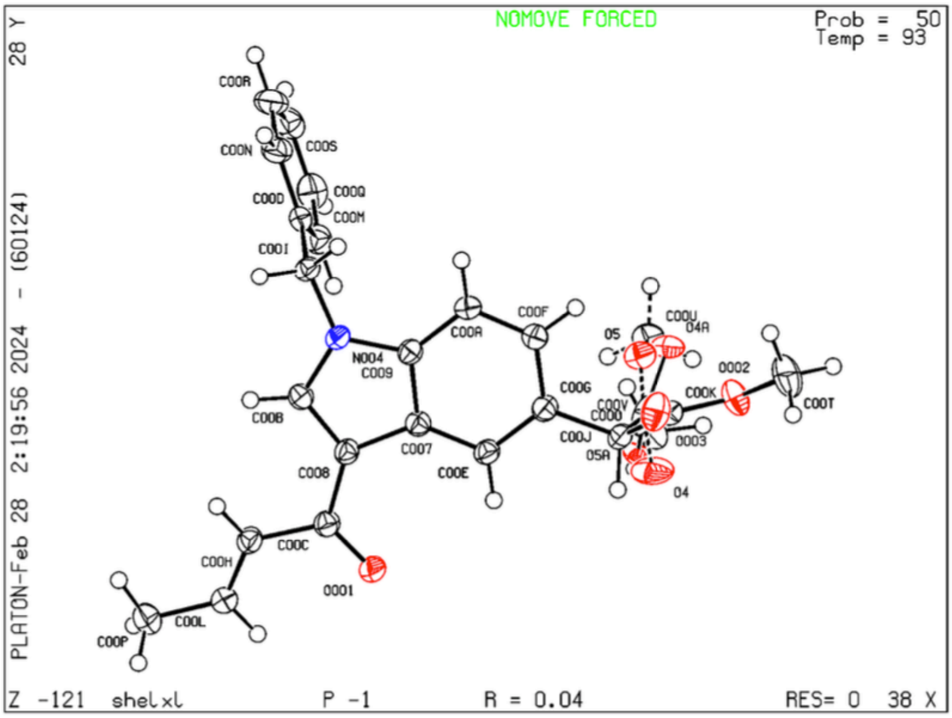

ORTEP of **3a**

CCDC No. 2448262

Bond precision:	C–C = 0.0023 A		Wavelength=1.54187
Cell:	a=8.1618(2)	b=11.0202(3)	c=12.1858(3)
	alpha=80.226(6)	beta=71.038(5)	gamma=81.929(6)
Temperature:	93 K		

	Calculated	Reported
Volume	1017.24(6)	1017.24(6)
Space group	P –1	P –1
Hall group	–P 1	–P 1
Moiety formula	C24 H23 N O5	C24 H23 N O5
Sum formula	C24 H23 N O5	C24 H23 N O5
Mr	405.43	405.43
Dx,g cm–3	1.324	1.324
Z	2	2
Mu (mm–1)	0.760	0.760
F000	428.0	428.0
F000'	429.37	
h,k,lmax	9,13,14	9,13,14
Nref	3723	3652
Tmin,Tmax	0.927,0.927	0.771,1.000
Tmin'	0.927	

Correction method= # Reported T Limits: Tmin=0.771 Tmax=1.000
AbsCorr = MULTI-SCAN

Data completeness= 0.981 Theta(max)= 68.186

R(reflections)= 0.0429(2920) wR2(reflections)= 0.1165(3652)

S = 1.054 Npar= 305

8. Computational Details

DFT calculations were performed with the Gaussian 16 program.[11] The molecular structure optimizations were carried out using the hybrid density functional method based on Becke's three-parameter exchange function and the Lee-Yang-Parr nonlocal correlation functional (UB3LYP),[12] and the Def2-SVPP basis set for H, C, N, O, F, Cu, and Sb. The vibrational frequencies were computed at the same level to check whether each optimized structure is at an energy minimum on the potential energy surface (no imaginary frequency) or a transition state (one imaginary frequency) and to evaluate its zero-point vibrational energy (ZPVE) and thermal corrections at 298.15 K. The intrinsic reaction coordinate (IRC) method was used to track minimum energy paths from transition structures to the corresponding local minima.[13] Single-point energies were calculated at the UCAM-B3LYP level using the 6-311+G** basis set for H, C, N, O, F and the SDD basis set for Cu and Sb in dichloromethane as the solvent.[14]

	E(UB3LYP/6-31G*/Def2-SVPP) (A.U.)	E(UCAM-B3LYP/6-311+G**/SDD) (A.U.)
**RT_{SM}+RT_{Cu}**	**-3834.99278**	**-2158.4868**
**$TS1_{C4}$**	**-3834.99221**	**-2158.4859**
**$CP1_{C4}$**	**-3835.03068**	**-2158.5471**
**CP2**	**-3835.05449**	**-2158.5608**
**TS2**	**-3835.04225**	**-2158.5532**
**CP3**	**-3835.06308**	**-2158.5612**
**TS3**	**-3835.04796**	**-2158.5554**
**CP4**	**-3835.05169**	**-2158.5611**
**CP5**	**-3835.11412**	**-2158.609**
**TS4**	**-3835.03607**	**-2158.5334**
**CP6**	**-3835.04246**	**-2158.5406**
**$TS1_{C5}$**	**-3834.97558**	**-2158.4637**
**$CP1_{C5}$**	**-3835.02573**	**-2158.534**
**$TS1_{C6}$**	**-3834.97623**	**-2158.4689**
**$CP1_{C6}$**	**-3835.03304**	**-2158.5314**
**$TS1_{C7}$**	**-3834.97356**	**-2158.4616**
**$CP1_{C7}$**	**-3835.01986**	**-2158.5212**
**$TS1_{C2}$**	**-3834.96459**	**-2158.4611**
**$CP1_{C2}$**	**-3835.01267**	**-2158.5277**

RT_{SM}+RT_{Cu}

Zero-point correction=	0.406078 (Hartree/Particle)
Thermal correction to Energy=	0.448867
Thermal correction to Enthalpy=	0.449812
Thermal correction to Gibbs Free Energy=	0.325263
Sum of electronic and zero-point Energies=	-3834.586698
Sum of electronic and thermal Energies=	-3834.543908
Sum of electronic and thermal Enthalpies=	-3834.542964
Sum of electronic and thermal Free Energies=	-3834.667512

Cartesian Coordinates

Atom	X	Y	Z
C	-1.74444800	-0.79799200	2.57320200
C	-0.34748800	-0.79052400	2.48599200
C	0.02401600	-2.07460000	1.92306600
C	-1.18627600	-2.77801000	1.69838000
N	-2.24435300	-1.95670200	2.09327400
H	-2.41510800	0.01013900	2.85715900
C	1.24676300	-2.70508200	1.64288200
H	2.19302100	-2.19025300	1.81320100
C	-1.21472600	-4.07745300	1.18514100
H	-2.15639800	-4.60426500	1.01175400
C	1.22599600	-4.00634100	1.13760000
H	2.17262100	-4.50665400	0.91143500
C	0.01282300	-4.68301900	0.90599700
H	0.02954200	-5.70212400	0.50715000
C	0.48604700	0.36279400	2.69004200
O	1.58893400	0.47467700	2.07192400
C	0.04290300	1.41905000	3.60963600
H	-0.86287600	1.23347800	4.19639500
C	0.71670100	2.57930200	3.74352400

H	1.61474800	2.71241500	3.13058900
C	0.32593300	3.70834100	4.63704500
H	-0.58413300	3.48975000	5.22103500
H	1.14597900	3.95627500	5.33879300
H	0.14767300	4.62105700	4.03576700
C	2.44064000	-0.44715900	-0.78249200
C	3.84720400	-0.84724500	-0.58420800
C	1.66572000	-1.10356800	-1.80971400
O	4.09833600	-1.55979600	0.36219200
O	1.93863600	-0.41820200	-2.78661700
O	4.71244200	-0.34080600	-1.44387400
O	0.88374700	-2.11781200	-1.67615700
C	0.06766200	-2.51432000	-2.81240300
H	-0.42792200	-1.62690500	-3.22925600
H	0.70771900	-3.01183100	-3.55878400
H	-0.67122000	-3.20811800	-2.39412500
C	6.09730100	-0.66555700	-1.23629500
H	6.42358300	-0.32884500	-0.23894900
H	6.25361500	-1.75300600	-1.32404700
H	6.64467300	-0.13150700	-2.02471700
Cu	1.56863100	1.10835100	0.16558500
H	0.53248500	5.36685500	-0.40869200
C	0.51978200	4.58093500	-1.18037900
C	1.02989400	3.28779700	-0.62306400
H	-0.52596900	4.40539700	-1.49091500
H	1.10477300	4.88347900	-2.06300900
O	1.58728700	2.42528300	-1.37469100
O	0.88677400	2.98291700	0.59998800
F	-3.07128000	0.72542900	0.49950500
Sb	-2.25729100	0.54465400	-1.24428700
F	-1.91739900	2.43901400	-1.30846300

F	-0.49982200	0.32899200	-0.36057500
F	-2.46018800	-1.37847600	-1.09846200
F	-1.34495400	0.29058000	-2.92616400
F	-3.95235700	0.72312500	-2.11115800
C	-3.64503100	-2.22993000	1.82534200
H	-3.79954800	-2.30605100	0.73647700
H	-3.95699900	-3.16732700	2.31817400
H	-4.25200300	-1.39715200	2.20831900

TS1*C4*

Zero-point correction= 0.406161 (Hartree/Particle)

Thermal correction to Energy= 0.447858

Thermal correction to Enthalpy= 0.448802

Thermal correction to Gibbs Free Energy= 0.328052

Sum of electronic and zero-point Energies= -3834.586050

Sum of electronic and thermal Energies= -3834.544354

Sum of electronic and thermal Enthalpies= -3834.543410

Sum of electronic and thermal Free Energies= -3834.664160

Cartesian Coordinates

Atom	X	Y	Z
C	-1.62978900	-0.46751700	2.72895500
C	-0.25728600	-0.21830500	2.61037500
C	0.34424700	-1.47409400	2.22652000
C	-0.71015400	-2.41544700	2.13363600
N	-1.90345000	-1.75690500	2.43695400
H	-2.43894700	0.23562200	2.91510700
C	1.66745900	-1.89251200	1.99909800
H	2.50164300	-1.20403000	2.12601500
C	-0.48517300	-3.75620600	1.81312400
H	-1.30761000	-4.47239700	1.74126400

C	1.89814700	-3.23781000	1.68520900
H	2.92473100	-3.56996100	1.50833400
C	0.83763400	-4.15463300	1.59004500
H	1.04647700	-5.19946200	1.34091700
C	0.38066400	1.07107400	2.61678200
O	1.46448000	1.24005900	1.98466100
C	-0.23804800	2.19393900	3.33249200
H	-1.07784300	1.97136000	3.99961900
C	0.18834300	3.46201900	3.16158900
H	1.01738900	3.62144700	2.46249900
C	-0.39686300	4.66697000	3.81851700
H	-1.22468200	4.41567800	4.50305300
H	0.37829600	5.21639800	4.38751300
H	-0.77392900	5.37249200	3.05286300
C	2.41776900	-0.50456800	-0.57115800
C	3.88524000	-0.65881500	-0.39289500
C	1.74793700	-1.35368700	-1.55349900
O	4.34769600	-1.26428800	0.54725400
O	2.01138600	-0.89943800	-2.65280500
O	4.58198900	-0.01717500	-1.31992700
O	0.98235100	-2.35201500	-1.23839800
C	0.25938500	-3.00548400	-2.31118400
H	-0.29819300	-2.25258100	-2.88476300
H	0.97027400	-3.55197900	-2.95239900
H	-0.43052300	-3.69242100	-1.80639300
C	6.01256600	-0.07013600	-1.20770300
H	6.33828500	0.36483200	-0.24874600
H	6.36536100	-1.11230300	-1.27316200
H	6.39427800	0.52045300	-2.05163700
Cu	1.44554400	1.17872100	-0.02841500
H	0.14188600	5.04645700	-1.74226000

C	0.15815400	4.07248400	-2.25673000
C	0.76011200	3.02254700	-1.37422300
H	-0.88287100	3.76208800	-2.45917300
H	0.70138400	4.13860700	-3.21229300
O	1.36816800	2.02080300	-1.87168700
O	0.64733900	3.06268800	-0.11241300
F	-3.18250300	0.41228700	0.45762700
Sb	-2.34075300	0.04320000	-1.24433500
F	-2.22862400	1.92600700	-1.63221100
F	-0.58379900	0.19380200	-0.34924300
F	-2.31716700	-1.83974900	-0.77316100
F	-1.39641000	-0.38015100	-2.87102600
F	-4.04147200	-0.13012600	-2.10151000
C	-3.22943400	-2.32609700	2.26663600
H	-3.37327900	-2.59972100	1.20890000
H	-3.35344500	-3.21403400	2.91083600
H	-3.97984200	-1.57035000	2.53902200

CP1*$_{C4}$*

Zero-point correction=	0.408463 (Hartree/Particle)
Thermal correction to Energy=	0.449741
Thermal correction to Enthalpy=	0.450685
Thermal correction to Gibbs Free Energy=	0.330957
Sum of electronic and zero-point Energies=	-3834.622221
Sum of electronic and thermal Energies=	-3834.580942
Sum of electronic and thermal Enthalpies=	-3834.579998
Sum of electronic and thermal Free Energies=	-3834.699727

Cartesian Coordinates

Atom	X	Y	Z
C	-1.20939700	-2.04590700	1.88155800

C	-0.07562000	-1.19601700	1.91645300
C	0.94294500	-1.86383400	1.21562300
C	0.40558400	-3.12559800	0.82274900
N	-0.92625200	-3.18421400	1.24250900
H	-2.21835900	-1.83731100	2.23062700
C	2.35143900	-1.52255500	0.89138300
H	2.86959000	-1.17451200	1.80585800
C	1.15919000	-4.09169700	0.18866400
H	0.71410000	-5.04568800	-0.10961300
C	3.11545400	-2.66928900	0.33009200
H	4.18252100	-2.48947400	0.17561000
C	2.54244400	-3.85498500	-0.05074800
H	3.14040900	-4.63507000	-0.52888900
C	0.00327300	0.14771000	2.49524900
O	0.99251100	0.86280200	2.22808100
C	-1.06984900	0.64401500	3.35515000
H	-1.81360300	-0.06309900	3.73400100
C	-1.19385700	1.96812900	3.59248700
H	-0.45050800	2.63134200	3.13149000
C	-2.28799500	2.60523800	4.37714000
H	-2.97953800	1.86830100	4.81843200
H	-1.86941000	3.23559400	5.18547300
H	-2.86787300	3.28672200	3.72470700
C	2.61163800	-0.31063800	-0.10696200
C	3.96015000	0.27465900	0.17166500
C	2.29533100	-0.57754800	-1.55018400
O	4.74906000	-0.17217000	0.98627000
O	2.92074000	-0.18709100	-2.50469600
O	4.19090300	1.39693000	-0.51973800
O	1.17195700	-1.32374800	-1.65576600
C	0.63100000	-1.48577000	-2.96966000

H	0.40371900	-0.50207900	-3.40718800
H	1.34048400	-2.02721800	-3.61897400
H	-0.30217000	-2.04933800	-2.83599300
C	5.47104300	2.00294600	-0.36135400
H	5.63256500	2.31218400	0.68534500
H	6.27177200	1.30249400	-0.65289000
H	5.46993800	2.87879800	-1.02587100
Cu	1.36503100	1.30928500	0.28359100
H	-0.36684600	5.15232400	-1.03940200
C	-0.33467500	4.20620700	-1.60370100
C	0.46107800	3.17545700	-0.85684300
H	-1.36741700	3.81916800	-1.68533800
H	0.07203700	4.36326200	-2.61438700
O	1.16064200	2.31476900	-1.47344300
O	0.40123200	3.09678700	0.41030300
F	-3.11374800	-0.11238400	0.95375200
Sb	-2.63700400	0.11022100	-0.91980700
F	-2.97172300	2.00097000	-0.82288600
F	-0.80564800	0.44632400	-0.29722000
F	-2.19454600	-1.79144000	-0.94199600
F	-2.03968100	0.25247100	-2.73793500
F	-4.43581800	-0.27896000	-1.44556100
C	-1.89310500	-4.20259800	0.85701500
H	-2.15034200	-4.07837700	-0.20704900
H	-1.48397800	-5.20780100	1.04868000
H	-2.80431000	-4.06790000	1.45769400

CP2

Zero-point correction= 0.409921 (Hartree/Particle)

Thermal correction to Energy= 0.450536

Thermal correction to Enthalpy= 0.451480

Thermal correction to Gibbs Free Energy= 0.334799
Sum of electronic and zero-point Energies= -3834.644571
Sum of electronic and thermal Energies= -3834.603955
Sum of electronic and thermal Enthalpies= -3834.603011
Sum of electronic and thermal Free Energies= -3834.719693

Cartesian Coordinates

Atom	X	Y	Z
C	-1.71638700	1.85261900	1.87802400
C	-1.83393400	0.45657000	1.66671300
C	-2.76281100	0.30320800	0.63164500
C	-3.22830300	1.61068000	0.28361300
N	-2.54939600	2.53618300	1.08166700
H	-1.03745100	2.37711900	2.54754400
C	-3.20717100	-0.88340000	-0.16131800
H	-3.57771500	-1.66410900	0.53232800
C	-4.21689200	1.81386500	-0.65121000
H	-4.57858700	2.82166400	-0.87751100
C	-4.30088300	-0.56609800	-1.12386200
H	-4.69481100	-1.41202000	-1.69570900
C	-4.78166700	0.69499800	-1.34105300
H	-5.57031200	0.87321600	-2.07654800
C	-1.16357000	-0.63420700	2.43238700
O	-1.64706700	-1.75932700	2.39233100
C	0.04056500	-0.30013900	3.22395800
H	0.52084300	0.66720500	3.05284300
C	0.57726500	-1.19376800	4.07608200
H	0.06929200	-2.16228600	4.17783600
C	1.81819100	-0.98210100	4.88010700
H	2.25706900	0.01557800	4.71234800
H	1.61466800	-1.10591200	5.96170900

H	2.57746400	-1.74483200	4.61883500
C	-2.00179900	-1.55540400	-0.84753200
C	-1.52423300	-2.80360600	-0.35703500
C	-1.26371000	-0.87288000	-1.85083400
O	-0.35444100	-3.24740000	-0.48152900
O	-0.16738400	-1.23801200	-2.34742400
O	-2.42995200	-3.55595400	0.27829400
O	-1.83230100	0.24565400	-2.31632600
C	-1.11824100	0.99754400	-3.30764100
H	-0.07953700	1.17216600	-2.99566000
H	-1.13384700	0.46121100	-4.27168000
H	-1.66008200	1.95078100	-3.39548900
C	-1.95545600	-4.67339500	1.03079800
H	-1.45193100	-5.40177800	0.37496200
H	-1.26016800	-4.33180400	1.81366400
H	-2.84993500	-5.12321700	1.48650500
Cu	1.11786900	-2.13120300	-1.17119900
H	5.26395200	-2.45498800	0.22099300
C	4.86758200	-1.69580800	-0.47064200
C	3.43307200	-1.97249400	-0.80761400
H	4.90102100	-0.70265100	0.01319800
H	5.47345500	-1.64486900	-1.38969900
O	2.88830000	-1.44230300	-1.82390300
O	2.69984400	-2.69764500	-0.06244900
F	1.17313300	2.24451000	1.71476900
Sb	1.60848100	1.87972500	-0.15037000
F	3.33865500	1.21303800	0.35848200
F	0.88749600	0.06892800	0.09770200
F	-0.20610400	2.45466400	-0.58475000
F	1.91310600	1.52858700	-2.01327000
F	2.24723900	3.67673200	-0.33067400

C	-2.53648000	3.97798500	0.88584800
H	-1.94544600	4.22216300	-0.01259100
H	-3.56578900	4.35950000	0.78845800
H	-2.06567700	4.45028400	1.76071400

TS2

Zero-point correction=	0.409390 (Hartree/Particle)
Thermal correction to Energy=	0.449494
Thermal correction to Enthalpy=	0.450439
Thermal correction to Gibbs Free Energy=	0.334528
Sum of electronic and zero-point Energies=	-3834.632857
Sum of electronic and thermal Energies=	-3834.592753
Sum of electronic and thermal Enthalpies=	-3834.591809
Sum of electronic and thermal Free Energies=	-3834.707719

Cartesian Coordinates

Atom	X	Y	Z
C	2.90354600	-1.95782300	0.21087700
C	2.45048000	-0.86475300	0.96563500
C	2.78031500	0.27463100	0.20298500
C	3.46147800	-0.14002000	-0.94873100
N	3.51822100	-1.52955500	-0.91940400
H	2.77965200	-3.02310600	0.39701800
C	2.51962700	1.70093700	0.43202900
H	2.55590800	1.97006000	1.49103800
C	4.00379100	0.78774200	-1.86651100
H	4.56755800	0.44458100	-2.73939900
C	3.15987900	2.63283900	-0.50687800
H	3.20494200	3.68817800	-0.23343200
C	3.82894600	2.15322400	-1.66083300
H	4.24902000	2.86602100	-2.37488800

C	1.77727100	-0.82194500	2.28686800
O	1.43219400	0.26212600	2.74829700
C	1.59810900	-2.08996000	3.03534000
H	1.86844400	-3.02941000	2.54432400
C	1.09249600	-2.09494900	4.28047800
H	0.82200800	-1.12058000	4.70854000
C	0.84933400	-3.30865600	5.11810200
H	1.15774900	-4.23669500	4.60728300
H	1.38987400	-3.24006600	6.08228400
H	-0.22544400	-3.39425100	5.37102800
C	1.19253600	2.29464000	-0.13903500
C	0.47858300	3.13015600	0.80841000
C	0.47766400	1.88157300	-1.33057800
O	-0.75837000	3.20792100	0.92006800
O	-0.71361900	2.18184000	-1.56816400
O	1.25469100	3.81123500	1.64902400
O	1.16247600	1.20077300	-2.22569100
C	0.47584900	0.76852300	-3.41507700
H	-0.44215800	0.22536200	-3.15427700
H	0.23852400	1.64255900	-4.04364900
H	1.18566000	0.10494600	-3.92794200
C	0.61746800	4.49263600	2.73829700
H	-0.06170400	5.27324600	2.36014800
H	0.04792900	3.77696500	3.35232400
H	1.43429100	4.94016300	3.32184900
Cu	-1.99786100	1.99042900	-0.07814400
H	-5.79319200	0.50191300	1.51134400
C	-5.31975200	0.19888000	0.56475800
C	-4.09465400	1.01479400	0.29449200
H	-4.99071100	-0.85355400	0.64180700
H	-6.02716200	0.27703800	-0.27613200

O	-3.62215400	1.12827300	-0.87829400
O	-3.44504600	1.57871800	1.23425300
F	-0.20320700	-2.66109400	0.81067700
Sb	-1.14050100	-1.91627100	-0.70285600
F	-2.82523800	-1.99457800	0.22729400
F	-0.87535800	-0.12336000	0.08547200
F	0.57854500	-1.72020900	-1.59844700
F	-1.96170600	-1.08455400	-2.23376800
F	-1.37447400	-3.66246500	-1.45574100
C	3.90700900	-2.38676800	-2.02588500
H	3.08672700	-2.45262500	-2.76207000
H	4.81600000	-1.99589900	-2.51088700
H	4.12372800	-3.39556300	-1.64245000

CP3

Zero-point correction=	0.410185 (Hartree/Particle)
Thermal correction to Energy=	0.450989
Thermal correction to Enthalpy=	0.451933
Thermal correction to Gibbs Free Energy=	0.332650
Sum of electronic and zero-point Energies=	-3834.652895
Sum of electronic and thermal Energies=	-3834.612090
Sum of electronic and thermal Enthalpies=	-3834.611146
Sum of electronic and thermal Free Energies=	-3834.730429

Cartesian Coordinates

Atom	X	Y	Z
C	-5.25275900	-1.28977800	-0.84369500
C	-4.58515700	-0.37980600	-0.02067000
C	-3.29781200	-0.95242100	0.23205600
C	-3.24294700	-2.17721700	-0.42752400
N	-4.44490900	-2.36551700	-1.08502100

H	-6.25083200	-1.24880900	-1.27612400
C	-2.19078000	-0.52943300	1.09799200
H	-2.47774100	0.04241500	1.98123400
C	-2.10855900	-3.06123100	-0.37024200
H	-2.11877200	-4.01941800	-0.89718900
C	-1.02198600	-1.44804300	1.15851100
H	-0.45270100	-1.51867700	2.08710600
C	-1.02670400	-2.69042900	0.36072200
H	-0.14811100	-3.33446100	0.44328800
C	-5.06529300	0.91218200	0.50671100
O	-4.35371900	1.58227400	1.24846800
C	-6.42899500	1.37034900	0.11478700
H	-7.02595300	0.74003400	-0.55370800
C	-6.92689000	2.53444300	0.56626200
H	-6.27873000	3.11992900	1.23212900
C	-8.27421900	3.09596900	0.24305300
H	-8.85142500	2.43924700	-0.43038000
H	-8.86460600	3.25458700	1.16641100
H	-8.18005200	4.09028200	-0.23527500
C	-0.79484800	0.00765000	0.54860300
C	-0.08276000	0.91030700	1.49528300
C	-0.50762800	0.22136900	-0.88870800
O	0.73417500	1.78905100	1.20421100
O	0.27177200	1.06539100	-1.34901300
O	-0.40675100	0.71503400	2.75789400
O	-1.17582000	-0.55595600	-1.70688800
C	-0.79934000	-0.53837300	-3.09227900
H	-0.97355700	0.45821700	-3.52709800
H	0.26399400	-0.80847500	-3.18261100
H	-1.43744200	-1.29133400	-3.57553800
C	0.35777900	1.40963600	3.75743500

H	1.41762500	1.12590600	3.66493700
H	0.24643700	2.49830700	3.63688900
H	-0.05551900	1.07885100	4.72002100
Cu	1.72838900	2.09273500	-0.48563900
H	5.37080600	3.99540100	-1.68673000
C	4.42282500	4.56416500	-1.69009200
C	3.32145800	3.65582400	-1.22740000
H	4.53166100	5.41835400	-1.00357300
H	4.23197300	4.90823400	-2.71870700
O	3.02582300	3.54947000	0.00299300
O	2.67124400	2.93561500	-2.04739600
F	1.88475000	-0.95342900	1.75498500
Sb	3.04057300	-1.50030700	0.28285400
F	3.13781500	0.41660100	-0.18248800
F	4.56159700	-1.32334900	1.42678500
F	2.73588100	-3.33871100	0.74688500
F	4.13970600	-1.93403000	-1.22058200
F	1.46538500	-1.56163200	-0.86203000
C	-4.79565900	-3.52688100	-1.88171900
H	-4.79715400	-4.44285800	-1.26509400
H	-5.80297400	-3.38426400	-2.30130200
H	-4.08342200	-3.66228800	-2.71422600

TS3

Zero-point correction=	0.408858 (Hartree/Particle)
Thermal correction to Energy=	0.449319
Thermal correction to Enthalpy=	0.450263
Thermal correction to Gibbs Free Energy=	0.331051
Sum of electronic and zero-point Energies=	-3834.639098
Sum of electronic and thermal Energies=	-3834.598637
Sum of electronic and thermal Enthalpies=	-3834.597693

Sum of electronic and thermal Free Energies= -3834.716905

Cartesian Coordinates

Atom	X	Y	Z
C	-5.46238300	-1.33921000	-0.80761200
C	-4.96751800	-0.32472600	-0.02201900
C	-3.61593300	-0.72506800	0.33009900
C	-3.38144400	-1.99337300	-0.28755600
N	-4.50702500	-2.34047100	-0.96220200
H	-6.43648000	-1.45484900	-1.27990800
C	-2.62608700	-0.18137400	1.12978300
H	-2.80424500	0.72698300	1.70655500
C	-2.15878600	-2.72266700	-0.16810300
H	-2.03719400	-3.70333800	-0.63361700
C	-1.28818100	-0.80629000	1.17559300
H	-0.93221400	-0.91220700	2.21609800
C	-1.13699300	-2.13195100	0.50656500
H	-0.16664100	-2.62238000	0.59846200
C	-5.63424100	0.92355700	0.42465000
O	-5.05729100	1.67210000	1.20182800
C	-6.99538400	1.21751800	-0.09562300
H	-7.45689600	0.51496600	-0.79846000
C	-7.65382400	2.32950500	0.27804200
H	-7.13528700	2.99540000	0.98085800
C	-9.01718100	2.73487200	-0.17817400
H	-9.45939400	2.00778600	-0.88047200
H	-9.69975300	2.84798500	0.68628400
H	-8.98354100	3.72477100	-0.67308800
C	-0.45333800	0.36369900	0.60877600
C	0.25285400	1.18238100	1.54423100
C	-0.28275700	0.57351900	-0.79034600

O	1.13407900	2.02976600	1.28372600
O	0.53638500	1.35355500	-1.32663300
O	-0.09546300	0.99502900	2.82352200
O	-1.08781000	-0.14577100	-1.57946400
C	-0.75513100	-0.19382900	-2.96981900
H	-0.83012800	0.80590400	-3.42693800
H	0.26649500	-0.58511000	-3.09560400
H	-1.48837500	-0.87719300	-3.42433900
C	0.71886100	1.61574800	3.82357600
H	1.75686400	1.25483800	3.74249200
H	0.70235600	2.71190200	3.71472800
H	0.28066200	1.31680700	4.78675800
Cu	2.09037300	2.16395400	-0.43353800
H	6.03615200	3.33474700	-1.64169700
C	5.21155600	4.07093800	-1.65514600
C	3.95619300	3.38993200	-1.18654900
H	5.47778000	4.89753300	-0.97778500
H	5.09140200	4.43295500	-2.68834400
O	3.64762300	3.35798400	0.04387900
O	3.18762800	2.79708100	-2.00452800
F	1.72254800	-1.28470500	1.68686500
Sb	2.92780000	-1.72429000	0.21991700
F	3.16935100	0.20659300	-0.05814700
F	4.39654300	-1.75605800	1.44588600
F	2.49739500	-3.57953700	0.49812700
F	4.07692300	-2.10280000	-1.26317700
F	1.40759500	-1.61518800	-0.99616600
C	-4.69161800	-3.56604100	-1.72667600
H	-4.59998900	-4.44983000	-1.07261300
H	-5.69560200	-3.55969800	-2.17573600
H	-3.94181400	-3.63607400	-2.53265600

CP4

Zero-point correction= 0.408824 (Hartree/Particle)

Thermal correction to Energy= 0.450100

Thermal correction to Enthalpy= 0.451045

Thermal correction to Gibbs Free Energy= 0.329125

Sum of electronic and zero-point Energies= -3834.642863

Sum of electronic and thermal Energies= -3834.601587

Sum of electronic and thermal Enthalpies= -3834.600642

Sum of electronic and thermal Free Energies= -3834.722562

Cartesian Coordinates

Atom	X	Y	Z
C	-5.34731600	-1.40067000	-0.40458900
C	-5.02030300	-0.15088700	0.05622600
C	-3.65897200	-0.26429000	0.56636900
C	-3.25504200	-1.63409600	0.36900500
N	-4.28683600	-2.28811200	-0.21577900
H	-6.26524100	-1.76293600	-0.86495000
C	-2.76140800	0.63444200	1.08419000
H	-3.03334900	1.68584700	1.19617000
C	-1.98451300	-2.14111400	0.74111300
H	-1.68590400	-3.17334800	0.54577800
C	-1.42331500	0.18323400	1.52783300
H	-1.43815400	0.32198200	2.63686300
C	-1.11606400	-1.26313100	1.32125400
H	-0.12331100	-1.59014700	1.63744900
C	-5.83748000	1.08870200	0.06480500
O	-5.37251000	2.11339600	0.54524100
C	-7.20299800	1.03358400	-0.51803000
H	-7.56408700	0.08678600	-0.93471200

C	-7.98945400	2.12500400	-0.54192500
H	-7.56958000	3.04376100	-0.11085800
C	-9.37269500	2.19508800	-1.10073100
H	-9.71084800	1.22813000	-1.51055000
H	-10.09105100	2.51739400	-0.32225800
H	-9.42966600	2.95579300	-1.90323200
C	-0.25892700	1.07330100	1.07507500
C	0.83903500	1.25448700	1.95622300
C	-0.12582300	1.39958400	-0.29916800
O	1.95427700	1.75856700	1.68426600
O	0.85884300	1.93327400	-0.86144300
O	0.62684500	0.82633900	3.21392800
O	-1.20381400	1.11423700	-1.04993200
C	-1.05269100	1.19742200	-2.46815000
H	-0.80804900	2.22593200	-2.77876000
H	-0.25750000	0.51111800	-2.80012000
H	-2.02436900	0.89410300	-2.88672700
C	1.76146200	0.76555100	4.08308300
H	2.52477600	0.09664400	3.65455600
H	2.19293500	1.76762000	4.23710400
H	1.38297300	0.36085300	5.03349400
Cu	2.67145500	2.02531900	-0.12237800
H	6.61315400	2.19268000	-1.80915600
C	6.09874100	3.12497900	-1.51200500
C	4.74646100	2.76878000	-0.95904700
H	6.71026400	3.62434500	-0.74404300
H	5.99517700	3.76039700	-2.40560100
O	4.59220000	2.50781500	0.27325700
O	3.73133600	2.68502600	-1.71512500
F	1.89079000	-1.43157000	1.47433100
Sb	2.32272600	-1.97022000	-0.35464600

F	3.13754700	-0.20296300	-0.57664700
F	3.97336900	-2.71044600	0.26634300
F	1.34339400	-3.60125700	-0.03517200
F	2.72867400	-2.44113100	-2.16492100
F	0.64446400	-1.18149700	-0.95458600
C	-4.28040100	-3.69189200	-0.60790700
H	-4.10125600	-4.33578100	0.26947200
H	-5.25799200	-3.94595000	-1.04299100
H	-3.49365400	-3.87941600	-1.35798800

CP5

Zero-point correction=	0.411288 (Hartree/Particle)
Thermal correction to Energy=	0.452168
Thermal correction to Enthalpy=	0.453112
Thermal correction to Gibbs Free Energy=	0.333065
Sum of electronic and zero-point Energies=	-3834.702829
Sum of electronic and thermal Energies=	-3834.661950
Sum of electronic and thermal Enthalpies=	-3834.661005
Sum of electronic and thermal Free Energies=	-3834.781053

Cartesian Coordinates

Atom	X	Y	Z
C	5.88866900	-1.24058200	0.00000000
C	4.76863500	-0.41961500	-0.00000700
C	3.62929800	-1.31482000	-0.00000400
C	4.14237000	-2.63918700	0.00000800
N	5.52730200	-2.56083600	0.00001200
H	6.94508700	-0.97533600	-0.00000100
C	2.23989000	-1.11748800	-0.00001100
H	1.86231700	-0.09534000	-0.00002000
C	3.30488800	-3.76132200	0.00001500

H	3.70296600	-4.77914100	0.00002500
C	1.39252300	-2.22954600	-0.00000600
H	-0.60507300	-3.04818700	-0.00001900
C	1.93012600	-3.53845000	0.00000900
H	1.25052400	-4.39556300	0.00001500
C	4.71639600	1.05318800	-0.00001800
O	3.63934700	1.64341400	-0.00003400
C	6.01115100	1.79443700	-0.00000900
H	6.94475100	1.22144600	0.00000500
C	6.05807000	3.13736200	-0.00001400
H	5.09715200	3.66880500	-0.00002600
C	7.30033900	3.96970100	-0.00000400
H	8.21633000	3.35419700	0.00000900
H	7.32700500	4.63461000	-0.88510900
H	7.32698500	4.63461800	0.88509600
C	-0.13359600	-2.05951600	-0.00000900
C	-0.59793500	-1.33317200	-1.25564800
C	-0.59794500	-1.33318900	1.25563500
O	-0.54240000	-0.11753800	-1.42006300
O	-0.54240200	-0.11755700	1.42006900
O	-1.00032500	-2.14845500	-2.19301000
O	-1.00035400	-2.14848300	2.19297900
C	-1.55333900	-1.56113700	3.39370500
H	-0.79293700	-0.94108700	3.89383700
H	-2.42852100	-0.95052500	3.12117700
H	-1.84162500	-2.41387100	4.02280800
C	-1.55329700	-1.56109700	-3.39373600
H	-2.42848900	-0.95049800	-3.12121300
H	-0.79289300	-0.94103100	-3.89384500
H	-1.84156400	-2.41382400	-4.02285700
Cu	-0.43407000	1.32090200	0.00001300

H	1.34202600	5.21673700	-0.90757600
C	1.52753700	4.62144300	0.00003200
C	0.68590800	3.38350600	0.00002800
H	2.58270900	4.29100800	0.00002200
H	1.34204000	5.21671900	0.90765600
O	0.33546600	2.81831300	-1.08291100
O	0.33549700	2.81828700	1.08296400
F	-2.87976800	-1.30992300	-0.00000300
Sb	-4.16017400	0.17797300	-0.00000400
F	-2.59674300	1.39230200	0.00002800
F	-4.04833600	0.15536900	-1.92532000
F	-5.61359400	-1.06430600	-0.00002900
F	-5.31003900	1.70207100	-0.00000800
F	-4.04838500	0.15533900	1.92531500
C	6.42713300	-3.69623200	0.00001600
H	6.27128800	-4.32157700	-0.89736900
H	7.46668700	-3.33474200	0.00003100
H	6.27126600	-4.32158500	0.89739200

TS4

Zero-point correction=	0.408552 (Hartree/Particle)
Thermal correction to Energy=	0.449059
Thermal correction to Enthalpy=	0.450003
Thermal correction to Gibbs Free Energy=	0.330349
Sum of electronic and zero-point Energies=	-3834.627519
Sum of electronic and thermal Energies=	-3834.587011
Sum of electronic and thermal Enthalpies=	-3834.586067
Sum of electronic and thermal Free Energies=	-3834.705722

Cartesian Coordinates

Atom	X	Y	Z

C	-4.83359200	-1.28905200	-0.96638900
C	-4.16377600	-0.60353600	0.03733700
C	-3.00192700	-1.40677500	0.36644300
C	-3.09196800	-2.59769900	-0.41065900
N	-4.20271800	-2.46459300	-1.24726600
H	-5.71532000	-0.99894600	-1.53634900
C	-1.96869300	-1.12776900	1.28181800
H	-2.15558600	-0.39491600	2.06876300
C	-2.44971400	-3.81310900	-0.10798800
H	-2.80612500	-4.73259400	-0.58164200
C	-0.93190800	-2.85611400	1.62983600
H	-0.53838800	-3.04189700	2.63186300
C	-1.56148100	-3.91752800	0.95533500
H	-1.40358200	-4.91524200	1.38061700
C	-4.46344400	0.72585600	0.59296700
O	-3.73478900	1.22663200	1.44642200
C	-5.67605700	1.43872600	0.09824100
H	-6.29999600	0.95801300	-0.66326500
C	-6.01244900	2.64922700	0.57590200
H	-5.34951800	3.07730400	1.33973600
C	-7.20109900	3.45547100	0.16051700
H	-7.80585100	2.94494700	-0.60867700
H	-7.85207500	3.67093200	1.03007300
H	-6.88758300	4.43953400	-0.23905000
C	-0.55132900	-1.54863600	1.03423700
C	0.38445200	-0.67333300	1.87476900
C	-0.07834800	-1.43773300	-0.42092600
O	0.52920600	0.52251700	1.63725400
O	-0.06840800	-0.35583700	-1.00695000
O	0.94338300	-1.26230200	2.89105700
O	0.28324700	-2.55018800	-0.98110900

C	0.83998500	-2.48686000	-2.31467600
H	0.09173400	-2.07299500	-3.00889100
H	1.74209900	-1.85636500	-2.30299300
H	1.08204300	-3.52681500	-2.57107600
C	1.88014000	-0.49006200	3.68392000
H	2.67465300	-0.10232900	3.02837100
H	1.34859000	0.34072700	4.17404700
H	2.27451800	-1.19733800	4.42536100
Cu	0.13468000	1.44910900	-0.11902600
H	-1.57293400	5.06775400	-1.88440800
C	-0.81838100	5.05148700	-1.08250900
C	-0.50024600	3.63913700	-0.69093000
H	0.10378800	5.52735300	-1.46363100
H	-1.16295200	5.62327800	-0.20655500
O	-0.07529700	3.35382500	0.47072600
O	-0.61082600	2.67982600	-1.51760400
F	5.21201100	-1.14274100	-0.09307700
Sb	3.80199900	0.12777600	-0.32838600
F	3.44271100	-0.51535100	-2.11253100
F	2.49837200	-1.18294700	0.32566900
F	3.97901600	0.74705800	1.49064000
F	2.27674600	1.36644000	-0.54376600
F	4.97263800	1.48898200	-0.97982700
C	-4.68175500	-3.48281800	-2.15875000
H	-5.09770700	-4.35084500	-1.61365400
H	-5.47276900	-3.05543400	-2.79409900
H	-3.86171700	-3.83474200	-2.80755800

CP6

Zero-point correction= 0.409427 (Hartree/Particle)

Thermal correction to Energy= 0.450370

Thermal correction to Enthalpy=	0.451315
Thermal correction to Gibbs Free Energy=	0.331117
Sum of electronic and zero-point Energies=	-3834.633035
Sum of electronic and thermal Energies=	-3834.592092
Sum of electronic and thermal Enthalpies=	-3834.591147
Sum of electronic and thermal Free Energies=	-3834.711345

Cartesian Coordinates

Atom	X	Y	Z
C	-5.06838700	-1.32898300	-0.78866400
C	-4.31940600	-0.55050100	0.06678700
C	-3.08541400	-1.30530200	0.32725300
C	-3.26104900	-2.60109300	-0.32938900
N	-4.45743500	-2.52179900	-1.05702200
H	-6.02226700	-1.10227900	-1.26464800
C	-1.97936700	-0.86301200	0.99579800
H	-2.07324300	0.07989500	1.53816900
C	-2.57079400	-3.76735300	-0.10113500
H	-2.95159700	-4.69028800	-0.54859300
C	-0.70608200	-2.94662400	1.38652300
H	-0.03832000	-3.23067000	2.20140600
C	-1.50805900	-3.90426000	0.84904700
H	-1.34723100	-4.92427100	1.21919200
C	-4.61245100	0.81231400	0.53508100
O	-3.80994500	1.42693500	1.23440700
C	-5.91640200	1.42376900	0.14554700
H	-6.61520500	0.84474300	-0.46820700
C	-6.24530000	2.66511400	0.54218900
H	-5.50732400	3.19521100	1.15891700
C	-7.51853900	3.37936500	0.21916700
H	-8.19606500	2.76860400	-0.40218500

H	-8.05525500	3.66252100	1.14547200
H	-7.31217500	4.32590200	-0.31728500
C	-0.60402000	-1.51681200	0.91451500
C	0.32054700	-0.65500200	1.80482000
C	-0.06158400	-1.34718900	-0.53601900
O	0.48728500	0.54440000	1.59855100
O	0.01378900	-0.24598500	-1.07924800
O	0.83918100	-1.26002900	2.83295800
O	0.23626400	-2.45168100	-1.14915100
C	0.81394500	-2.35649200	-2.47233100
H	0.10132100	-1.86492900	-3.15326100
H	1.75157900	-1.78246900	-2.41858000
H	0.99759100	-3.39527900	-2.77765800
C	1.75503900	-0.50811600	3.66744100
H	2.59274900	-0.14795100	3.05105800
H	1.22274500	0.33989700	4.12577600
H	2.09217800	-1.22100200	4.43148800
Cu	0.25837200	1.52571100	-0.15232500
H	-1.13283000	5.26358900	-1.94871600
C	-0.43749200	5.19148900	-1.09789100
C	-0.22132600	3.75681600	-0.71765400
H	0.53247800	5.62455100	-1.40403100
H	-0.81177800	5.76404500	-0.23469600
O	0.11180200	3.42809000	0.46269400
O	-0.32960200	2.82046900	-1.57004000
F	5.18307500	-1.34454300	-0.01946400
Sb	3.85853300	0.01463300	-0.25529900
F	3.50784400	-0.56010300	-2.06419100
F	2.46213600	-1.23127300	0.33297000
F	4.02628200	0.57775500	1.58283000
F	2.41567700	1.34703700	-0.46991400

F	5.12470500	1.31800100	-0.84325600
C	-5.03813200	-3.61349200	-1.80692300
H	-5.35084500	-4.44140300	-1.14211400
H	-5.92161800	-3.24707800	-2.35211400
H	-4.31280700	-4.00796100	-2.53968100

TS1*C5*

Zero-point correction= 0.405265 (Hartree/Particle)

Thermal correction to Energy= 0.447536

Thermal correction to Enthalpy= 0.448480

Thermal correction to Gibbs Free Energy= 0.324239

Sum of electronic and zero-point Energies= -3834.570314

Sum of electronic and thermal Energies= -3834.528043

Sum of electronic and thermal Enthalpies= -3834.527099

Sum of electronic and thermal Free Energies= -3834.651340

Cartesian Coordinates

Atom	X	Y	Z
C	-3.13585200	-1.89066500	1.22609800
C	-3.15592800	-0.50761500	1.10799500
C	-2.00082800	-0.03456500	1.84616000
C	-1.36862000	-1.18460500	2.39628300
N	-2.07615600	-2.29875900	1.98913300
H	-3.80453500	-2.63530800	0.79690700
C	-1.41291200	1.22437700	2.05112200
H	-1.87708300	2.12196100	1.63866600
C	-0.20497800	-1.11092100	3.17725300
H	0.26538600	-2.00881000	3.58509900
C	-0.22951900	1.30007500	2.81109800
H	0.21342400	2.27816100	3.00652100
C	0.35280200	0.14356200	3.38141400

H	1.27157600	0.24307300	3.96269800
C	-4.11341400	0.32470600	0.37015700
O	-3.93893600	1.53862900	0.26065000
C	-5.30580600	-0.34108800	-0.22908000
H	-5.38415400	-1.43198800	-0.17035300
C	-6.28642300	0.36757700	-0.81411600
H	-6.16608700	1.45909600	-0.83236300
C	-7.52500100	-0.19846400	-1.43274100
H	-7.55892800	-1.29956500	-1.36742800
H	-8.43091100	0.20814000	-0.94240800
H	-7.59767600	0.08862300	-2.49987700
C	1.67065600	1.91502200	0.49844600
C	2.95156500	1.40225800	1.03374700
C	1.49696300	3.39087900	0.51628700
O	3.05959700	1.20030000	2.22052900
O	0.98682100	3.97055400	1.44314800
O	3.86314600	1.25234400	0.09925600
O	1.93910000	3.91878900	-0.61257300
C	1.62050800	5.29859900	-0.86732100
H	2.10652000	5.54013000	-1.82224000
H	0.52779700	5.41131600	-0.94905500
H	2.00721600	5.93875900	-0.05861500
C	5.12824100	0.67393400	0.48210700
H	4.96095900	-0.35293500	0.83640800
H	5.73307400	0.67325900	-0.43445100
H	5.60310600	1.28614800	1.26557200
Cu	0.20814300	0.93152200	-0.43870000
Sb	1.67031000	-2.05658200	-0.99378400
F	1.82819100	-0.29244100	-1.82930100
F	3.10018100	-1.56940400	0.20542100
F	1.29358400	-3.60006500	0.08764900

F	2.89663600	-2.90555200	-2.18140200
F	0.47103900	-0.98607900	0.20984400
F	0.16724700	-2.44918600	-2.10386500
O	-1.38369000	0.55098400	-1.60665000
C	-1.51577000	1.79077000	-1.82732900
C	-2.64938700	2.33663500	-2.63547300
H	-2.96183100	1.60405600	-3.39623000
H	-3.49158800	2.50178800	-1.93846700
H	-2.37130700	3.29479800	-3.10176600
O	-0.65207900	2.56560600	-1.29215000
C	-1.63957200	-3.67155000	2.17964000
H	-0.69859400	-3.85154300	1.62904800
H	-1.48284200	-3.88104900	3.25177000
H	-2.41632000	-4.35184000	1.79862600

CP1*C5*

Zero-point correction= 0.407747 (Hartree/Particle)

Thermal correction to Energy= 0.449649

Thermal correction to Enthalpy= 0.450593

Thermal correction to Gibbs Free Energy= 0.326958

Sum of electronic and zero-point Energies= -3834.617987

Sum of electronic and thermal Energies= -3834.576085

Sum of electronic and thermal Enthalpies= -3834.575141

Sum of electronic and thermal Free Energies= -3834.698776

Cartesian Coordinates

Atom	X	Y	Z
C	-3.36036300	-2.19232900	0.89323500
C	-3.34732800	-0.82472100	0.82178700
C	-2.09650400	-0.41525000	1.45255800
C	-1.43241500	-1.62580600	1.87649600

N	-2.21346500	-2.66806900	1.53468000
H	-4.09786400	-2.90836000	0.53429700
C	-1.48881300	0.79230200	1.65010100
H	-1.96495000	1.72590100	1.34329300
C	-0.17800100	-1.63049600	2.54269900
H	0.29530400	-2.56367500	2.84896800
C	-0.14002100	0.86800100	2.27814900
H	-0.28761300	1.46475700	3.21240700
C	0.44580800	-0.43324500	2.71214600
H	1.42857600	-0.38959000	3.18429600
C	-4.35274100	0.09520900	0.23065100
O	-4.16601000	1.30249400	0.28079500
C	-5.55748300	-0.49815400	-0.40491600
H	-5.66527800	-1.58826000	-0.42348800
C	-6.50205300	0.28346800	-0.95868400
H	-6.33378900	1.36774200	-0.91103100
C	-7.74823600	-0.19264100	-1.63037000
H	-7.83287200	-1.29270100	-1.62928900
H	-8.64384200	0.22965600	-1.13484700
H	-7.78317900	0.16048800	-2.67920400
C	0.78881100	1.74155200	1.36144100
C	2.27180500	1.46623700	1.49347400
C	0.45080500	3.18760200	1.50182700
O	2.87626200	1.25048000	2.51504100
O	-0.57873600	3.60688100	1.99138900
O	2.84622500	1.48166700	0.27472500
O	1.41527800	3.98575700	1.01446900
C	1.10734600	5.37589100	0.92553900
H	2.02493200	5.86409400	0.56536000
H	0.28412400	5.53072100	0.20896900
H	0.81745000	5.77886900	1.91003200

C	4.25137700	1.20107800	0.19315400
H	4.46006500	0.23307800	0.67193500
H	4.47928500	1.15359300	-0.87925600
H	4.82287800	2.00276900	0.68938300
Cu	0.56291700	1.38562200	-0.65368000
Sb	1.81499700	-2.13577300	-0.81492500
F	2.88177400	-0.82443300	-1.72559100
F	2.63003800	-1.62195600	0.86224200
F	0.61975200	-3.29075700	0.19421000
F	3.07661400	-3.54420200	-1.08078300
F	0.49434800	-0.66500300	-0.44578400
F	0.83293100	-2.54687400	-2.40144500
O	0.31500300	1.47416700	-2.67573500
C	-0.13701200	2.64736300	-2.54894000
C	-0.54647500	3.46199500	-3.74823100
H	-0.66989600	2.81577100	-4.63042500
H	-1.47832800	4.01105300	-3.53467500
H	0.23965200	4.20946100	-3.95994000
O	-0.22086700	3.14446500	-1.38002400
C	-1.88928700	-4.07824000	1.73388900
H	-0.94568500	-4.30742300	1.21469600
H	-1.79331600	-4.29806500	2.81066300
H	-2.70253500	-4.68918800	1.31602500

TS1*C6*

Zero-point correction=	0.405727 (Hartree/Particle)
Thermal correction to Energy=	0.448012
Thermal correction to Enthalpy=	0.448956
Thermal correction to Gibbs Free Energy=	0.323862
Sum of electronic and zero-point Energies=	-3834.570498
Sum of electronic and thermal Energies=	-3834.528213

Sum of electronic and thermal Enthalpies= -3834.527269

Sum of electronic and thermal Free Energies= -3834.652364

Cartesian Coordinates

Atom	X	Y	Z
C	3.48132800	-2.07467800	-0.24326700
C	4.00200200	-0.89283800	-0.77800900
C	2.86346400	-0.13556800	-1.22357900
C	1.70523800	-0.91206900	-0.91584700
N	2.12518500	-2.09565100	-0.32208800
H	4.00528200	-2.92351200	0.19512900
C	2.70921000	1.10372800	-1.88320700
H	3.59652400	1.68352600	-2.14035100
C	0.41442300	-0.48204800	-1.21780100
H	-0.45608900	-1.12117200	-1.06297000
C	1.43041300	1.53908200	-2.19974200
H	1.29510900	2.48815900	-2.72370400
C	0.28259600	0.77608100	-1.85921100
H	-0.69981100	1.05364800	-2.24349300
C	5.41270600	-0.47605500	-0.88779900
O	5.70350200	0.61258300	-1.37208100
C	6.46300300	-1.41240900	-0.39289500
H	6.15908400	-2.37799900	0.02670100
C	7.76647700	-1.09057500	-0.46009500
H	8.00796700	-0.11073400	-0.89352900
C	8.90499900	-1.94439800	-0.00246500
H	8.56535000	-2.90664200	0.41779400
H	9.59764800	-2.15485100	-0.84043000
H	9.50469600	-1.41942300	0.76632800
C	-0.85411200	2.28084700	0.07591300
C	-0.04204000	3.46359300	0.45972800

C	-2.23368200	2.49707600	-0.41540600
O	-0.47591000	4.15297400	1.35316700
O	-3.01379300	2.83638800	0.44373200
O	1.11705200	3.61416800	-0.15117200
O	-2.48790700	2.29398400	-1.68848000
C	-3.88077900	2.29942900	-2.08112800
H	-4.29047500	3.31732900	-1.98209000
H	-3.88442700	1.97536400	-3.12992000
H	-4.44145600	1.59275700	-1.45196800
C	2.00051300	4.61246100	0.38607800
H	1.53031600	5.60814000	0.34554800
H	2.25199500	4.36170200	1.42885800
H	2.89847100	4.57960300	-0.24606000
Cu	-0.39619400	0.63511200	1.07214900
F	-2.21065100	-0.22085600	0.92363900
Sb	-3.39623700	-1.51783500	-0.07886400
F	-4.78631600	-0.19605500	0.02247700
F	-2.72883700	-0.61865200	-1.66063900
F	-1.82678000	-2.64933900	-0.15834200
F	-4.44697100	-2.73829900	-1.10187100
F	-3.88924000	-2.26388600	1.60614100
O	0.50447700	-0.71380800	2.28524800
C	1.35947700	0.16574700	2.61138600
O	1.25936000	1.31201700	2.07030600
C	2.46868500	-0.13116000	3.57893100
H	2.58409700	0.70643100	4.28641100
H	2.26954900	-1.06771700	4.12128700
H	3.41689300	-0.22593900	3.01918000
C	1.24445300	-3.15380300	0.15626400
H	0.60430500	-2.77511600	0.96834300
H	0.59771800	-3.51495900	-0.65921600

H	1.86333600	-3.98503000	0.52698300

CP1$_{C6}$

Zero-point correction=	0.407973 (Hartree/Particle)
Thermal correction to Energy=	0.449692
Thermal correction to Enthalpy=	0.450636
Thermal correction to Gibbs Free Energy=	0.327604
Sum of electronic and zero-point Energies=	-3834.625069
Sum of electronic and thermal Energies=	-3834.583351
Sum of electronic and thermal Enthalpies=	-3834.582406
Sum of electronic and thermal Free Energies=	-3834.705439

Cartesian Coordinates

Atom	X	Y	Z
C	3.95583400	-0.59127300	1.45896600
C	4.32162100	-0.16523400	0.16175300
C	3.11860400	0.25612700	-0.45726000
C	2.06915600	0.07050000	0.50883400
N	2.63922400	-0.45284000	1.67248200
H	4.59208800	-0.99613900	2.24643100
C	2.80254500	0.77385200	-1.75202500
H	3.60582900	0.92479400	-2.47345900
C	0.76641700	0.38132000	0.26940000
H	-0.01268400	0.26027500	1.02287500
C	1.49805400	1.05528800	-2.03811800
H	1.21818100	1.42736800	-3.02820400
C	0.37620200	0.81671600	-1.08514600
H	-0.07941500	-0.14534200	-1.46979700
C	5.67014600	-0.14999000	-0.45955700
O	5.81025300	0.27976300	-1.59648000
C	6.81613400	-0.66655700	0.33530400

H	6.63665000	-1.04417000	1.34812000
C	8.06094100	-0.68005700	-0.17519700
H	8.17759400	-0.29120500	-1.19568700
C	9.28684100	-1.17589300	0.51887700
H	9.07446900	-1.54525900	1.53660400
H	9.75702800	-1.99410900	-0.06026900
H	10.04689500	-0.37365700	0.58745200
C	-0.77140300	1.82842700	-1.09636200
C	-0.46814900	3.24478500	-0.80578500
C	-1.95572100	1.52767600	-1.90832800
O	-1.20172900	4.19485600	-0.97623300
O	-3.07889400	1.86242100	-1.48866300
O	0.77951300	3.36806100	-0.28230000
O	-1.78022000	0.78733200	-2.98236300
C	-2.93254200	0.13241800	-3.55609400
H	-3.70876500	0.87257000	-3.80237200
H	-2.56056900	-0.36355800	-4.46276100
H	-3.31884900	-0.61312100	-2.84297200
C	1.16878000	4.68322100	0.10523400
H	1.12788200	5.37431600	-0.75318200
H	0.50558100	5.06756600	0.89870900
H	2.20032300	4.59626600	0.47776900
Cu	-2.29955600	1.70984500	0.42452800
F	-2.26453500	-0.55791200	0.25246900
Sb	-1.94074600	-2.47746100	-0.09617300
F	-3.49327900	-2.47761400	-1.22912200
F	-0.88303900	-1.86585800	-1.61854100
F	-0.33485200	-2.27346800	0.98773300
F	-1.51382900	-4.29928700	-0.48058400
F	-2.95737500	-2.91878900	1.46094600
O	-3.56934300	1.91094800	2.01080700

C	-2.57880300	1.76739100	2.77739700
O	-1.41312400	1.66244800	2.26204300
C	-2.75782400	1.68022000	4.26994700
H	-1.88799300	2.11007300	4.79196000
H	-3.68530200	2.18808600	4.57590200
H	-2.83740400	0.61447400	4.55329500
C	1.87649500	-0.84139700	2.85469300
H	1.34273900	0.03509500	3.25909300
H	1.13738500	-1.61000900	2.57383700
H	2.56950100	-1.23495600	3.61300600

TS1$_{C7}$

Zero-point correction= 0.404978 (Hartree/Particle)
Thermal correction to Energy= 0.447349
Thermal correction to Enthalpy= 0.448293
Thermal correction to Gibbs Free Energy= 0.322267
Sum of electronic and zero-point Energies= -3834.568581
Sum of electronic and thermal Energies= -3834.526209
Sum of electronic and thermal Enthalpies= -3834.525265
Sum of electronic and thermal Free Energies= -3834.651292

Cartesian Coordinates

Atom	X	Y	Z
C	-2.67477100	-0.28591100	-1.93714100
C	-2.89986200	-1.37509200	-1.09956400
C	-1.67667200	-2.13455800	-1.11777400
C	-0.76976000	-1.44976000	-1.97275100
N	-1.42447000	-0.33784000	-2.47431000
H	-3.32843800	0.54901900	-2.18290200
C	-1.27232600	-3.32208100	-0.48162700
H	-1.97671200	-3.83967600	0.17148700

C	0.54433900	-1.90884800	-2.18111700
H	1.21591200	-1.43253000	-2.89519900
C	0.02419800	-3.80606100	-0.70459800
H	0.34833100	-4.73056800	-0.21872100
C	0.91289100	-3.12130600	-1.53999700
H	1.91868100	-3.50717400	-1.70883800
C	-4.12191200	-1.72119800	-0.34401800
O	-4.18316200	-2.77368800	0.28217800
C	-5.25628900	-0.75693800	-0.36888800
H	-5.13870900	0.18397200	-0.91708900
C	-6.40167200	-1.01744100	0.28510300
H	-6.45378200	-1.97239600	0.82506300
C	-7.59717800	-0.12231100	0.34532600
H	-7.45097500	0.81025100	-0.22605800
H	-8.49461700	-0.63826100	-0.04820700
H	-7.83115300	0.14529900	1.39411000
C	2.43962000	-0.53610700	-0.66362000
C	3.62924800	-1.43389000	-0.58023100
C	2.65223400	0.60758200	-1.60097700
O	3.85824800	-2.33748500	-1.35054300
O	2.25339900	0.63974200	-2.74203000
O	4.38970900	-1.08291800	0.44935700
O	3.37542800	1.54082800	-1.00008100
C	3.63707500	2.75534000	-1.72723400
H	4.30236900	3.34303900	-1.07988600
H	4.12679400	2.52621300	-2.68752300
H	2.68982700	3.28756200	-1.89545400
C	5.55318400	-1.88000200	0.70934200
H	5.25073600	-2.90081500	0.99409400
H	6.20226600	-1.92253800	-0.18042900
H	6.06888800	-1.38603400	1.54424500

Cu	1.14067200	-0.59273400	0.84440200
F	-0.38896500	0.51186600	0.02449500
Sb	-0.36726100	2.41343800	0.66607800
F	1.26090400	1.72823400	1.51433700
F	0.67605800	2.78447900	-0.92518900
F	-1.95491900	2.71758000	-0.36617200
F	-0.12823300	4.20270700	1.27722300
F	-1.39210400	1.91481700	2.19419600
C	0.99529900	-1.95936400	2.77809800
O	2.04419900	-1.96583900	2.05381900
C	0.91317000	-2.78083000	4.03195300
O	0.04036600	-1.21291900	2.41457200
H	1.62289200	-3.62171500	3.99591400
H	1.17136900	-2.13739200	4.89291100
H	-0.11693000	-3.14267600	4.17985200
C	-0.84472600	0.70564100	-3.30622100
H	-0.26199200	1.41223600	-2.69350100
H	-0.17995400	0.25837000	-4.06003200
H	-1.65941400	1.24450800	-3.81382500

CP1*C7*

Zero-point correction=	0.408465 (Hartree/Particle)
Thermal correction to Energy=	0.449954
Thermal correction to Enthalpy=	0.450899
Thermal correction to Gibbs Free Energy=	0.328582
Sum of electronic and zero-point Energies=	-3834.611395
Sum of electronic and thermal Energies=	-3834.569905
Sum of electronic and thermal Enthalpies=	-3834.568961
Sum of electronic and thermal Free Energies=	-3834.691277

Cartesian Coordinates

Atom	X	Y	Z

C	-2.94933300	1.24318800	-0.98586700
C	-4.01890600	0.42861500	-0.69943400
C	-3.50177900	-0.92293000	-0.79634600
C	-2.11316800	-0.82138800	-1.09348100
N	-1.81596400	0.48060600	-1.24883300
H	-2.87936100	2.32761500	-1.04898100
C	-4.11462100	-2.17107300	-0.78801700
H	-5.18926600	-2.22307500	-0.59571200
C	-1.23087000	-2.00607400	-1.28178800
H	-0.80522100	-1.92235900	-2.31281900
C	-3.36825600	-3.34542300	-1.07287800
H	-3.87908300	-4.31104400	-1.09765700
C	-2.01418900	-3.28042000	-1.27487300
H	-1.45181400	-4.19178800	-1.48685500
C	-5.42461800	0.79296700	-0.40156500
O	-6.26396000	-0.08746000	-0.25460300
C	-5.76413000	2.23546300	-0.29196500
H	-4.96719700	2.97627600	-0.41847700
C	-7.02326700	2.63359400	-0.03542400
H	-7.77531700	1.84225300	0.08504300
C	-7.48269300	4.04856300	0.09962000
H	-6.66090400	4.77208200	-0.03580400
H	-8.27425500	4.27365500	-0.64121000
H	-7.93787100	4.21691100	1.09487200
C	0.07182400	-2.10443900	-0.42381600
C	-0.05396600	-3.01144200	0.76336900
C	1.26566600	-2.25253000	-1.31742900
O	-0.94941000	-3.79908600	0.97917900
O	1.25230000	-1.89294200	-2.48281700
O	0.93589600	-2.78689100	1.65686300

O	2.32719700	-2.80003700	-0.72854700
C	3.57308800	-2.73657900	-1.43636900
H	4.26871800	-3.36809100	-0.86527500
H	3.45613500	-3.11399400	-2.46454800
H	3.93542200	-1.69594200	-1.45646900
C	0.94848000	-3.57471900	2.85072500
H	0.00711100	-3.44330600	3.40881900
H	1.07761800	-4.64074200	2.60165100
H	1.80234800	-3.20863400	3.43747500
Cu	0.64998000	-0.50567100	0.87530800
F	1.51074600	2.31118500	0.44984100
Sb	3.21146400	1.70918100	-0.27997200
F	2.45904000	-0.13291100	0.11203400
F	2.32368800	1.58222700	-1.98667200
F	3.83074700	3.47547300	-0.65148500
F	4.73571400	0.80735600	-1.01649600
F	3.97987800	1.68676700	1.46813200
C	-0.55123200	0.51590900	2.65903700
O	-1.15026900	-0.00980200	1.65545100
C	-1.34116600	1.22413400	3.72532400
O	0.70397100	0.44882600	2.70485100
H	-1.41955500	2.29266800	3.45273700
H	-2.35822900	0.80868400	3.80151900
H	-0.81778600	1.16080100	4.69226400
C	-0.54114300	1.06526600	-1.66384100
H	0.14219900	0.28277700	-2.01881600
H	-0.72287700	1.76782000	-2.49336600
H	-0.07159100	1.61356100	-0.83299900

TS1*C2*

Zero-point correction= 0.405298 (Hartree/Particle)

Thermal correction to Energy=	0.447167
Thermal correction to Enthalpy=	0.448111
Thermal correction to Gibbs Free Energy=	0.325148
Sum of electronic and zero-point Energies=	-3834.559295
Sum of electronic and thermal Energies=	-3834.517426
Sum of electronic and thermal Enthalpies=	-3834.516482
Sum of electronic and thermal Free Energies=	-3834.639445

Cartesian Coordinates

Atom	X	Y	Z
C	2.73758700	1.13594300	1.07193300
C	1.41467300	1.61247800	0.95328800
C	1.42659100	2.61922100	-0.05564200
C	2.77824100	2.72984700	-0.50013900
N	3.54845800	1.84053600	0.22354700
H	3.16125700	0.52928100	1.86856300
C	0.41366900	3.38258800	-0.67974700
H	-0.63376100	3.23614100	-0.40548200
C	3.14908100	3.62045400	-1.51663100
H	4.18228200	3.70066100	-1.86250600
C	0.78558000	4.27178500	-1.67551400
H	0.02279500	4.87451000	-2.17621300
C	2.13792300	4.39239100	-2.08354300
H	2.39105800	5.09719100	-2.88161500
C	0.24214400	1.13157400	1.71889900
O	-0.05371100	-0.08069800	1.69123500
C	-0.54634400	2.09280800	2.47757000
H	-0.13657800	3.10080500	2.60200000
C	-1.78264600	1.76888600	2.92564700
H	-2.16160100	0.76345100	2.70619700
C	-2.70830300	2.69044700	3.63682300

H	-2.27518300	3.68940500	3.81308900
H	-3.63909100	2.79581600	3.04614400
H	-3.01720000	2.25213800	4.60578700
C	2.21315400	-1.46113200	0.50309400
C	3.19433600	-1.72496800	-0.57884500
C	2.37559200	-2.07931900	1.82751400
O	3.80557100	-0.84929300	-1.14794900
O	2.08268800	-3.25288600	1.88100700
O	3.25465600	-3.02021400	-0.85745000
O	2.75955600	-1.33132400	2.85616100
C	2.74157000	-1.96804400	4.14783500
H	1.71999600	-2.30309900	4.38788900
H	3.42495500	-2.83200300	4.15869600
H	3.07484600	-1.20026700	4.85938200
C	3.93281800	-3.39790200	-2.06395500
H	3.42366200	-2.94746500	-2.93118200
H	4.98489900	-3.07088700	-2.03693700
H	3.86645300	-4.49364700	-2.10710300
Cu	0.34654000	-1.02200800	-0.08368200
O	0.05092000	0.35941200	-1.78146800
C	0.19962300	-0.65913500	-2.48473000
O	0.55962600	-1.76438400	-1.92059000
C	-0.09155000	-0.66243200	-3.96089100
H	-1.18434400	-0.78458900	-4.06962400
H	0.18912300	0.30691700	-4.40313300
H	0.41700600	-1.49275200	-4.47513200
F	-2.92558300	-0.43533800	-2.20075300
Sb	-3.32968400	-0.43680500	-0.31994700
F	-4.27586900	-2.08989100	-0.48954300
F	-1.65410100	-1.50356300	-0.05497000
F	-2.25251900	1.15580900	-0.06278700

F	-3.56388600	-0.48468300	1.59976500
F	-4.90501600	0.62389600	-0.55795000
C	4.98190600	1.67849300	0.08021400
H	5.22570200	1.28174300	-0.91857900
H	5.49437600	2.64411000	0.23664800
H	5.33879400	0.95919800	0.83185400

CP1*C2*

Zero-point correction= 0.408551 (Hartree/Particle)
Thermal correction to Energy= 0.449602
Thermal correction to Enthalpy= 0.450546
Thermal correction to Gibbs Free Energy= 0.332355
Sum of electronic and zero-point Energies= -3834.604116
Sum of electronic and thermal Energies= -3834.563065
Sum of electronic and thermal Enthalpies= -3834.562121
Sum of electronic and thermal Free Energies= -3834.680312

Cartesian Coordinates

Atom	X	Y	Z
C	2.50759600	1.03763500	0.58497500
C	1.13506000	1.65862500	0.46423500
C	1.05465400	2.35691200	-0.71242900
C	2.36651200	2.27904800	-1.34237300
N	3.19343200	1.55036600	-0.60402400
H	3.00642800	1.49032600	1.46436200
C	-0.02216200	3.03380900	-1.37847500
H	-1.02949200	2.98669500	-0.96258400
C	2.62730400	2.97175800	-2.55666900
H	3.61004000	2.93839700	-3.02967000
C	0.25271500	3.67219400	-2.55579700
H	-0.54215100	4.19020300	-3.09858600

C	1.57490500	3.64755000	-3.12799700
H	1.73661400	4.17215100	-4.07520500
C	0.05339200	1.39157400	1.45182300
O	-0.16088600	0.21001900	1.76629600
C	-0.70799700	2.50098300	2.00673000
H	-0.33401900	3.51718500	1.84187900
C	-1.89364500	2.26835200	2.61845400
H	-2.23956800	1.22893300	2.67523400
C	-2.80739800	3.31208000	3.15378400
H	-2.41045500	4.33496100	3.04180600
H	-3.78470500	3.24091400	2.63802400
H	-3.01932200	3.12127500	4.22391900
C	2.48746500	-0.50578300	0.81902000
C	3.21090200	-1.36155200	-0.16646700
C	2.50818800	-0.89551500	2.26046100
O	3.57080500	-0.99480800	-1.27051200
O	2.42057100	-2.00949200	2.71553400
O	3.41661700	-2.60305600	0.28102200
O	2.58327500	0.20072300	3.07936200
C	2.46818600	-0.06139200	4.47695400
H	1.50802800	-0.55641300	4.69908900
H	3.28854700	-0.71145900	4.82316800
H	2.51955000	0.91876800	4.97464500
C	3.91906100	-3.54749800	-0.66038800
H	3.18991500	-3.68980300	-1.47446500
H	4.88166500	-3.21353900	-1.08340300
H	4.04999700	-4.48362400	-0.09813500
Cu	0.44785500	-1.14643100	0.37728200
O	0.46745400	-0.18249100	-1.83388400
C	0.57139300	-1.39030500	-2.11234100
O	0.74810800	-2.27587300	-1.19000800

C	0.43295800	-1.88855700	-3.53233200
H	-0.64797100	-2.01028700	-3.72753300
H	0.82885900	-1.14045100	-4.23748900
H	0.93040000	-2.86053900	-3.67380600
F	-2.62119500	-1.11367700	-2.08973300
Sb	-3.16264200	-0.62981600	-0.31182600
F	-4.17482200	-2.23850300	-0.11807800
F	-1.55715400	-1.61677800	0.35671000
F	-2.03538500	0.95769800	-0.39856900
F	-3.53012500	-0.14582500	1.52443700
F	-4.66018600	0.38268100	-0.94128800
C	4.63504400	1.49347600	-0.79309500
H	4.86933800	1.05787500	-1.77469600
H	5.06637300	2.50809800	-0.70454700
H	5.07229400	0.84399700	-0.02412800

9. Copy of ^{1}H-NMR and ^{13}C-NMR Spectra

3a

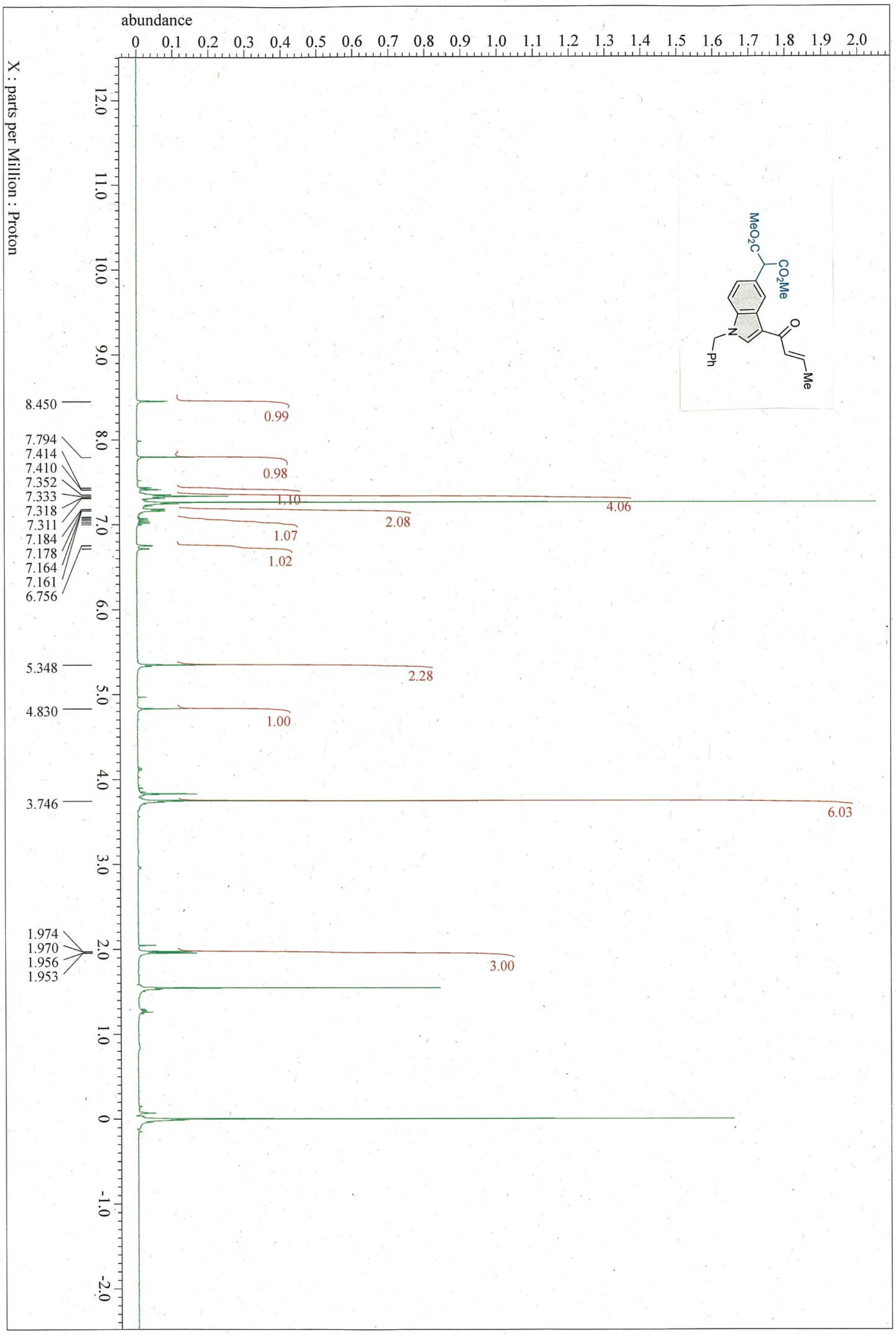

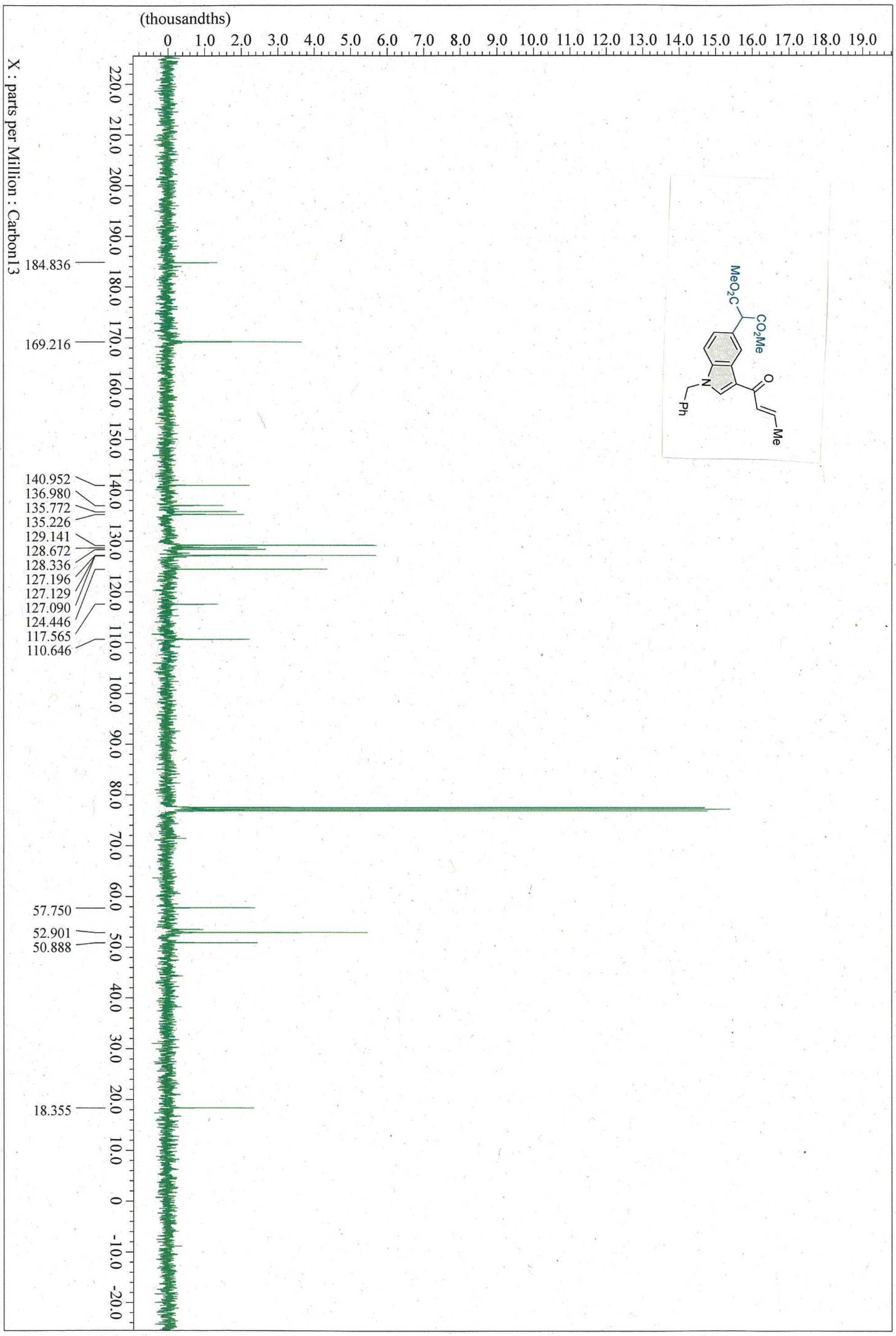
(thousandths)
X : parts per Million : Carbon13
184.836
169.216
140.952
136.980
135.772
135.226
129.141
128.672
128.336
127.196
127.129
127.090
124.446
117.565
110.646
57.750
52.901
50.888
18.355
MeO2C
CO2Me
O
N
Ph
Me

3b

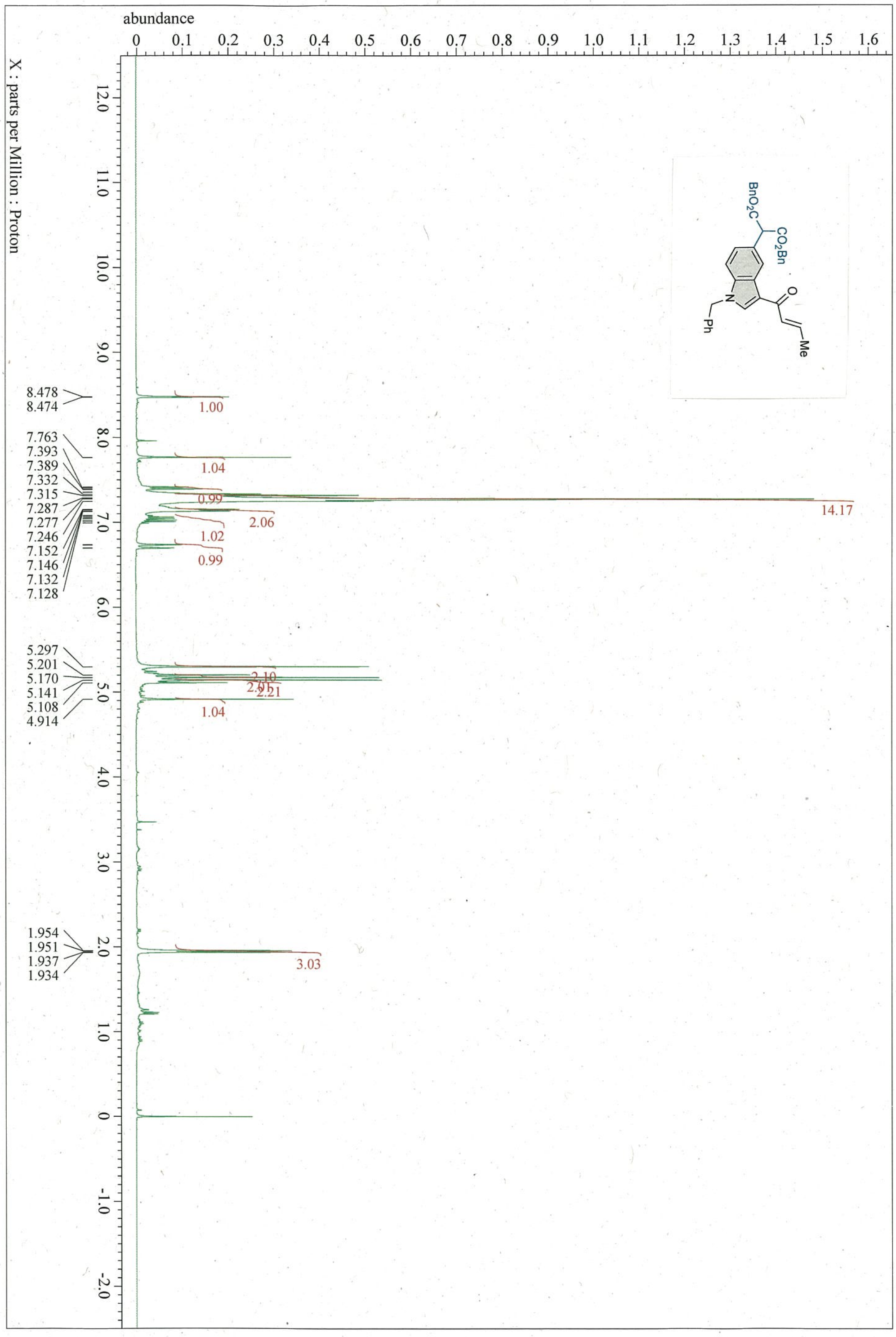
abundance
X : parts per Million : Proton
8.478
8.474
7.763
7.393
7.389
7.332
7.315
7.287
7.277
7.246
7.152
7.146
7.132
7.128
5.297
5.201
5.170
5.141
5.108
4.914
1.954
1.951
1.937
1.934
1.00
1.04
0.99
2.06
14.17
1.02
0.99
1.04
3.03
BnO2C
CO2Bn
O
N
Ph
Me

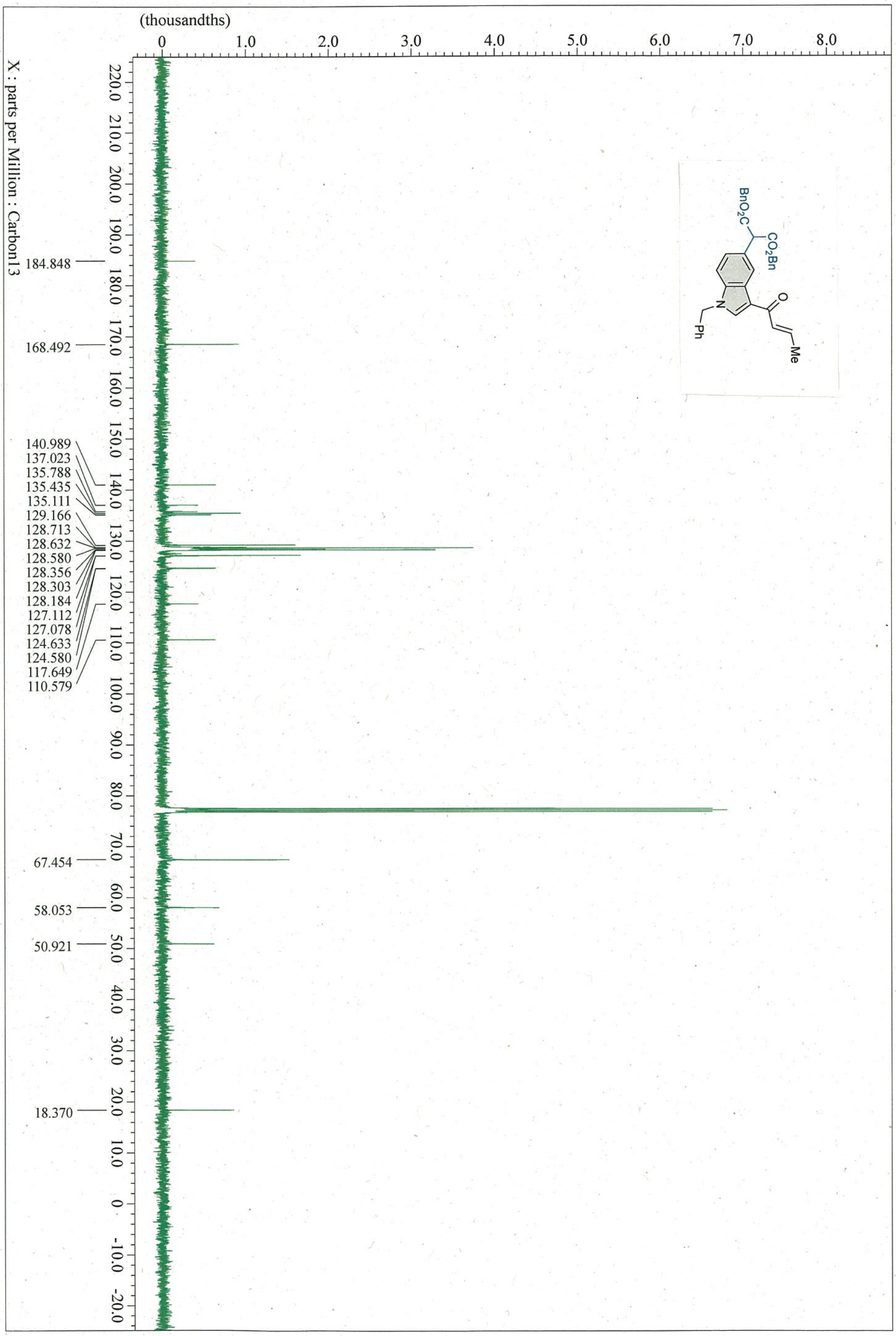
(thousandths)
0 1.0 2.0 3.0 4.0 5.0 6.0 7.0 8.0
X : parts per Million : Carbon13
220.0 210.0 200.0 190.0 180.0 170.0 160.0 150.0 140.0 130.0 120.0 110.0 100.0 90.0 80.0 70.0 60.0 50.0 40.0 30.0 20.0 10.0 0 -10.0 -20.0
184.848
168.492
140.989
137.023
135.788
135.435
135.111
129.166
128.713
128.632
128.580
128.356
128.303
128.184
127.112
127.078
124.633
124.580
117.649
110.579
67.454
58.053
50.921
18.370
BnO2C
CO2Bn
O
N
Ph
Me

3c

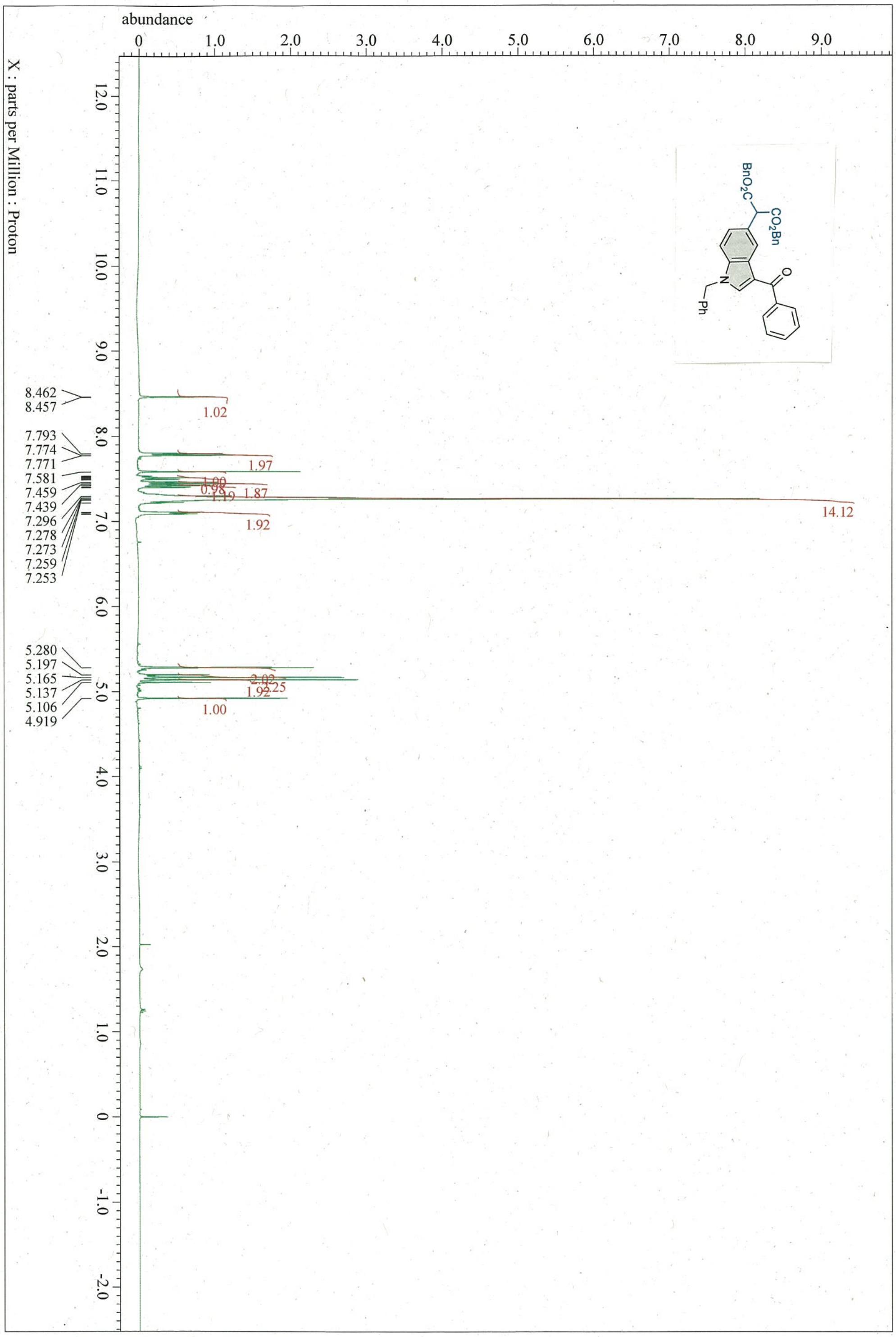
X : parts per Million : Proton
abundance
8.462
8.457
7.793
7.774
7.771
7.581
7.459
7.439
7.296
7.278
7.273
7.259
7.253
5.280
5.197
5.165
5.137
5.106
4.919
1.02
1.97
1.87
14.12
1.92
1.00

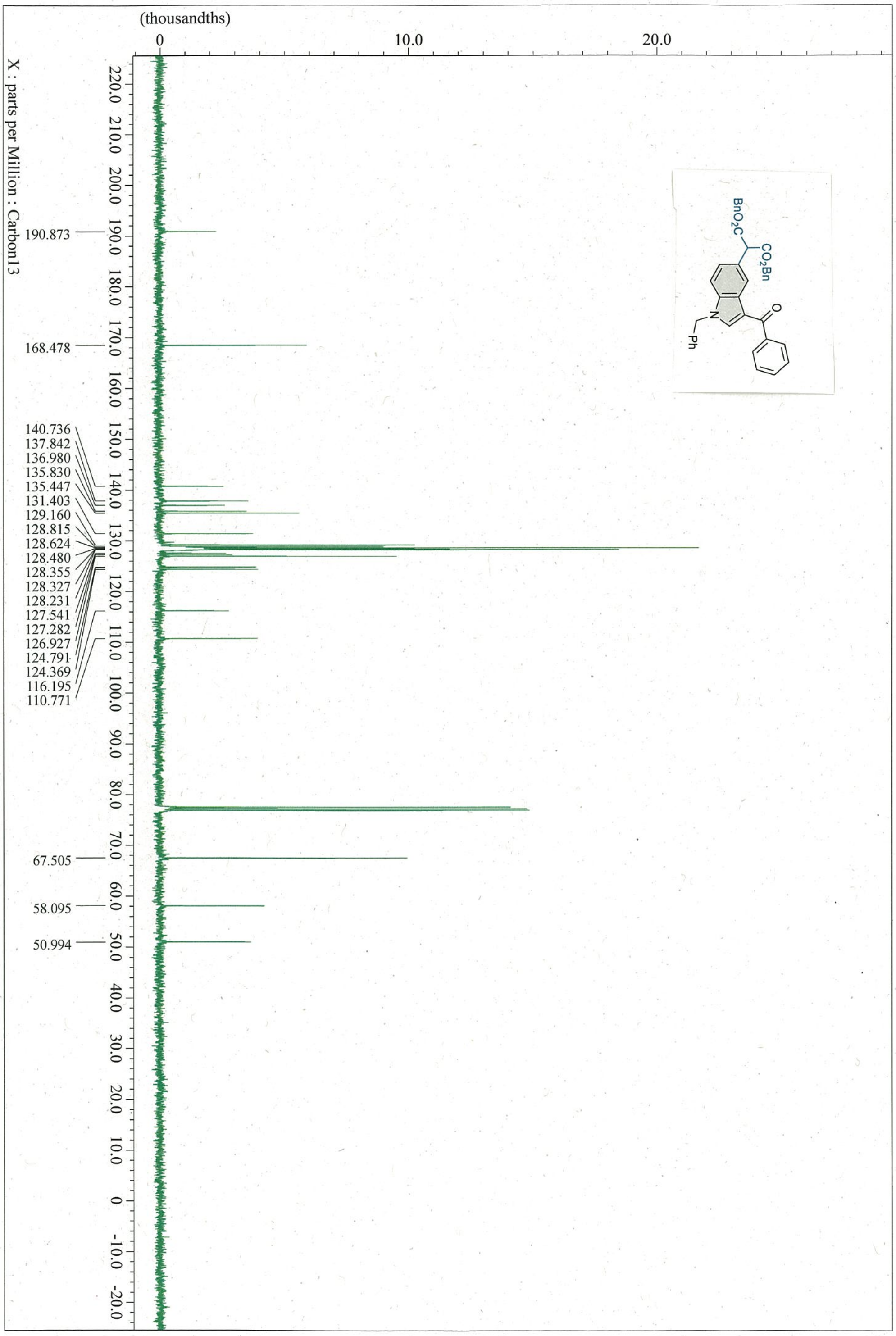
(thousandths)
0
10.0
20.0
X : parts per Million : Carbon13
190.873
168.478
140.736
137.842
136.980
135.830
135.447
131.403
129.160
128.815
128.624
128.480
128.355
128.327
128.231
127.541
127.282
126.927
124.791
124.369
116.195
110.771
67.505
58.095
50.994
BnO2C
CO2Bn
N
O
Ph

3d

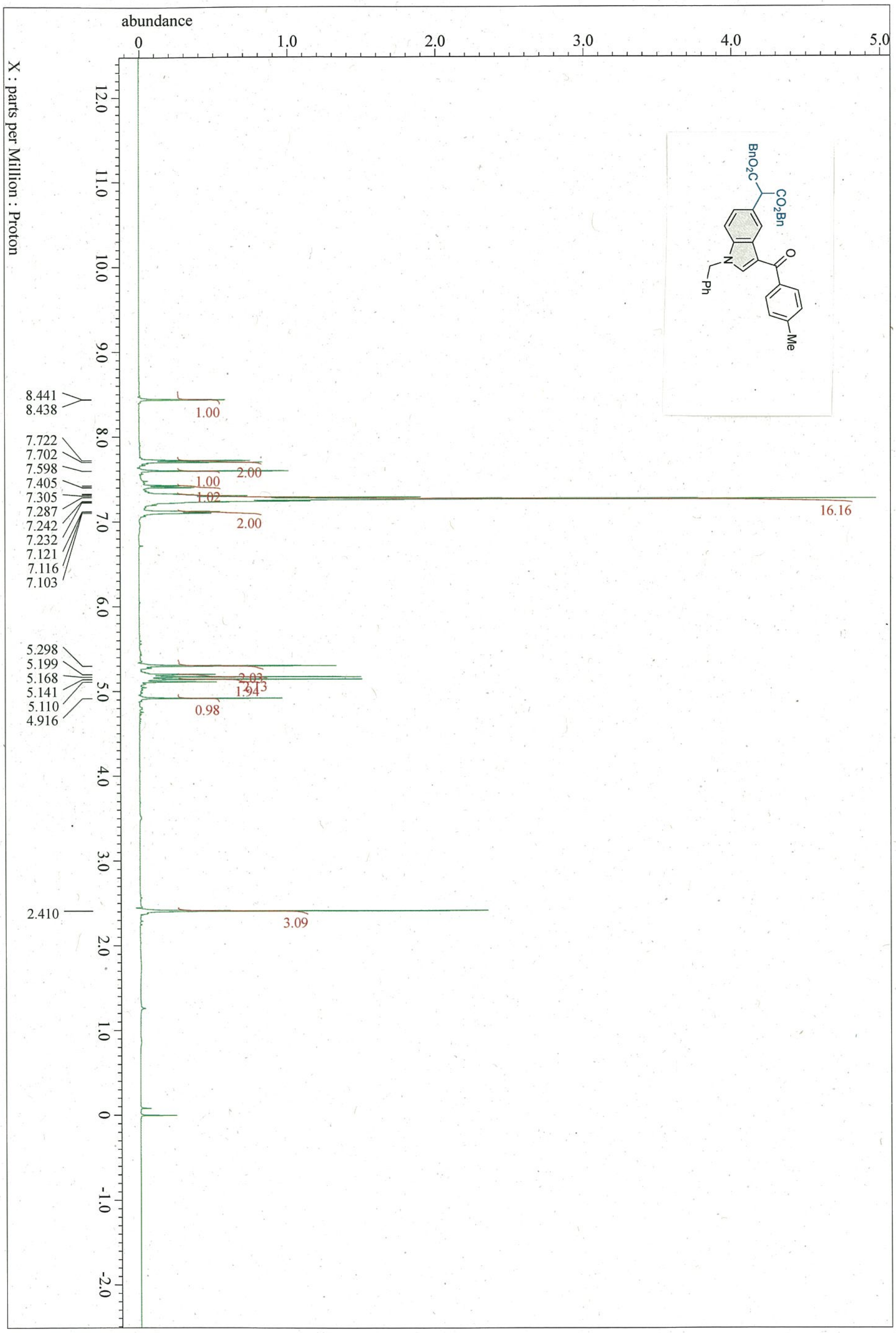
abundance
X : parts per Million : Proton
8.441
8.438
7.722
7.702
7.598
7.405
7.305
7.287
7.242
7.232
7.121
7.116
7.103
5.298
5.199
5.168
5.141
5.110
4.916
2.410
1.00
2.00
1.00
1.02
16.16
2.00
2.03
1.94
0.98
3.09
BnO_2C
CO_2Bn
N
Ph
O
Me

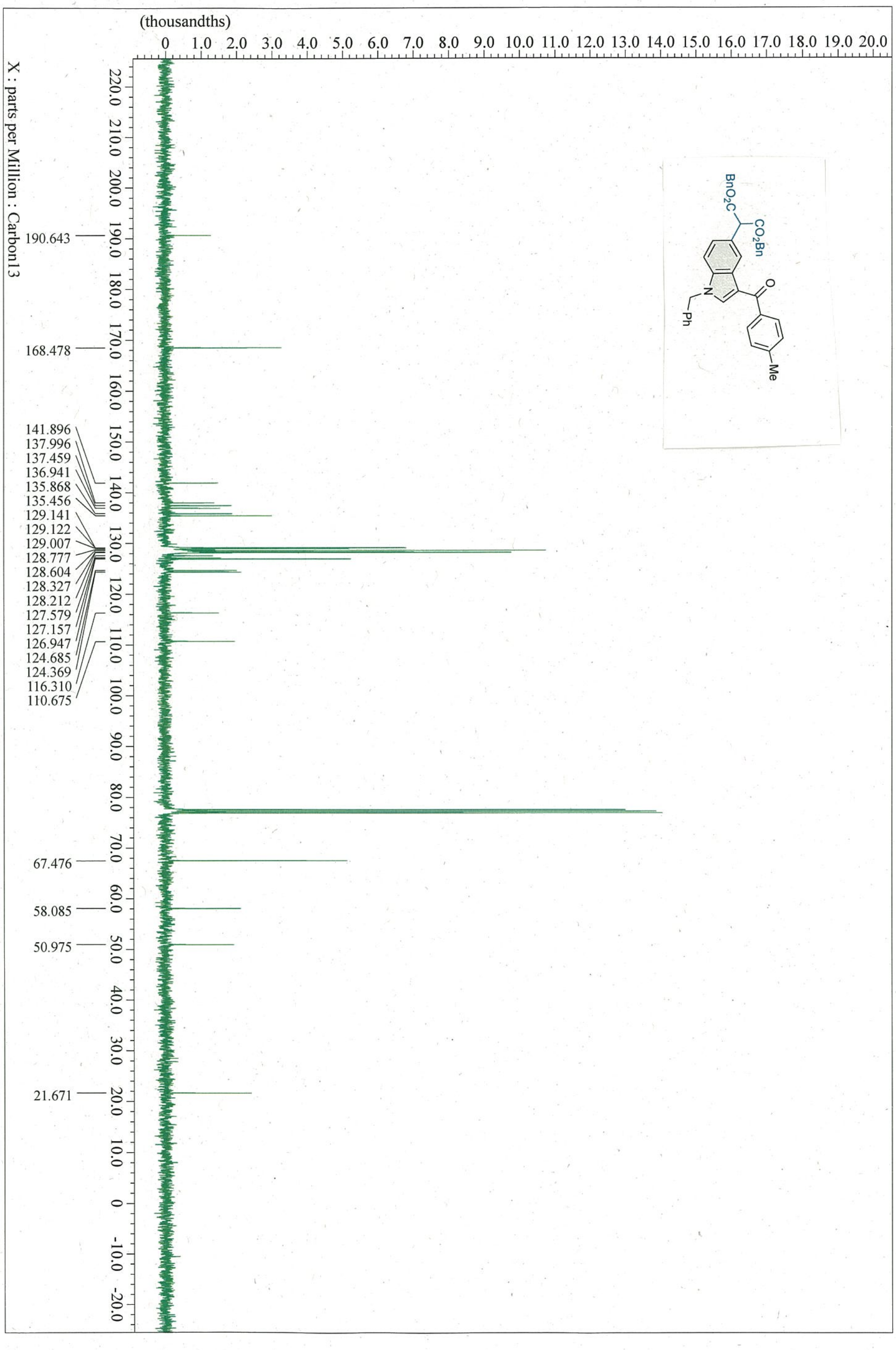

X : parts per Million : Carbon13
(thousandths)
190.643
168.478
141.896
137.996
137.459
136.941
135.868
135.456
129.141
129.122
129.007
128.777
128.604
128.327
128.212
127.579
127.157
126.947
124.685
124.369
116.310
110.675
67.476
58.085
50.975
21.671
BnO2C
CO2Bn
O
N
Ph
Me

3e

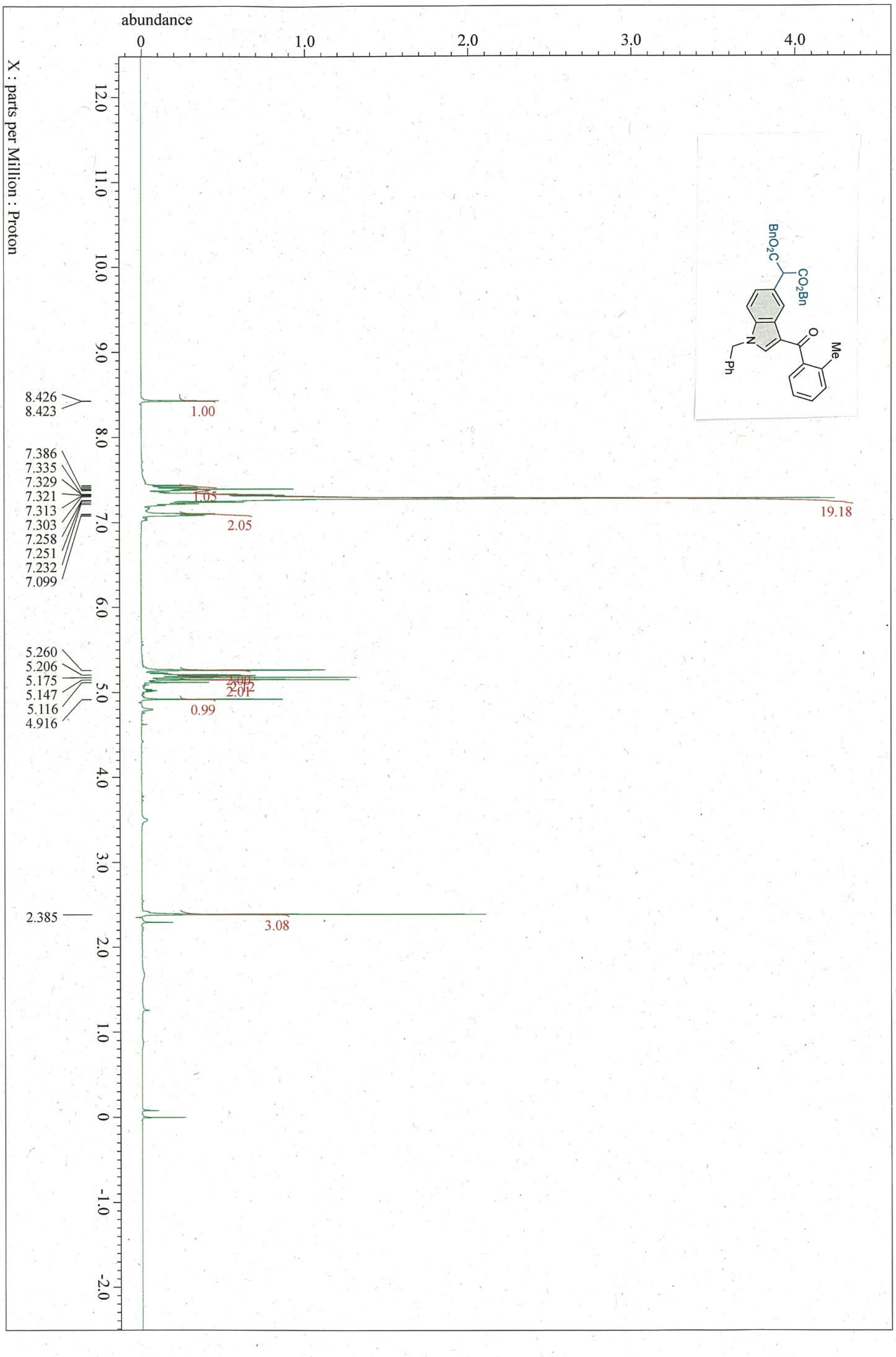

abundance
X : parts per Million : Proton
8.426
8.423
7.386
7.335
7.329
7.321
7.313
7.303
7.258
7.251
7.232
7.099
5.260
5.206
5.175
5.147
5.116
4.916
2.385
1.00
1.05
19.18
2.05
2.00
2.01
0.99
3.08
BnO2C
CO2Bn
Me
Ph
N
O

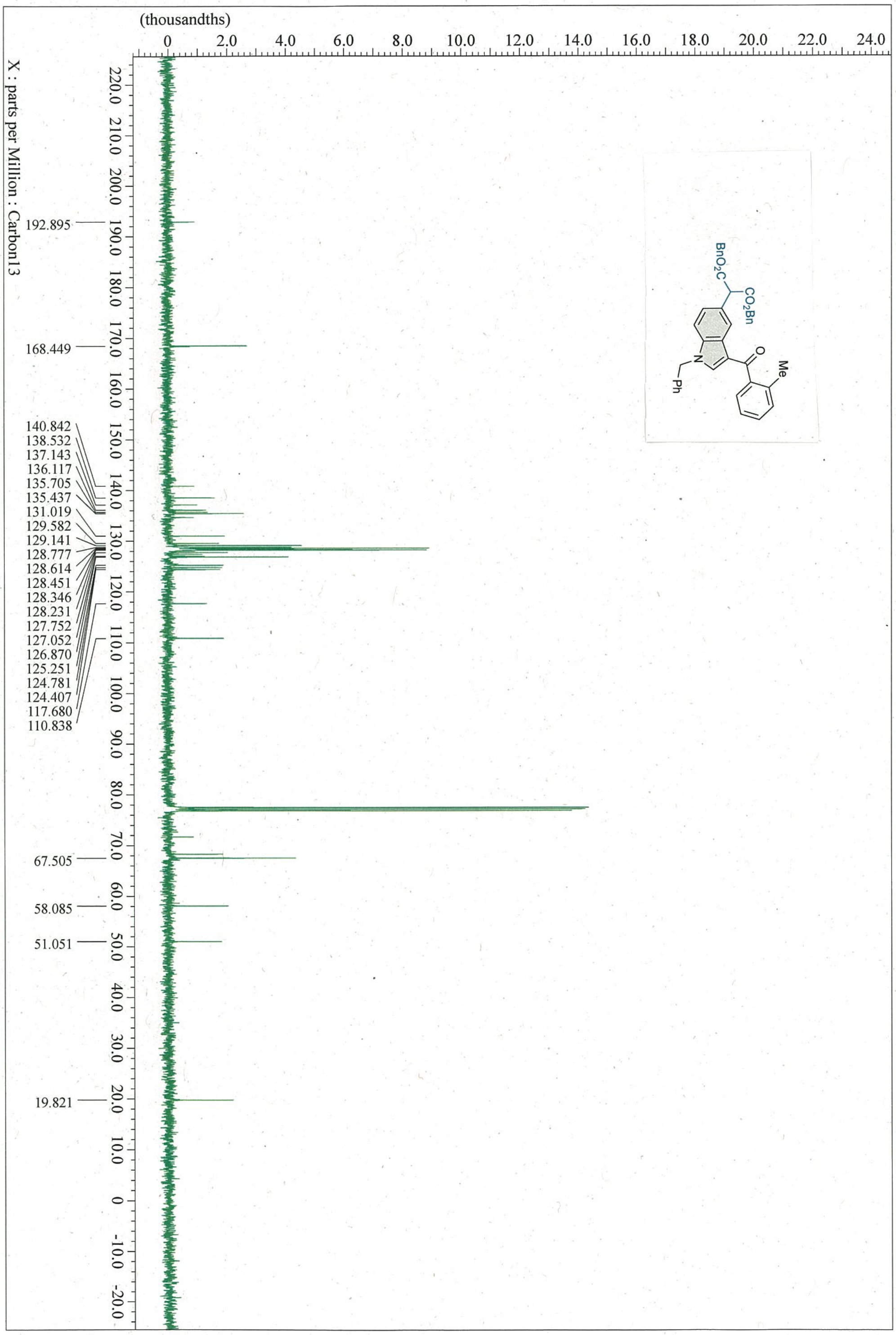
(thousandths)
X : parts per Million : Carbon13
192.895
168.449
140.842
138.532
137.143
136.117
135.705
135.437
131.019
129.582
129.141
128.777
128.614
128.451
128.346
128.231
127.752
127.052
126.870
125.251
124.781
124.407
117.680
110.838
67.505
58.085
51.051
19.821
BnO2C
CO2Bn
Me
Ph

3f

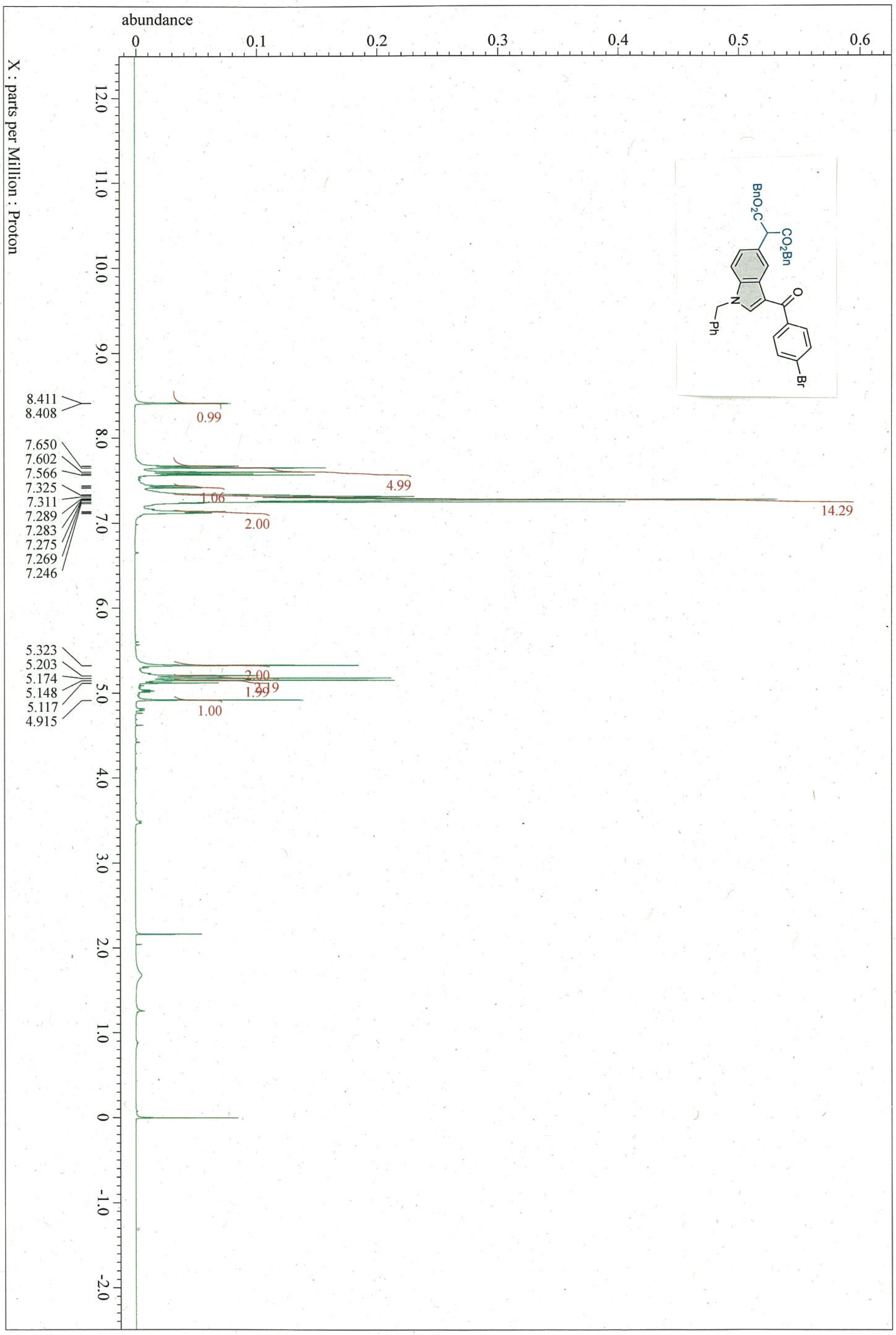
abundance
X : parts per Million : Proton
8.411
8.408
7.650
7.602
7.566
7.325
7.311
7.289
7.283
7.275
7.269
7.246
5.323
5.203
5.174
5.148
5.117
4.915
0.99
4.99
1.06
14.29
2.00
2.00
1.99
1.00
BnO2C
CO2Bn
N
Ph
O
Br

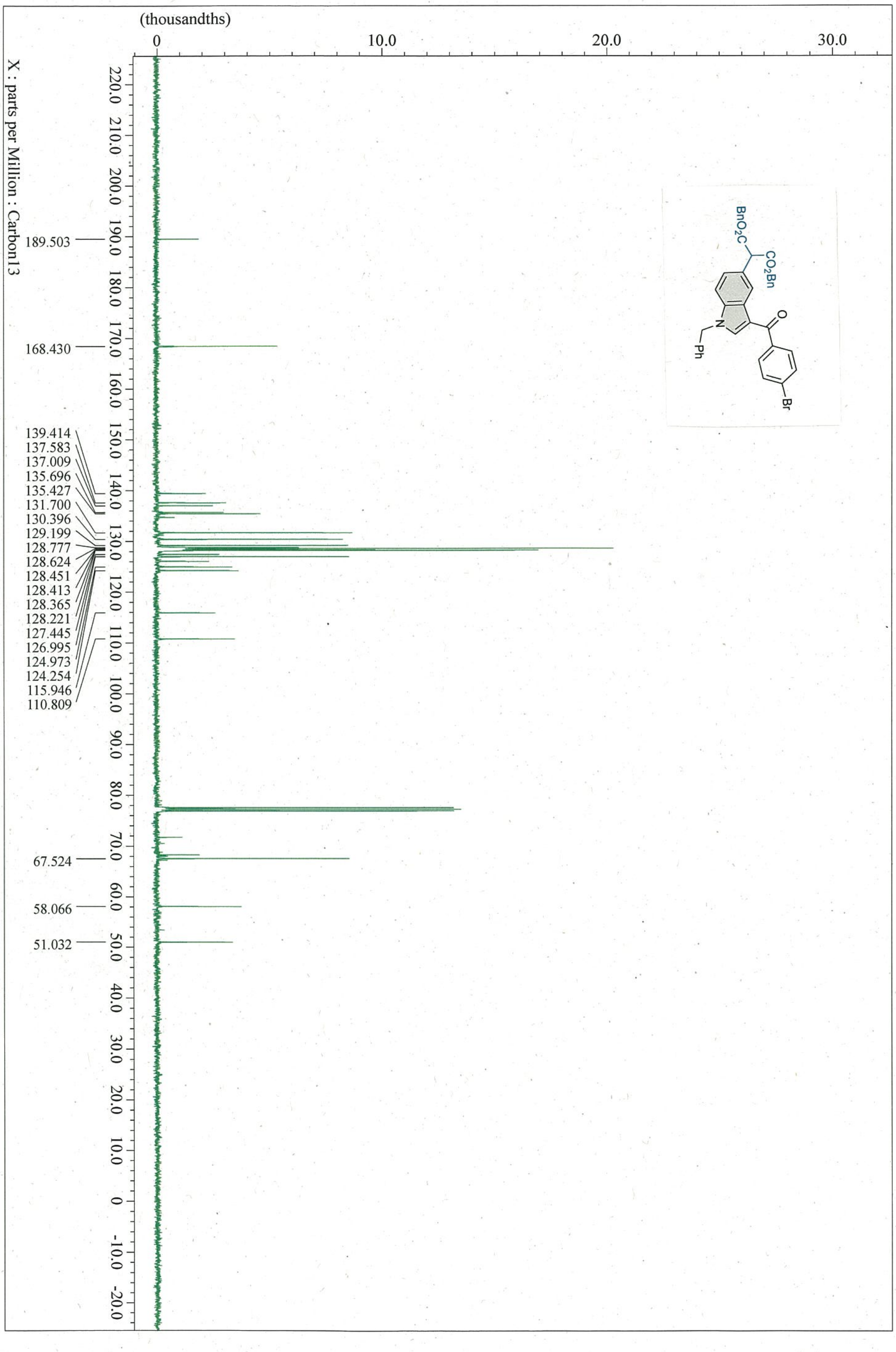
(thousandths)
0
10.0
20.0
30.0
X : parts per Million : Carbon13
220.0 210.0 200.0 190.0 180.0 170.0 160.0 150.0 140.0 130.0 120.0 110.0 100.0 90.0 80.0 70.0 60.0 50.0 40.0 30.0 20.0 10.0 0 -10.0 -20.0
189.503
168.430
139.414
137.583
137.009
135.696
135.427
131.700
130.396
129.199
128.777
128.624
128.451
128.413
128.365
128.221
127.445
126.995
124.973
124.254
115.946
110.809
67.524
58.066
51.032
BnO2C
CO2Bn
N
Ph
O
Br

3g

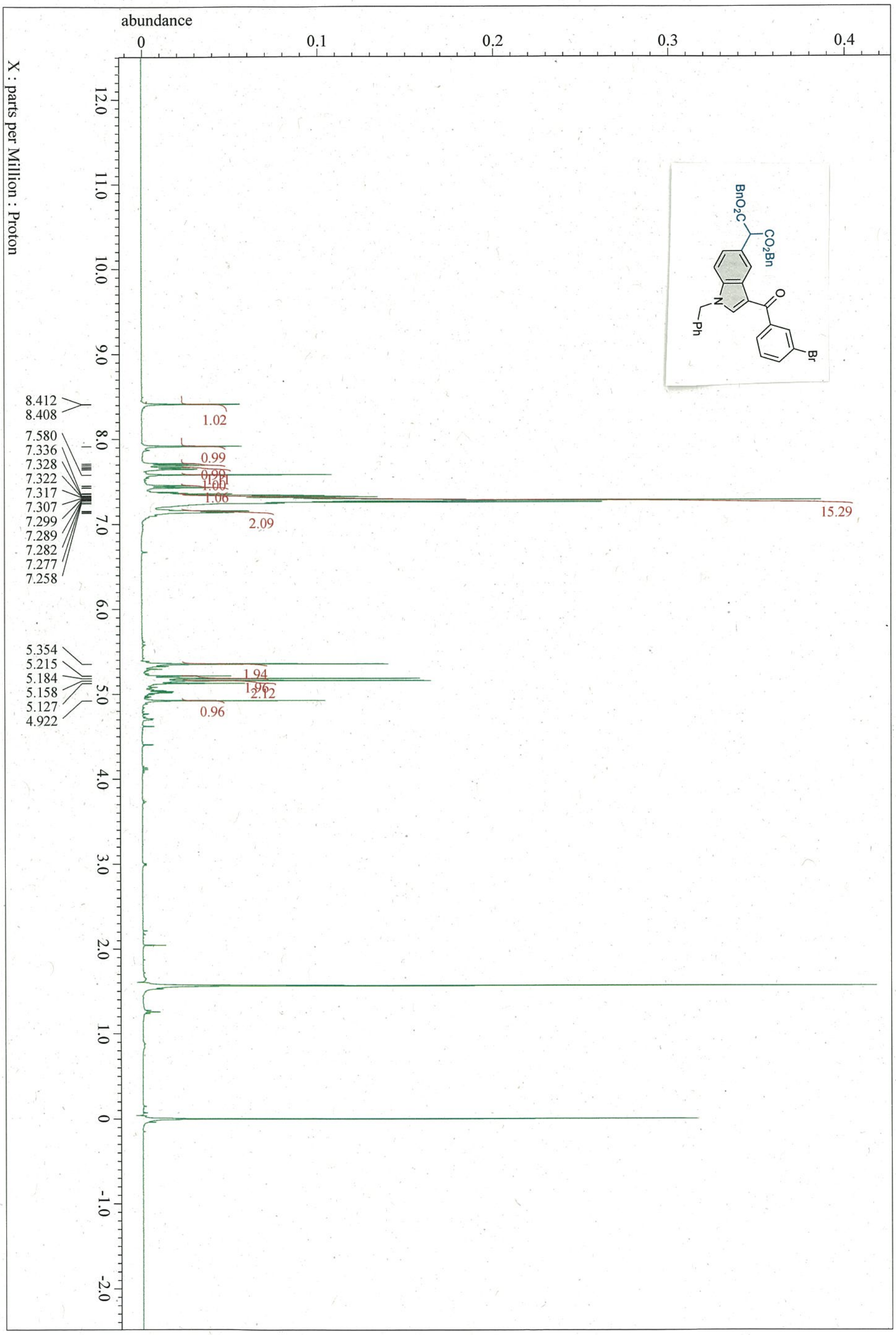

abundance
0
0.1
0.2
0.3
0.4
X : parts per Million : Proton
12.0
11.0
10.0
9.0
8.0
7.0
6.0
5.0
4.0
3.0
2.0
1.0
0
-1.0
-2.0
8.412
8.408
7.580
7.336
7.328
7.322
7.317
7.307
7.299
7.289
7.282
7.277
7.258
5.354
5.215
5.184
5.158
5.127
4.922
1.02
0.99
1.06
2.09
15.29
1.94
2.12
0.96
BnO_2C
CO_2Bn
O
N
Ph
Br

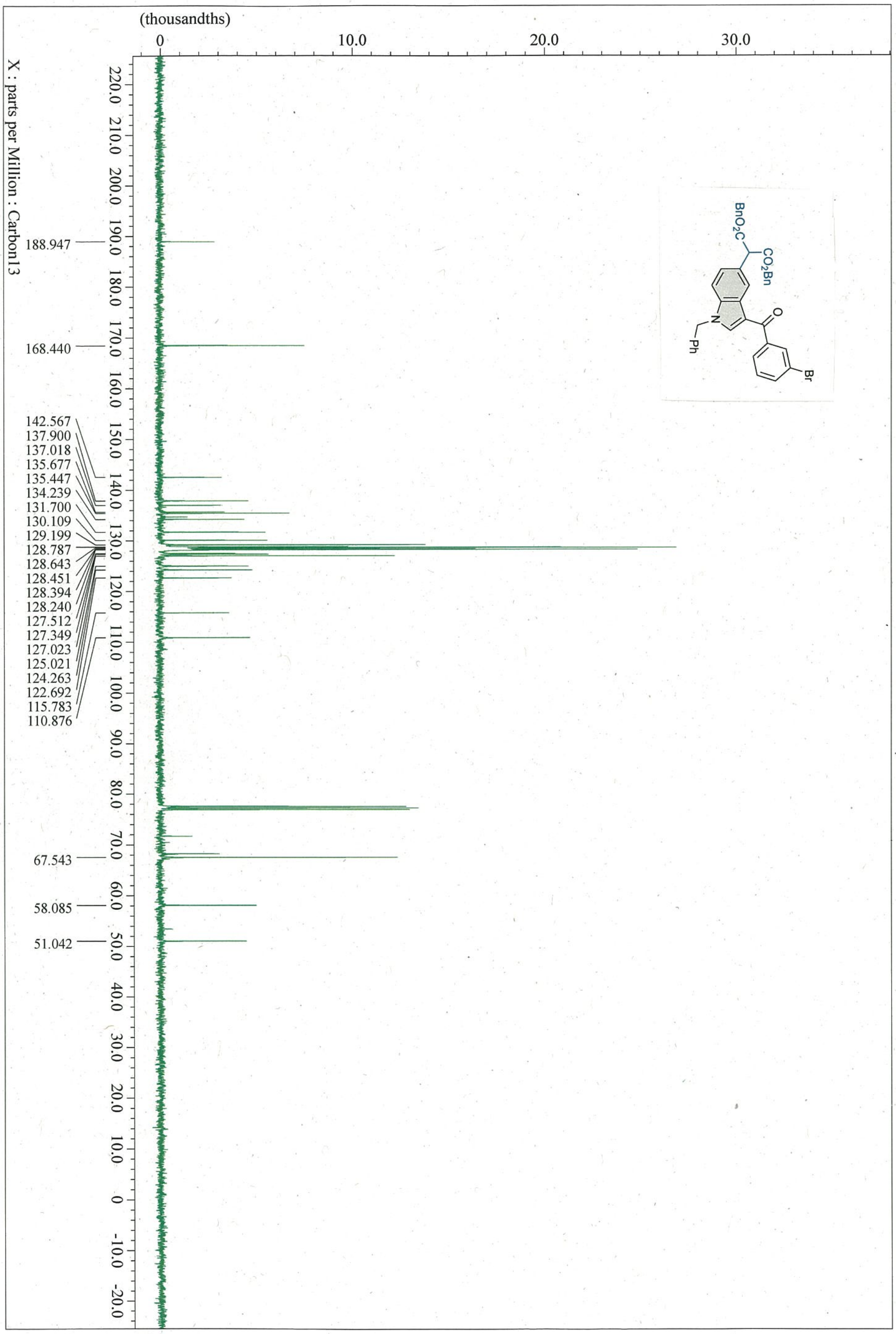
(thousandths)
0
10.0
20.0
30.0
X : parts per Million : Carbon13
188.947
168.440
142.567
137.900
137.018
135.677
135.447
134.239
131.700
130.109
129.199
128.787
128.643
128.451
128.394
128.240
127.512
127.349
127.023
125.021
124.263
122.692
115.783
110.876
67.543
58.085
51.042
BnO2C
CO2Bn
N
Ph
O
Br

3h

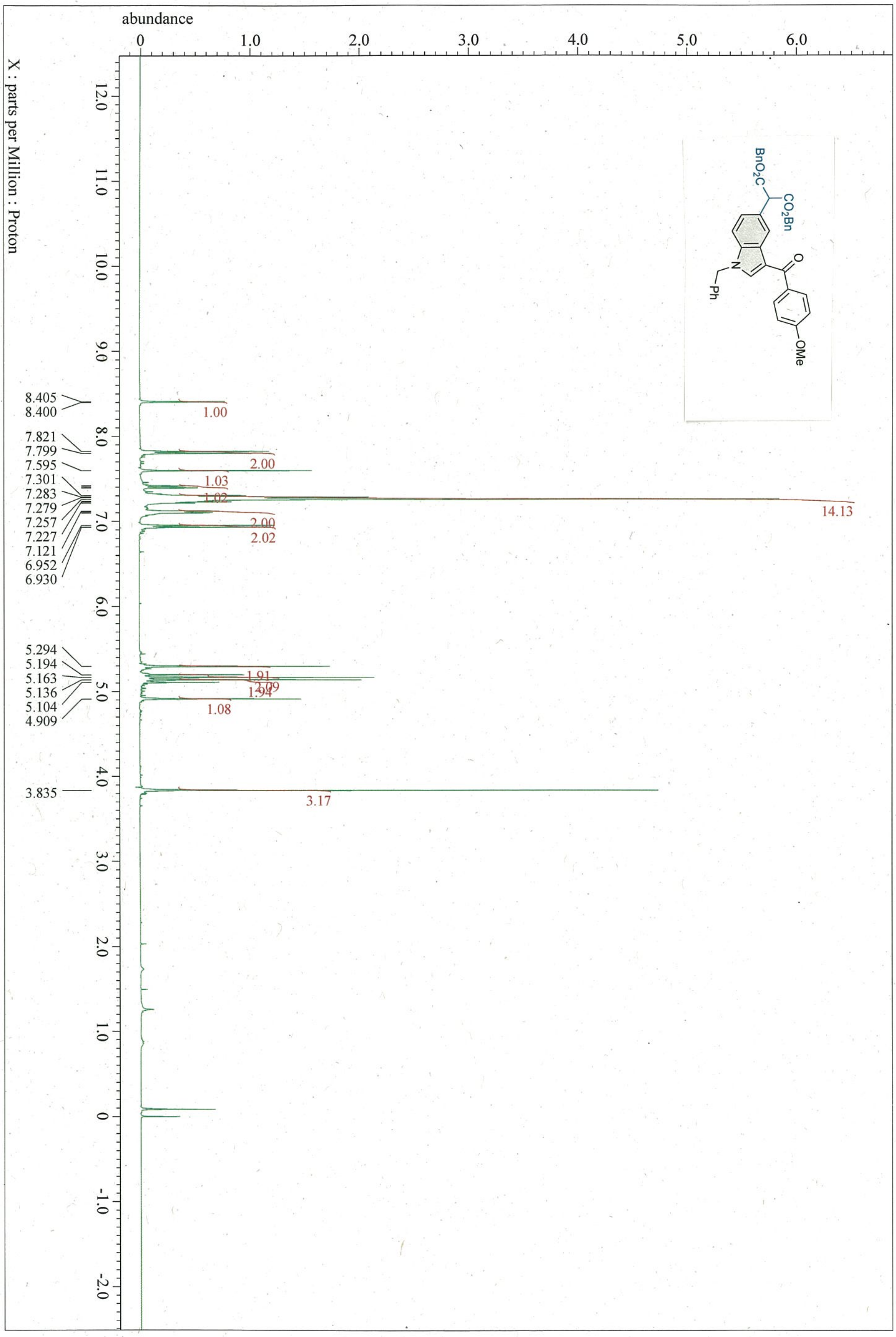
abundance
X : parts per Million : Proton
8.405
8.400
7.821
7.799
7.595
7.301
7.283
7.279
7.257
7.227
7.121
6.952
6.930
5.294
5.194
5.163
5.136
5.104
4.909
3.835
1.00
2.00
1.03
1.02
14.13
2.00
2.02
1.91
2.09
1.94
1.08
3.17
BnO2C
CO2Bn
N
O
Ph
OMe

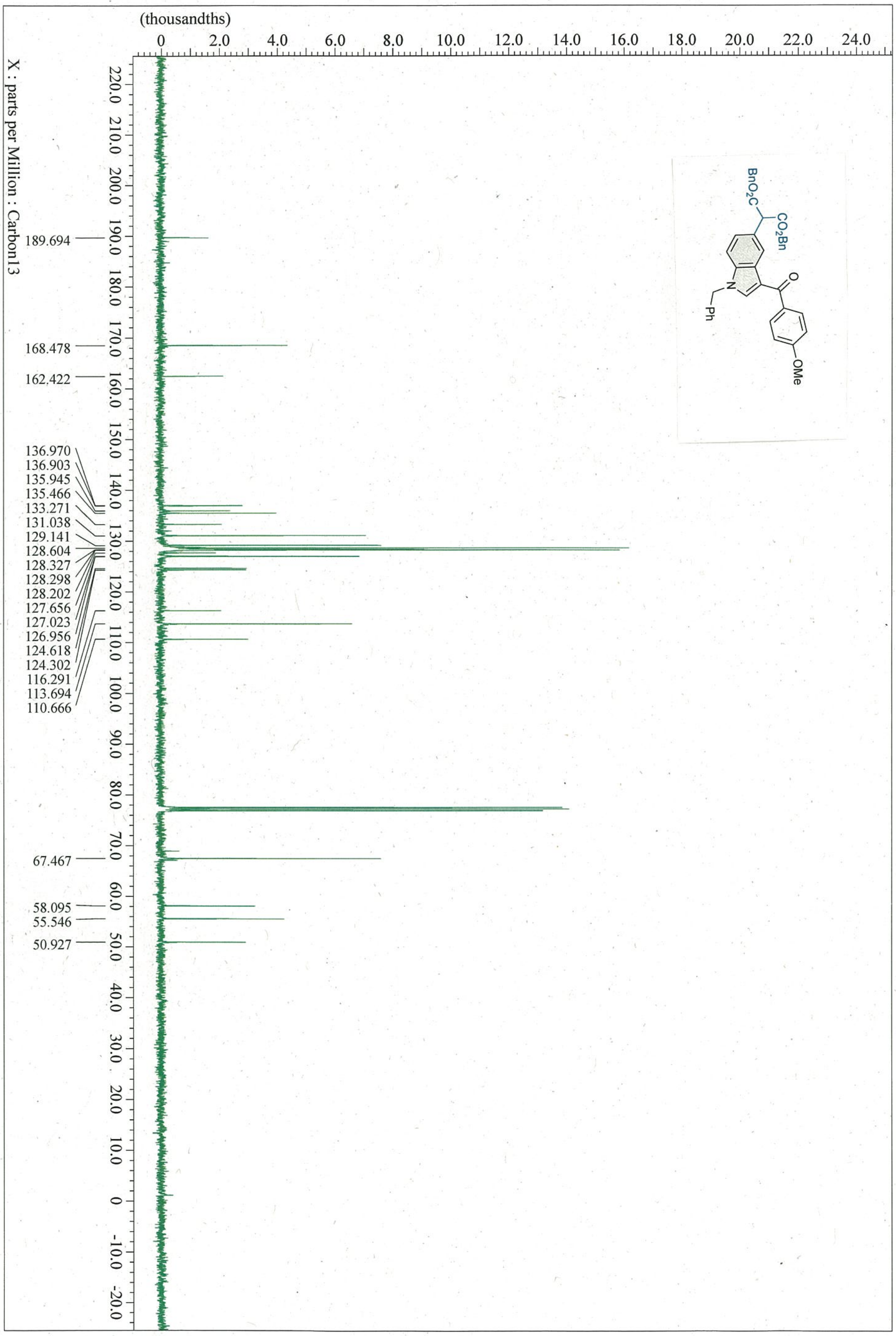
(thousandths)
X : parts per Million : Carbon13
189.694
168.478
162.422
136.970
136.903
135.945
135.466
133.271
131.038
129.141
128.604
128.327
128.298
128.202
127.656
127.023
126.956
124.618
124.302
116.291
113.694
110.666
67.467
58.095
55.546
50.927
BnO2C
CO2Bn
N
Ph
O
OMe

3i

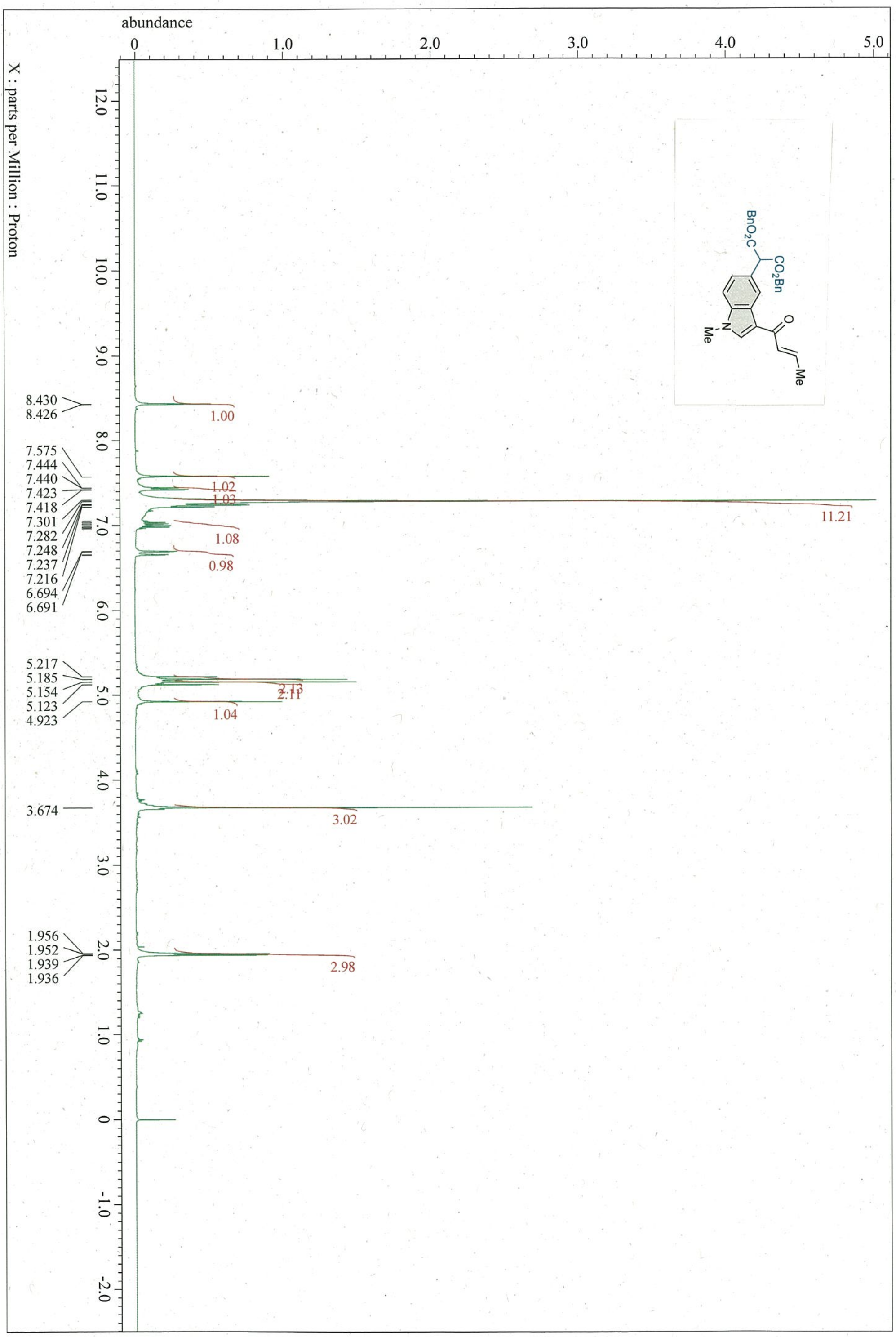
abundance
X : parts per Million : Proton
BnO2C
CO2Bn
N
Me
O
Me
8.430
8.426
7.575
7.444
7.440
7.423
7.418
7.301
7.282
7.248
7.237
7.216
6.694
6.691
5.217
5.185
5.154
5.123
4.923
3.674
1.956
1.952
1.939
1.936
1.00
1.02
1.03
11.21
1.08
0.98
2.13
2.11
1.04
3.02
2.98

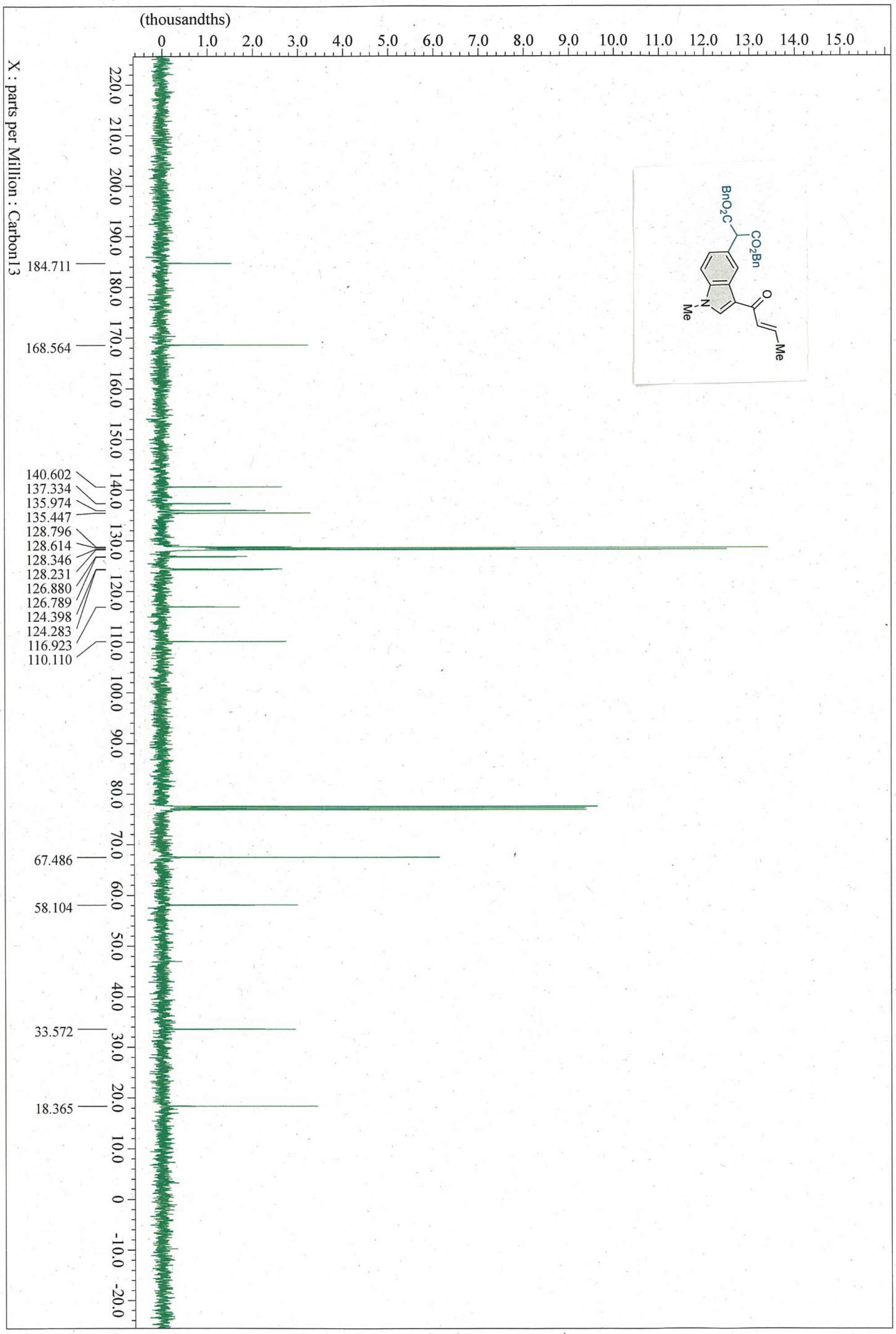
(thousandths)
0 1.0 2.0 3.0 4.0 5.0 6.0 7.0 8.0 9.0 10.0 11.0 12.0 13.0 14.0 15.0
X : parts per Million : Carbon13
220.0 210.0 200.0 190.0 180.0 170.0 160.0 150.0 140.0 130.0 120.0 110.0 100.0 90.0 80.0 70.0 60.0 50.0 40.0 30.0 20.0 10.0 0 -10.0 -20.0
184.711
168.564
140.602
137.334
135.974
135.447
128.796
128.614
128.346
128.231
126.880
126.789
124.398
124.283
116.923
110.110
67.486
58.104
33.572
18.365
BnO2C
CO2Bn
O
N
Me
Me

3j

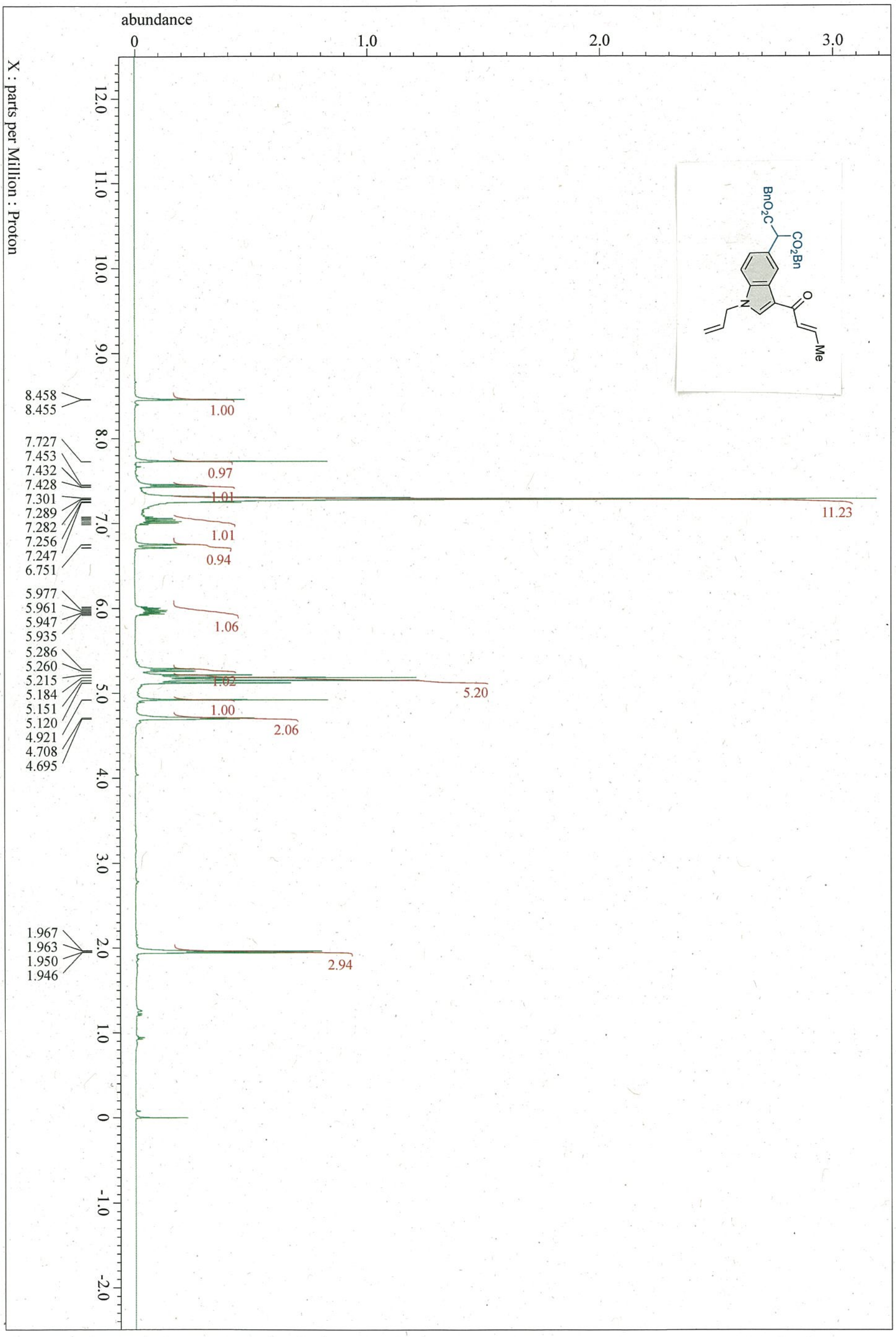
abundance
X : parts per Million : Proton
8.458
8.455
7.727
7.453
7.432
7.428
7.301
7.289
7.282
7.256
7.247
6.751
5.977
5.961
5.947
5.935
5.286
5.260
5.215
5.184
5.151
5.120
4.921
4.708
4.695
1.967
1.963
1.950
1.946
1.00
0.97
1.01
11.23
1.01
0.94
1.06
1.02
5.20
1.00
2.06
2.94
BnO2C
CO2Bn
N
O
Me

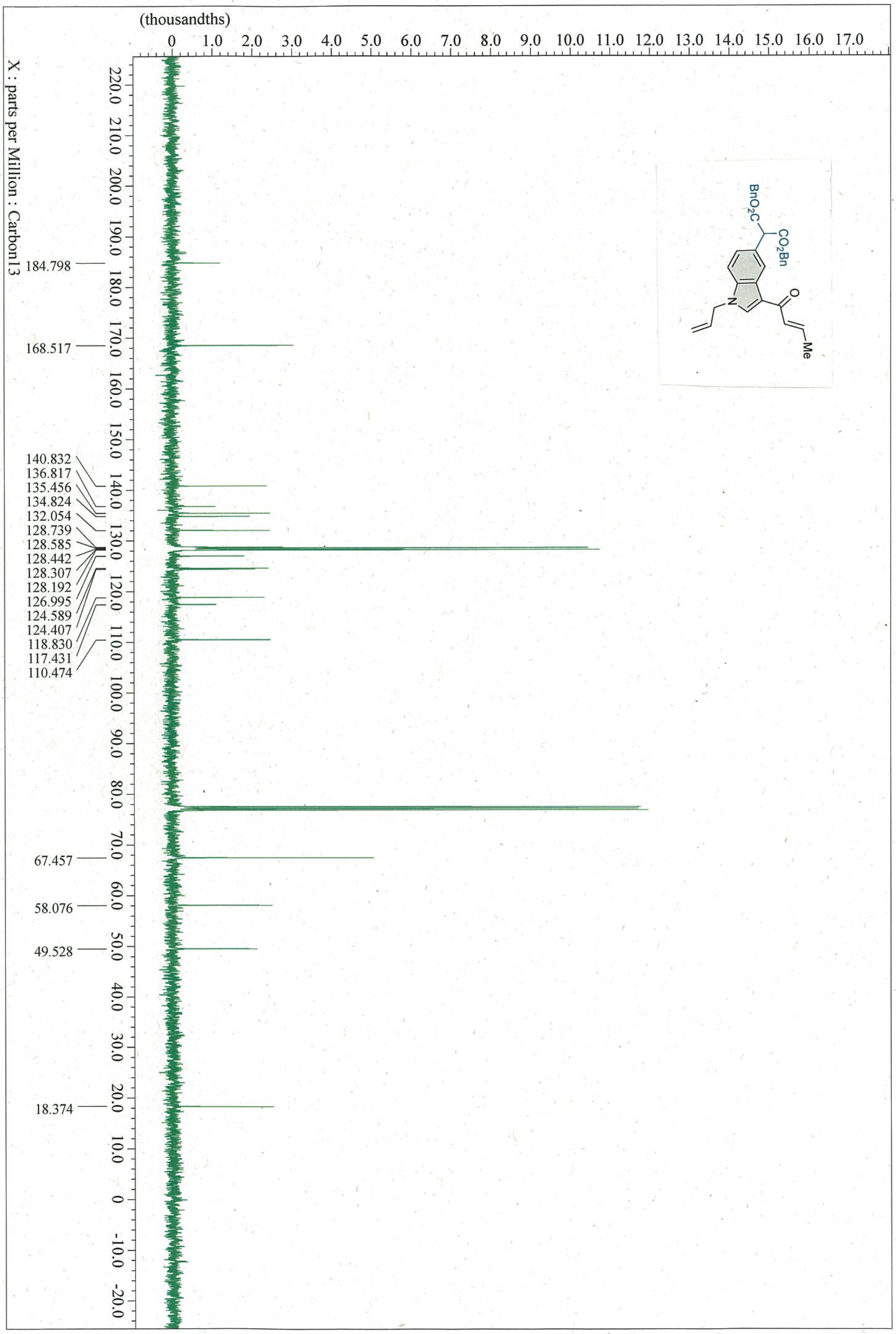

(thousandths)
X : parts per Million : Carbon13
184.798
168.517
140.832
136.817
135.456
134.824
132.054
128.739
128.585
128.442
128.307
128.192
126.995
124.589
124.407
118.830
117.431
110.474
67.457
58.076
49.528
18.374
BnO2C
CO2Bn
N
O
Me

3k

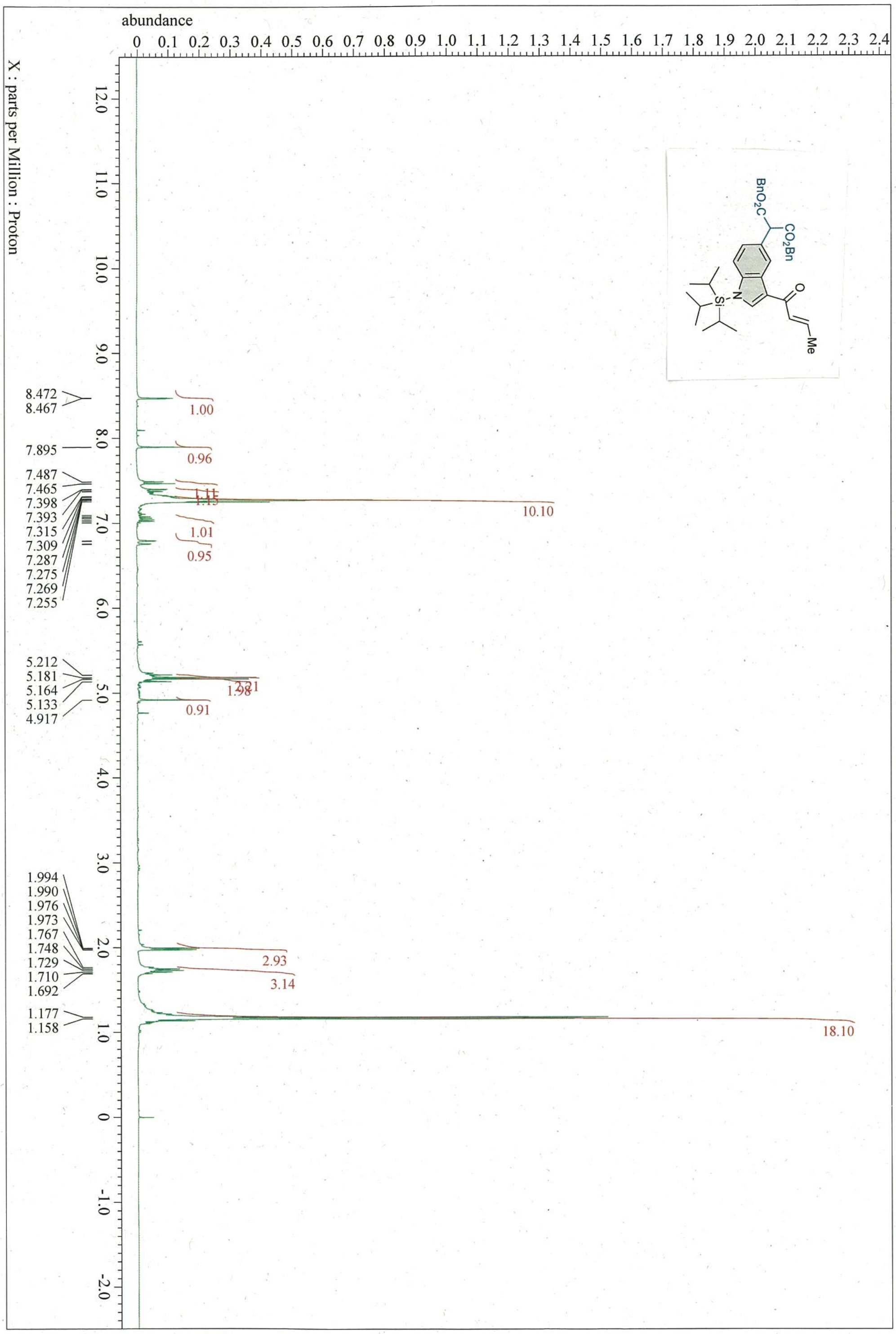

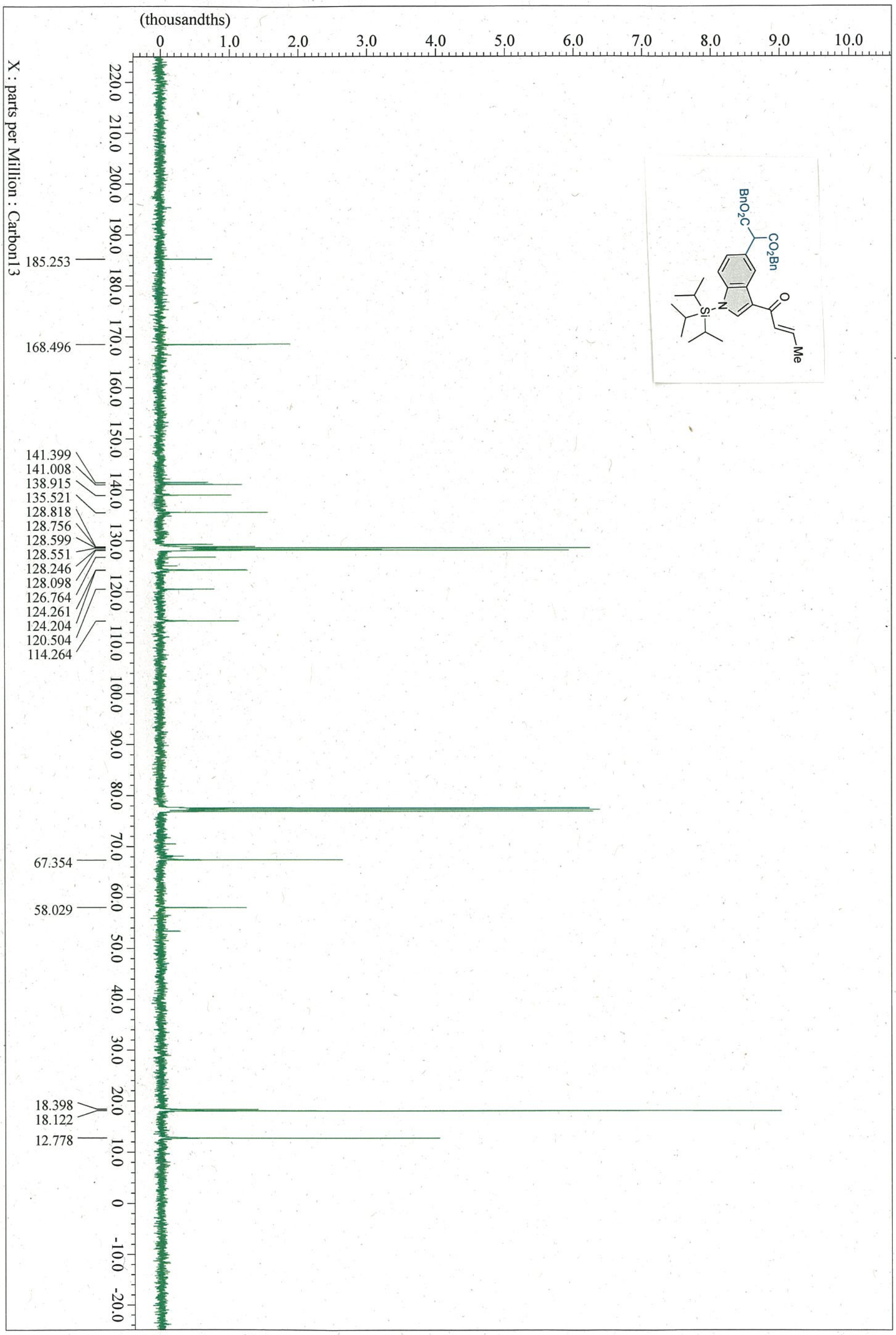
X : parts per Million : Carbon13
(thousandths)
185.253
168.496
141.399
141.008
138.915
135.521
128.818
128.756
128.599
128.551
128.246
128.098
126.764
124.261
124.204
120.504
114.264
67.354
58.029
18.398
18.122
12.778
BnO2C
CO2Bn
Si
N
O
Me

3l

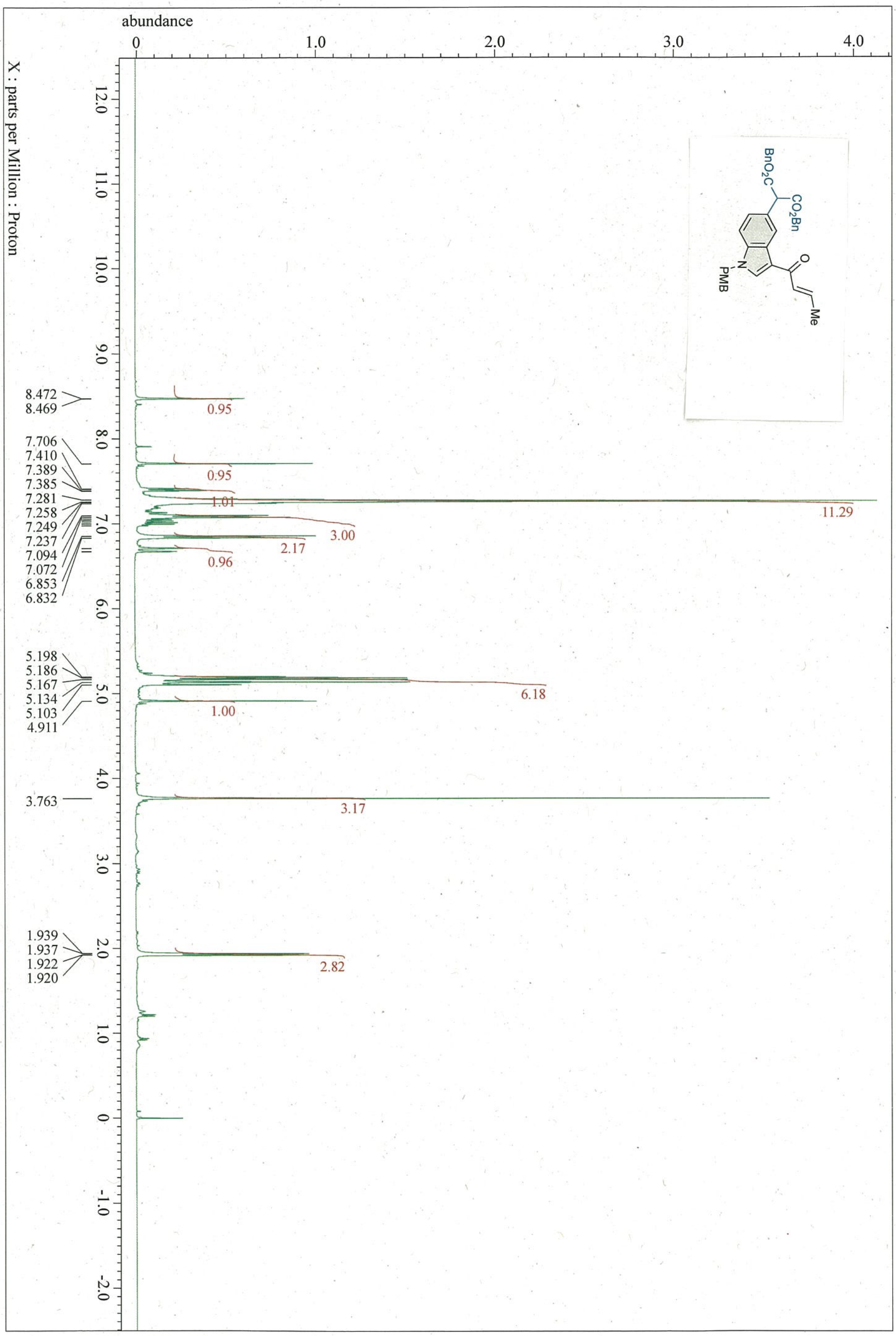
abundance
X : parts per Million : Proton
8.472
8.469
7.706
7.410
7.389
7.385
7.281
7.258
7.249
7.237
7.094
7.072
6.853
6.832
5.198
5.186
5.167
5.134
5.103
4.911
3.763
1.939
1.937
1.922
1.920
0.95
0.95
1.01
11.29
3.00
2.17
0.96
6.18
1.00
3.17
2.82
BnO2C
CO2Bn
N
PMB
O
Me

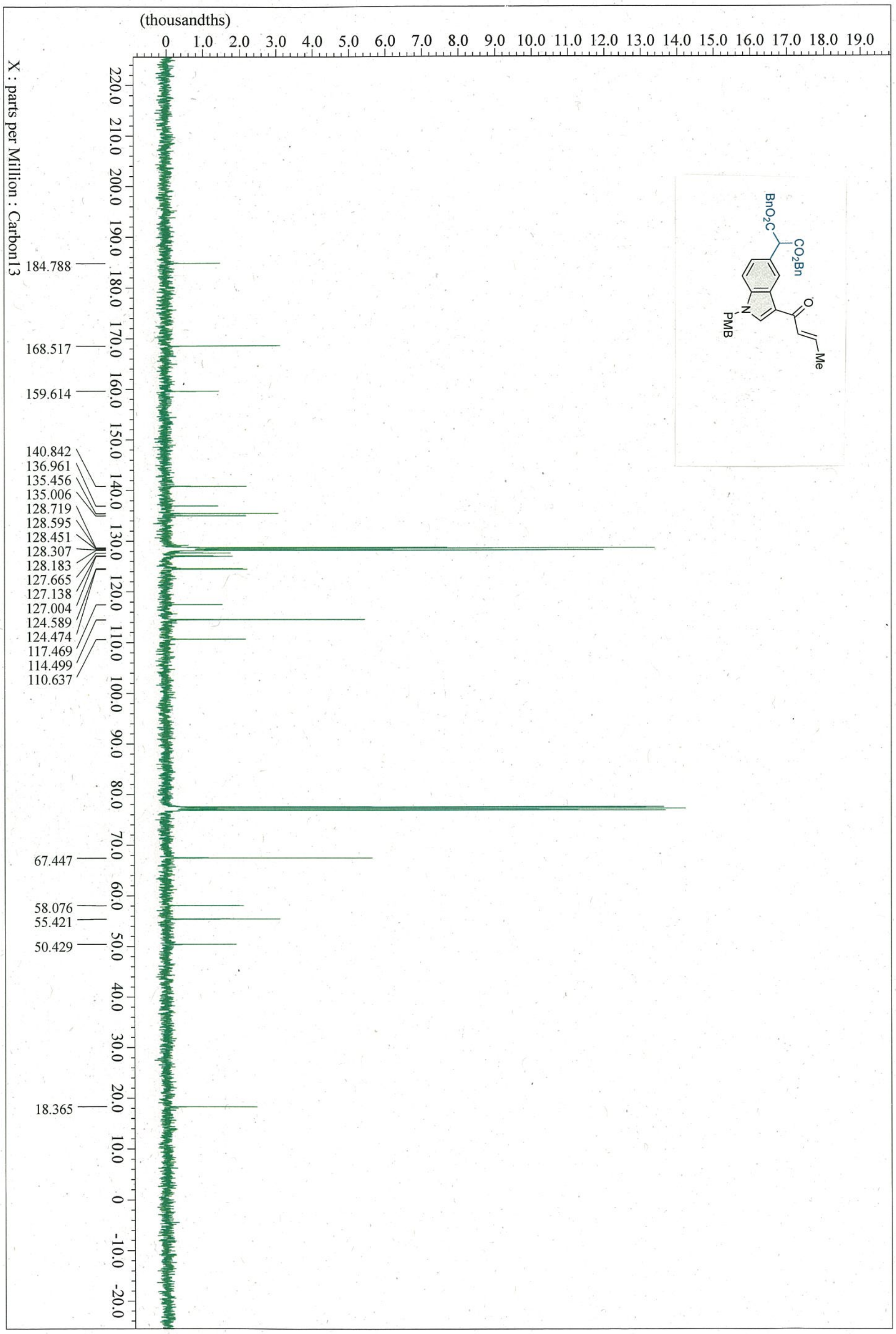
(thousandths)
X : parts per Million : Carbon13
184.788
168.517
159.614
140.842
136.961
135.456
135.006
128.719
128.595
128.451
128.307
128.183
127.665
127.138
127.004
124.589
124.474
117.469
114.499
110.637
67.447
58.076
55.421
50.429
18.365
BnO_2C
CO_2Bn
N
PMB
O
Me

3m

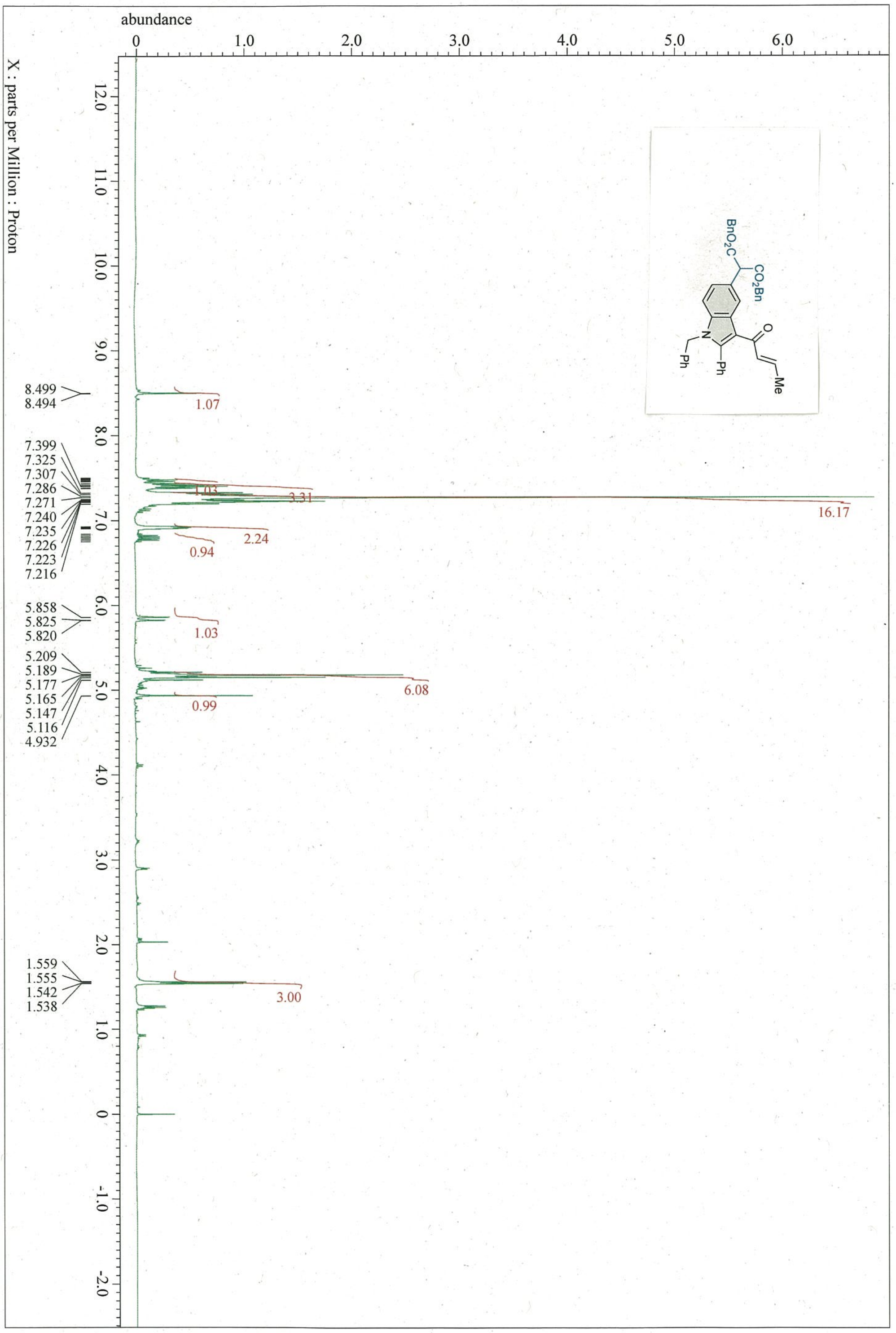
abundance
X : parts per Million : Proton
8.499
8.494
7.399
7.325
7.307
7.286
7.271
7.240
7.235
7.226
7.223
7.216
5.858
5.825
5.820
5.209
5.189
5.177
5.165
5.147
5.116
4.932
1.559
1.555
1.542
1.538
1.07
1.03
3.31
16.17
2.24
0.94
1.03
6.08
0.99
3.00
BnO2C
CO2Bn
N
Ph
Ph
O
Me

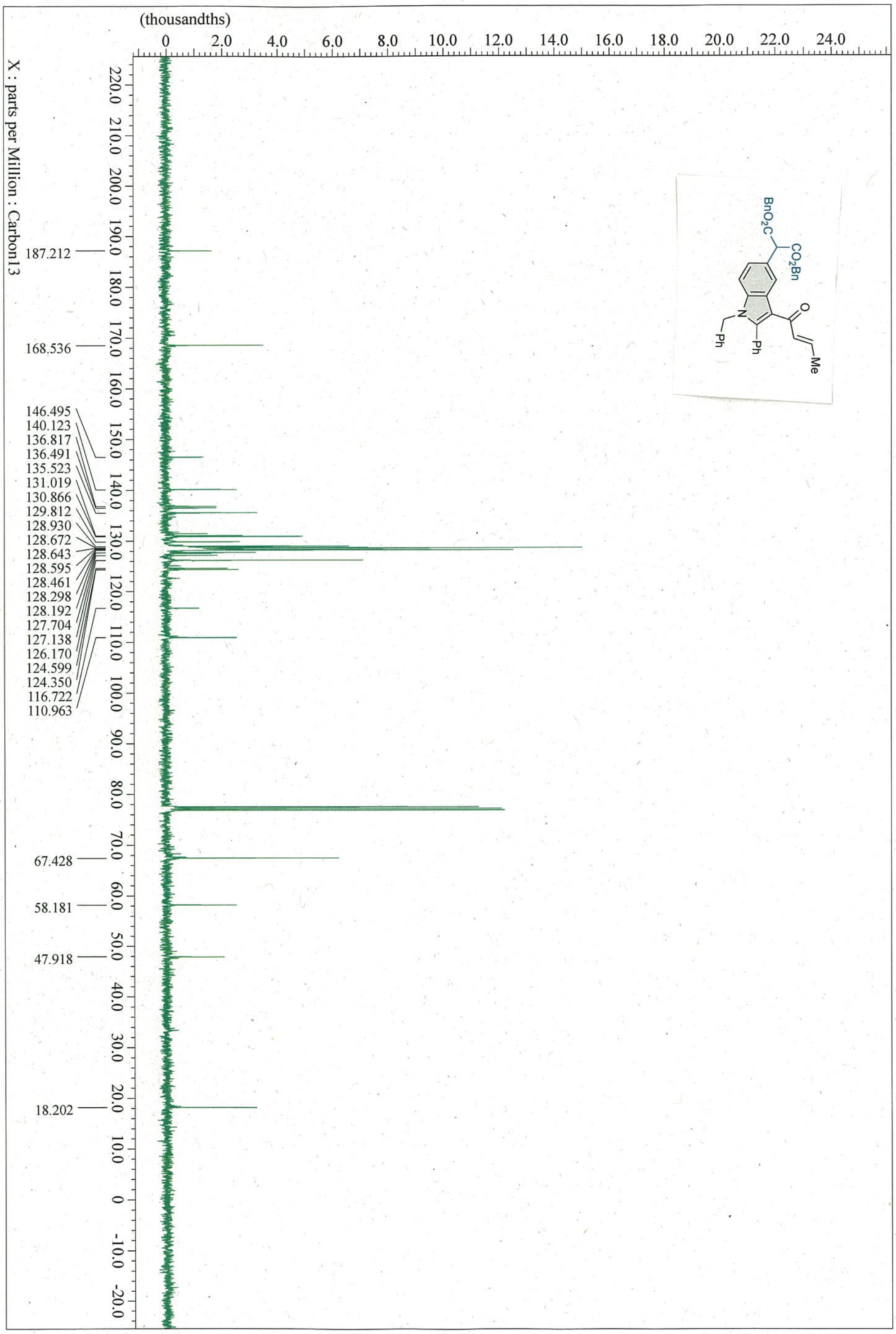
(thousandths)
0 2.0 4.0 6.0 8.0 10.0 12.0 14.0 16.0 18.0 20.0 22.0 24.0
X : parts per Million : Carbon13
220.0 210.0 200.0 190.0 180.0 170.0 160.0 150.0 140.0 130.0 120.0 110.0 100.0 90.0 80.0 70.0 60.0 50.0 40.0 30.0 20.0 10.0 0 -10.0 -20.0
187.212
168.536
146.495
140.123
136.817
136.491
135.523
131.019
130.866
129.812
128.930
128.672
128.643
128.595
128.461
128.298
128.192
127.704
127.138
126.170
124.599
124.350
116.722
110.963
67.428
58.181
47.918
18.202
BnO2C
CO2Bn
O
N
Ph
Ph
Me

3n

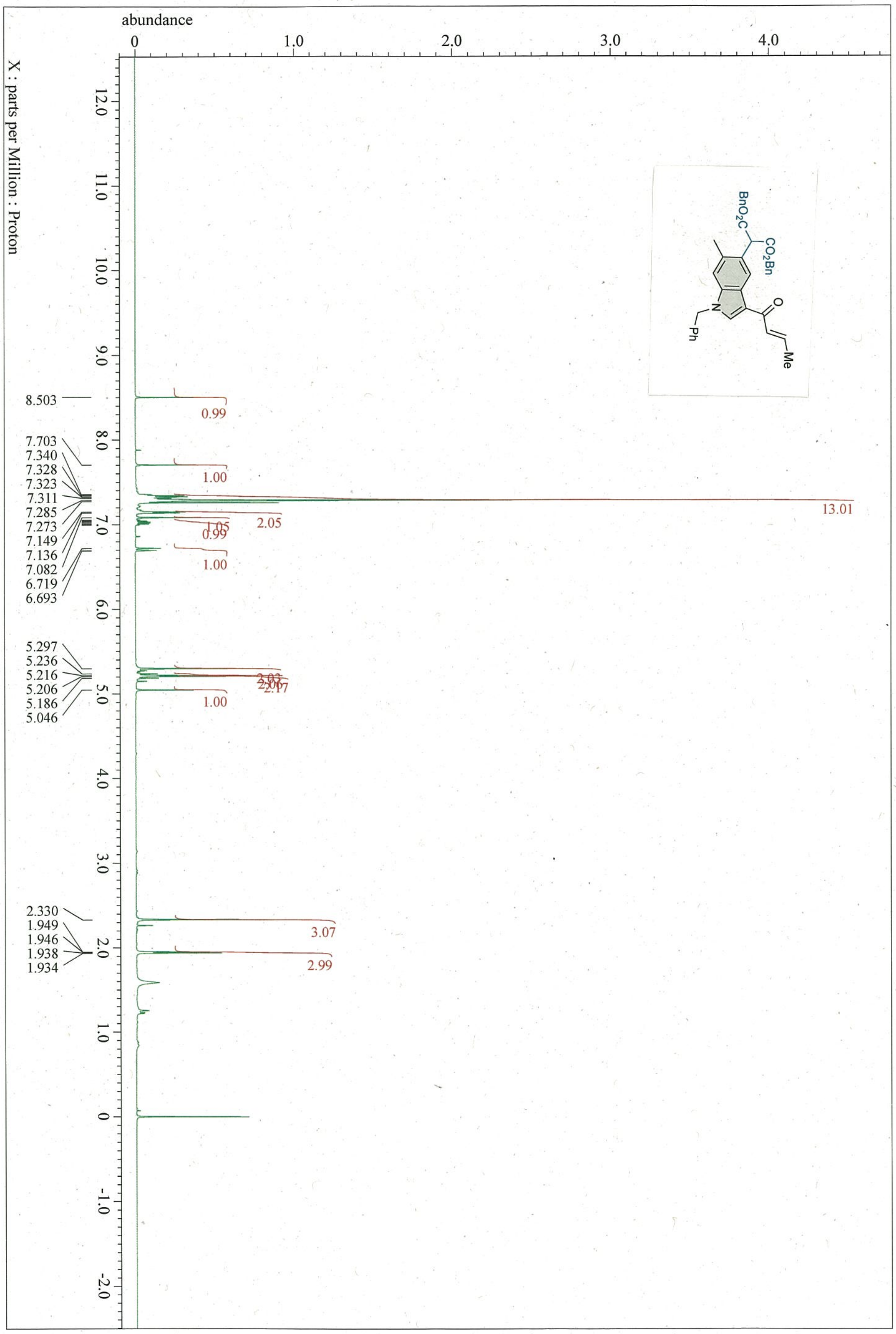
abundance
X : parts per Million : Proton
8.503
7.703
7.340
7.328
7.323
7.311
7.285
7.273
7.149
7.136
7.082
6.719
6.693
5.297
5.236
5.216
5.206
5.186
5.046
2.330
1.949
1.946
1.938
1.934
0.99
1.00
13.01
2.05
1.05
0.99
1.00
2.07
2.00
2.17
1.00
3.07
2.99
BnO2C
CO2Bn
N
Ph
O
Me

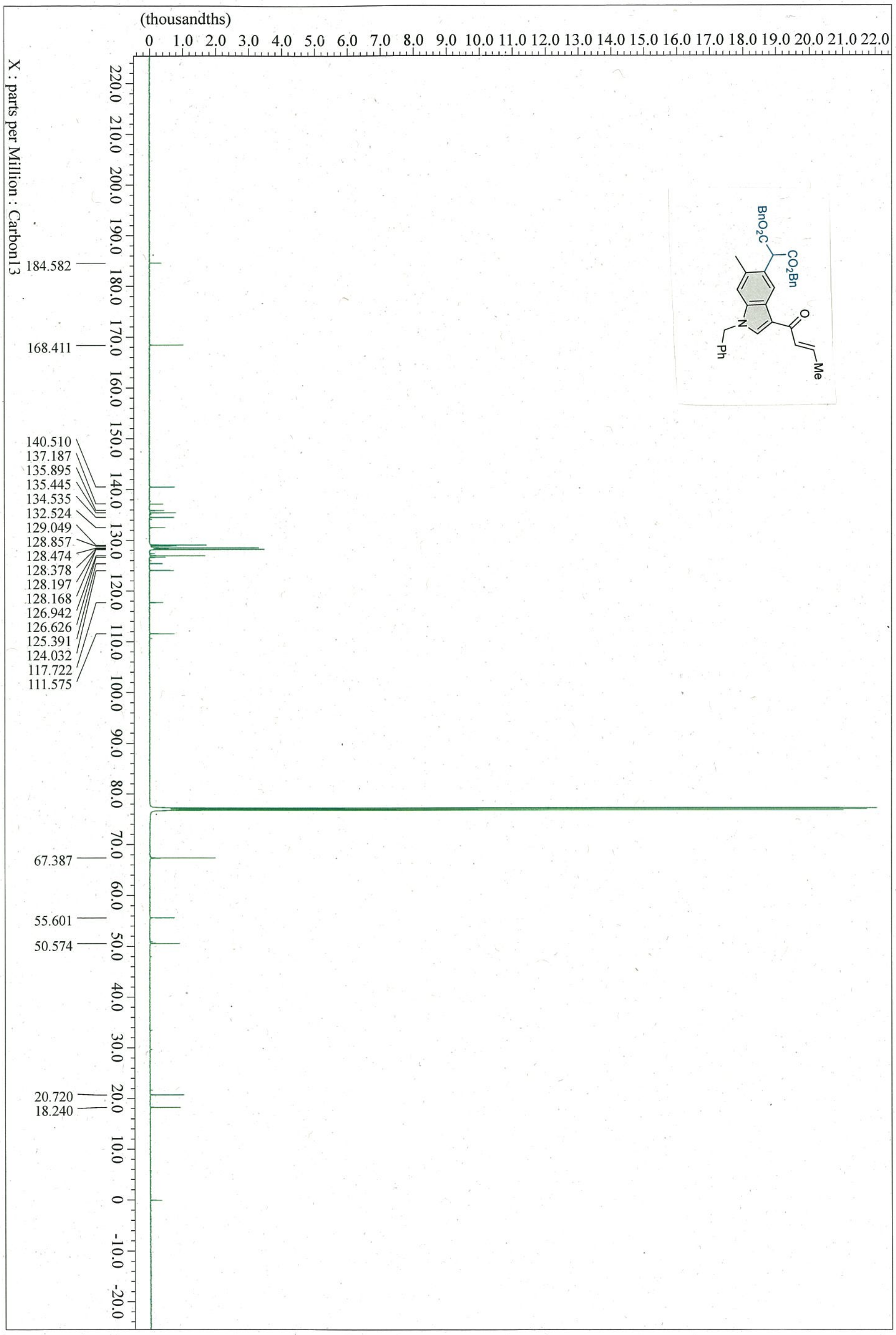

X : parts per Million : Carbon13
(thousandths)
184.582
168.411
140.510
137.187
135.895
135.445
134.535
132.524
129.049
128.857
128.474
128.378
128.197
128.168
126.942
126.626
125.391
124.032
117.722
111.575
67.387
55.601
50.574
20.720
18.240
BnO2C
CO2Bn
N
Ph
O
Me

3o

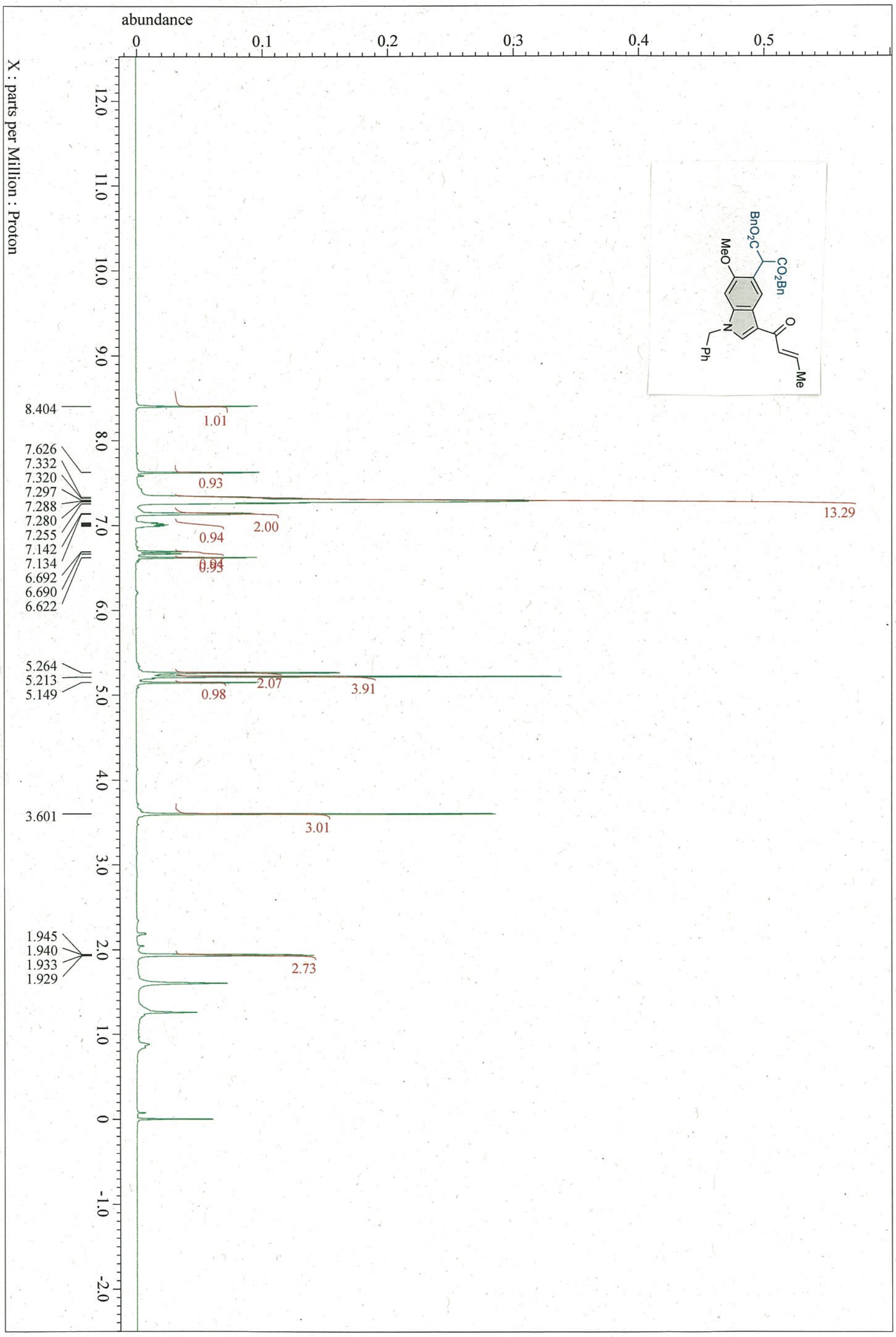
abundance
0
0.1
0.2
0.3
0.4
0.5
X : parts per Million : Proton
12.0
11.0
10.0
9.0
8.0
7.0
6.0
5.0
4.0
3.0
2.0
1.0
0
-1.0
-2.0
8.404
7.626
7.332
7.320
7.297
7.288
7.280
7.255
7.142
7.134
6.692
6.690
6.622
5.264
5.213
5.149
3.601
1.945
1.940
1.933
1.929
1.01
0.93
13.29
2.00
0.94
2.07
3.91
0.98
3.01
2.73
BnO2C
CO2Bn
MeO
O
N
Ph
Me

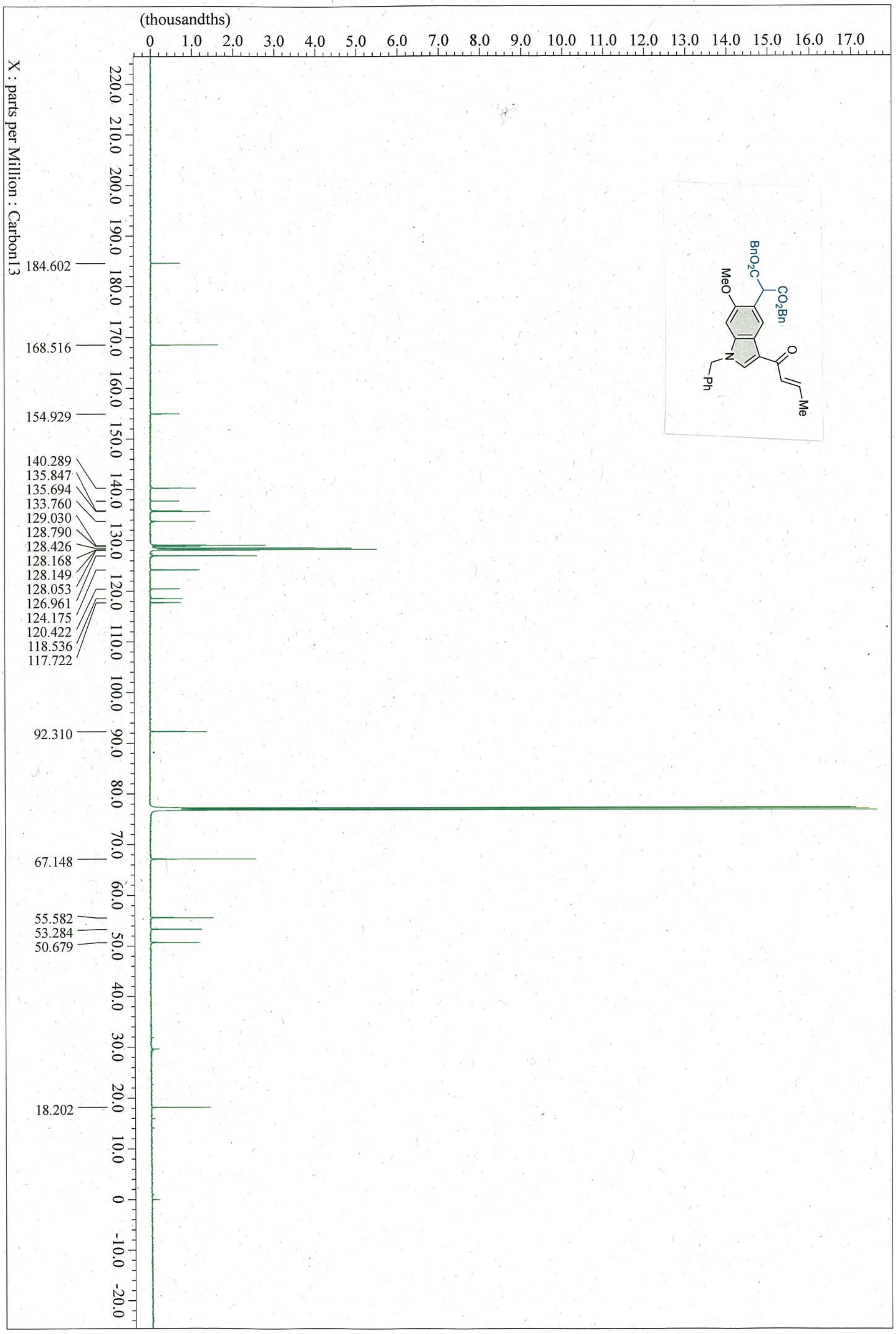

(thousandths)
X : parts per Million : Carbon13
184.602
168.516
154.929
140.289
135.847
135.694
133.760
129.030
128.790
128.426
128.168
128.149
128.053
126.961
124.175
120.422
118.536
117.722
92.310
67.148
55.582
53.284
50.679
18.202
BnO2C
CO2Bn
MeO
N
Ph
O
Me

3p

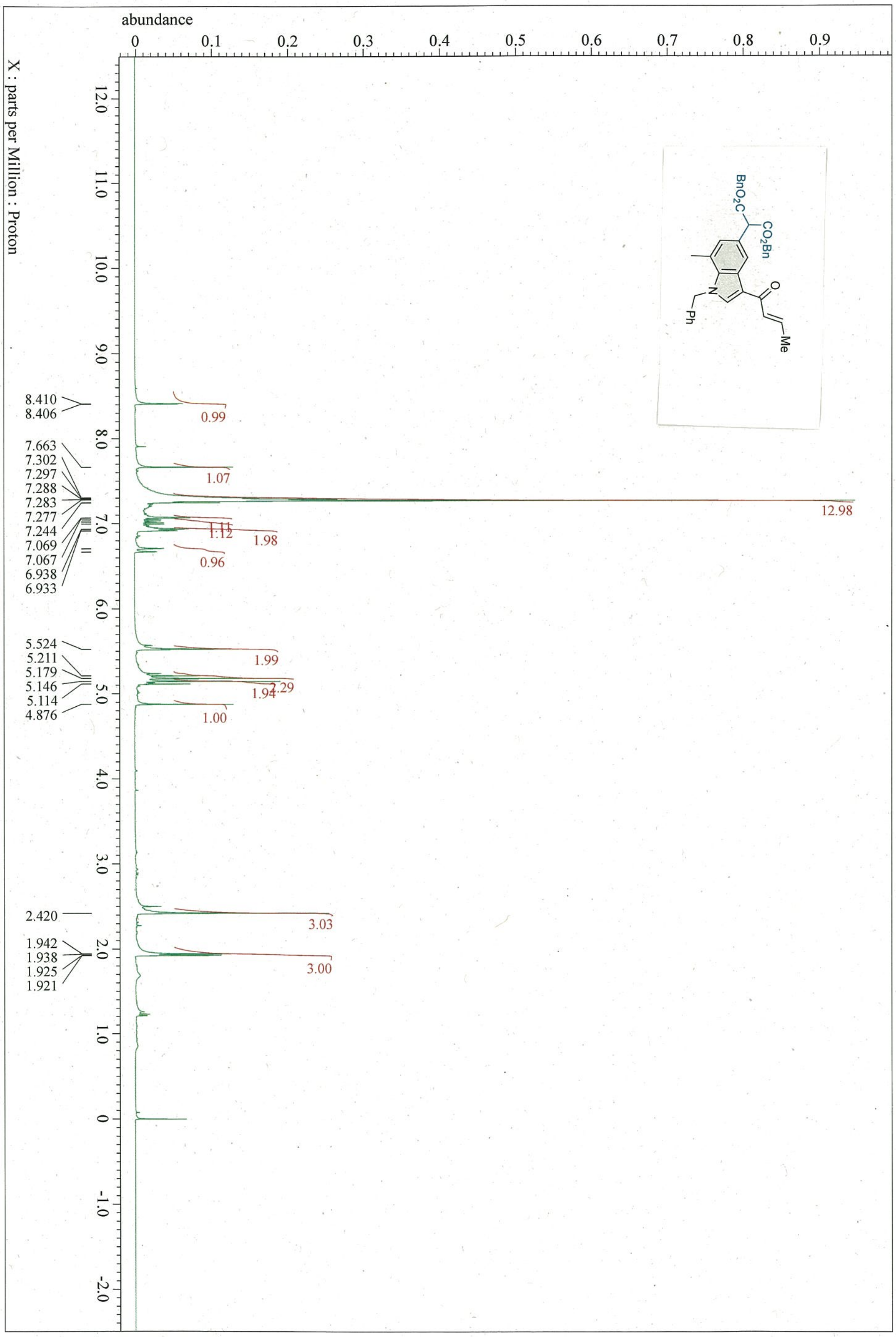

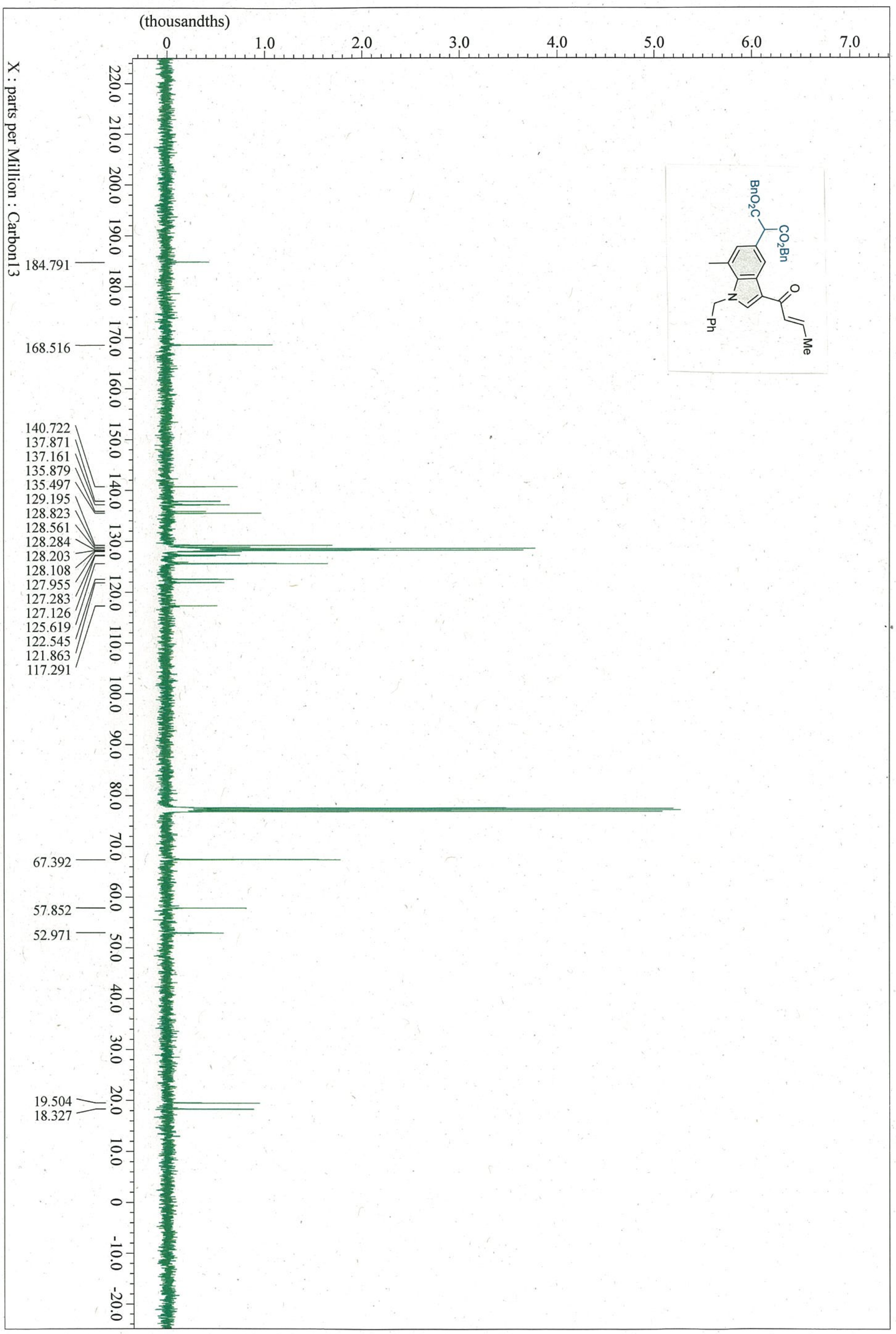
X : parts per Million : Carbon13
(thousandths)
184.791
168.516
140.722
137.871
137.161
135.879
135.497
129.195
128.823
128.561
128.284
128.203
128.108
127.955
127.283
127.126
125.619
122.545
121.863
117.291
67.392
57.852
52.971
19.504
18.327
BnO2C
CO2Bn
N
O
Ph
Me

3q

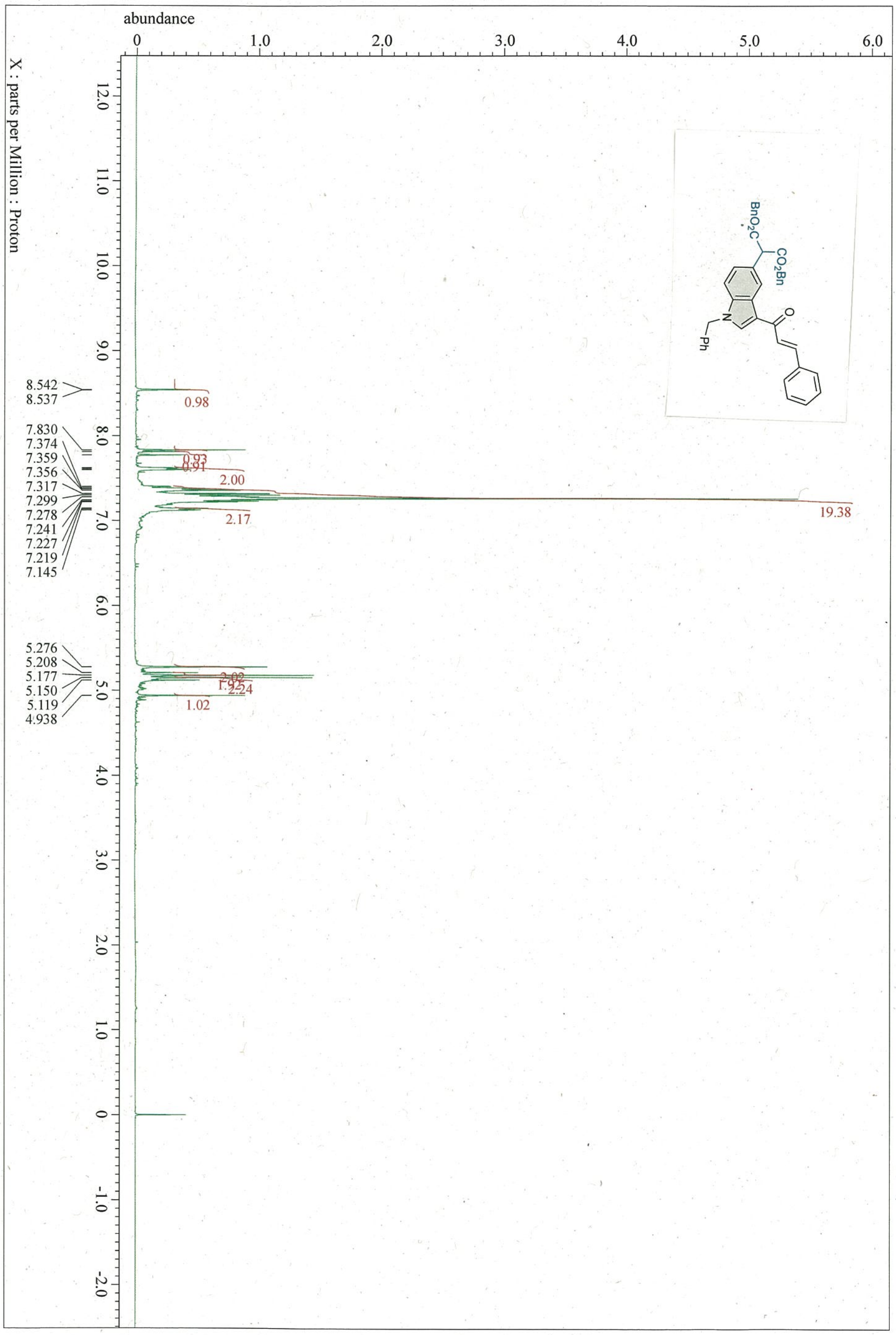
abundance
X : parts per Million : Proton
8.542
8.537
7.830
7.374
7.359
7.356
7.317
7.299
7.278
7.241
7.227
7.219
7.145
5.276
5.208
5.177
5.150
5.119
4.938
0.98
2.00
2.17
19.38
1.02
BnO2C
CO2Bn
N
Ph
O

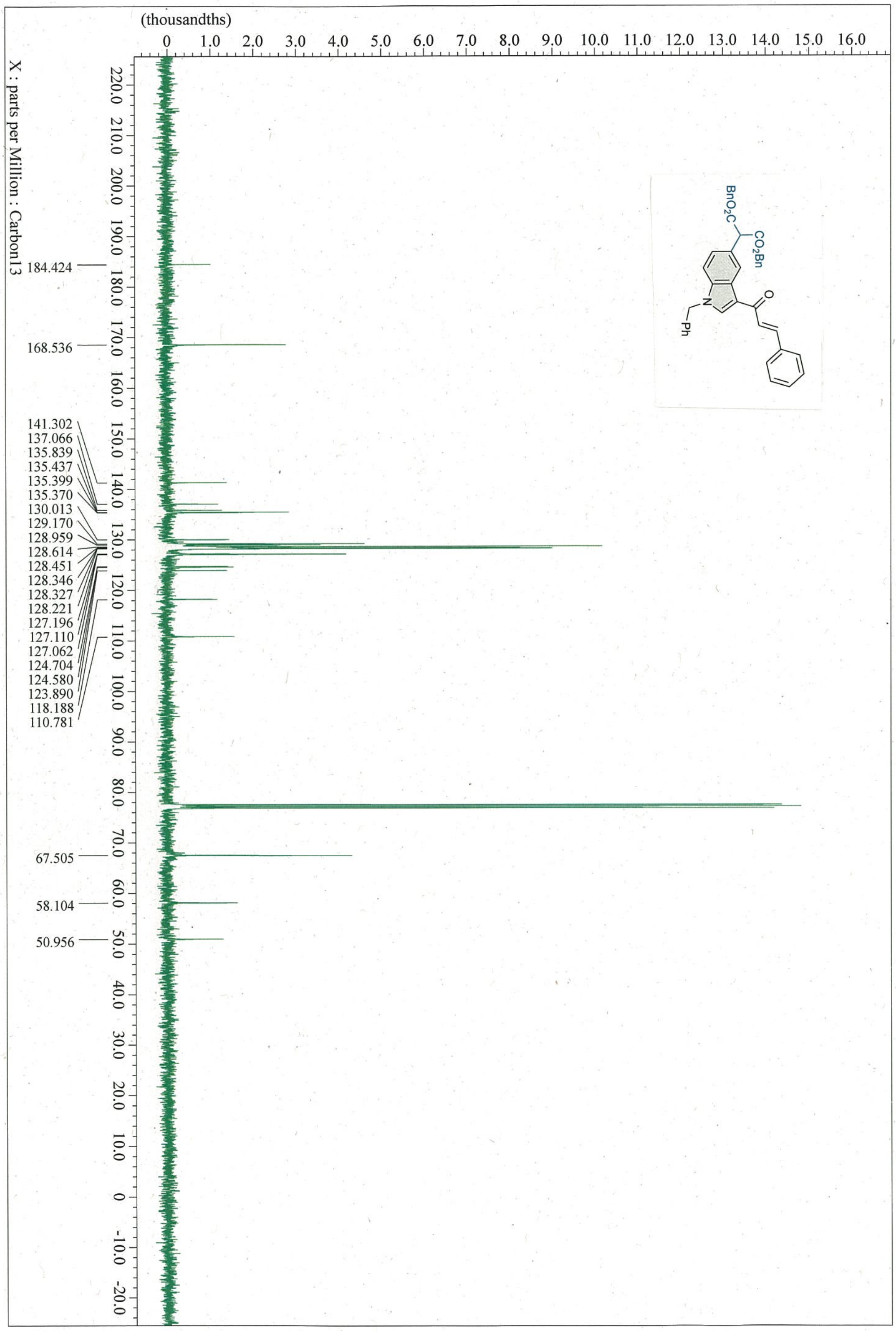

X : parts per Million : Carbon13
(thousandths)
184.424
168.536
141.302
137.066
135.839
135.437
135.399
135.370
130.013
129.170
128.959
128.614
128.451
128.346
128.327
128.221
127.196
127.110
127.062
124.704
124.580
123.890
118.188
110.781
67.505
58.104
50.956
BnO2C
CO2Bn
O
N
Ph

3r

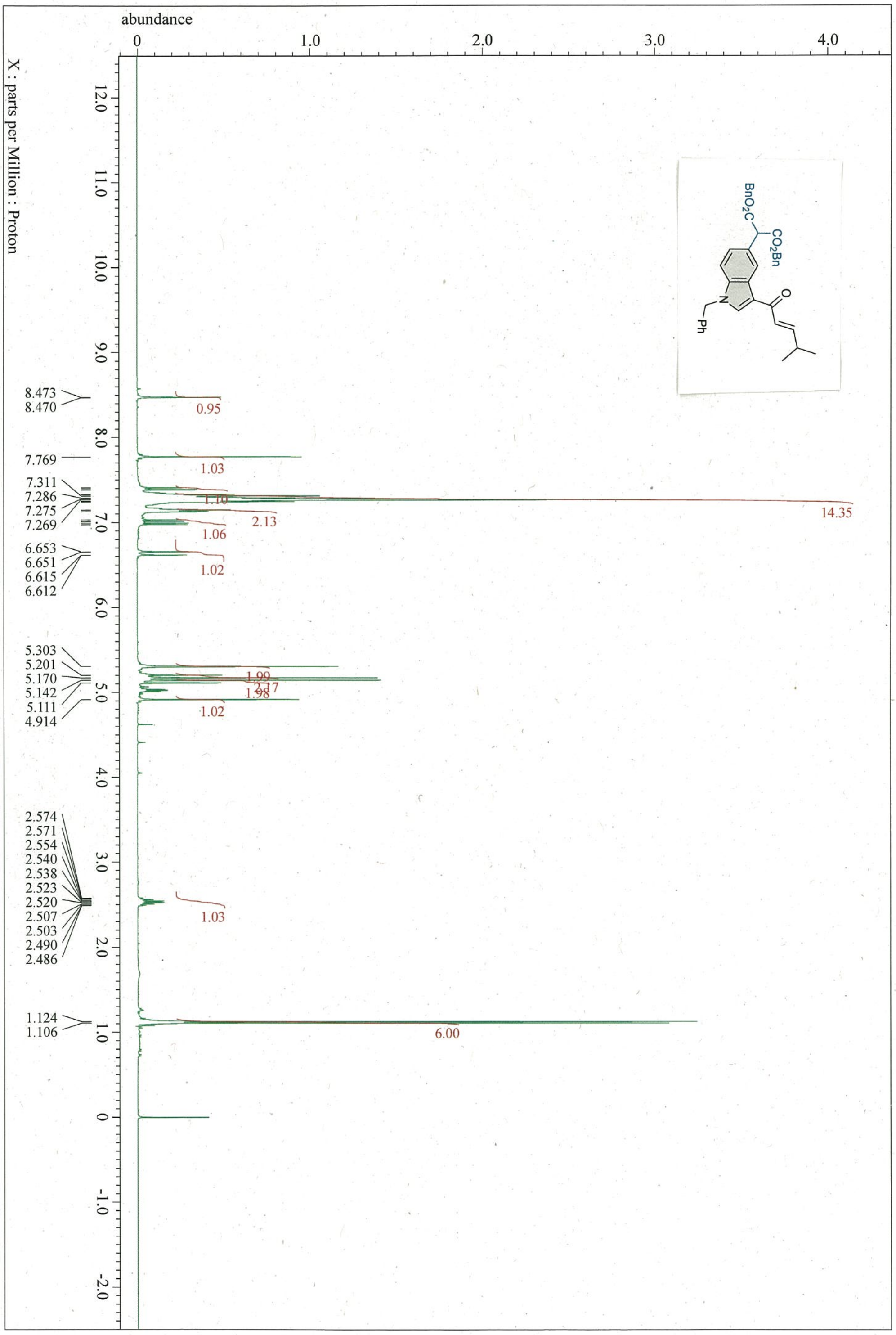

abundance
X : parts per Million : Proton
8.473
8.470
7.769
7.311
7.286
7.275
7.269
6.653
6.651
6.615
6.612
5.303
5.201
5.170
5.142
5.111
4.914
2.574
2.571
2.554
2.540
2.538
2.523
2.520
2.507
2.503
2.490
2.486
1.124
1.106
0.95
1.03
14.35
2.13
1.06
1.02
1.02
1.03
6.00
BnO2C
CO2Bn
N
O
Ph

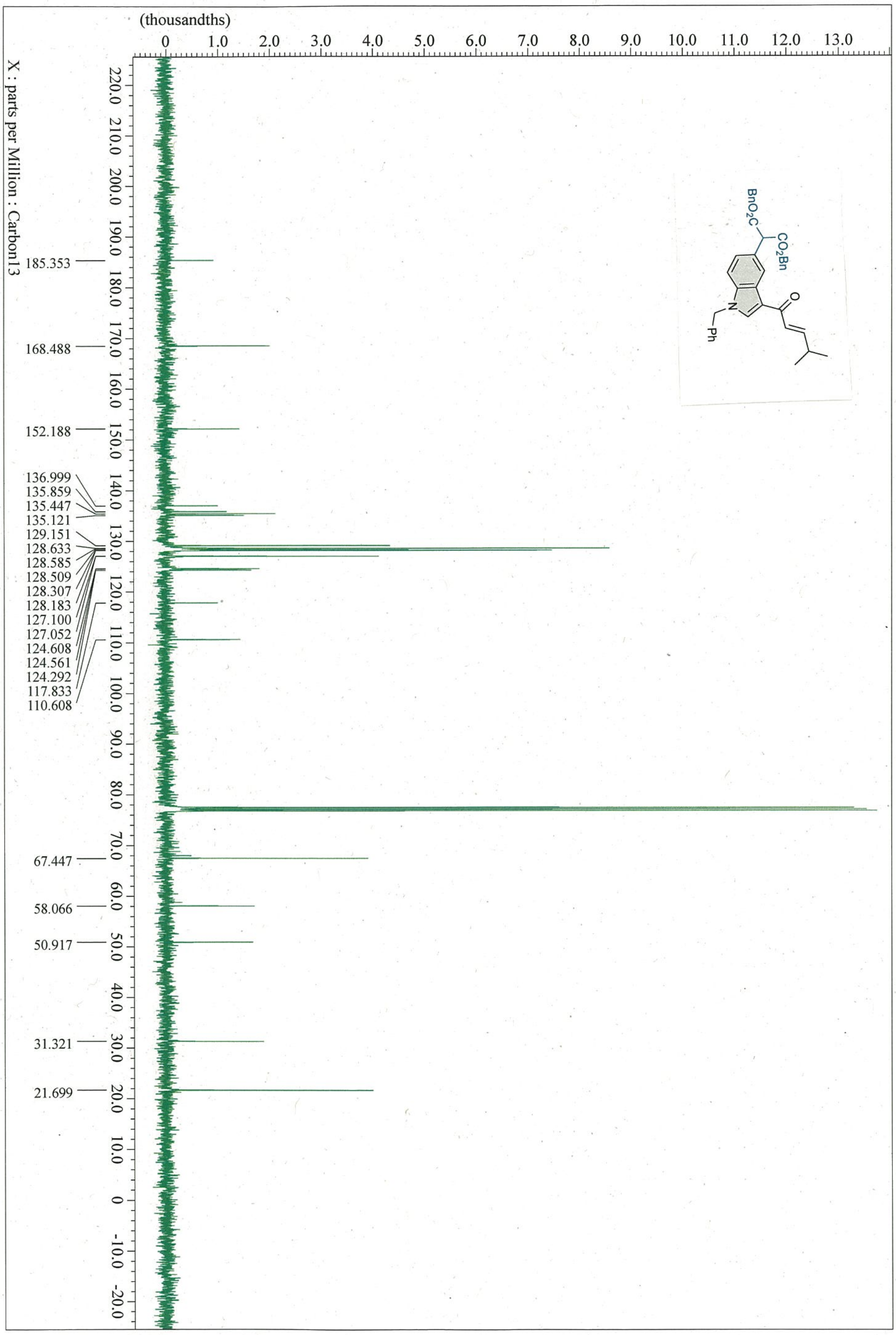
(thousandths)
X : parts per Million : Carbon13
185.353
168.488
152.188
136.999
135.859
135.447
135.121
129.151
128.633
128.585
128.509
128.307
128.183
127.100
127.052
124.608
124.561
124.292
117.833
110.608
67.447
58.066
50.917
31.321
21.699
BnO2C
CO2Bn
N
Ph
O

3s

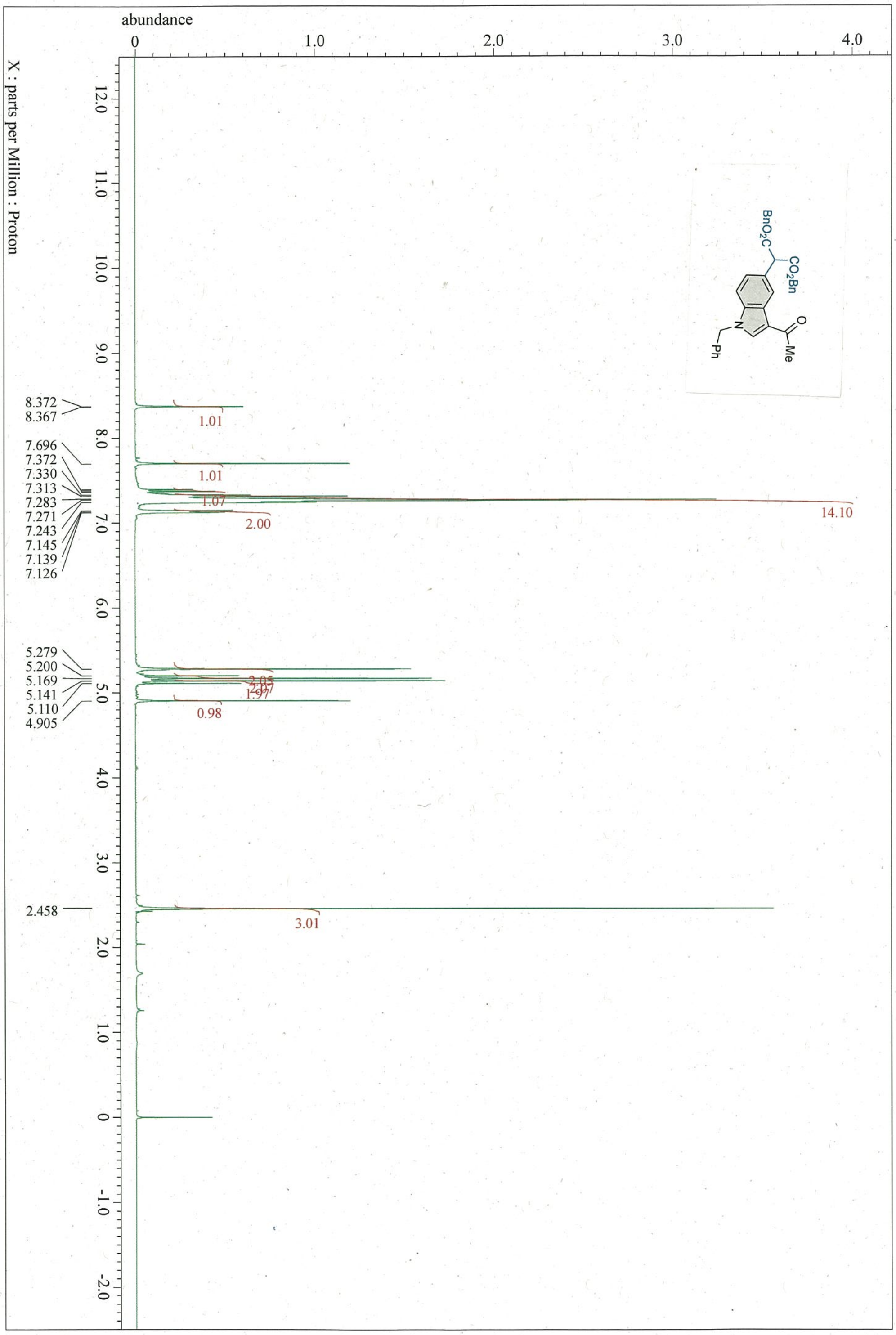

abundance
0
1.0
2.0
3.0
4.0
X : parts per Million : Proton
12.0
11.0
10.0
9.0
8.0
7.0
6.0
5.0
4.0
3.0
2.0
1.0
0
-1.0
-2.0
8.372
8.367
7.696
7.372
7.330
7.313
7.283
7.271
7.243
7.145
7.139
7.126
5.279
5.200
5.169
5.141
5.110
4.905
2.458
1.01
1.01
1.07
14.10
2.00
2.05
1.97
0.98
3.01
BnO2C
CO2Bn
N
O
Me
Ph

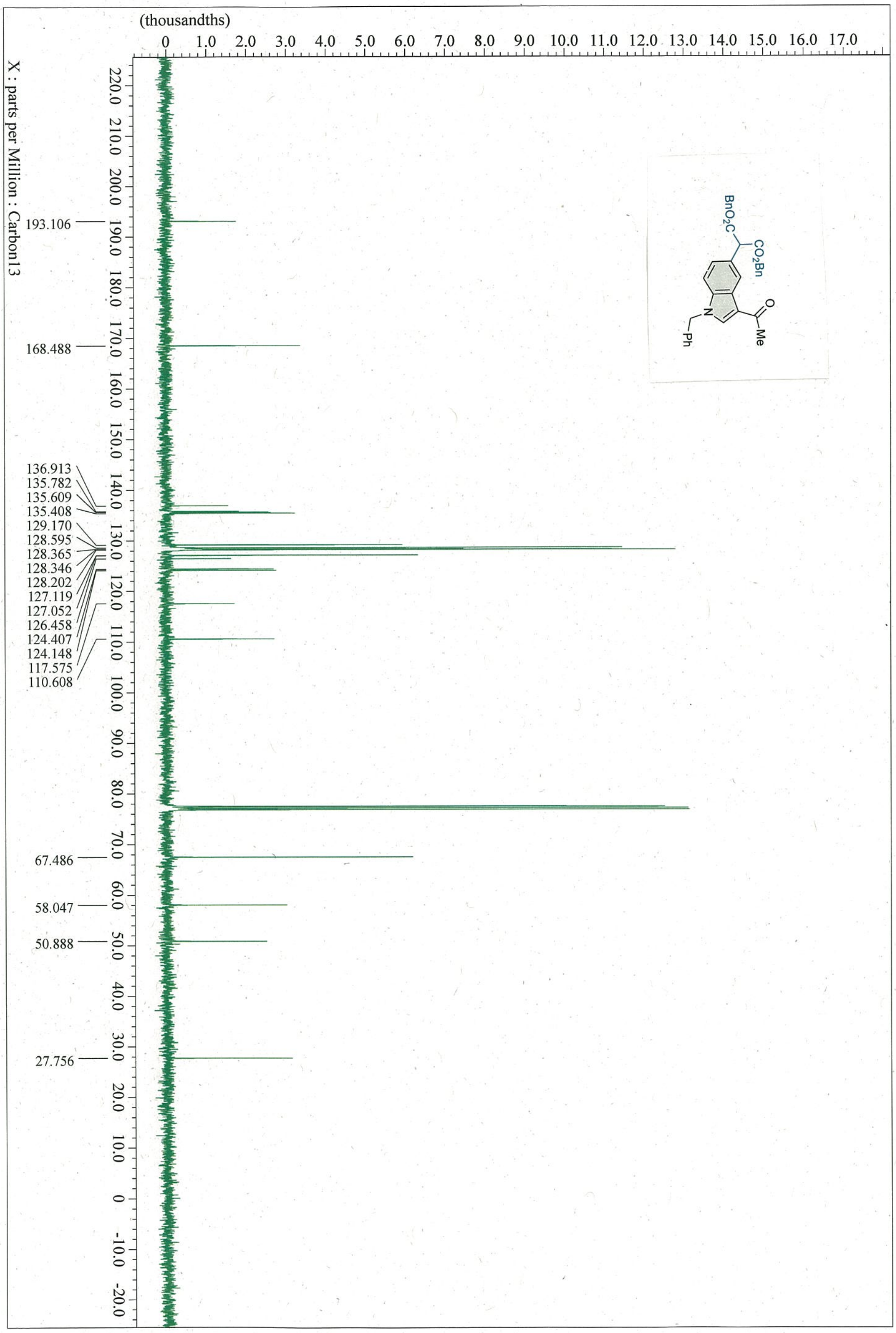
(thousandths)
X : parts per Million : Carbon13
193.106
168.488
136.913
135.782
135.609
135.408
129.170
128.595
128.365
128.346
128.202
127.119
127.052
126.458
124.407
124.148
117.575
110.608
67.486
58.047
50.888
27.756
BnO2C
CO2Bn
O
Me
N
Ph

3t

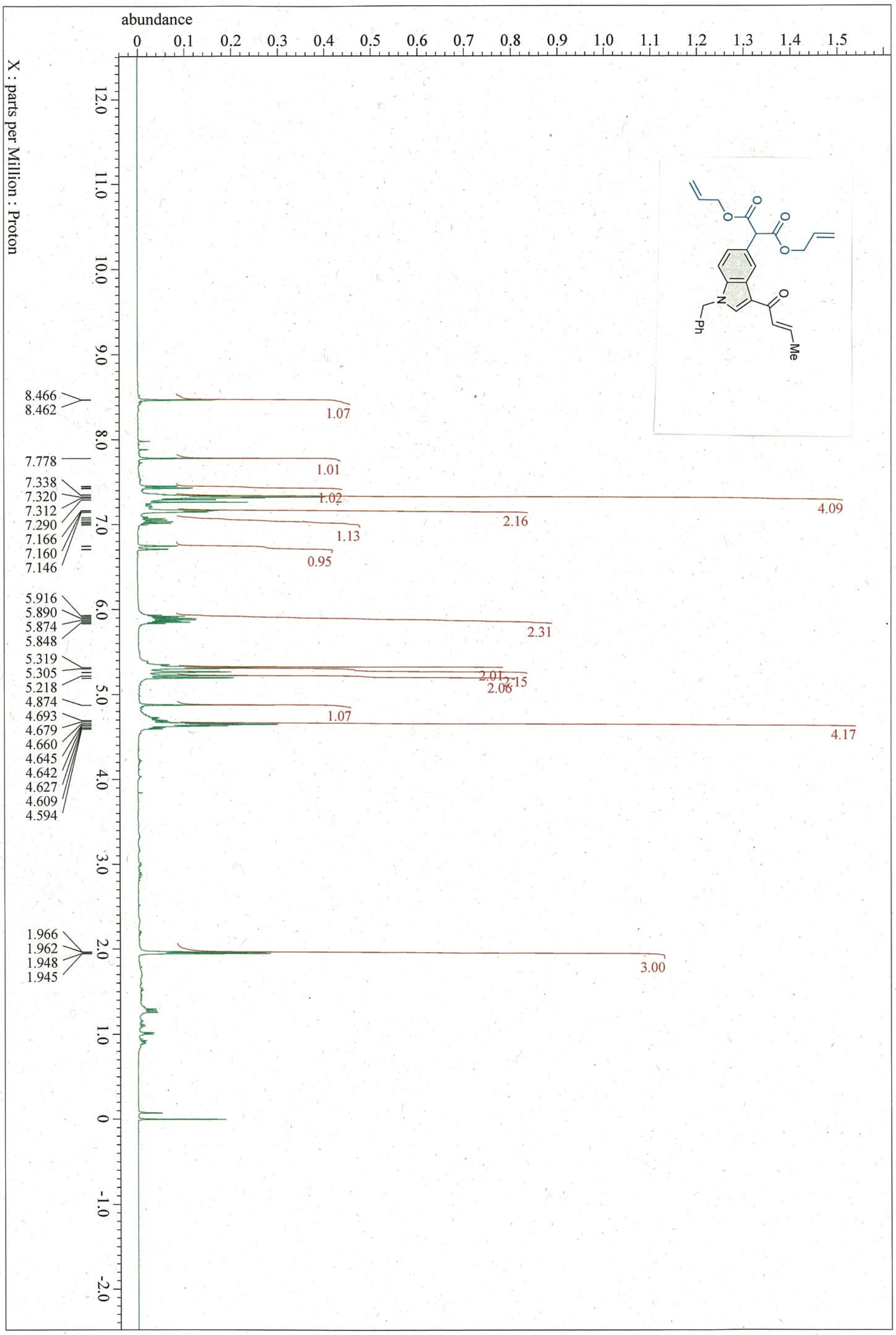
abundance
X : parts per Million : Proton
8.466
8.462
7.778
7.338
7.320
7.312
7.290
7.166
7.160
7.146
5.916
5.890
5.874
5.848
5.319
5.305
5.218
4.874
4.693
4.679
4.660
4.645
4.642
4.627
4.609
4.594
1.966
1.962
1.948
1.945
1.07
1.01
1.02
4.09
2.16
1.13
0.95
2.31
2.01
2.15
2.06
1.07
4.17
3.00
N
O
Ph
Me

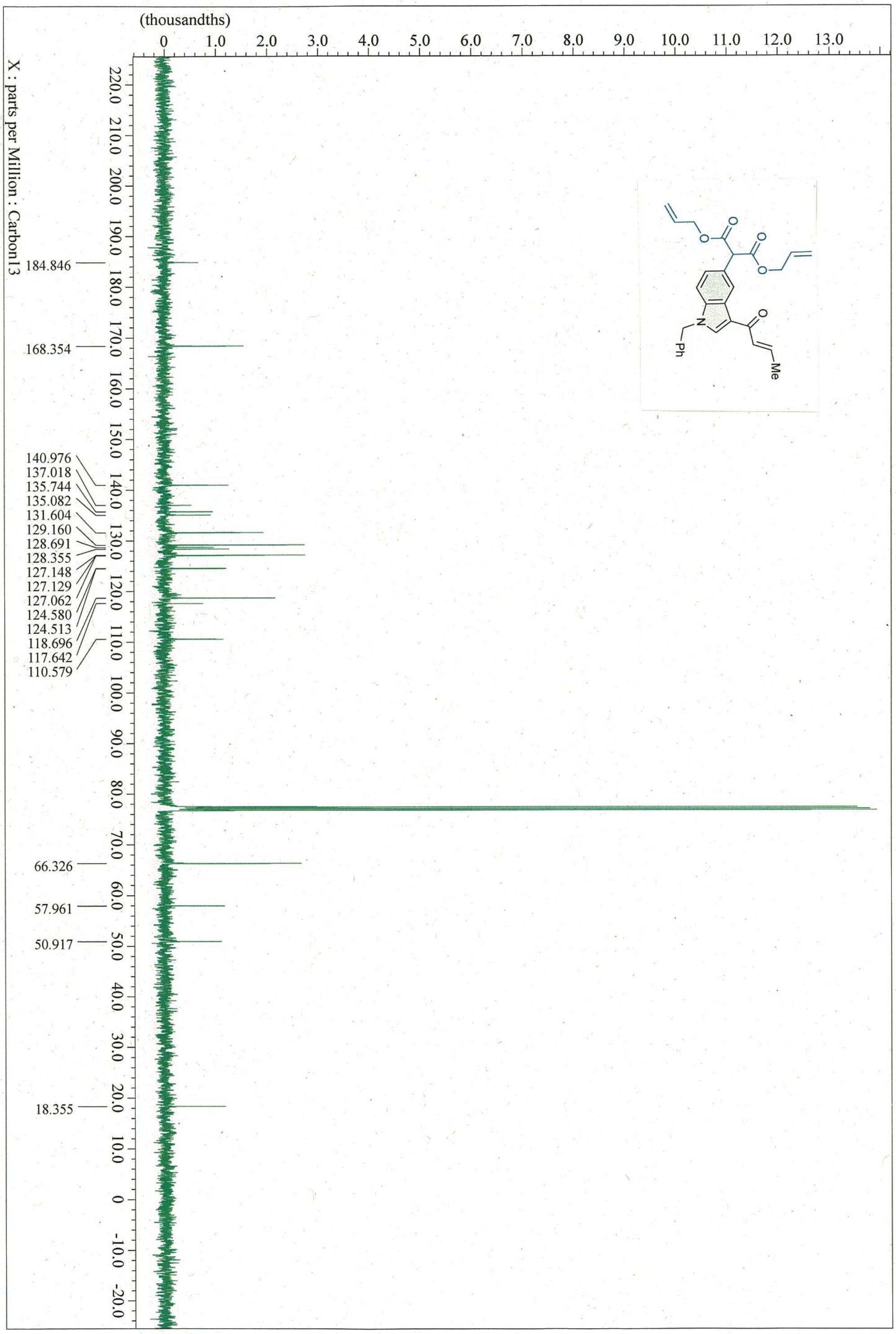

(thousandths)
0 1.0 2.0 3.0 4.0 5.0 6.0 7.0 8.0 9.0 10.0 11.0 12.0 13.0
X : parts per Million : Carbon13
220.0 210.0 200.0 190.0 180.0 170.0 160.0 150.0 140.0 130.0 120.0 110.0 100.0 90.0 80.0 70.0 60.0 50.0 40.0 30.0 20.0 10.0 0 -10.0 -20.0
184.846
168.354
140.976
137.018
135.744
135.082
131.604
129.160
128.691
128.355
127.148
127.129
127.062
124.580
124.513
118.696
117.642
110.579
66.326
57.961
50.917
18.355
O
O
O
O
N
O
Ph
Me

3u

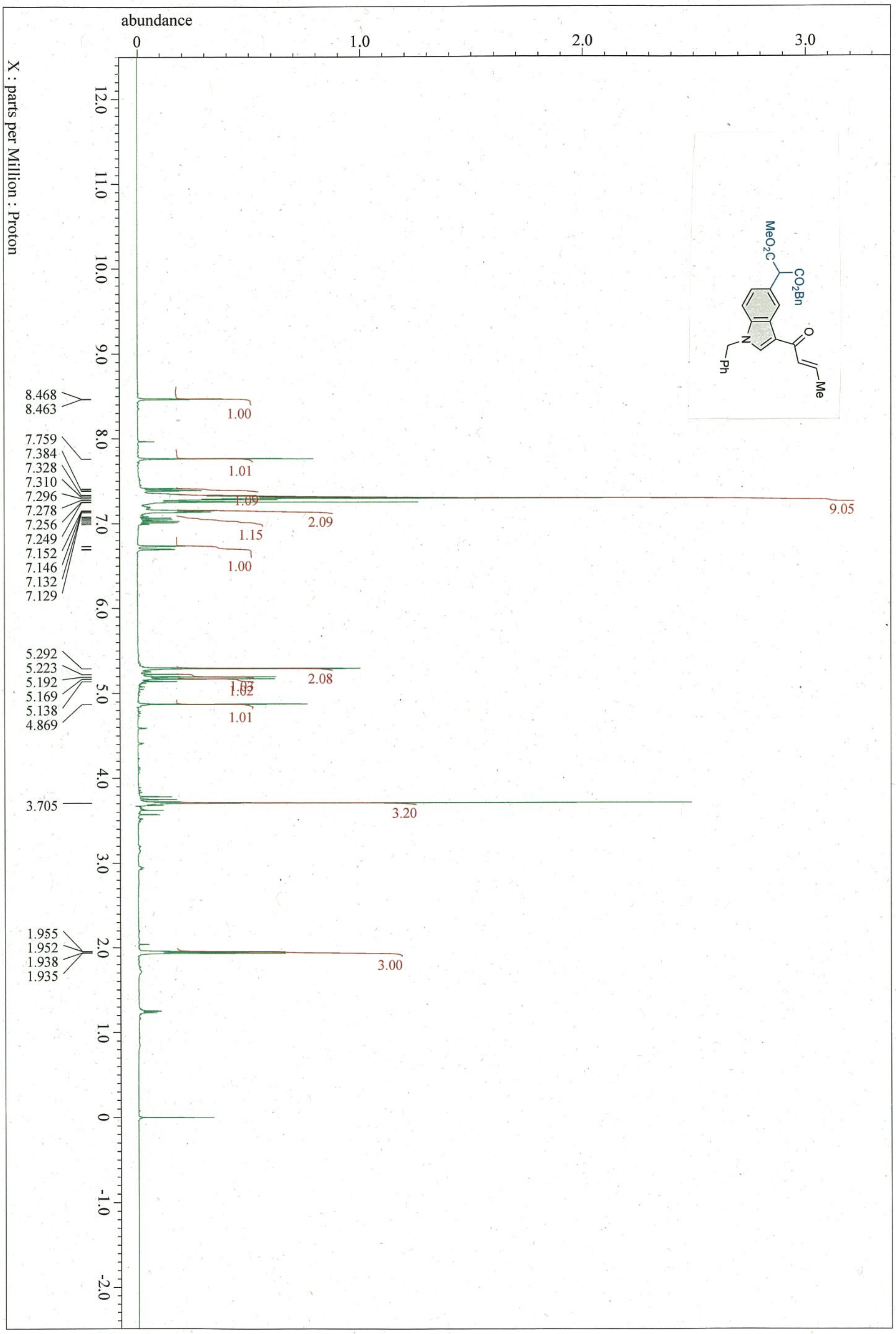
abundance
0
1.0
2.0
3.0
X : parts per Million : Proton
12.0
11.0
10.0
9.0
8.0
7.0
6.0
5.0
4.0
3.0
2.0
1.0
0
-1.0
-2.0
8.468
8.463
7.759
7.384
7.328
7.310
7.296
7.278
7.256
7.249
7.152
7.146
7.132
7.129
5.292
5.223
5.192
5.169
5.138
4.869
3.705
1.955
1.952
1.938
1.935
1.00
1.01
9.05
2.09
1.15
1.00
2.08
1.01
3.20
3.00
MeO2C
CO2Bn
O
N
Ph
Me

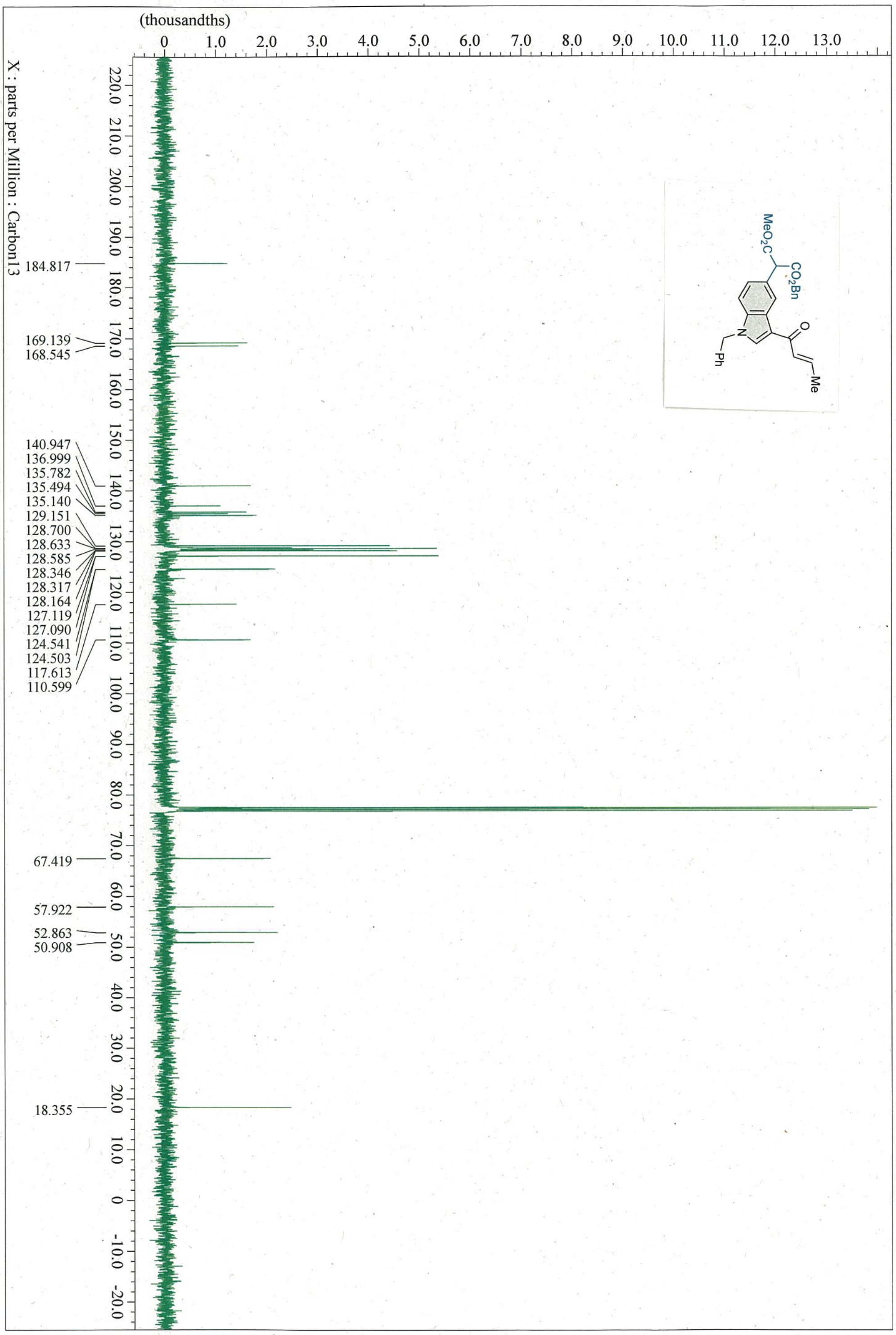
(thousandths)
X : parts per Million : Carbon13
184.817
169.139
168.545
140.947
136.999
135.782
135.494
135.140
129.151
128.700
128.633
128.585
128.346
128.317
128.164
127.119
127.090
124.541
124.503
117.613
110.599
67.419
57.922
52.863
50.908
18.355
MeO2C
CO2Bn
O
N
Ph
Me

3v

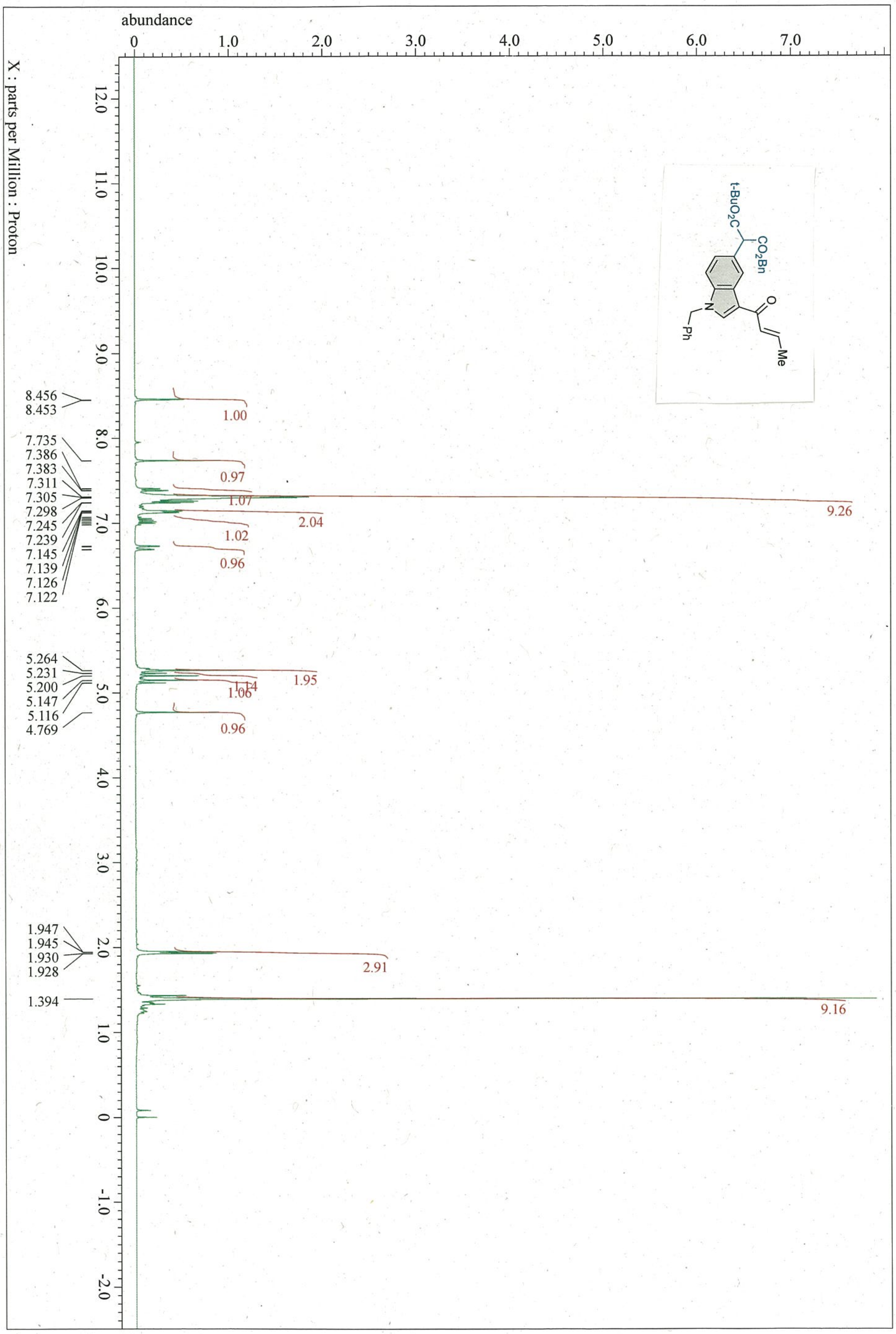

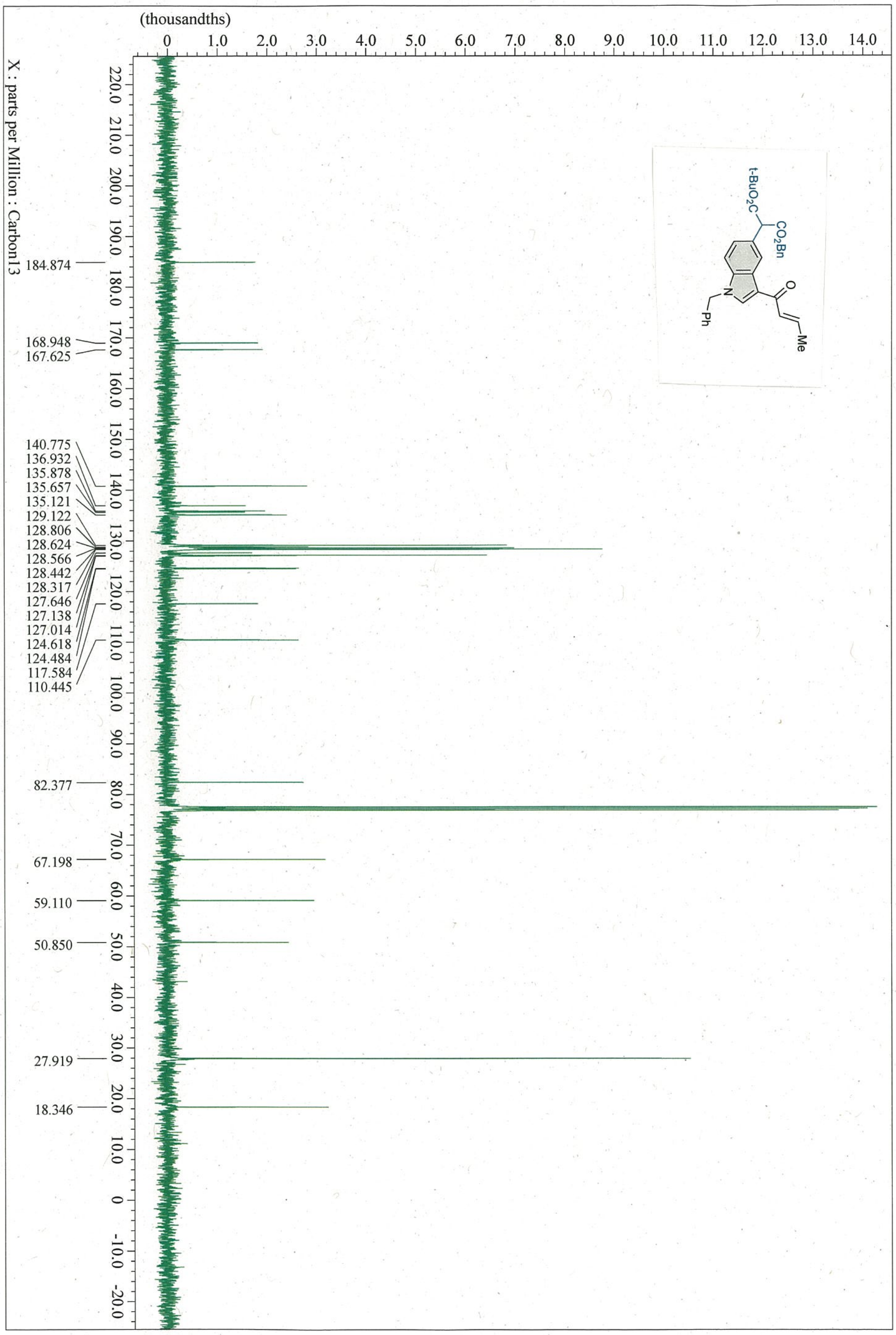
(thousandths)
X : parts per Million : Carbon13
184.874
168.948
167.625
140.775
136.932
135.878
135.657
135.121
129.122
128.806
128.624
128.566
128.442
128.317
127.646
127.138
127.014
124.618
124.484
117.584
110.445
82.377
67.198
59.110
50.850
27.919
18.346
t-BuO2C
CO2Bn
O
N
Ph
Me

3w

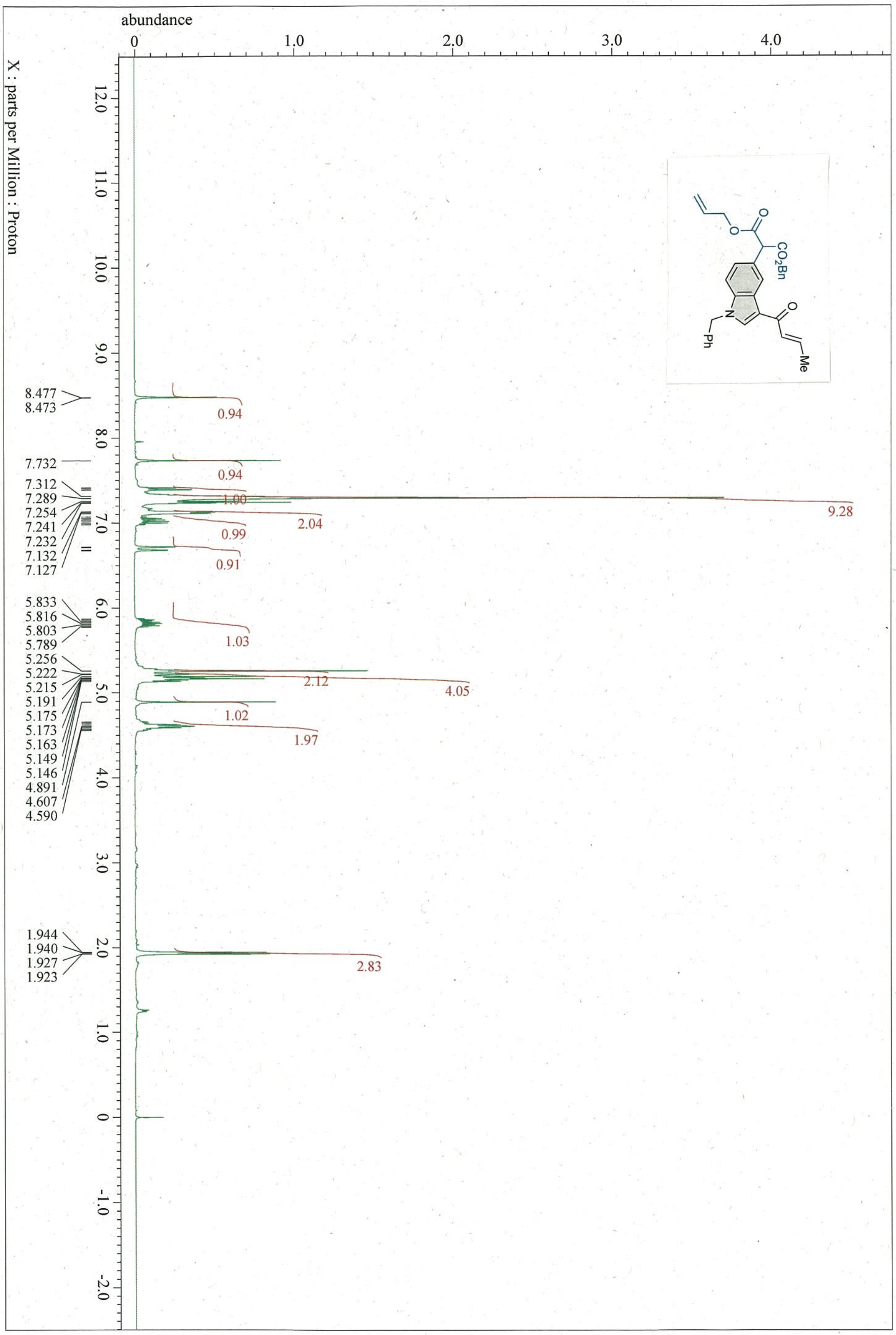
abundance
0
1.0
2.0
3.0
4.0
X : parts per Million : Proton
12.0
11.0
10.0
9.0
8.0
7.0
6.0
5.0
4.0
3.0
2.0
1.0
0
-1.0
-2.0
8.477
8.473
7.732
7.312
7.289
7.254
7.241
7.232
7.132
7.127
5.833
5.816
5.803
5.789
5.256
5.222
5.215
5.191
5.175
5.173
5.163
5.149
5.146
4.891
4.607
4.590
1.944
1.940
1.927
1.923
0.94
0.94
1.00
9.28
2.04
0.99
0.91
1.03
2.12
4.05
1.02
1.97
2.83
O
O
CO_2Bn
O
N
Ph
Me

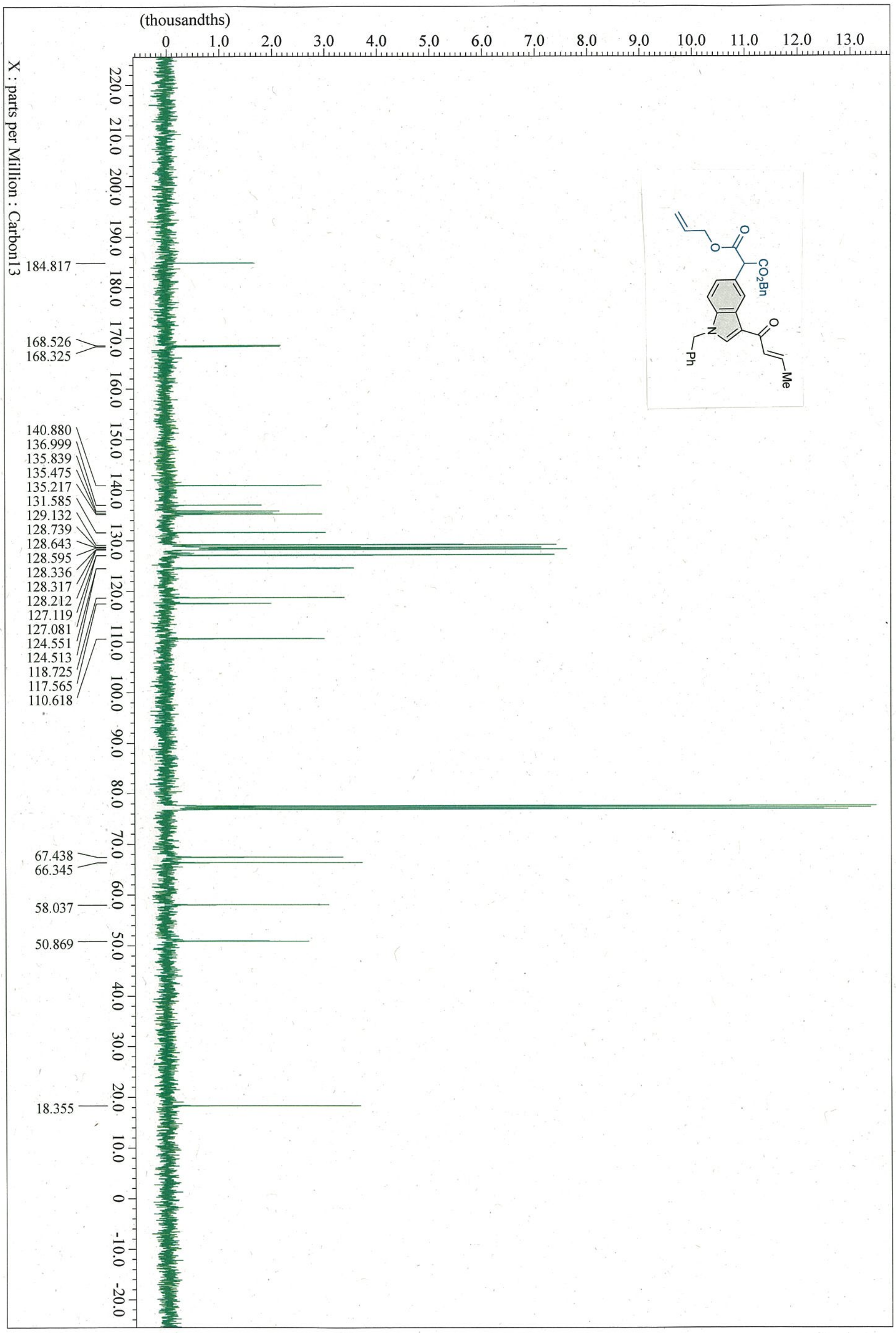

X : parts per Million : Carbon13
(thousandths)
184.817
168.526
168.325
140.880
136.999
135.839
135.475
135.217
131.585
129.132
128.739
128.643
128.595
128.336
128.317
128.212
127.119
127.081
124.551
124.513
118.725
117.565
110.618
67.438
66.345
58.037
50.869
18.355
CO2Bn
O
N
Ph
Me

3x

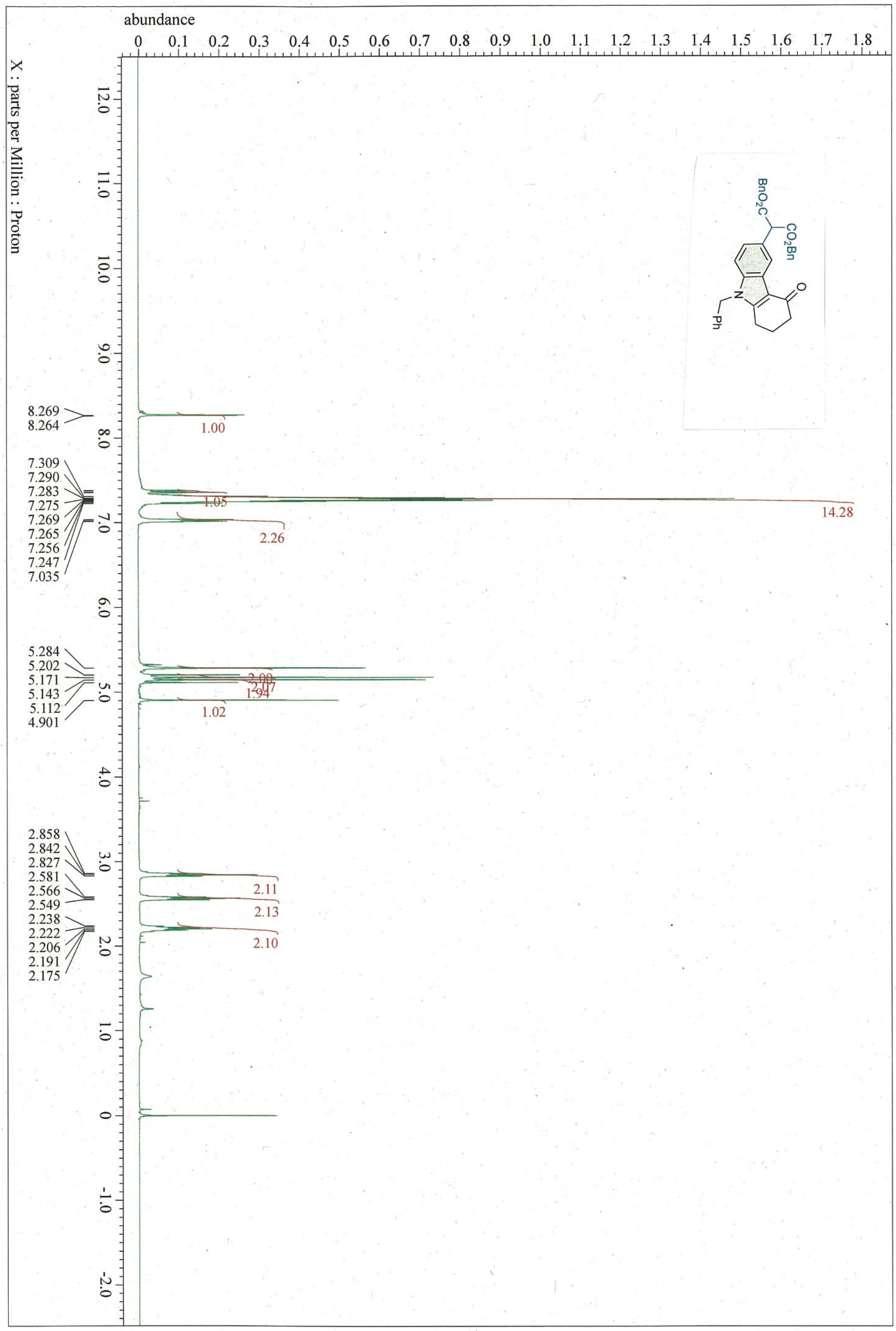

abundance
X : parts per Million : Proton
8.269
8.264
7.309
7.290
7.283
7.275
7.269
7.265
7.256
7.247
7.035
5.284
5.202
5.171
5.143
5.112
4.901
2.858
2.842
2.827
2.581
2.566
2.549
2.238
2.222
2.206
2.191
2.175
1.00
1.05
14.28
2.26
1.94
1.02
2.11
2.13
2.10
BnO2C
CO2Bn
N
O
Ph

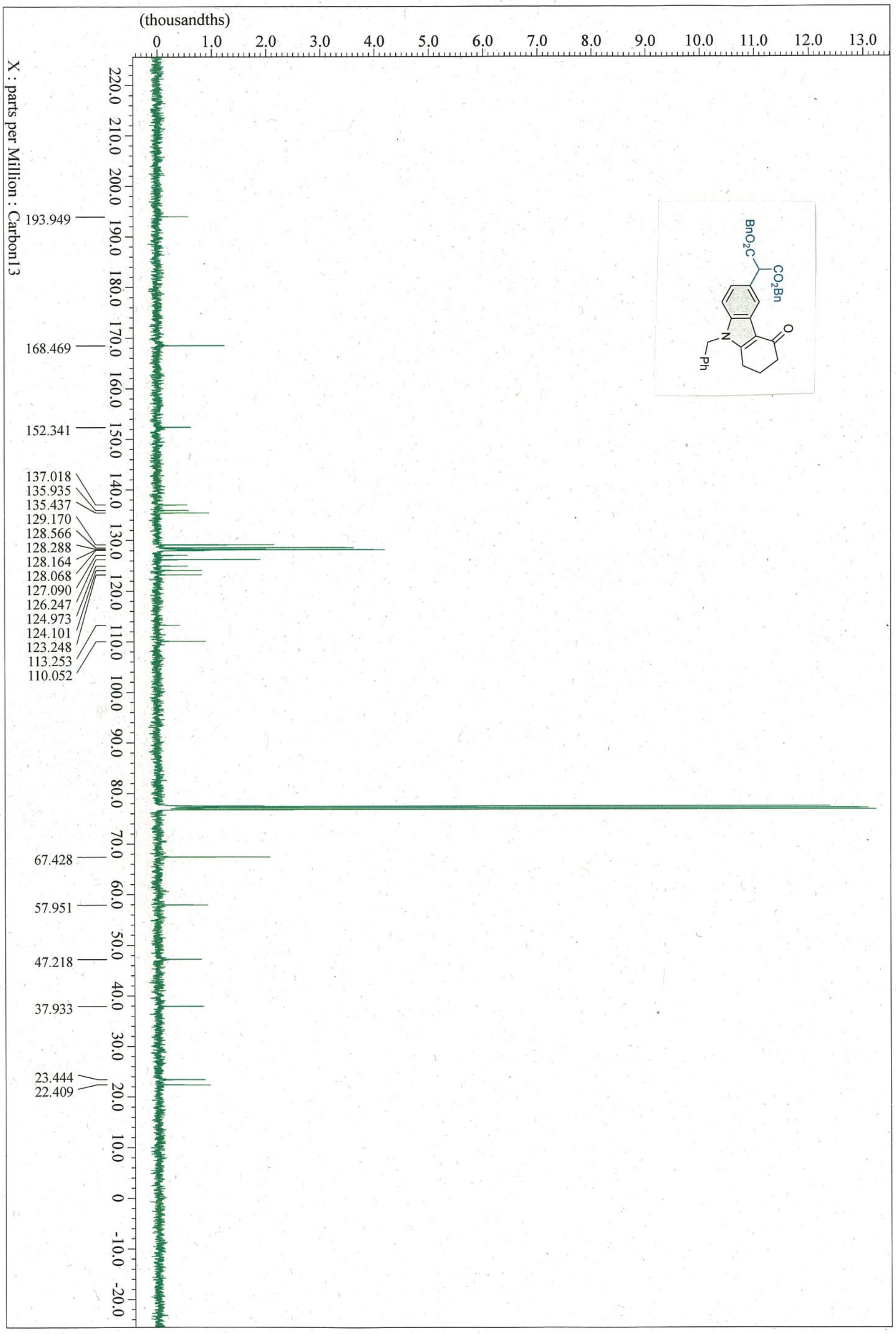

(thousandths)
X : parts per Million : Carbon13
193.949
168.469
152.341
137.018
135.935
135.437
129.170
128.566
128.288
128.164
128.068
127.090
126.247
124.973
124.101
123.248
113.253
110.052
67.428
57.951
47.218
37.933
23.444
22.409
BnO2C
CO2Bn
O
N
Ph

5y

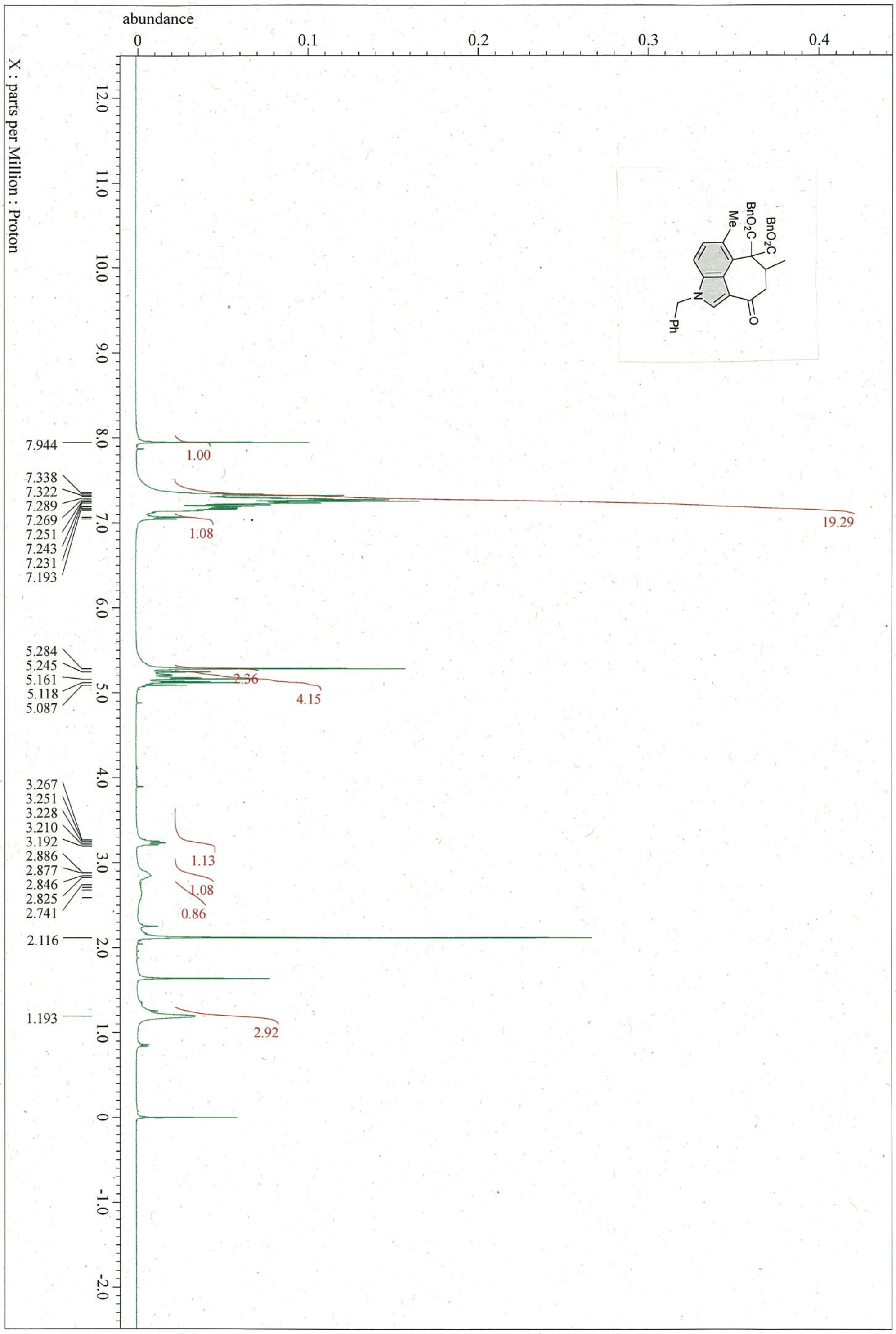
abundance
0
0.1
0.2
0.3
0.4
X : parts per Million : Proton
12.0
11.0
10.0
9.0
8.0
7.0
6.0
5.0
4.0
3.0
2.0
1.0
0
-1.0
-2.0
7.944
7.338
7.322
7.289
7.269
7.251
7.243
7.231
7.193
5.284
5.245
5.161
5.118
5.087
3.267
3.251
3.228
3.210
3.192
2.886
2.877
2.846
2.825
2.741
2.116
1.193
1.00
19.29
1.08
2.36
4.15
1.13
1.08
0.86
2.92
BnO2C
BnO2C
Me
N
Ph
O

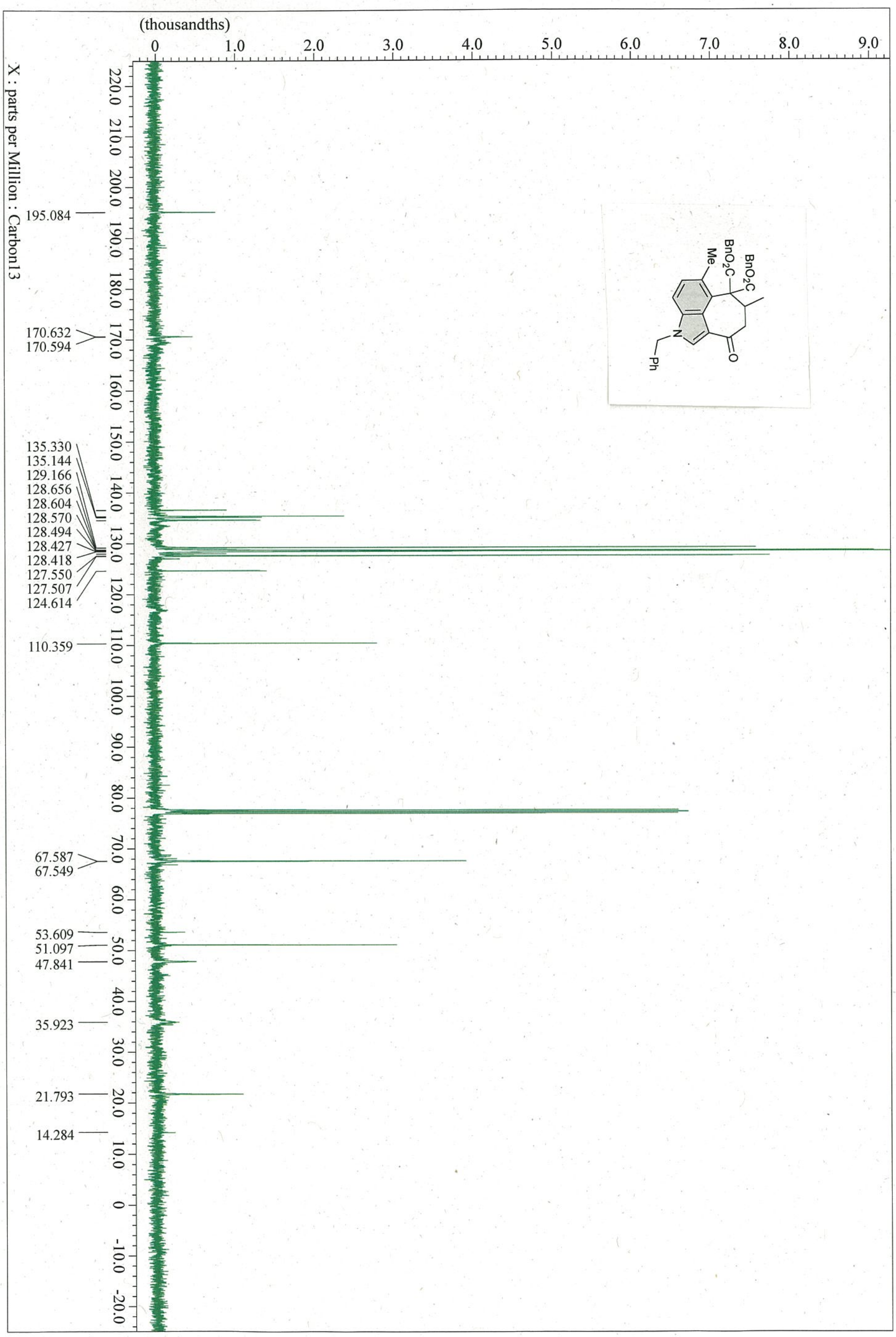

X : parts per Million : Carbon13
(thousandths)
195.084
170.632
170.594
135.330
135.144
129.166
128.656
128.604
128.570
128.494
128.427
128.418
127.550
127.507
124.614
110.359
67.587
67.549
53.609
51.097
47.841
35.923
21.793
14.284
BnO2C
BnO2C
Me
N
Ph
O

6

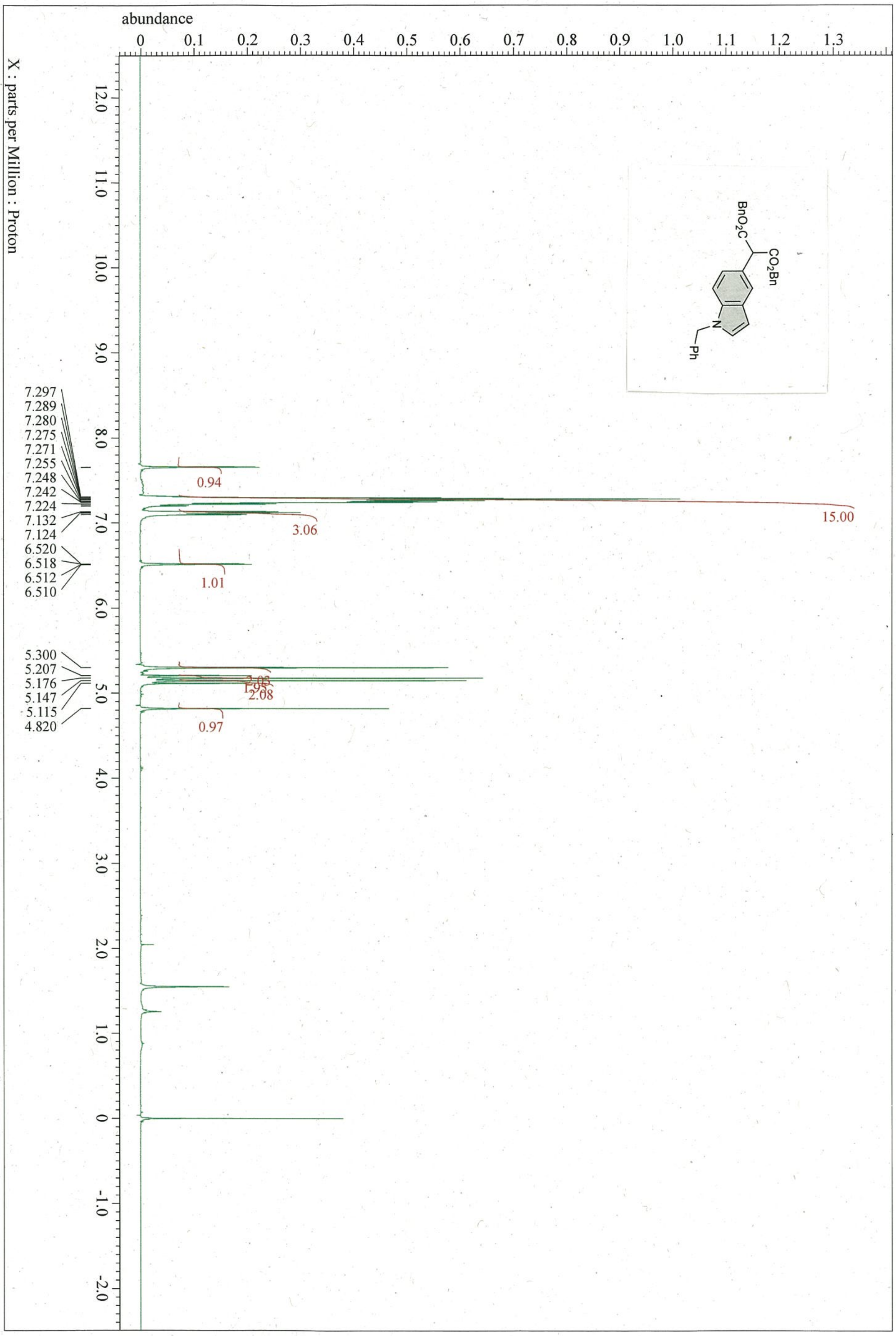

abundance
X : parts per Million : Proton
7.297
7.289
7.280
7.275
7.271
7.255
7.248
7.242
7.224
7.132
7.124
6.520
6.518
6.512
6.510
5.300
5.207
5.176
5.147
5.115
4.820
0.94
15.00
3.06
1.01
0.97
BnO2C
CO2Bn
N
Ph

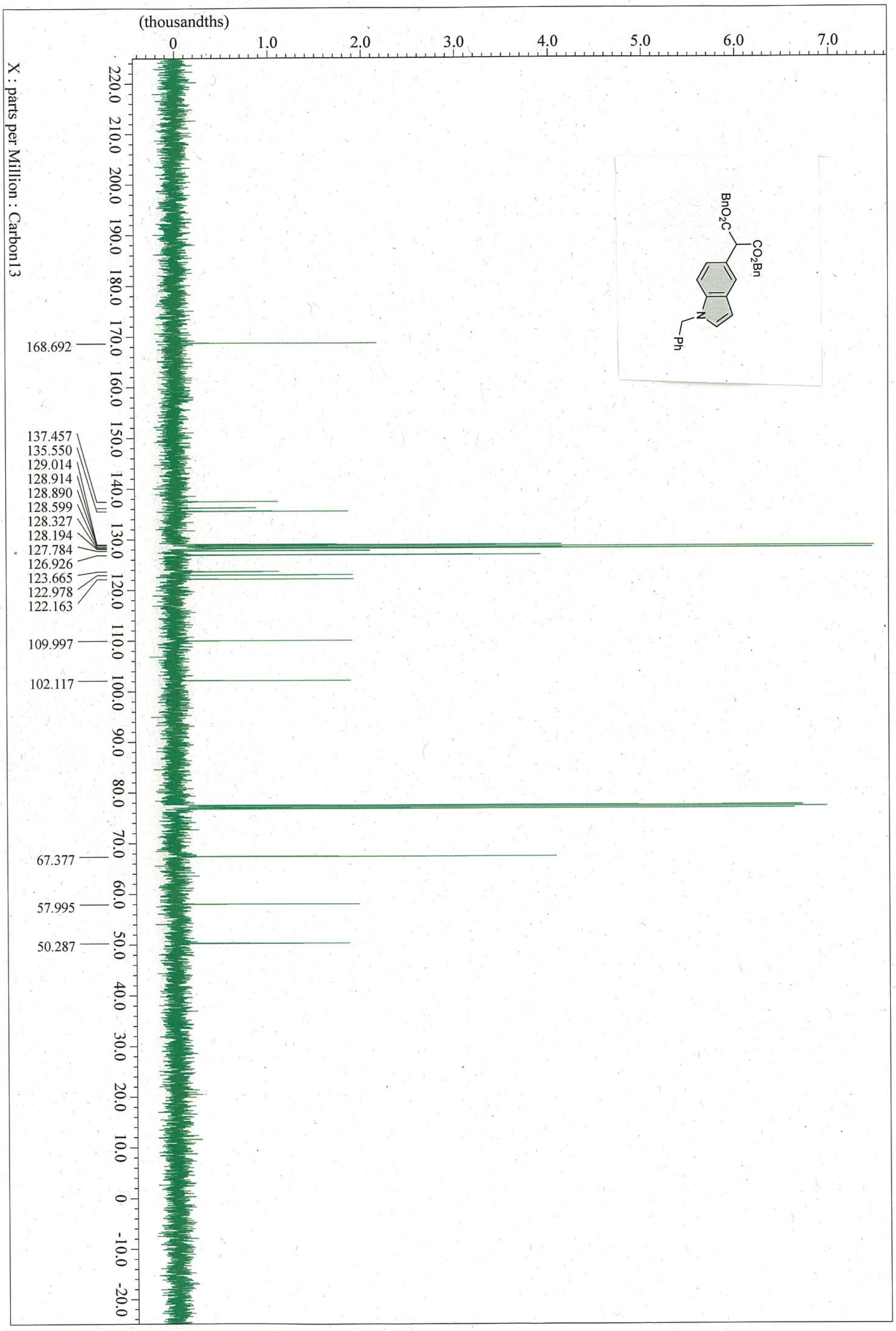
(thousandths)
X : parts per Million : Carbon13
168.692
137.457
135.550
129.014
128.914
128.890
128.599
128.327
128.194
127.784
126.926
123.665
122.978
122.163
109.997
102.117
67.377
57.995
50.287
BnO2C
CO2Bn
N
Ph

7

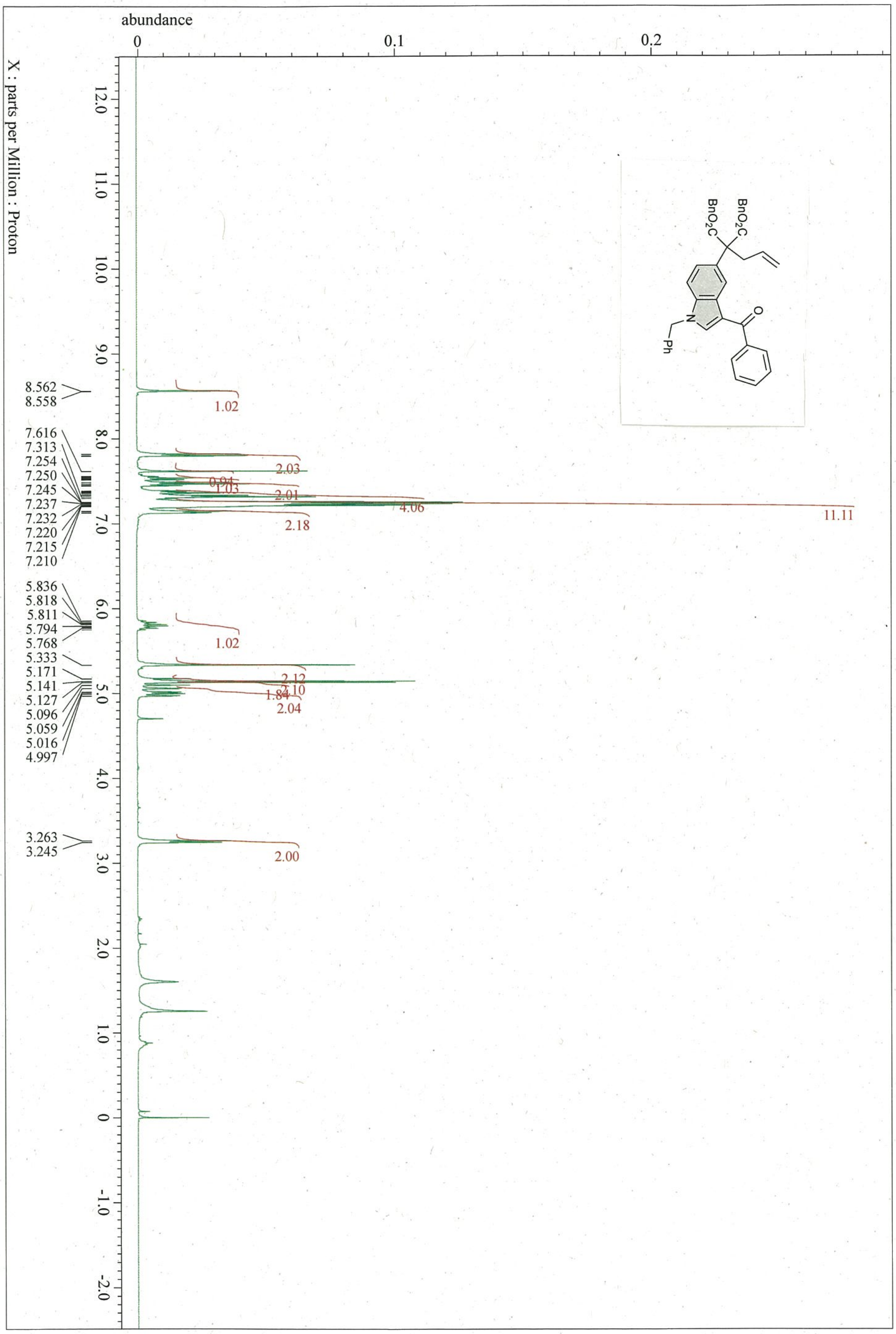
abundance
0
0.1
0.2
X : parts per Million : Proton
12.0
11.0
10.0
9.0
8.0
7.0
6.0
5.0
4.0
3.0
2.0
1.0
0
-1.0
-2.0
8.562
8.558
7.616
7.313
7.254
7.250
7.245
7.237
7.232
7.220
7.215
7.210
5.836
5.818
5.811
5.794
5.768
5.333
5.171
5.141
5.127
5.096
5.059
5.016
4.997
3.263
3.245
1.02
2.03
0.94
1.03
2.01
4.06
11.11
2.18
1.02
2.12
2.10
1.84
2.04
2.00
BnO2C
BnO2C
N
Ph
O

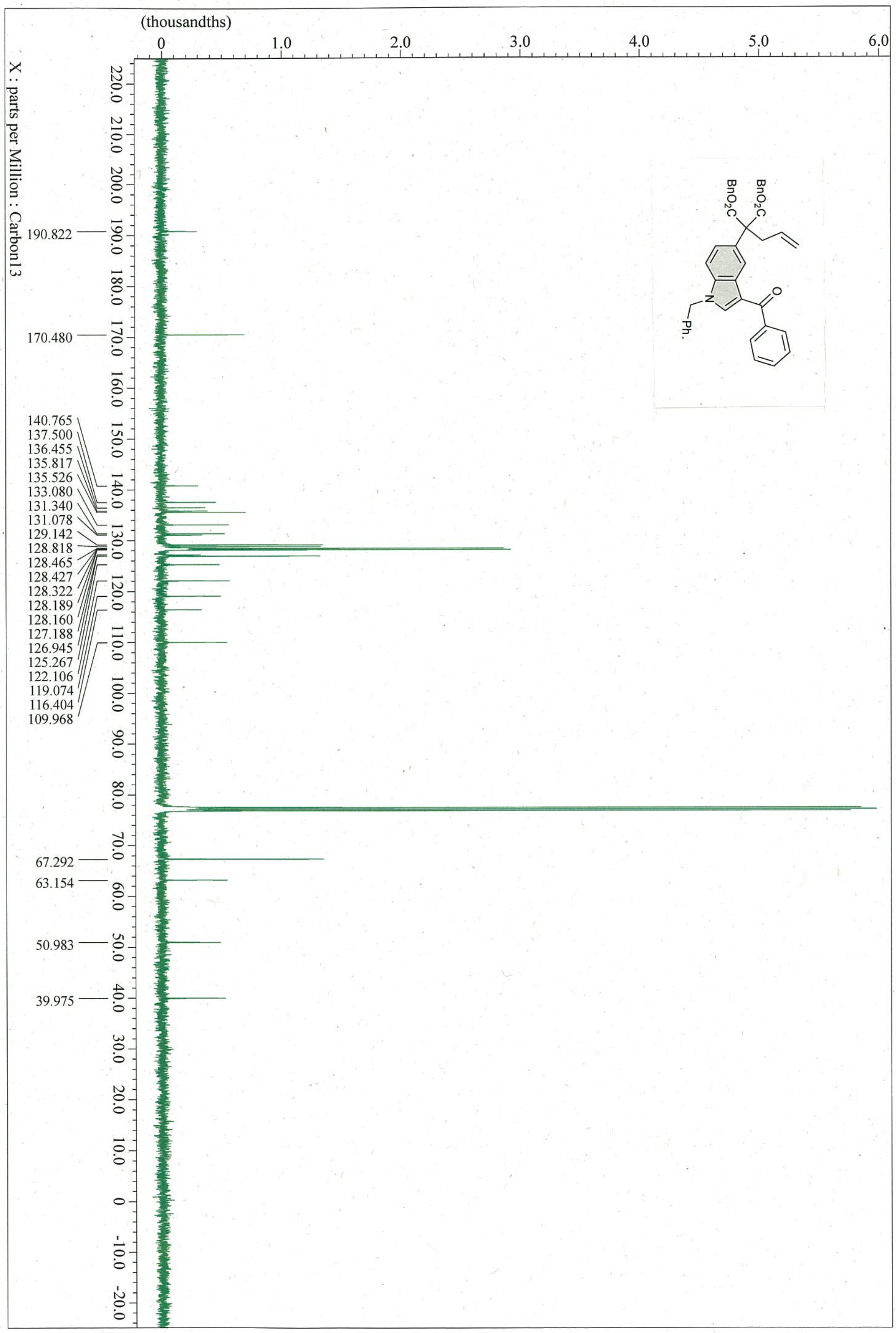

(thousandths)
X : parts per Million : Carbon13
190.822
170.480
140.765
137.500
136.455
135.817
135.526
133.080
131.340
131.078
129.142
128.818
128.465
128.427
128.322
128.189
128.160
127.188
126.945
125.267
122.106
119.074
116.404
109.968
67.292
63.154
50.983
39.975
BnO2C
BnO2C
N
O
Ph

8

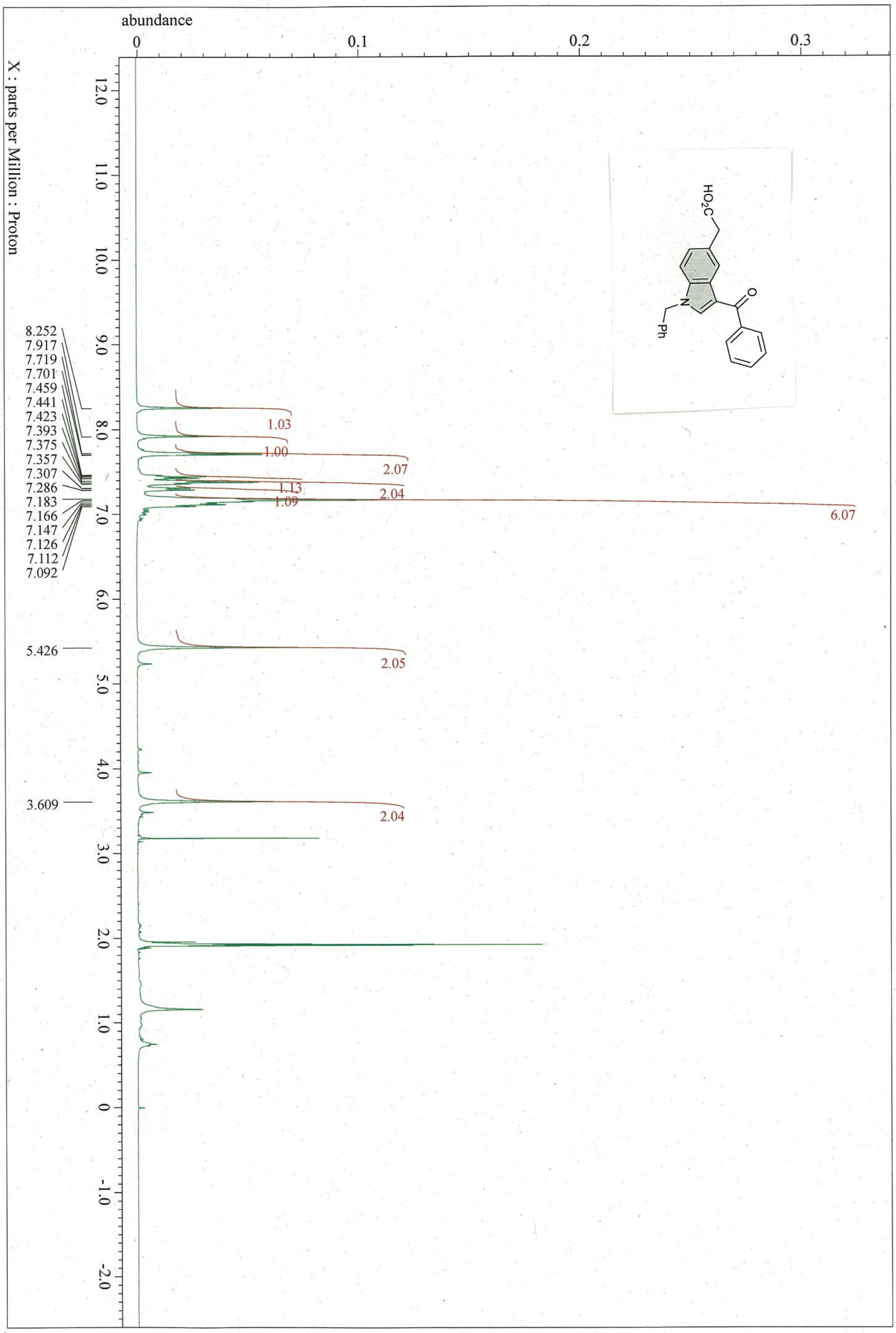
abundance
X : parts per Million : Proton
8.252
7.917
7.719
7.701
7.459
7.441
7.423
7.393
7.375
7.357
7.307
7.286
7.183
7.166
7.147
7.126
7.112
7.092
5.426
3.609
1.03
1.00
2.07
1.13
2.04
1.09
6.07
2.05
2.04
HO2C
N
Ph
O

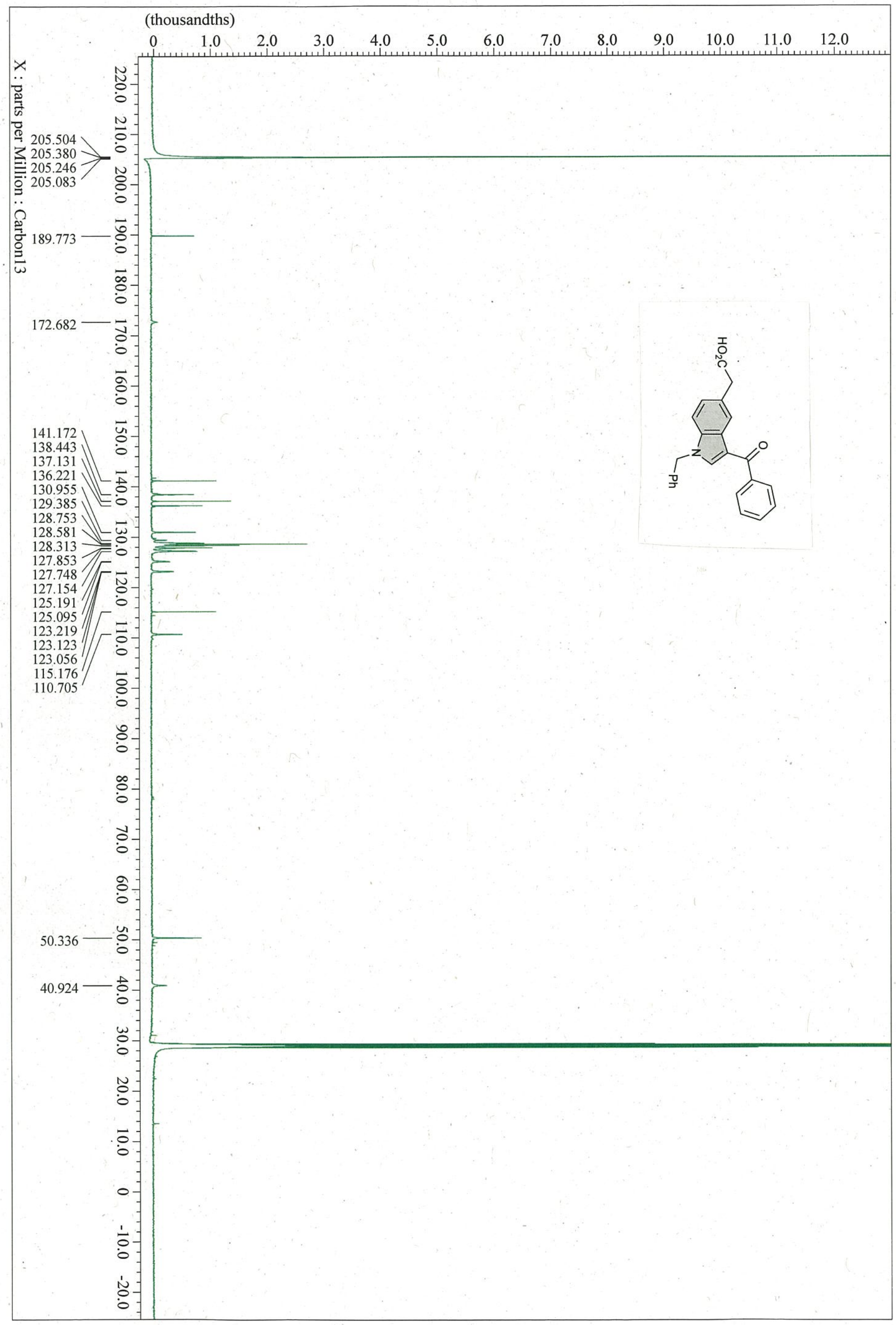
(thousandths)
X : parts per Million : Carbon13
205.504
205.380
205.246
205.083
189.773
172.682
141.172
138.443
137.131
136.221
130.955
129.385
128.753
128.581
128.313
127.853
127.748
127.154
125.191
125.095
123.219
123.123
123.056
115.176
110.705
50.336
40.924
HO2C
N
Ph
O

1e

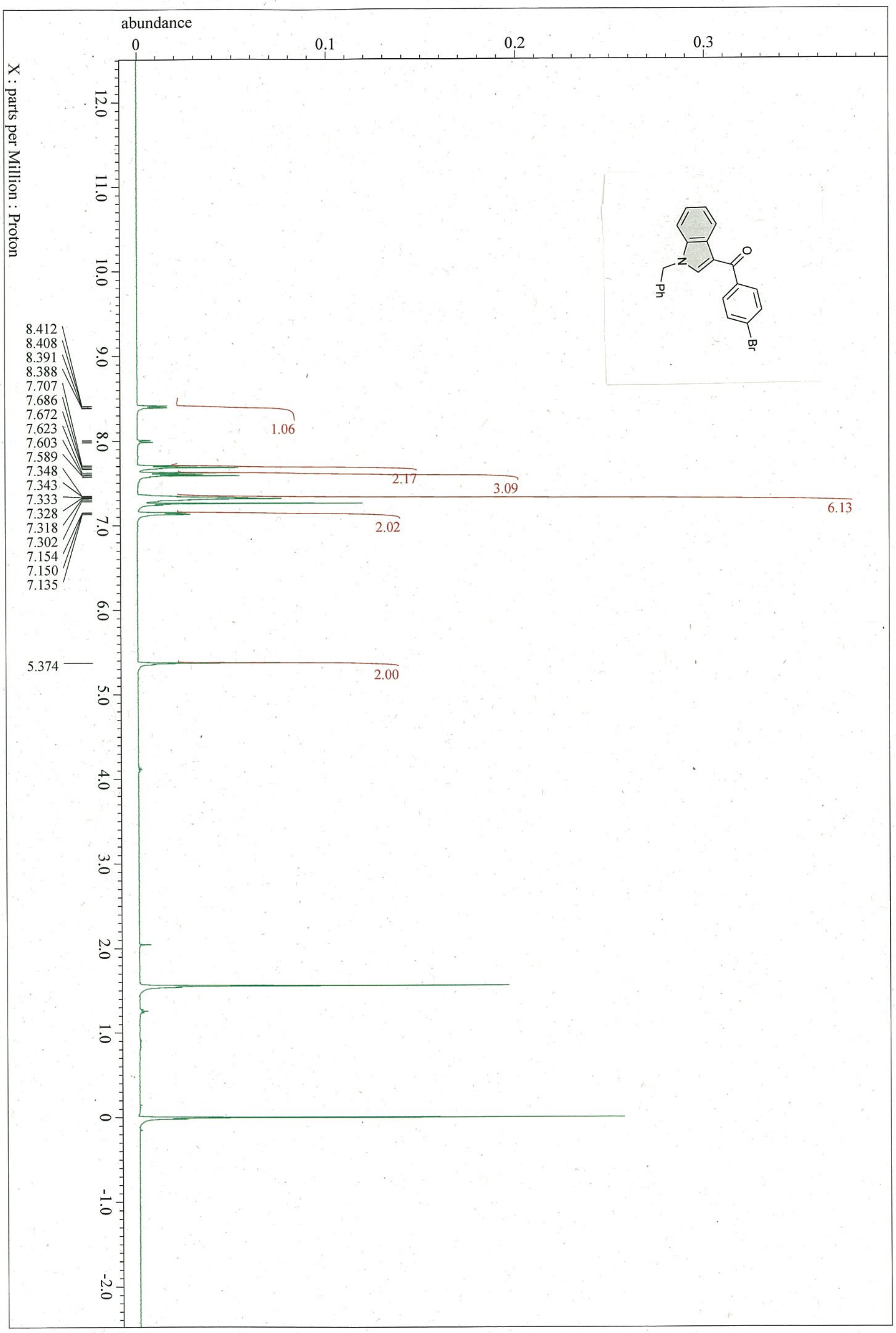
abundance
X : parts per Million : Proton
8.412
8.408
8.391
8.388
7.707
7.686
7.672
7.623
7.603
7.589
7.348
7.343
7.333
7.328
7.318
7.302
7.154
7.150
7.135
5.374
1.06
2.17
3.09
6.13
2.02
2.00
Ph
N
O
Br

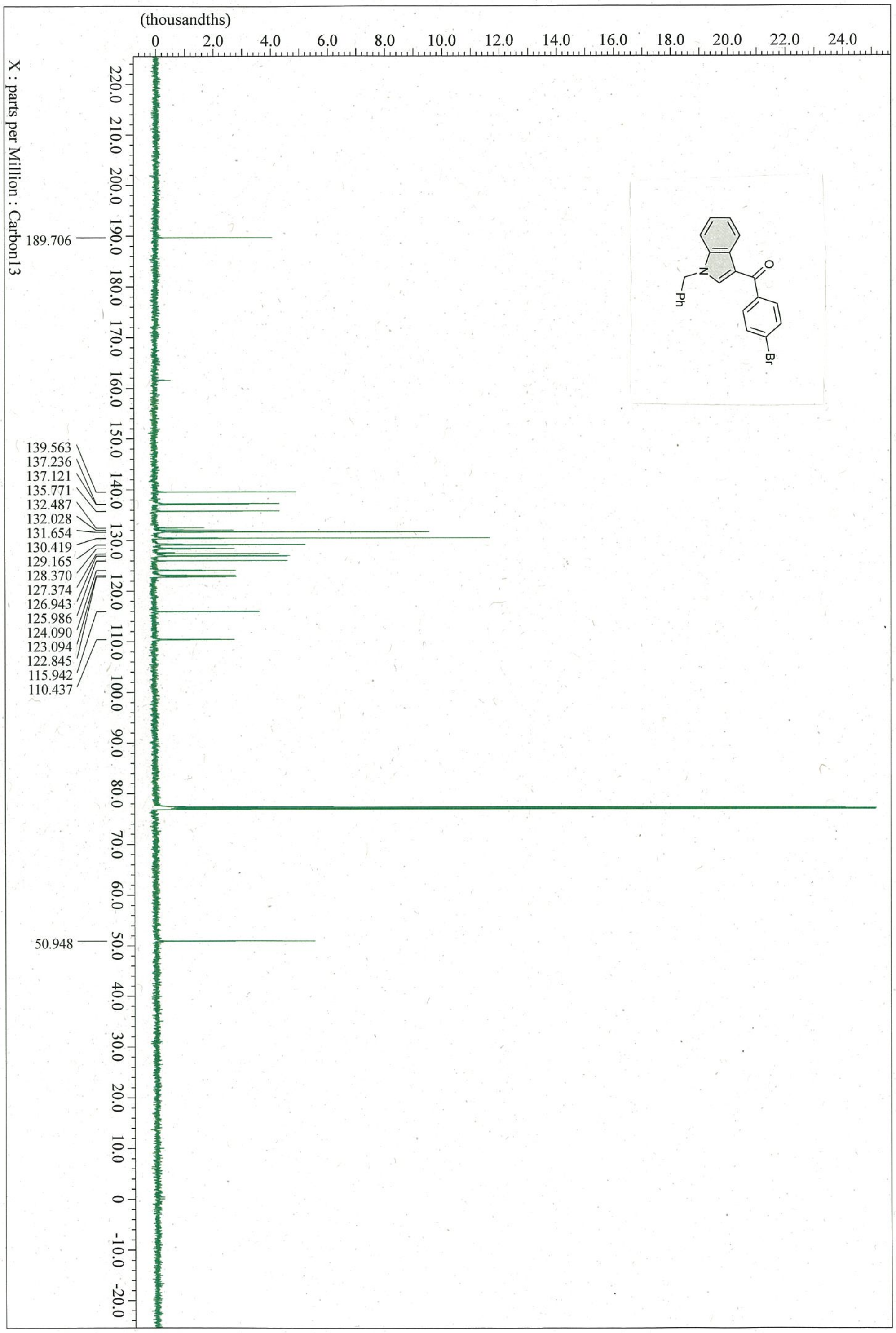
(thousandths)
X : parts per Million : Carbon13
189.706
139.563
137.236
137.121
135.771
132.487
132.028
131.654
130.419
129.165
128.370
127.374
126.943
125.986
124.090
123.094
122.845
115.942
110.437
50.948
N
O
Ph
Br

1f

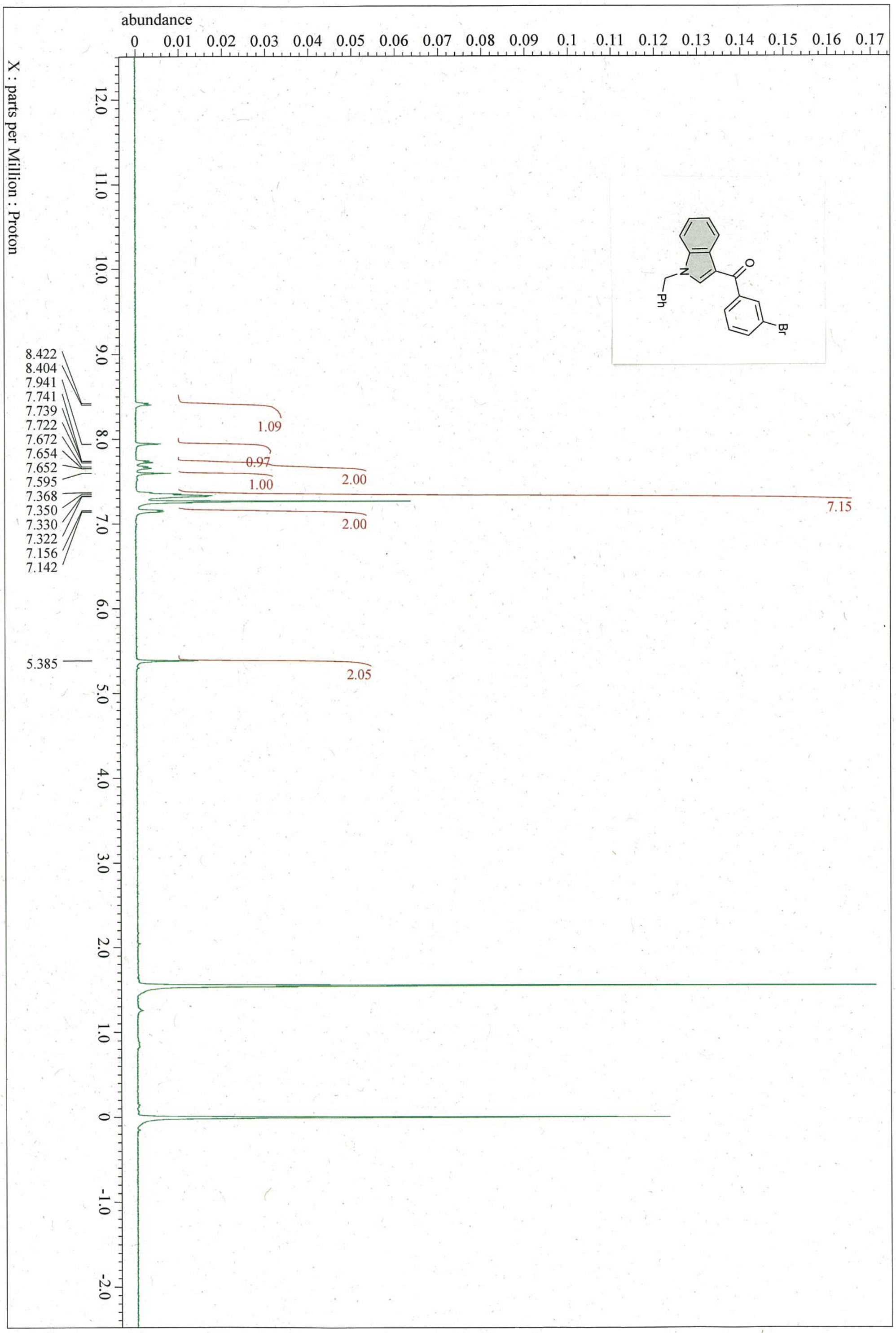
abundance
X : parts per Million : Proton
8.422
8.404
7.941
7.741
7.739
7.722
7.672
7.654
7.652
7.595
7.368
7.350
7.330
7.322
7.156
7.142
5.385
1.09
0.97
1.00
2.00
7.15
2.00
2.05
Ph
Br

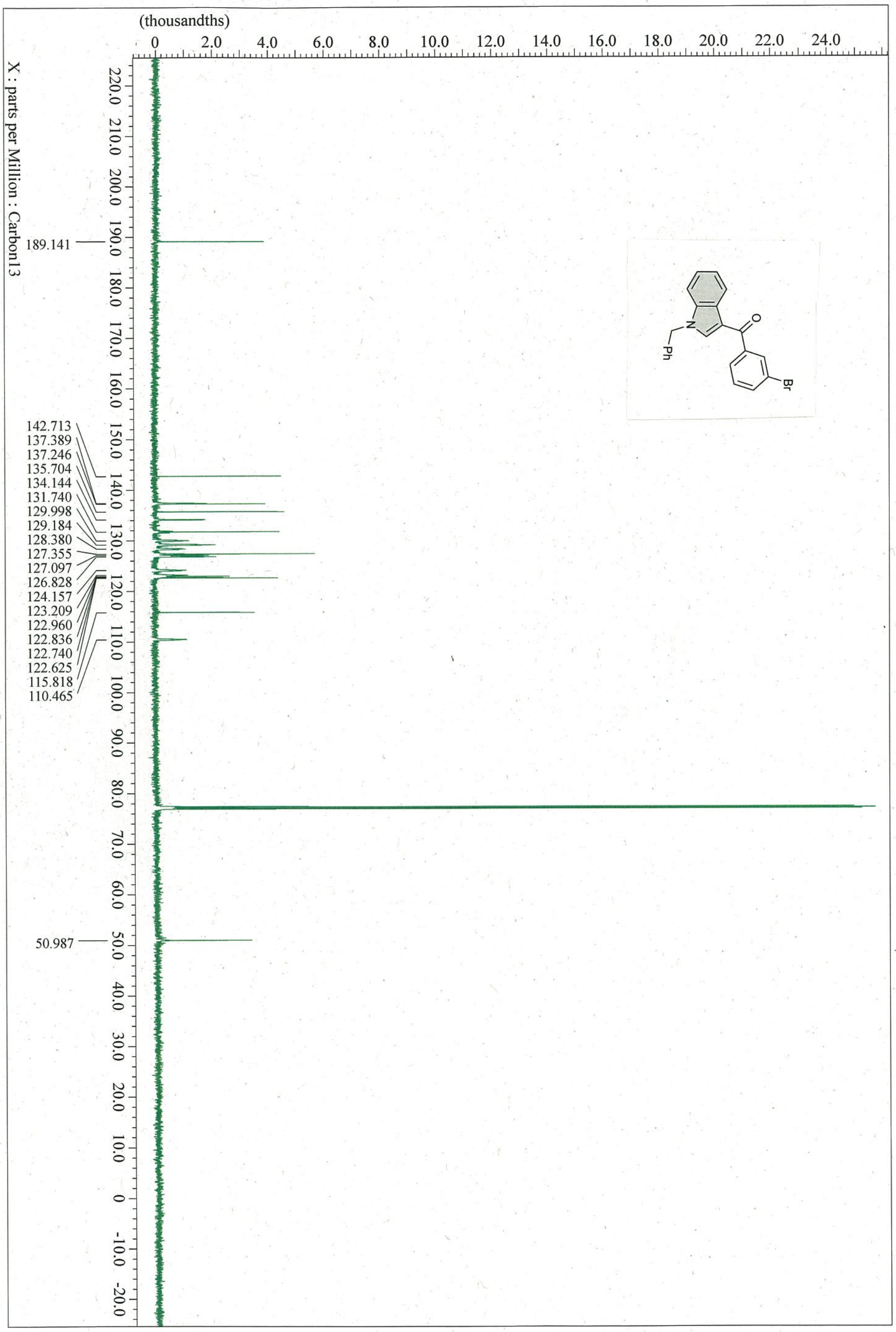

(thousandths)
X : parts per Million : Carbon13
189.141
142.713
137.389
137.246
135.704
134.144
131.740
129.998
129.184
128.380
127.355
127.097
126.828
124.157
123.209
122.960
122.836
122.740
122.625
115.818
110.465
50.987
Ph
Br

1i

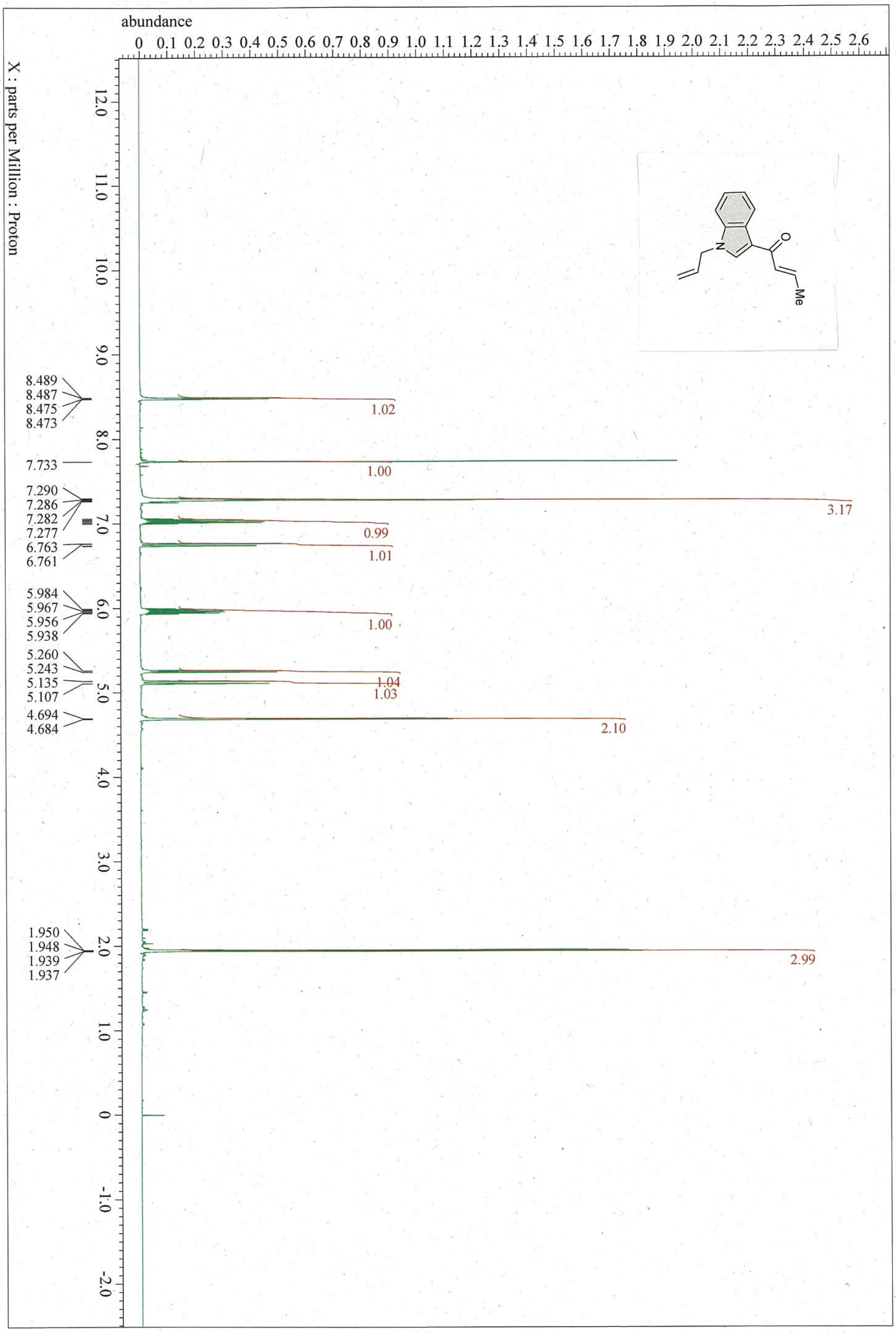

X : parts per Million : Proton
abundance
8.489
8.487
8.475
8.473
7.733
7.290
7.286
7.282
7.277
6.763
6.761
5.984
5.967
5.956
5.938
5.260
5.243
5.135
5.107
4.694
4.684
1.950
1.948
1.939
1.937
1.02
1.00
3.17
0.99
1.01
1.00
1.04
1.03
2.10
2.99
O
N
Me

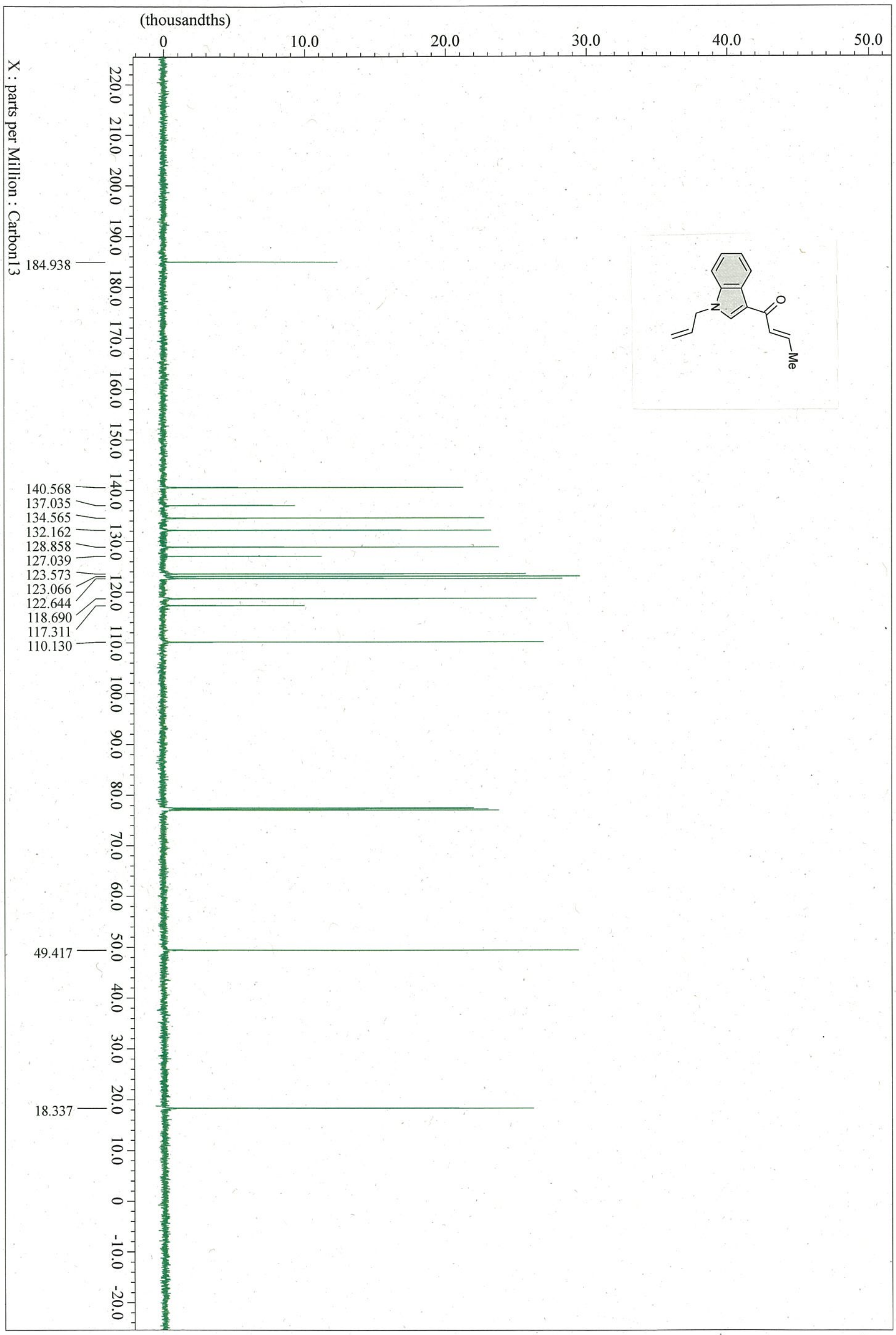

(thousandths)
X : parts per Million : Carbon13
184.938
140.568
137.035
134.565
132.162
128.858
127.039
123.573
123.066
122.644
118.690
117.311
110.130
49.417
18.337
N
O
Me

1m

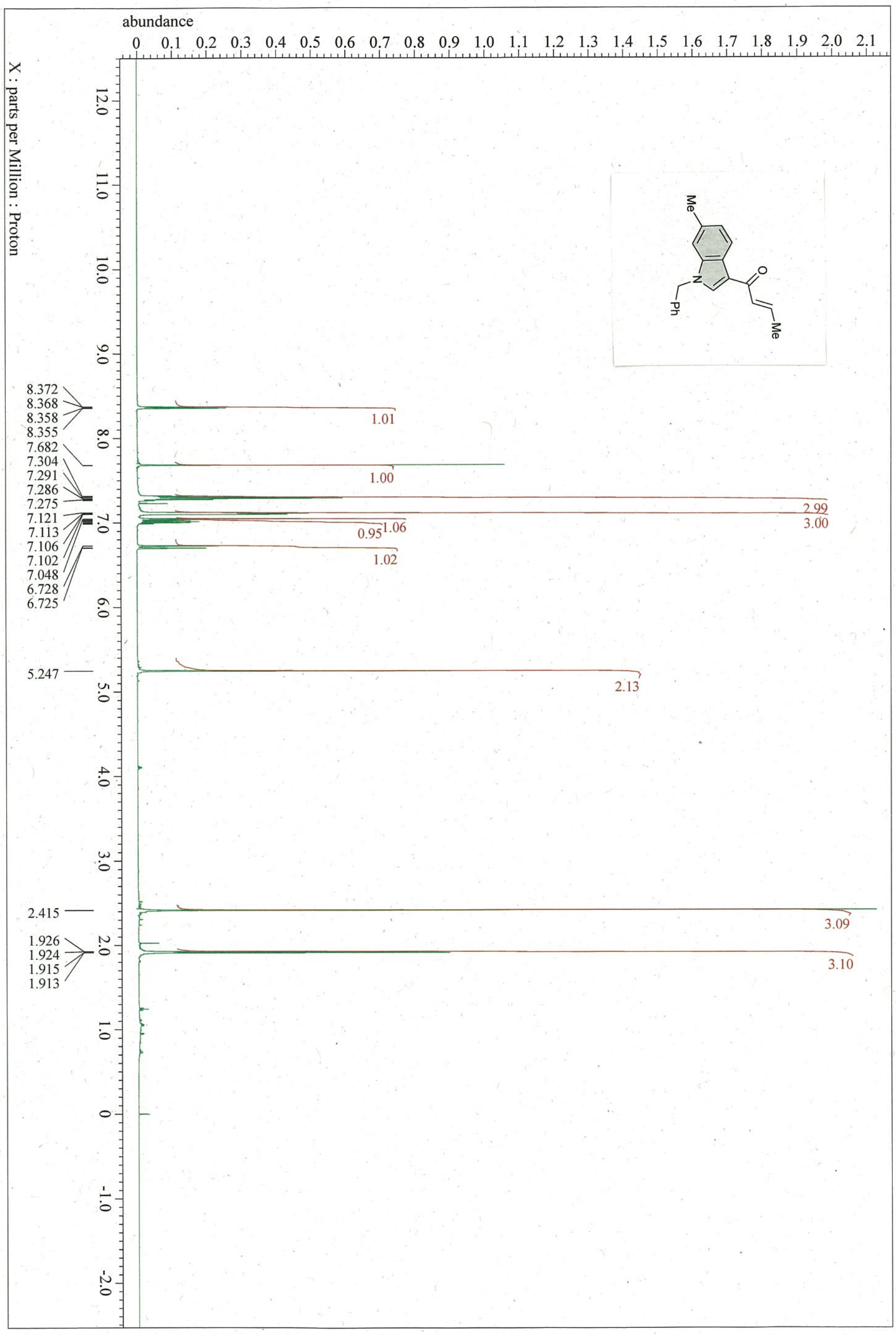

abundance
X : parts per Million : Proton
8.372
8.368
8.358
8.355
7.682
7.304
7.291
7.286
7.275
7.121
7.113
7.106
7.102
7.048
6.728
6.725
5.247
2.415
1.926
1.924
1.915
1.913
1.01
1.00
2.99
3.00
0.95
1.06
1.02
2.13
3.09
3.10
Me
N
O
Ph
Me

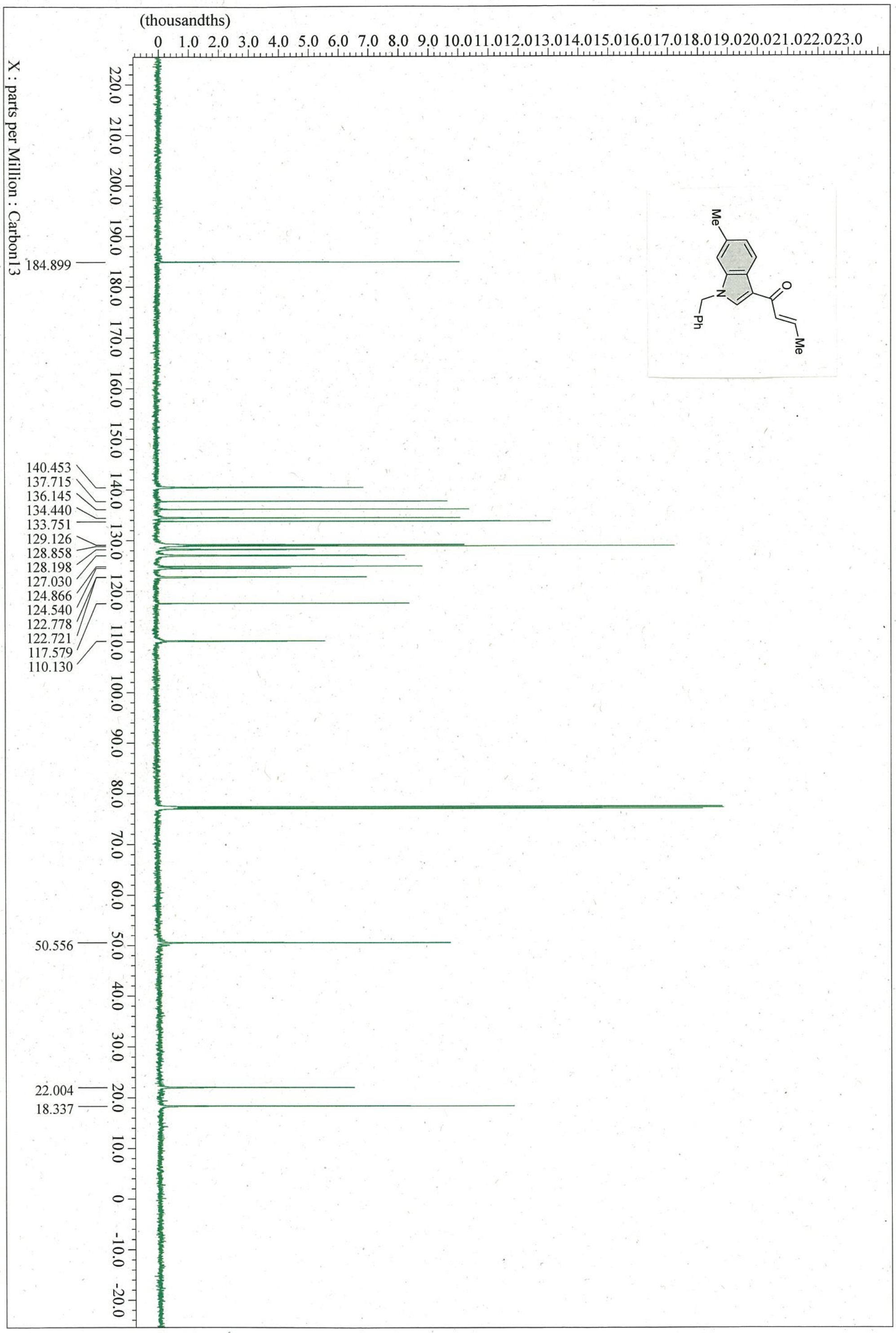
X : parts per Million : Carbon13
(thousandths)
184.899
140.453
137.715
136.145
134.440
133.751
129.126
128.858
128.198
127.030
124.866
124.540
122.778
122.721
117.579
110.130
50.556
22.004
18.337
Me
N
O
Ph
Me

1n

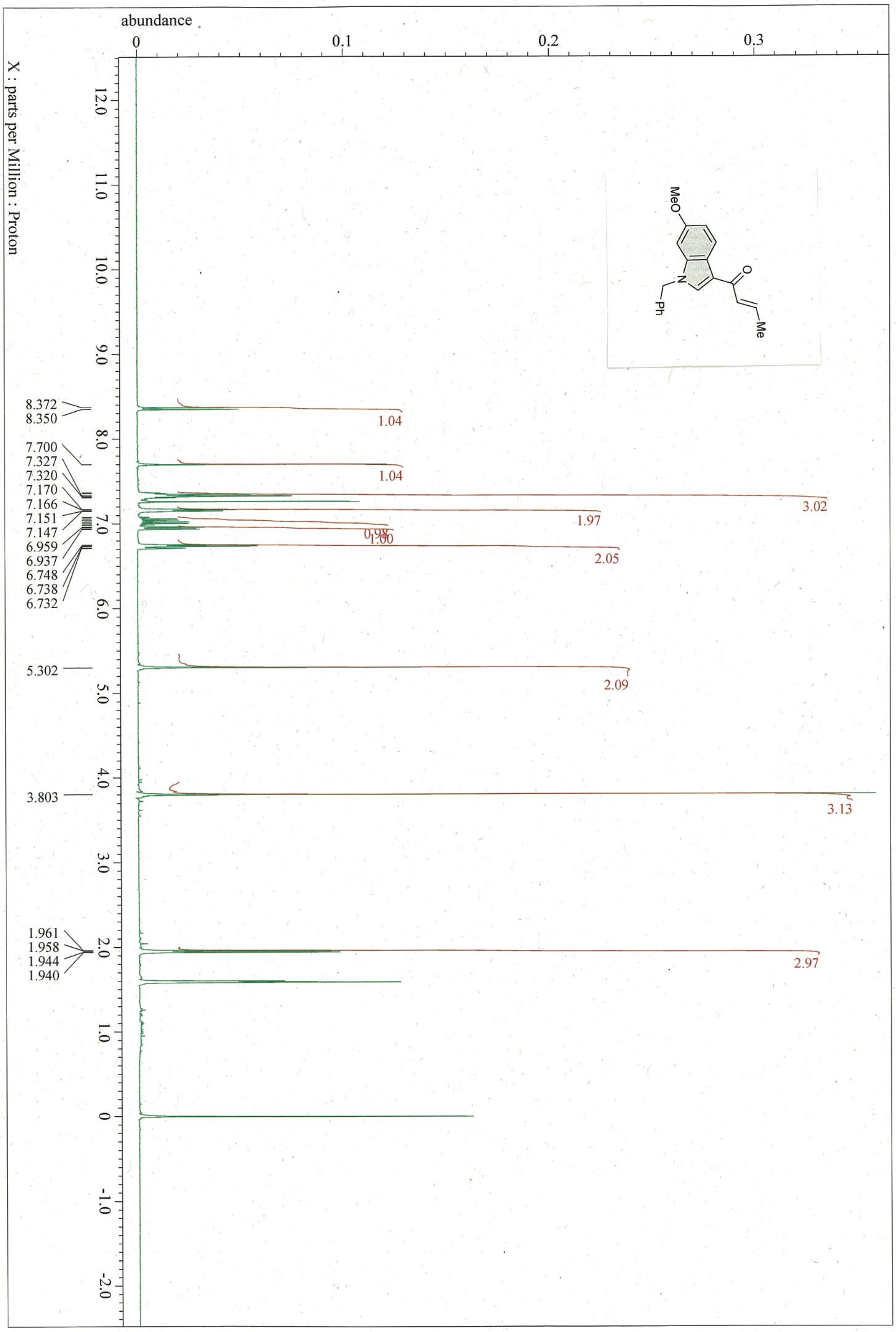
abundance
X : parts per Million : Proton
MeO
N
Ph
O
Me
8.372
8.350
7.700
7.327
7.320
7.170
7.166
7.151
7.147
6.959
6.937
6.748
6.738
6.732
5.302
3.803
1.961
1.958
1.944
1.940
1.04
1.04
3.02
1.97
0.98
1.00
2.05
2.09
3.13
2.97

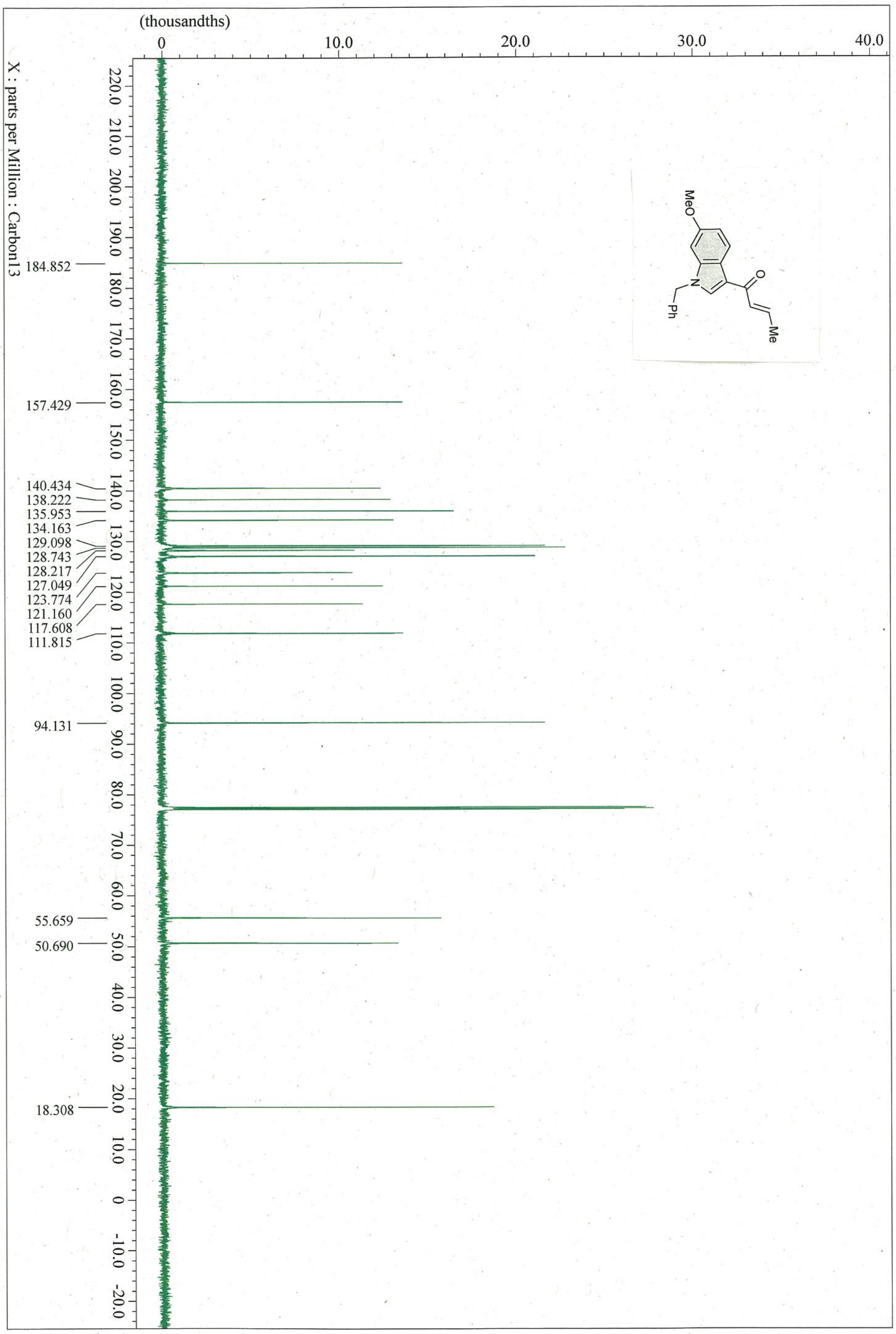
(thousandths)
X : parts per Million : Carbon13
184.852
157.429
140.434
138.222
135.953
134.163
129.098
128.743
128.217
127.049
123.774
121.160
117.608
111.815
94.131
55.659
50.690
18.308
MeO
N
O
Ph
Me

10. References

1 S. Harada, M. Yanagawa and T. Nemoto, *ACS Catal.*, 2020, **10**, 11971.

2 S. Basak, T. Paul and T. Punniyamurthy, *Org. Lett.*, 2022, **24**, 554.

3 J. Tjutrins and B. A. Arndtsen, *J. Am. Chem. Soc.*, 2015, **137**, 12050.

4 T. Okauchi, M. Itonaga, T. Minami, T. Owa, K. Kitoh and H. Yoshino, *Org. Lett.*, 2000, **2**, 1485.

5 V. Vaillancourt and K. F. Albizati, *J. Am. Chem. Soc.*, 1993, **115** , 3499.

6 A. Biswas and R. Samanta, *Eur. J. Org. Chem.*, 2018, **2018**, 1426.

7 H. Ma, X. Xie, P. Jing, W. Zhang and X. She, *Org. Biomol. Chem.*, 2015, **13**, 5255.

8 P. Zhang, S. Xu, S. Wang, X.-J. Zhang and M. Yan, *J. Org. Chem.*, 2024, **89**, 17310.

9 M. W. Ha, M. Lee, S. Choi, S. Kim, S. Hong, Y. Park, M. Kim, T.-S. Kim, J. Lee, J. K. Lee and H. Park, *J. Org. Chem.*, 2015, **80**, 3270.

10 P. Muller and C. Bolea, *Helv. Chim. Acta*, 2001, **84**, 1093.

11 Gaussian 16, Revision C.01, M. J. Frisch, G. W. Trucks, H. B. Schlegel, G. E. Scuseria, M. A. Robb, J. R. Cheeseman, G. Scalmani, V. Barone, G. A. Petersson, H. Nakatsuji, X. Li, M. Caricato, A. V. Marenich, J. Bloino, B. G. Janesko, R. Gomperts, B. Mennucci, H. P. Hratchian, J. V. Ortiz, A. F. Izmaylov, J. L. Sonnenberg, D. Williams-Young, F. Ding, F. Lipparini, F. Egidi, J. Goings, B. Peng, A. Petrone, T. Henderson, D. Ranasinghe, V. G. Zakrzewski, J. Gao, N. Rega, G. Zheng, W. Liang, M. Hada, M. Ehara, K. Toyota, R. Fukuda, J. Hasegawa, M. Ishida, T. Nakajima, Y. Honda, O. Kitao, H. Nakai, T. Vreven, K. Throssell, J. A. Montgomery, Jr., J. E. Peralta, F. Ogliaro, M. J. Bearpark, J. J. Heyd, E. N. Brothers,

K. N. Kudin, V. N. Staroverov, T. A. Keith, R. Kobayashi, J. Normand, K. Raghavachari, A. P. Rendell, J. C. Burant, S. S. Iyengar, J. Tomasi, M. Cossi, J. M. Millam, M. Klene, C. Adamo, R. Cammi, J. W. Ochterski, R. L. Martin, K. Morokuma, O. Farkas, J. B. Foresman and D. J. Fox, Gaussian, Inc., Wallingford CT, 2019.

12 A. D. Becke, *Phys. Rev. A*, 1988, **38**, 3098.

13 K. Fukui, *Acc. Chem. Res.*, 1981, **14**, 363.

14 T. Yanai, D. P. Tew and N. C. Handy, *Chem. Phys. Lett.*, 2004, **393**, 51.
